# Single Crystals of Vanadium Oxides as a Lens for Understanding Structural and Electronic Phase Transformations, Ion Transport, Chemo-Mechanical Coupling, and Electrothermal Neuronal Emulation

**DOI:** 10.1021/acs.chemrev.5c00413

**Published:** 2025-10-23

**Authors:** John Ponis, Shruti Hariyani, George Agbeworvi, Sarbajeet Chakraborty, Victor Balcorta, James Pérez-Vázquez, Benjamin L. Rogers, Yu-Hsiang Chiang, Amanda Jessel, Timothy D. Brown, R. Stanley Williams, Matt Pharr, Xiaofeng Qian, Sarbajit Banerjee

**Affiliations:** † Department of Chemistry, 14736 Texas A&M University, College Station, Texas 77843, United States; ‡ Department of Materials Science and Engineering, Texas A&M University, College Station, Texas 77843, United States; § Laboratory for Battery Science, PSI Center for Energy and Environmental Sciences, Paul Scherrer Institute, Forschungsstrasse 111, CH-5232 Villigen PSI, Switzerland; ∥ Laboratory for Inorganic Chemistry, Department of Chemistry and Applied Biosciences, 27219 ETH Zurich, Vladimir-Prelog-Weg 2, CH-8093 Zürich, Switzerland; ⊥ Department of Mechanical Engineering, Texas A&M University, College Station, Texas 77843, United States; # 1105 Sandia National Laboratories, Livermore, California 94550, United States; ⊗ Department of Electrical Engineering, Texas A&M University, College Station, Texas 77843, United States

## Abstract

Vanadium oxides cystallize in a diverse array of structures and compositions arising from the redox versatility of vanadium, variable covalency of V–O bonds, and myriad coordination geometries. Their open frameworks present abundant interstitial sites that enable insertion of guest-ions. In such compounds, V3*d* electron and spin localization and disorder couple strongly to structural preferences. The rich structural diversity manifests as a “rugged” free energy landscape with multiple interconvertible polymorphs. Such a landscape sets up structural, electronic, and magnetic transitions that underpin the promise of these materials as ion-insertion battery electrodes; compact primitives for brain-inspired computing, and heterogeneous catalysts. Here, we examine the structural and compositional diversity, electronic instabilities, defect dynamics, structure transformations, mechanical properties, and surface structure of vanadium oxides using single crystals as a distinctive lens. Single crystals enable the measurement of structure–function correlations without the ensemble and orientational averaging inevitable in polycrystalline materials. Their well-defined surfaces further enable examination of facet-dependent reactivity toward molecular adsorbates, ion fluxes, and lattice (mis)­matched solids. We provide a comprehensive account of vanadium-oxide single-crystal studies, from delineation of common structural motifs to single-crystal growth techniques, topochemical modification strategies, mechanisms underpinning electronic instabilities, and implementation as electrothermal neurons and battery electrode materials.

## Graphical abstract

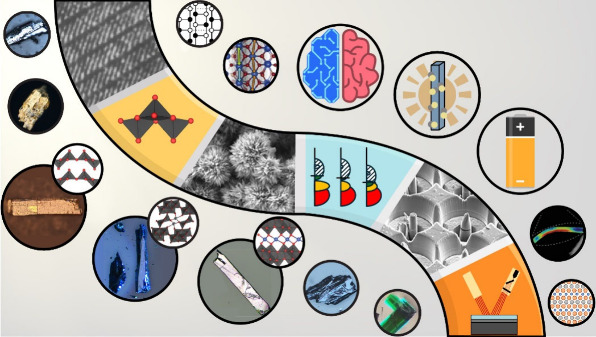

## 1 Introduction

### 1.1 Single Crystals as a Distinctive Lens into the Structure and Dynamics of Condensed Matter

The long-range periodic ordering of atoms in single crystals enables scattering of coherent X-ray, neutron, and electron beams, which allows for the unambiguous resolution of the atomistic structure of periodic solids. Since the advent of crystallography in 1913 with structure solution of the rock salt phase of NaCl,^1^ whenever single crystals are accessible, they have greatly expanded fundamental understanding of atomistic and molecular structure, and have served as a lens for developing structure–function correlations, building chemical intuition of dynamical processes from static images, and for informing targeted strategies for eliciting new and improved function. The earliest direct measurements of magnetic ordering were obtained by neutron scattering from single crystals of MnO and α-Fe_2_O_3_, which confirmed the Néel model of antiferromagnetism.^2^ The 1958 solution of the structure of sperm whale myoglobin from single-crystal diffraction data heralded a new era in macromolecular crystallography.^3^ The last few decades have seen the explosive growth of protein crystallography abetted by the availability of high-brightness X-ray sources, which has transformed understanding of complex biological processes and underpinned modern drug discovery,^4,5^ and indeed shaped much of current molecular biology.

Single crystals have value much beyond just serving as platforms to enable structure elucidation. Quartz single crystals were key to oscillators used for frequency modulation in radios; indeed, the search for alternatives to natural quartz crystals sourced from Brazil during World War II spurred early attempts at scalable single crystal growth that have now become ubiquitous tools in materials science. Similarly, the microelectronics revolution that has been the key driver of information economies over the last century has been enabled by the ability to prepare large single crystals of silicon.^6,7^ In this review, we will emphasize synthetic strategies for the growth of single crystals of binary, ternary, and more complex vanadium oxides and examine the vast structural diversity, dynamical phase transformations, distinctive anisotropic properties, and coupling to external thermal, stress, voltage, electrochemical flux, and magnetic fields that underpin their function in diverse fields such as battery cathodes and anodes, neuronal and synaptic devices, and as electrocatalysts and photocatalysts for energy-relevant catalysis.^8–17^

Much of the value of single crystals derives from the translation symmetry that is one of their key defining characteristics. Long-range order enables extrapolation of the structure and orientation of one region of a specimen given the structure and orientation in a distant region. The ability to precisely describe the positions of a large number of atoms using a small number of variables renders structure solutions from scattering data mathematically tractable. For the dense inorganic solids under consideration here, in contrast to molecules, the structural periodicity of the single crystals further underpins wave-like electronic and phonon states distributed over enormous length scales, which give rise to emergent complexity not manifested in discrete small molecules where the strongest bonding motifs are strongly localized. Quasi-particle states that arise in crystalline vanadium oxides underpin emergent complexity manifested as spin- and charge ordering, superconductivity, metal–insulator transformations, and stabilize a variety of exotic strongly correlated phases with closely coupled spin, charge, orbital, lattice, and atomic degrees of freedom.^8,18–21^

The mathematical treatment of crystallography yields crystal structures as snapshots of atomic arrangements configured according to precise symmetry relationships with some perspective of disorder derived from partial or mixed occupancies and the magnitude of thermal ellipsoids. Yet such static images can yield detailed insights into possible mechanisms of dynamical transformations and can provide unprecedented insight into pathways for structural transformations spanning the range from diffusionless Martensitic transformations to ion-transport-induced reconfiguration.^22–24^ The ability to align reactive surfaces with those of other reagents without interrupting crystalline order yields a rich class of single-crystal-to-single-crystal transformations that are yielding unprecedented insight into ion transport pathways, sequence of interstitial sites occupied by guest ions, structural distortions induced by said distortions, and reaction mechanisms.^23,25–31^ In this review, we examine the role of single crystals as a lens for understanding order–disorder and electronic structure transitions, and for investigating the structural effects of ion exchange reactions in periodic solids. Unlike polycrystalline films or powders, single crystals are a distinctive platform for enabling single-entity studies without ensemble averaging across populations of different materials with diverse internal states and thus provide a means of interrogating intrinsic material properties with high fidelity (Figure 1). For anisotropic crystal symmetries, single crystals enable examination of field coupling to properties and observation of a stimuli-induced response without orientation averaging. Notably, as in the radio frequency modulation example, single crystals present individual domains through which electromagnetic radiation as well as phonons and charge carriers can be coherently propagated.

**Figure 1:**
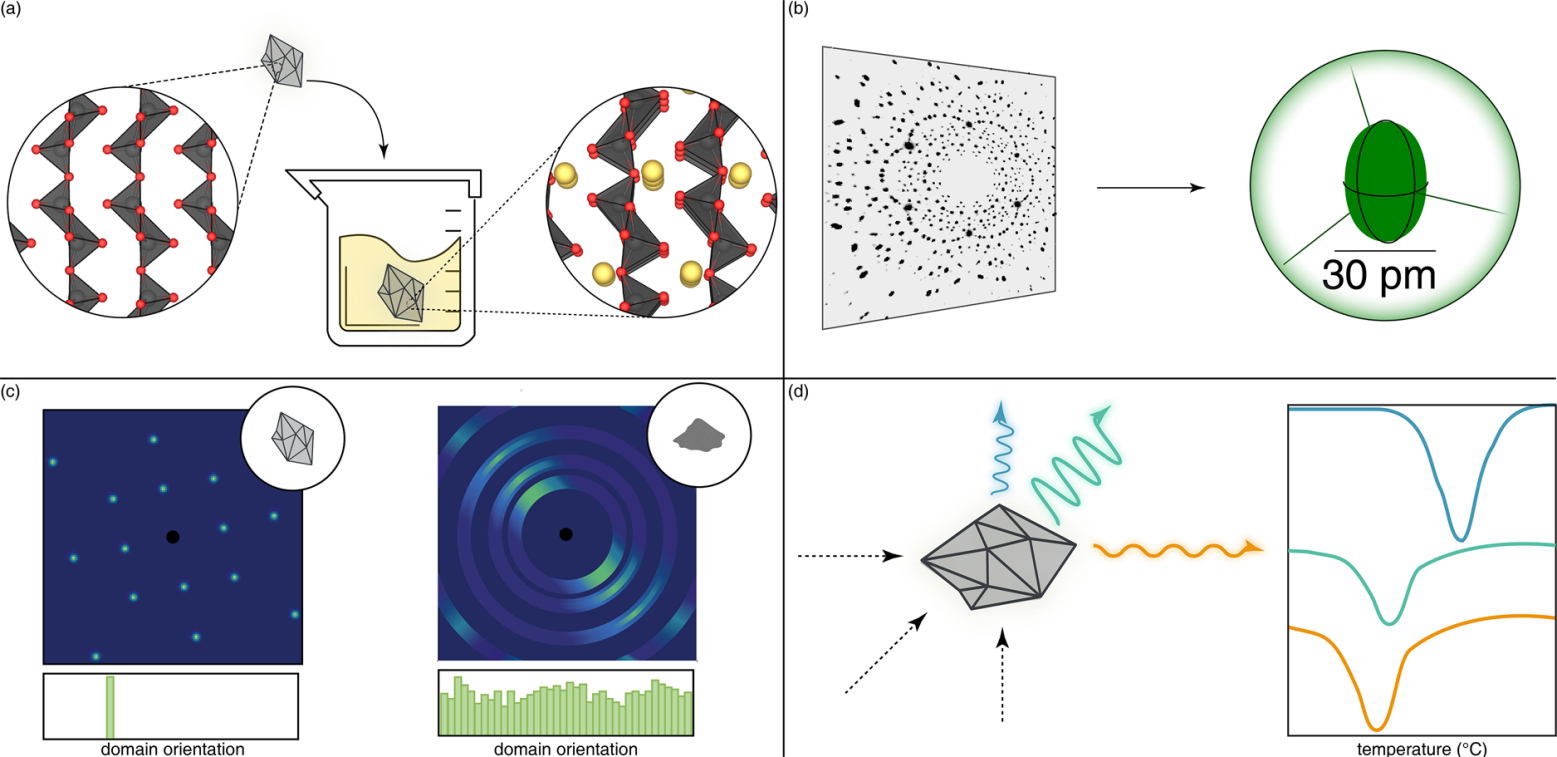
Distinctive science enabled by single crystals. (a) Single-crystal-to-single-crystal transformations provide insights into dynamics of structural, electronic, and magnetic transitions; (b) high-resolution atomic structure determination, (c) measurement of intrinsic material properties without ensemble averaging inherent in polycrystalline materials; and (d) anisotropic direction- and angle-resolved response to external stimuli without orientational averaging.

Drawing upon a plethora of single-crystal studies that have become available in the literature, we focus primarily on vanadium oxides given the wealth of structural diversity, their “rugged” free energy landscapes that lends itself to myriad interconvertible metastable phases^10^ and their ability to serve as a rich “sandbox” for strongly correlated electron systems where systematic variations in structure and composition can engender decoupling of transformation characteristics and thus illuminate design principles for modulating otherwise challenging correlated electron systems.

#### 1.1.1 A Note on Polymorph Naming

Among the vanadium oxides, the pentoxide family (V_2_O_5_ polymorphs and M_*x*_V_2_O_5_ derivatives) adopt a uniquely vast set of structures and compositions, many of which share structural motifs that will be discussed in detail below. Progress in exploring the synthetic space has been sporadic over the last century, with contributions from solid-state chemists, condensed matter physicists, materials scientists, and geologists. The naming of newly discovered materials has been left to their discoverers and has proceeded according to several different conventions. Therefore, Greek letter designations often correspond between binary polymorphs and ternary analogues but sometimes do not. For example, ternary α-M_*x*_V_2_O_5_ compounds (e.g., α-NaV_2_O_5_ and α-Li_*x*_V_2_O_5_)^32^ consistently feature a common α-V_2_O_5_ framework structure. By contrast, the layered structure of the binary β-V_2_O_5_ ^33^ is unrelated to the tunnel structure shared among the large family of β- and β′-M_*x*_V_2_O_5_ compounds, wherein β and β′ designate the site occupied by the ion M, respectively (*x*, 0.5, *z*) and (*x*, 0, *z*), Wyckoff position 4i in both cases.^34,35^ Additional complications arise when a material is named before its structural similarity to other named phases is understood. This is especially the case for the so-called double-layer bronzes. The parent binary polymorph λ-V_2_O_5_ ^36^ (previously also designated as ε′-V_2_O_5_ by comparison to ε-Cu_0.9_V_2_O_5_ from which it was first synthesized^37^) adopts several different layer stacking angles depending on the intercalated guest ion identity and concentration. Structures in this family have been designated δ-, ε-, μ-, ν-, ν′-, τ-, and λ-M_*x*_V_2_O_5_ according to the coordination geometry of the guest ion (or unit cell parameters if no structure solutions were yet known).^36,38,39^ These are not to be confused with the unrelated δ-Li_*x*_V_2_O_5_ and ε-Li_*x*_V_2_O_5_, which feature a distorted α-V_2_O_5_ backbone visible in Figure 50.

Greek letter designations relate to crystal symmetry only indirectly. The crystal system of a ternary bronze is typically dictated by its V_2_O_5_ framework, and is thus shared with the corresponding binary compound, but guest ions frequently alter space group symmetry. For example, insertion of small amounts of lithium into α-V_2_O_5_ to form α-Li_0.1_V_2_O_5_ preserves the *Pmmn* space group, whereas distortions induced at higher lithium concentrations generate the space groups *P2*_*1*_ *mn* for ε-Li_*x*_V_2_O_5_ and *Amma* for δ-Li_1_V_2_O_5_ (see Section 5.2.1).^40^ Nearly all of the tunnel-structured β-M_*x*_V_2_O_5_ ternary bronzes share the parent ζ-V_2_O_5_ material’s *C2/m* space group, but Na-ion ordering below 230 K in β-Na_0.33_V_2_O_5_ generates a 1 × 2 × 1 supercell with the space group *P2*_*1*_ */m* (see Section 3.5.2).

To resolve some of the ambiguity which confronts those unfamiliar with the V_2_O_5_ bronzes, we have organized M_*x*_V_2_O_5_ materials into Table 1 according their shared parent structure. Structural descriptions of several of these materials follow in this work. We further point the reader to systematic descriptions in terms of V_2_O_5_ framework structure published in the literature, albeit these conventions have not been rigorously followed.^32,40^

**Table 1:** Binary V_2_O_5_ Polymorphs and Corresponding Ternary Materials^a^

Parent Structure	Ternary Examples	Prominent V_2_O_5_ lattice features
α-V_2_O_5_ ^32^	α-Li_*x*_V_2_O_5_ ^32^ (*Pmmn*), ε-Li_*x*_V_2_O_5_ ^32^ (*P2*_*1*_ *mn*), δ-Li_*x*_V_2_O_5_ ^32^ (*Amma*), α-NaV_2_O_5_ ^32^ (*Pnma*)	Layers made of zigzag chains of edge-shared square pyramids
(*Pmmn*)		
ζ-V_2_O_5_ ^41^	β-Na_0.33_V_2_O_5_ ^38^ (*C2/m*), β′-Cu_*x*_V_2_O_5_ ^38^ (*C2/m*), β-K_*x*_V_2_O_5_ ^38^ (*C2/m*)	Tunnels formed from V_2_O_10_ units and zigzag square pyramid chains
(*C2/m*)		
γ′-V_2_O_5_ ^27^	γ-Li_*x*_V_2_O_5_ ^32^ (*Pnma*), γ-Na_*x*_V_2_O_5_ ^42^ (*Pnma*), γ-K_*x*_V_2_O_5_ ^43^ (*Pnma*), γ-Zn_*x*_V_2_O_5_ ^44^ (*Pnma*)	Similar to α-V_2_O_5_, with every other zigzag chain inverted and canted
(*Pnma*)		
λ-V_2_O_5_ ^36^	ε-Cu_0.9_V_2_O_5_ ^32^ (*C2/m*), τ-Cu_*x*_Ag_*y*_V_2_O_5_ ^45^ (*C2/m*), λ-Li_*x*_V_2_O_5_ ^36^ (*C2/m*), δ-Pb_0.5_V_2_O_5_ ^46^ (*P* 1)	Double-layers made of edge-shared V_4_O_20_ units
(*C2/m*)		
β-V_2_O_5_ ^47^	Tetramethyalmmonium-V_8_O_20_ ^48^ (*C2/m*)	Double-layers made of corner-shared V_4_O_20_ units
(*P2*_*1*_ */m*)		
δ-V_2_O_5_ ^47^		Rutile slabs connected by corner-sharing
(*C2/c*)		

a Space groups indicated in parentheses.

### 1.2 Origins of Structural Diversity, Abundant Polymorphism, and Phase Transformations in Vanadium Oxides

The vanadium oxides exhibit a vast array of compositions and structures. Vanadium adopts a wide range of readily accessible oxidation states in oxides, ranging from V^2+^ (3*d* ^*3*^ electron configuration) to V^5+^ (3*d* ^*0*^), enabling a dense population of vanadium–oxygen stoichiometries—even lower vanadium oxidation states can be accessed in mixed valence compounds. Differences in vanadium–oxygen ratio between two phases necessitate differences in vanadium–oxygen connectivity and thus crystal structure. It is noteworthy that the redox promiscuity of both vanadium and oxygen enable a diversity of tetrahedral, square pyramidal, and octahedral local environments with varying extents of distortion. The phase diagram in Figure 2a presents a survey of binary structures according to vanadium–oxygen stoichiometry. Many neighboring phases can be generated by the application of simple rules relating composition and structure. The Magnéli and Wadsley–Roth phases (Figure 2b,c, respectively) are families of homologous materials related by such rules and will be discussed in more detail below. For several stoichiometries (such as V_2_O_3_, VO_2_, and V_2_O_5_ in Figure 2a) multiple polymorphs are known. In general, one of these structures will represent the minimum-energy (thermodynamically stable) atomic arrangement at the given composition and a specific temperature or pressure, whereas the rest are metastable but might represent the most stable configuration under a different set of state variables (e.g., temperature, pressure, surface stress).^10,49–52^ The large number of known metastable phases is a defining characteristic of vanadium oxides and reflects its “rugged” free energy landscape resulting from the facile valence fluxionality of vanadium and oxygen and their ability to bond in many different ways and to stabilize myriad local coordination environments.

**Figure 2:**
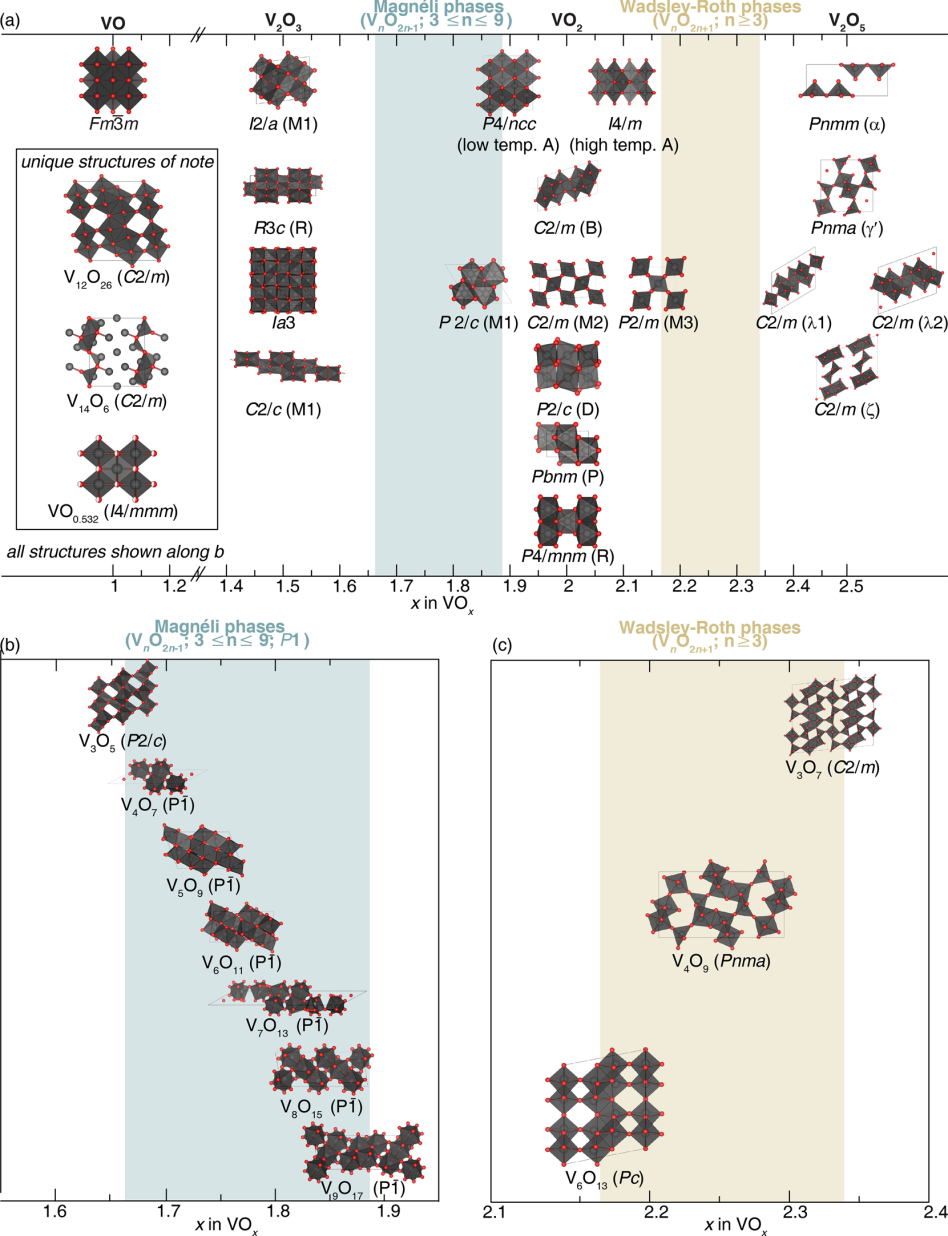
Schematic illustration of selected vanadium oxide phases as a function of oxygen stoichiometry. (a) Several distinct V_2_O_5_ polymorphs are shown whose structure is described based on connectivity of VO_5_/VO_6_ subunits below. The inset shows distinctive structures. The shaded blue and yellow regions delineate Magnéli and Wadsley–Roth phase regimes. A closer look at the (b) Magnéli and (c) Wadsley–Roth phases as a function of oxygen stoichiometry. Each crystal structure is shown along *b*.

For any given spatial arrangement of atoms and set of state variables (temperature, pressure, electromagnetic field, etc.), interparticle Coulombic interaction energies sum to a total enthalpy value. Addition of atoms to the otherwise fixed arrangement contributes new positional degrees of freedom and alters the enthalpy by the introduction of new interaction terms. As we shall see below, large families of vanadium oxides exhibit common idealized parent structures, within which differences arise from number and ordering of vacancies and resulting structural distortions. Notionally, we can collapse the numbers and positions of vacancies into respective composition and structure axes to construct a proxy slice of the free energy landscape, as depicted schematically in Figure 3. Stable and metastable phases occupy energy minima (red-shaded regions).

**Figure 3:**
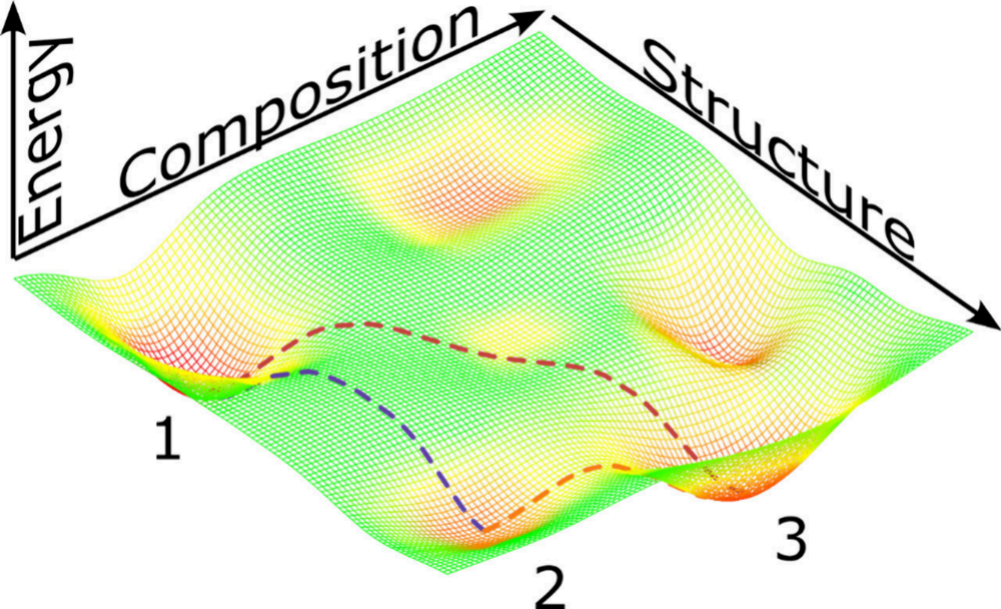
Schematic rendition of composition-structure free-energy landscape. Red regions represent (meta)­stable phases at (local) energy minima, whereas dashed lines represent transformation trajectories between phases.

In general, during synthesis, atoms are free to explore the free energy landscape depending upon the energy with which they begin their descent. Systems with sufficient energy to overcome kinetic barriers (such as in conventional high-temperature ceramic processing) will migrate down energy gradients with respect to composition (i.e., by exchanging atoms with their environment) and/or structure (i.e., by distorting or breaking bonds and through successive nucleation/dissolution phenomena) axes, constraining motion along the free energy landscape and limiting the set of possible configurations that can be accessed. To obtain metastable polymorphs, synthetic methods can either adopt a specific reaction trajectory that deposits the material at a certain local minima or can isolate a regime where the metastable polymorph is the global minimum whereupon the structure can be retained by rapid quenching to ambient conditions.^10,52^

At a fixed composition, heating a metastable structure above a critical temperature will transform into a more stable one (e.g., **2**→**1** along the purple trajectory), and the reverse reaction (**1**→**2**) will not occur spontaneously. Coupling with external variables such as pressure, temperature, or chemical potential, however, effectively contributes additional axes to the parameter space and can alter the relative energy of points in the composition–structure plane. By leveraging these external couplings, the free energy landscape can be navigated to converge on a metastable phase of interest. For example, a metastable phase may be prepared by first synthesizing a stable phase with a similar structure via a thermodynamically favorable composition modification (e.g., oxidation by calcination in air at high temperature, **1**→**3**), and then reverting to the desired composition under mild reaction conditions in order to preserve the new structure (e.g., oxygen abstraction by reduction in aqueous solution, **3**→**2**). Several V_2_O_5_ polymorphs only form spontaneously at high pressure and temperature but can be isolated at room temperature by rapid quenching.^47^

Guest ions, defined here to be residing in interstitial sites of otherwise stable or metastable parent frameworks, introduce additional structural and chemical complexity. Each additional ion contributes a new dimension to the space of possible compositions, and new types of bonds will couple energy to structure distortions differently. Incorporation of guest ions to form ternary (or higher-order) oxides is thermodynamically driven by electron transfer to electronegative vanadium centers, which in part explains the preponderance of ternary oxides in the [V/O > 2] composition range. Typically, a guest ion’s radius and formal charge do not match those of the host vanadium ion, so guest ions occupy structurally distinct positions in the crystal structure and thus often enforce new V–O frameworks not exhibited by binary oxides. In many cases where guest ion sites in a ternary oxide correspond to vacant interstices in a binary oxide structure, one phase may be converted to the other by an appropriate topochemical (structure-preserving) ion insertion or removal reaction. Electrochemical variations of such topochemical reactions form the basis for energy storage technologies as first outlined in Whittingham’s seminal work on reversible Li-ion insertion in α-V_2_O_5_.^53–55^ Several V_2_O_5_ polymorphs have only been stabilized in this way. These structure transformations are discussed in detail in Section 5.

We start this review by a detailed discussion of structural relationships between binary and ternary vanadium oxides, and briefly describe the experimental utility of single crystals in the remaining part of Section 1. Section 2 presents a detailed overview of single crystal growth methods and their technical considerations, particularly as they pertain to vanadium oxides. In Section 3, we begin our exploration of transformations by examining the atomic–electronic structure relationships that underpin the vanadium oxides’ wealth of electronic phase transitions. Section 4 examines the utility of electronic transitions for instantiating brain-like computing. Section 5 discusses topochemical transformations: composition transformations which preserve the structure of the host material. Section 6 describes crystal surfaces and interfaces, and presents heterogeneous catalysis as an example of controlling material behavior by modifying interfaces. The mechanical properties of transformed crystals—and methods for measuring them—are described in Section 7. Finally, in Section 8, we offer our outlook on the various topics covered previously.

#### 1.2.1 Binary Vanadium Oxides and their Structural Relationships: A Broader “Top Down” View

Despite the structural variety present among the vanadium oxides, similarities are readily apparent if we take a “top-down” view, considering individual phases as structural deviations from a simple shared parent lattice. Most binary vanadium oxide structures can be idealized as close-packed planes of oxygen anions stacked to form a hexagonal (HCP) or face-centered cubic (FCC) anion lattice with stoichiometric variation accommodated by ordered occupancy of a subset of octahedral (and occasionally tetrahedral) cation sites by vanadium ions.^56^ For example, VO exhibits the rock salt structure with all octahedral sites within the FCC oxygen lattice occupied by vanadium, whereas the spinel structure of V_3_O_4_ results from filling half of the octahedral and a quarter of the tetrahedral sites (Figure 4a,b).^57^ Larger vanadium deficiencies such as in VO_2_ (B) and α-V_2_O_5_, wherein a close-packed anion lattice could not be fully coordinated by the sparse cation framework, result in vacancies on 1/9 and 1/6 of oxygen sites, respectively (pink atoms in Figure 4c,d). A particular subset of the ordered-vacancy FCC vanadium oxides is the Wadsley–Roth phases (Figure 2c). Commonly found among early transition metal oxides, Wadsley–Roth structures are generated by subdividing a hypothetical ReO_3_-type structure featuring only corner-shared octahedra into blocks and shearing along the boundaries to create corner-shared planes.^58,59^ Rock-salt VO also serves as the parent structure for a series of suboxides VO_*x*_ for *x* < 1, which form by the ordering of oxygen vacancies at oxygen stoichiometries as low as *x* = 3/7 (inset in Figure 2a).^60,61^

**Figure 4:**
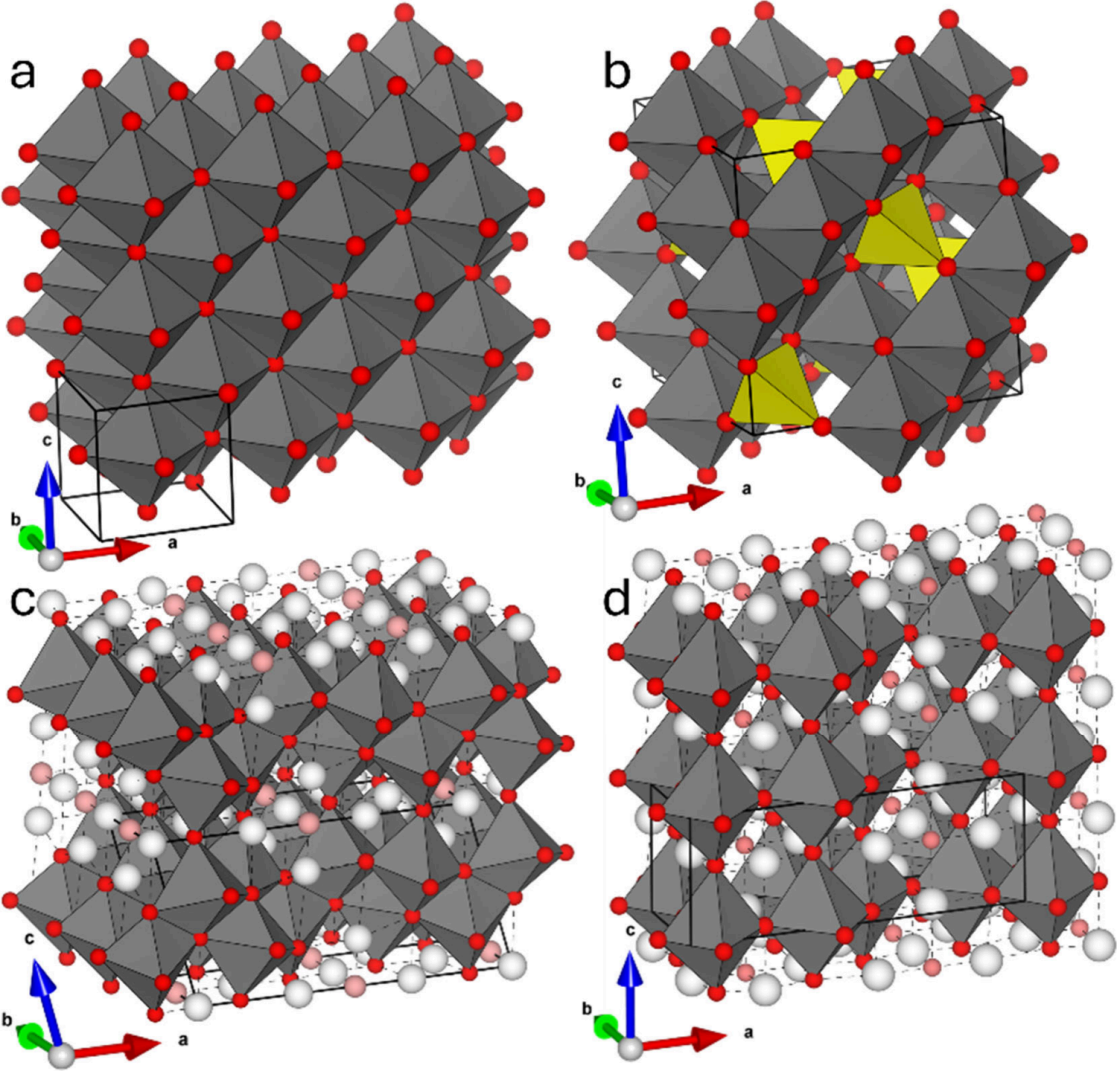
Comparison of binary vanadium oxides with FCC anion lattices. (a) The parent rock salt VO structure with V atoms in all octahedral sites. (b) Spinel V_3_O_4_ structure, with VO_4_ tetrahedra highlighted in yellow. (c,d) VO_2_ (B) and α-V_2_O_5_, with V and O vacancies in the rock salt parent lattice colored white and pink, respectively.

Vanadium oxides also form a family of homologous Magnéli phases flanked by the stoichiometric end-members (corundum) V_2_O_3_ and (rutile) VO_2_.^56^ These materials share a hypothetical nickelite-type VO parent structure, with vanadium ions filling all octahedral interstices in a HCP oxygen lattice. These structures can be generated from rutile VO_2_ by deleting the oxygen from every *n* ^th^ (211) VO plane, shuffling the vanadium atoms into adjacent layers, and shearing the structure perpendicular to the plane of oxygen vacancies to concatenate the new V- and O-terminated surfaces and re-establish octahedral coordination. Deletion of one O for every *n* VO_2_ in this way yields a stoichiometry of V_*n*_O_2*n*–1_, bounded by V_2_O_3_ for *n* = 2 and by VO_2_ as *n*→∞. Structurally, each Magnéli phase effectively comprises individual “layers” of V_2_O_3_ separated by *n*-2 “layers” of VO_2_, as shown in Figure 5a for the case of V_7_O_13_.

**Figure 5:**
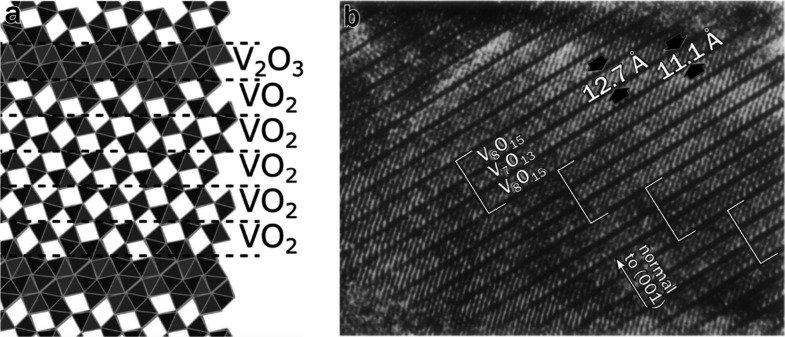
(a) Structure of V_7_O_13_ subdivided into VO_2_ and V_2_O_3_ layers. (b) Multibeam tunneling electron micrograph of microsyntactic intergrowth of V_7_O_13_ and V_8_O_15_. (b) was adapted with permission from ref 62. Copyright 1982 Elsevier.

At high values of *n*, differences in stoichiometry and formation enthalpy diminish and entropic contributions increase in significance, leading to a densely populated VO_*x*_ phase diagram and closely clustered local free energy minima in the vicinity of VO_2_. The ruggedness of the vanadium oxide free energy landscape makes the Magnéli phases extremely difficult to isolate as pure compounds. Their structural and compositional similarity imbue a strong tendency to form microsyntactic crystalline intergrowths (Figure 5b) between neighboring members,^62^ whereas their overall metastability leads to disproportionation into thermodynamically stable compositions upon heating as a result of oxygen vacancy diffusion along concentration gradients. The Magnéli phases are exemplary of the transformation behavior exhibited by vanadium oxides. All members except V_7_O_13_ exhibit metal–insulator transitions, whose behavior varies due to differences in interactions between electron states localized within VO_2_- and V_2_O_3_-structured regions, as discussed in Section 3.

#### 1.2.2 Binary Vanadium Oxides and their Structural Relationships: A Localized “Bottom Up” View

Although the “top-down” perspective of structure relationships of vanadium oxides outlined above is useful for identifying general composition–structure relationships, many of their dynamic physical and chemical properties emerge from local spatial relationships and electron interactions between structural subunits. These subunits are in turn generated from repetition of certain bonding motifs between VO_6_ octahedra or VO_5_ square pyramids, which form the base of the hierarchy of structural complexity. Zavalij and Whittingham have developed a thorough systematic description of the bonding motifs common among the vanadium oxides and their relationships to crystal structure and symmetry.^40^ What follows is a simplified discussion of three of those features that underpin the transformation behavior discussed in this work.

Many vanadium oxides feature infinite linear chains of edge-shared VO_6_ octahedra. In rutile VO_2_ (Figure 6a), the VO_6_ chain is offset by *c/*2 and rotated 90° about *c* to connect the apical corners of each chain to the shared edges of two of its neighbors, creating a distorted checkerboard pattern when viewed along *c* as in Figure 5a above. The vacant octahedral sites in the HCP oxygen lattice form tunnels along *c* connected laterally along *a* and *b* by tetrahedral interstices, facilitating the insertion and diffusion of guest ions.^63^ The proximity of vanadium ions within a chain allows their *t*_*2g*_ orbitals to overlap across the shared edge, whereas weak lateral interactions via corner-sharing (or no direct bonding) between adjacent chains confine the resulting collective states to one dimension. As we shall discuss in Section 3, dimensional confinement of vanadium 3*d* electron bands underpins the well-known metal–insulator transition behavior of VO_2_ and has been posited to explain transport anomalies in other vanadium oxides bearing this fundamental structure motif.^64^

**Figure 6:**
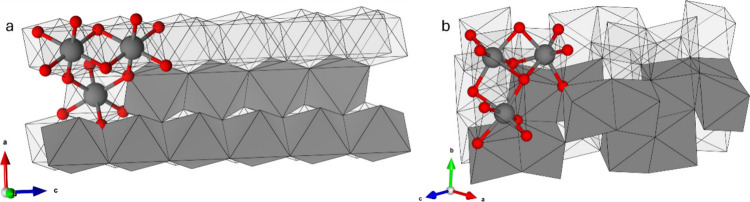
Extended patterns of VO_6_ octahedral edge and face sharing in (a) rutile VO_2_ (b) V_2_O_3_. Vanadium ions rendered in gray, oxygen in red.

Corundum-structured V_2_O_3_ (Figure 6b) comprises pairs of VO_6_ octahedra connected by shared faces perpendicular to the stacking direction of the close-packed planes in the HCP oxygen lattice. Each pair links with its neighbors laterally by shared edges. This motif is expressed in all of the Magnéli phases but is rare for VO_*x*_ compounds with [V/O > 2], as octahedral face sharing is impossible in the FCC anion lattices preferred at these stoichiometries because all octahedral faces are shared with tetrahedral vacancies. Because of the high octahedral site filling, octahedral vacancies do not form linear tunnels but are connected through pairs of empty tetrahedral sites; therefore, the mobility of guest ions in V_2_O_3_ is typically low (see Section 5). Short V–V distances between face-shared neighbors enforce strong interactions between their *d*-orbital systems. Although the distribution of octahedral vacancies ensures that only two VO_6_ octahedra connect by either edge- or corner-sharing in any direction (rather than forming infinite chains), V_2_O_3_ and the Magnéli phases are rich with electronic phase transitions.

The 3*d* ^0^ V^5+^ ion introduces additional structural complexity that bears further analysis and detailed discussion. We will examine first commonly observed features of V^5+^ coordination and bonding motifs in binary oxides before moving on to ternary and higher-order bronzes. The radius ratio for *r*(V^5+^)/*r*(O^2–^) based on Shannon ionic radii sits below the minimum threshold for stable octahedral coordination (Table 2).^65^ V^5+^O_6_ (and frequently V^4+^O_6_) octahedra are thus heavily distorted toward square pyramidal coordination, with V^5+^ ions displaced out of the basal plane toward the apical oxygen, as shown in Figure 7a. This proximity (among the shortest bonds in transition metal oxides),^66^ combined with the electronegativity of the vanadium ion, imparts significant covalency, and indeed double-bond character, to the “vanadyl” V–O_V_ interaction.^41,67^ Breaking of octahedral symmetry renders the interaction between polyhedra sensitive to which edge or corner is shared, so polyhedral orientation contributes to the hierarchy of complexity in vanadium oxides. Vanadyl oxygens usually coordinate to only one vanadium ion, disfavoring sharing along the associated superior axial polyhedral edges (orange), and thereby typically forming an interstice opposite the V–O_V_ bond. The connectivity and dimensionality of this interstitial site thus depend on the relative orientation of vanadyl bonds, dictated in turn by the connectivity of polyhedral subunits.

**Table 2:**
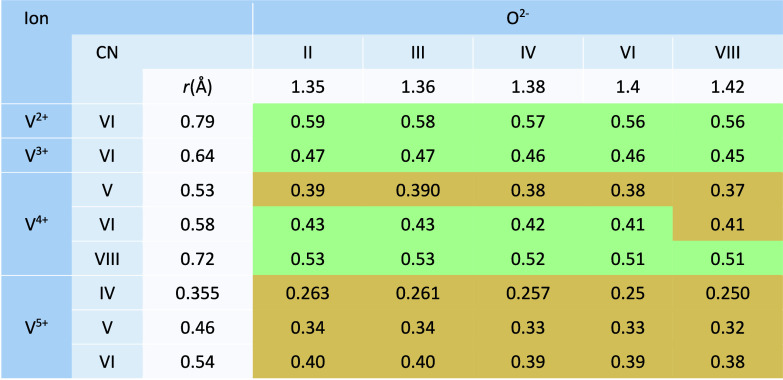
Radius Ratios for V^*n*+^ and O^2–^ Ions by Coordination Number^a^

a Green and orange values lie respectively above and below the minimum ratio for octahedral coordination. (CN: coordination number) Ionic radii values from ref 68.

**Figure 7:**
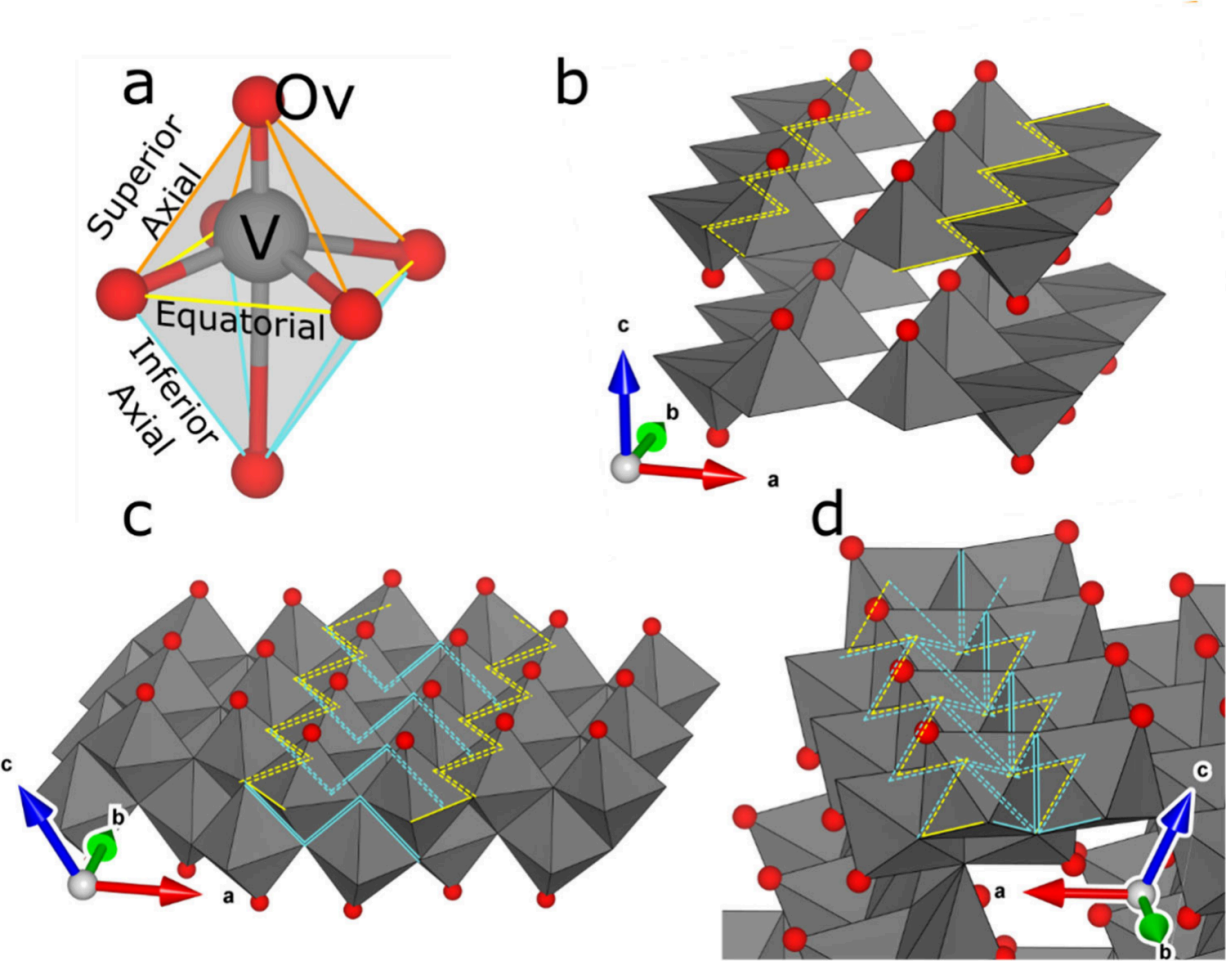
Structures formed by sharing of specific VO_6_ octahedral edges. (a) a single VO_6_ octahedron with superior axial, equatorial, and inferior axial edges colored orange, yellow, and blue, respectively. (b) Antiparallel equatorial edge-sharing in α-V_2_O_5_. The contrast between strong V–O bonding within layers and weak van der Waals bonding between them is responsible for the material’s pronounced anisotropy, the mechanical consequences of which are discussed in Section 7. (c) Parallel equatorial edge-sharing in λ-V_2_O_5_ (d) Mixed edge-sharing between axial and equatorial edges in ζ-V_2_O_5_.

In several materials, VO_5_/VO_6_ polyhedra share equatorial basal plane edges (yellow) to form extended zigzag chains.^40^ In α-V_2_O_5_, adjacent parallel chains connect through shared corners (Figure 7b) to form sheets featuring antiparallel sets of vanadyl bonds oriented toward planar interstices. The flexibility of the single layers derives from the corner-shared connectivity, which allows α-V_2_O_5_ layers to pucker upon intercalation of cations into the van der Waals’ gap (stabilizing γ-LiV_2_O_5_, which can be delithiated to stabilize the puckered polymorph denoted as γˈ-V_2_O_5_), engendering a series of structural phase transitions. As in the case of linear edge-shared chains in VO_2_, corner-sharing does not support strong V 3*d–*3*d* electronic state interactions between adjacent chains. Weak interactions between layers give rise to quasi-one-dimensional electronic states. Double layered λ-V_2_O_5_ (Figure 7c) likewise exhibits a layered structure, albeit with sharing of superior axial edges between stacked pairs of chains within each layer.^36^ Increased lateral intralayer bonding rigidifies the λ-V_2_O_5_ host lattice against distortion upon ion insertion. Nonetheless, the anisotropy of the physical properties of λ-V_2_O_5_ derivatives suggests one-dimensional band formation akin to α-V_2_O_5_. Edge-shared zigzag chains appear in several other configurations, including separated hexagonal pipes in Cs_0.3_V_2_O_5_,^69^ hexagonal tunnels with triply shared corners in Cs_0.35_V_2_O_5_,^70^ and as isolated chains in NaVO_3_•1.8H_2_O,^71^ although the physical properties of these materials have not been studied extensively.

Chains constructed from repeating V_4_O_16_ units are also commonly observed in V_2_O_5_ polymorphs and related materials. In each V_4_O_16_ unit, pairs of octahedra share inferior axial edges along one direction and share an inferior axial edge with an equatorial edge along the chain direction. These chains can then corner-share in several ways to form larger structures. In high-pressure β-V_2_O_5_, each chain shares two corners per V_4_O_16_ unit with its neighbors to give two-dimensional sheets separated by a van der Waals gap,^33^ whereas in ζ-V_2_O_5_ the interlayer space between similar sheets (with chains connected by a single corner) is divided into one-dimensional tunnels by corner sharing with α-V_2_O_5_-like zigzag chains (Figure 7d).

#### 1.2.3 Ternary Vanadium Oxides from Cation Insertion in Interstices

The tendency for vanadium oxides to form interconnected interstitial sites is apparent from both the top-down view of their structure relationships, by which they are described as extended patterns of densely ordered vacancies, and the bottom-up view, wherein they arise from a high degree of corner sharing between structure motifs with vanadyl oxygen atoms constituting the majority of Lewis basic sites that bind cations in the interstitial sites. Lower vanadium stoichiometries create more vacancies and allow for more variety and higher connectivity between interstitial sites. These interstices enable vanadium oxides to host a wide range of cation guest species and vastly expand the range of accessible compositions, spanning across s-, p-, and d-block (as well as organic) guest ions. V_2_O_5_ exhibits a particularly broad polymorphism, with each member able to host cations ions from across the periodic table, a phenomenon discussed at length in Section 5.

### 1.3 Single Crystals as Platforms for Investigating Structure–Function Correlations

The term “single crystal” is used with different meanings across different scientific disciplines. We adopt here a functional definition, to include any measured single-domain entity whose orientation-dependent properties are resolved experimentally. We thus exclude polycrystalline aggregates regardless of size, as well as measurements of single-domain entities which do not resolve orientation-dependent properties. We also narrow our discussion to exclude polyoxovanadate and oxovanadium­(IV) molecular crystals, as their chemical and electronic properties are largely determined by the structure of the individual molecules and clusters rather than emerging from their collective interactions within a solid matrix.

Several characteristics of single crystals render them uniquely valuable for studying material properties. Polycrystalline samples feature a large number of surfaces and interfaces between crystalline domains. Interfaces generally have different atomic structure and possibly even composition than the bulk material, and will thus exhibit different chemical, mechanical and transport properties whose contribution to the material’s overall behavior can be difficult to assess and highly variable across different orientations of grain boundaries. A single crystal, on the other hand, has a uniform bulk structure and composition (neglecting inevitable defects) with a small number of well-defined interfaces. Thus, measurements of single crystals can interrogate and distinguish surface and bulk effects without ensemble averaging (Figure 1). Notably, from a functional perspective, grain boundaries most often scatter and decohere electromagnetic radiation, phonons, and ions, in an oftentimes unpredictable manner degrading intrinsic transport properties of materials.

Polycrystalline material properties are also subject to statistical variation in the size, geometry, composition, stress environment, strain state, chemical environment, and thermal and electrical contact of individual domains. Except in rare circumstances, the relative orientations of crystalline domains within an aggregate specimen will exhibit some statistical variation. Measurements which involve the application and/or observation of an anisotropic external field will be averaged over the distribution of orientations so that anisotropic properties cannot be directly resolved. By contrast, single crystals allow precise control of the alignment of input and output fields with respect to crystal axes, so that angular dependencies can be associated with specific structural motifs without orientational averaging (Figure 1). We will briefly discuss several such techniques sketched in Figure 8 as they pertain to single crystals.

**Figure 8:**
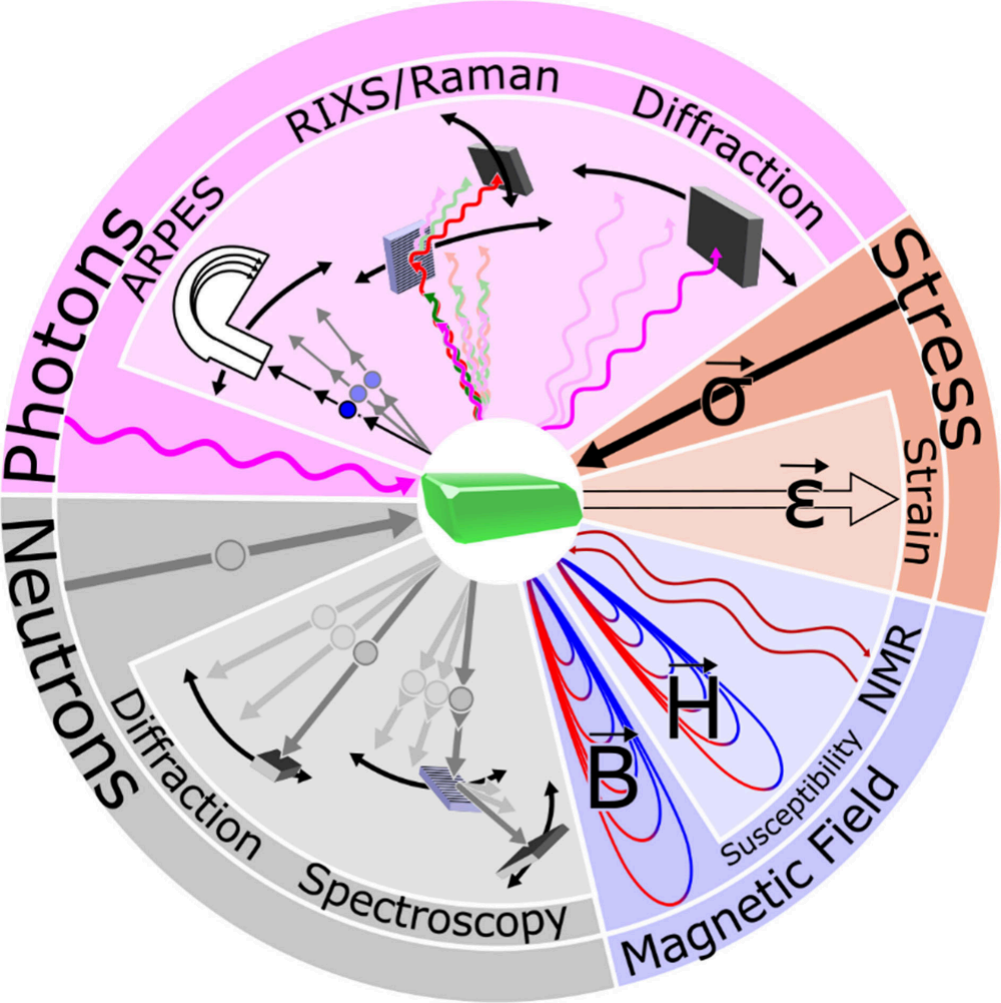
Angle-resolved methods for determining anisotropic properties of single-crystal specimens, grouped by stimulus type where sinusoidal lines represent photons, B⃗ is magnetic flux density, H⃗ is magnetic field strength, *ε⃗* is applied stress, and σ⃗ is strain.

Diffraction occurs by the same microscopic mechanism in single crystals as in polycrystalline specimens. In the latter case, however, the scattering vector *q* is simultaneously applied along a randomized population of crystallite orientations.^72^ This spherical ensemble averaging allows measurement of scattered intensity as a function only of the scattering vector magnitude, *I*(*q*). Single-crystal diffraction measures intensity as a function of each component of the scattering vector, *I*(*q*_*x*_ *,q*_*y*_ *,q*_*z*_), tripling the dimensionality of the data collected for each reflection and enabling resolution of reflections that would overlap in a powder diffraction pattern. *Ab initio* indexing and structure solution of single-crystal data has been a well-established practice for nearly 100 years,^73^ but has become increasingly tractable for powder diffraction patterns with monoclinic or lower symmetry by improvements in computing power.^74^

Under most circumstances, nearly all scattering intensity is elastic and involves an exchange of momentum, but not energy, between the specimen and the radiation field. Inelastic scattering techniques such as Raman spectroscopy, resonant inelastic X-ray scattering (RIXS), and inelastic neutron scattering involve energy-analyzing the scattered radiation to detect energy loss events such as electronic transitions, phonons, magnons, or other quasi-particle excitations. Dispersion curves can be constructed by plotting the differences in energy vs. momentum, *E*(*Q*), between incident and scattered radiation. However, the momentum and energy of the incoming radiation must exceed those of the excitation they are to generate. Optical Raman spectroscopy thus measures only very low-momentum quasi-particles such as optical phonons near the Brillouin zone center, although the use of polarized laser excitation enables selective enhancement of phonons oriented in different directions as per selection rules that enabling interrogation of anisotropic bonding in single crystals. RIXS employs photons with sufficient momentum to excite massive quasi-particles throughout the Brillouin zone, compensating for low scattering cross sections by tuning excitation energy to a core-level electron transition resonance and exploiting the high intensity of synchrotron radiation. RIXS interrogates electronic excitations including magnons, d–d transitions, and valence band transitions as well as phonon dispersion close to the elastic scattering peak.^75,76^ Neutrons are massive enough to scatter strongly from low-energy excitations like phonons and magnons and are extremely useful for measuring their dispersion relations but lack the energy to promote electron transitions between energy levels and are not a general-purpose probe of electronic structure.

Angle-resolved photoemission spectroscopy (ARPES) exploits the photoelectric effect much like conventional laboratory X-ray photoemission spectroscopy.^77,78^ The binding energy of an electron’s initial state is essentially the difference between the kinetic energy of the emitted photoelectron and that of the incident X-rays, and the initial state wave-vector is determined by crystal momentum conservation from its takeoff angle from the crystal surface. ARPES allows direct observation of a material’s valence band structure in reciprocal space and is an invaluable tool for studying electron–electron interactions that drive electronic structure transitions in single crystals.

The ability to orient magnetic fields along known crystallographic axes enables angle-dependent magnetic susceptibility and nuclear magnetic resonance (NMR) measurements.^79^ By passing an oriented single crystal through the bore of a powerful magnet and measuring the difference induced in the B and H fields, the magnetic susceptibility of a single crystal can be measured in multiple directions. The strengths and directions of multiple exchange couplings between nearby unpaired electrons, determined by fitting models to temperature-dependent susceptibility data, afford invaluable insight into electron interaction and localization behavior through phase transitions. Similarly, angle-dependent changes in Knight shifts in NMR spectra can help identify the spin orientation and distribution of paramagnetic electrons on lattice sites.^80^

The elasticity of materials can be measured by the application of directional stress and measurement of the resulting strain. The compressive, shear and torsional stiffness of anisotropic crystals varies with the direction of the applied stress but this anisotropy cannot be measured directly in polycrystalline samples. Single crystals, in principle, allow measurement of all 36 terms in the elasticity tensor, as well as anisotropic preferences for elastic misfit, direction of propagation of dislocations, and crack formation and propagation. This subject is treated in detail in Section 7.

## 2 Synthetic Routes and Strategies to Single Crystals

High-resolution structure elucidation, interrogating single crystals without ensemble or orientational averaging to build robust structure–function correlations, and functional applications uninterrupted by interfaces and grain boundaries requires access to high-quality single crystals. Challenges with growing single crystals represent a substantial bottleneck in the design and discovery of new materials. Despite being so pivotal, there is a drastic disparity between the constant search for *de novo* compounds (emphasized largely by the rapid growth and investments in X-ray, electron-beam and neutron facilities around the world that are used to characterize these compounds)^81^ and the continual loss of the art of single crystal growth. In the U.S. alone, there has been a marked decrease in the expertise of single crystal growth in industrial, academic, and national laboratory settings.^82^ Concordant with the focus of this review, this section will begin with a discussion of established vanadium oxide phase diagrams and trace the difficulties of obtaining vanadium oxide single crystals resulting from the closely segmented “rugged” free energy landscapes sketched in Figure 3 that require careful control of reaction trajectories to arrive at targeted compounds, and will conclude with a discussion of the different techniques that have been utilized to obtain vanadium oxide single crystals.

### 2.1 The V–O Phase Diagram

As noted above, vanadium adopts a broad range of oxidation states with formally divalent to pentavalent species commonly observed, which yields stoichiometric oxides VO, V_2_O_3_, VO_2_, and V_2_O_5_. As discussed above in Figure 2, there are also many phases that exist between VO_2_ and V_2_O_3_, which include the Magnéli (V_*n*_O_2*n*–1_; 3 ≤ *n* ≤ 9) and Wadsley–Roth (V_*n*_O_2*n*+1_; *n* ≥ 3) phases as well as substoichiometric mixed-valence phases V_12_O_26_, V_14_O_6_, and VO_0.532_ with structures correlated to parent compounds as described in Section 1.2. It comes to no surprise then, that the phase equilibria in the vanadium–oxygen phase space are complex. The isobaric vanadium–oxygen phase diagram, provided in Figure 9, exemplifies this complexity. Most vanadium oxides are incongruently melting, meaning many phase changes must occur from the liquidus to stabilize the final phase of interest. As a result, stabilizing these incongruently melting phases is inherently challenging because of the thermodynamic stability of a multitude of competing impurity phases. This challenge is further exacerbated by the fact that many of these phases, such as V_3_O_7_ and V_7_O_13_, can only be stabilized within rather narrow stoichiometric and temperature windows. One challenge that is largely overlooked by thermodynamic phase diagrams like the one provided in Figure 9, is that it does not include information on the variety of polymorphs that exist for many of the compositions (Figure 1) that can be accessed through kinetic constraints or under conditions of constrained equilibrium. Isolating each phase of interest thus requires precise control over the stoichiometry, temperature, partial pressure of oxygen, pH, and reaction environment (which, in some cases, can serve as a source of oxygen or can alter barriers to nucleation via epitaxial relationships),^83^ among other variables. Navigating the complexity of the phase diagram (Figure 9) and rugged free energy landscapes (Figure 3) requires use of a diverse range of crystal growth methods.

**Figure 9:**
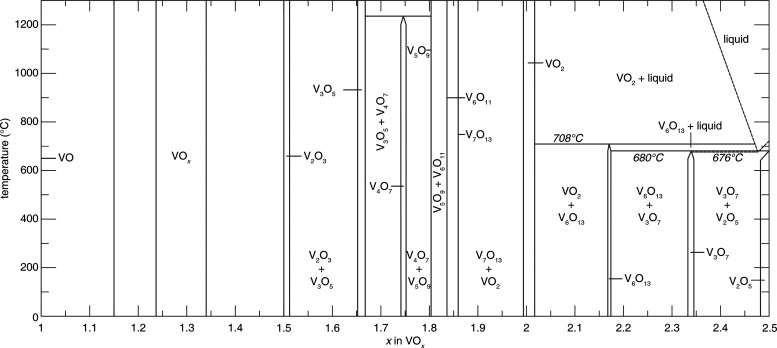
Experimental V–O binary phase diagram as a function of temperature. Reproduced with permission from reference 56. Copyright 2003 American Physics Society.

### 2.2 Single Crystal Growth Techniques

The growth of single crystals typically involves a change of state from a liquid (or supersaturated solution of monomers) to a solid or a gas to a solid. When in a fluid state, atoms and molecules are arranged randomly with no long-range order and considerable positional and configurational entropy. Single crystal growth requires the monomeric species to order in a periodic fashion. If crystal growth conditions are too rapid, the obtained crystal will contain disordered regions with defects and dislocations, or many smaller, single crystalline domains with varying orientations.^85^ Therefore, single crystal growth requires effective separation and control of nucleation and growth processes.

The choice of single crystal growth technique depends on the desired phase (for instance, whether incongruently melting or not, oxidation state of constituent elements) and the size of the crystal desired for the final application or characterization technique. These methods can be roughly divided into the following categories: solid-state, melt growth (Section 2.2.1), solution growth (Section 2.2.3), and vapor phase growth (Section 2.2.3).^85^ The variety of techniques under each of these single crystal growth types are shown in Figure 10, but for brevity we will only discuss the most common techniques that have been utilized to obtain vanadate single crystals. Melt growth techniques to obtain vanadates (Figure 11a) typically involve the Bridgman–Stockbarger (Figure 11b), Czochralski (Figure 11c), and floating-zone (Figure 11d) methods but also include lesser studied techniques such as Verneuil (flame-fusion methods) and skull melting. Solution methods such as hydrothermal (Figure 11e) and flux growth (Figure 11f) methods and vapor-phase methods like chemical vapor deposition (Figure 11g) have also shown promise in obtaining vanadate single crystals.

**Figure 10:**
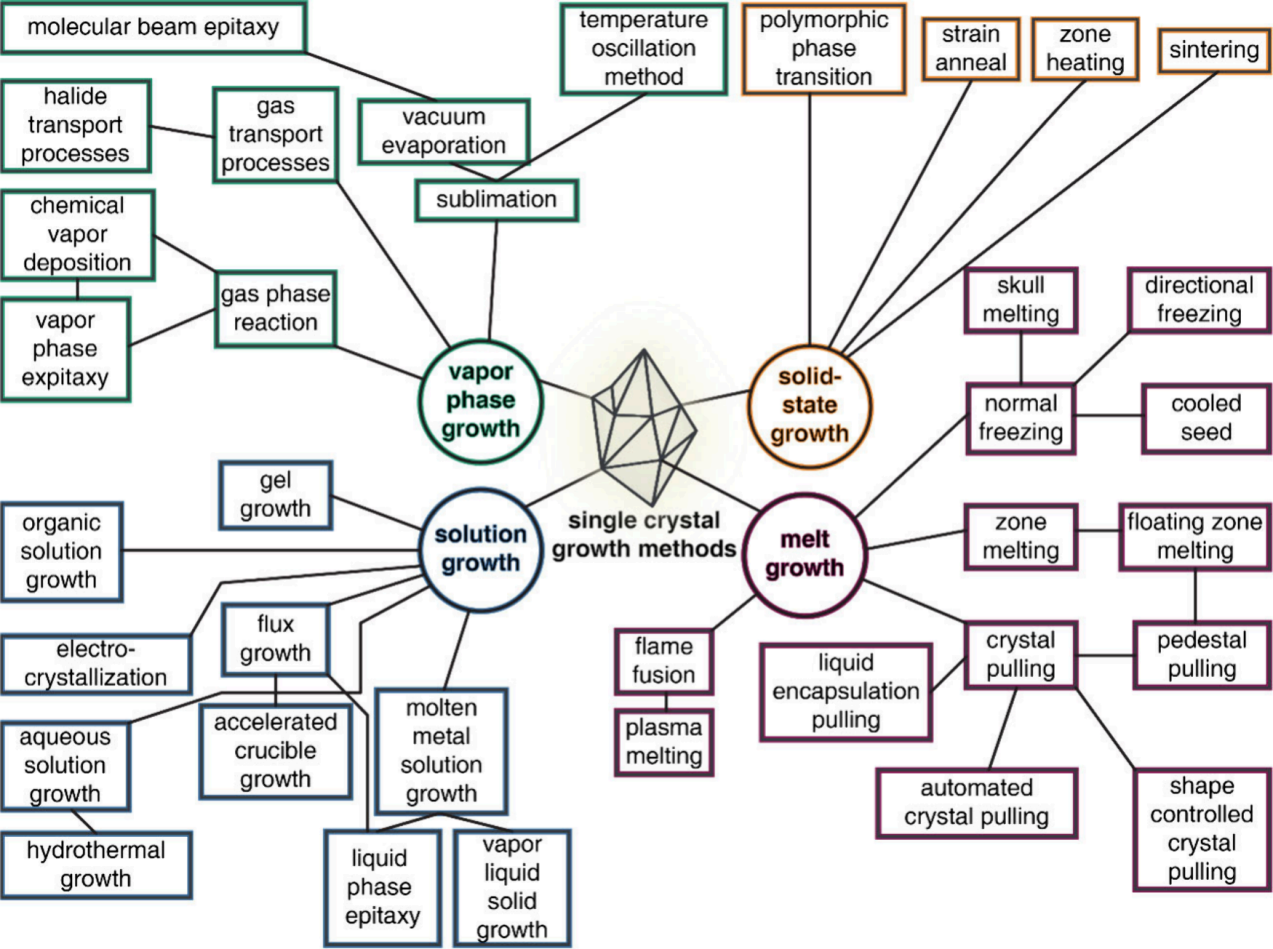
An overview on the various methods developed to obtain single crystals categorized by growth type: vapor phase growth (green), solution growth blue), solid-state growth (orange), and melt growth (purple). Reproduced with permission from reference 84. Copyright 1980 Elsevier.

**Figure 11:**
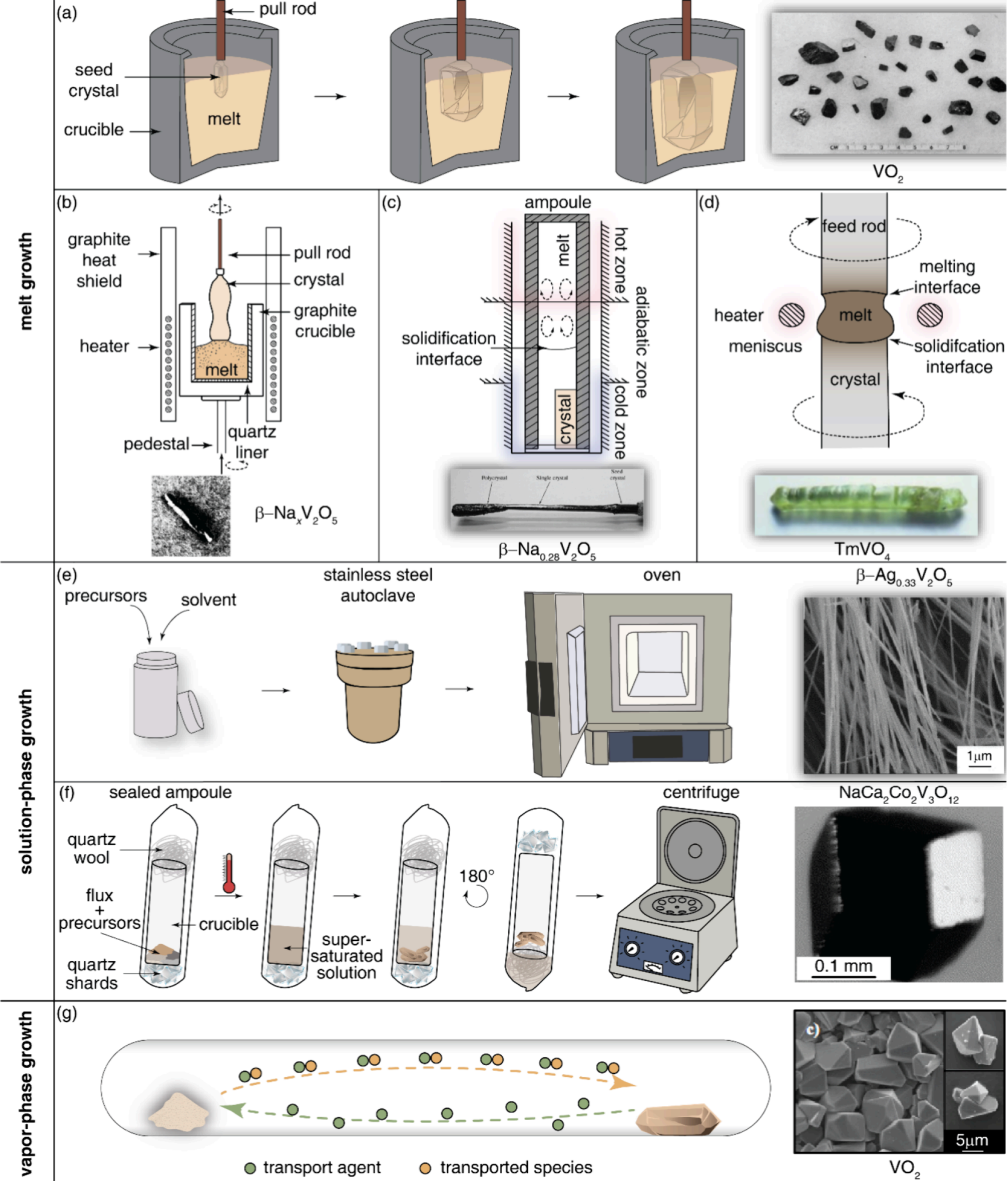
An overview of the most common techniques to obtain vanadate single crystals including (a) melt growth, (b) Bridgman–Stockbarger growth, (c) Czochralski growth, (d) floating zone growth, (e) hydrothermal growth, (f) flux growth, and (g) chemical vapor deposition. The image of the crystal in part (a) was reproduced with permission from reference 86. Copyright 1969 Elsevier. The image of the crystal in part (b) was reproduced with permission from reference 87. Copyright 1979 Elsevier. The image of the crystal in part (c) was reproduced with permission from reference 88. Copyright 2013 Springer Nature. The image of the crystal in part (d) was reproduced with permission from reference 89. Copyright 2006 Elsevier. The image of the crystal in part (e) was reproduced with permission from reference 90. Copyright 2006 Elsevier. The image of the crystal in part (f) was reproduced with permission from reference 91. Copyright 2006 Elsevier. The image of the crystal in part (g) was reproduced with permission from reference 92. Copyright 2007 Wiley.

#### 2.2.1 Melt Growth Techniques

The most common method for growing single crystals is from a melt. This involves precise conditions such that the atoms within the melt “freeze out” to form a nucleus.^93^ A nucleus can be a “seed crystal”, a single crystal that is typically too small for experimental characterization techniques, to induce heterogeneous growth of that particular lattice, or nucleation may occur spontaneously and homogeneously in a supercooled melt within the mold as a critical size is achieved. When seed crystals are utilized, a seed is placed at the end of a feed rod (Figure 11a) and a portion of the seed is melted. Then, either the furnace is slowly withdrawn from the seed or the mold is withdrawn from the furnace where the solid–liquid interface travels until the single crystal solidifies.^93^

The greatest challenge of obtaining single crystals from a melt, however, is that many desired phases, including most vanadates, do not congruently melt (Figure 2) and therefore crystals of varying composition are obtained from a single experiment. Vanadates, however, can exhibit a wide range of coloration (Figure 12) depending on the vanadium oxidation state and the presence of substitutional and interstitial dopants, which holds promise for application of machine learning methods for crystal sorting.^94^

**Figure 12:**
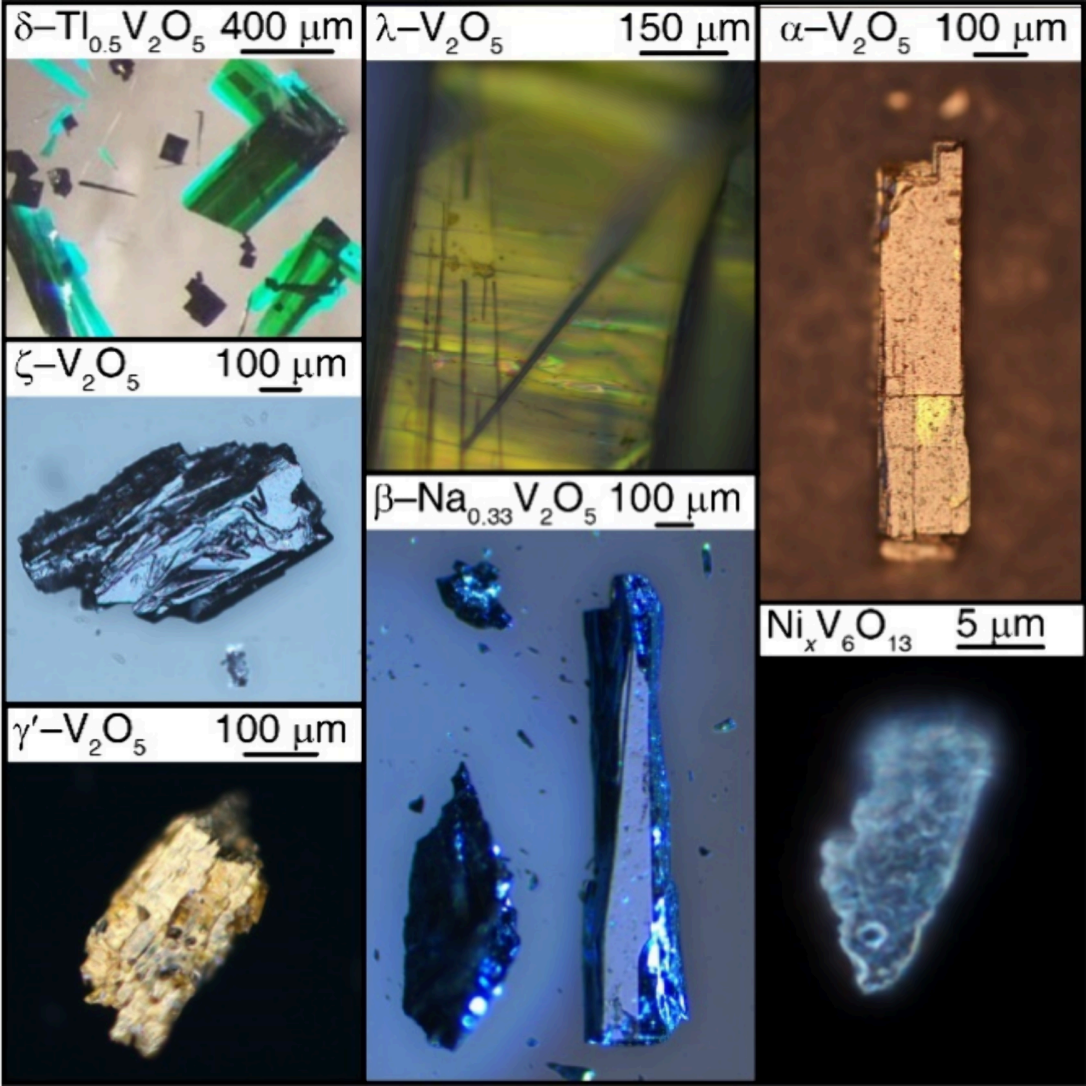
Vanadate single crystals grown by the authors through melt growth and hydrothermal synthesis. The image of δ-Tl_0.5_V_2_O_5_ was reproduced from reference 46 under a CC BY License. Copyright 2023 Agbeworvi et al.

While it is difficult to obtain phases that are incongruently melting directly from a melt, specific knowledge of the phase diagram, including phase transition temperatures, can be used to obtain single crystals of a phase with a stoichiometry different from the starting melt. The most popular example of this is the melt growth of VO_2_ single crystals from V_2_O_5_.^86,95^ For example, V_2_O_5_ can be placed in a Pt crucible and sintered at 1250 °C for 12 h under ambient conditions. Raising the temperature to 1350 °C and introducing N_2_ gas produces a neutral environment to continuously remove oxygen from the melt and establish an equilibrium partial pressure of oxygen within the atmosphere.^86^ The removal of oxygen causes the V^5+^ to reduce to V^4+^, effectively shifting the composition of the melt to VO_2_ and initiates precipitation of the desired phase. The melt can then be cooled to room temperature over a 24 h period to yield a large agglomeration of VO_2_ single crystals that could be easily separated (Figure 11a).^86^ Although this is not a typical melt growth that proceeds by cooling from a liquid, the gradual decomposition of the liquid V_2_O_5_ phase allows for the formation of macroscopic VO_2_ single crystals.

#### 2.2.2 Bridgman–Stockbarger Growth

The Bridgman–Stockbarger method is a widely used technique for the growth of single crystals, originating from the independent work of Percy Bridgman in 1925 and Donald Stockbarger in 1936.^96,97^ While subtly different, both methods involve utilizing a sealed starting material, usually a homogeneous precursor mixture or polycrystalline powder of the desired phase, which is placed within an ampule, often made of fused amorphous silica or another high-temperature-resistant, chemically inert material. A translation mechanism slowly moves the ampule from the hot zone to the cold zone, allowing the material to solidify in a controlled manner.^98^ This method relies on several fundamental transport processes, including (*i*) heat transfer, which requires a stable temperature gradient; (*ii*) mass transfer, as the molten material solidifies at the liquid–solid interface; (*iii*) diffusion, ensuring compositional homogeneity in the liquid phase; and (*iv*) convection, which can influence crystal quality because of fluid flows established within the melt.^99^ While both approaches rely on a temperature gradient and a translating crucible, their implementations differ subtly. Bridgman’s original method leverages a natural thermal gradient formed at the furnace exit where the molten material gradually cools as the ampule is withdrawn. Stockbarger instead introduced an insulating baffle separating two independently controlled furnace zones—one heated above the material’s melting point and the other cooled below its solidification temperature. This design creates a sharp, well-defined thermal gradient at the liquid–solid interface, enabling control over crystallization dynamics.

The Bridgman–Stockbarger method has been used to obtain single crystals of a variety of materials ranging from oxides,^96^ halides,^100^ semiconductors,^101^ and intermetallic alloys.^102^ Despite its versatility, the Bridgman–Stockbarger method is not well-suited for the growth of vanadium oxide single crystals. One of the primary challenges is the high volatility of V_2_O_5_, a common precursor during synthesis, at elevated temperatures. This high volatility leads to significant evaporation during the melting process, which makes it difficult to maintain a stable melt composition. Additionally, V_2_O_5_ is prone to thermal decomposition at high temperatures, resulting in the formation of lower vanadium oxides (e.g., VO_2_, V_2_O_3_) or even metallic vanadium, which disrupts the crystal growth process. This decomposition is inherently problematic due to the vanadium–oxygen system’s complex phase diagram (Figure 9), which features multiple nonstoichiometric oxides (e.g., VO_2_, V_6_O_13_, V_3_O_5_) and polymorphs with overlapping stability regions under varying oxygen partial pressures (*p*O_2_). In sealed ampule methods like Bridgman–Stockbarger, oxygen loss from V_2_O_5_ decomposition cannot be dynamically compensated, leading to a progressive reduction of the melt. This creates a thermodynamically unstable environment where competing phases nucleate simultaneously, resulting in polycrystalline aggregates rather than single-crystal growth of a target oxide.^103^ The chemical reactivity of V_2_O_5_ further complicates matters, as it can interact with the ampule, introducing impurities and compromising the purity of the resulting crystal. Finally, the method’s reliance on precise control of thermal gradients is particularly challenging for V_2_O_5_ because of its complex phase behavior and thermal properties, which makes it challenging to achieve high-quality single crystals using this technique.^99^

The difficulties in obtaining vanadium oxide single crystals through the Bridgman–Stockbarger method are evident as there are few reports of this method being successfully applied in the literature. Although incongruently melting, one example of a successful Bridgman–Stockbarger growth of vanadium oxide single crystals is of V_2_O_5_ itself, which was obtained by melting the V_2_O_5_ and utilizing a vertical displacement speed of 6 mm/h with a temperature gradient of ≈30 K.^104^ Interestingly, Bridgman–Stockbarger has also enabled the successful growth of β-Na_0.33_V_2_O_5_ (Figure 11b) through precise control of the sodium content within a melt made from NaVO_3_, VO_2_, and V_2_O_5_. Here, the presence of Na ions within the melt creates a critical thermodynamic sink toward Na-ion insertion within a polymorph of α-V_2_O_5_, ζ-V_2_O_5_ that stabilizes the β-Na_0.33_V_2_O_5_ phase during crystallization. While producing smaller crystals than Czochralski growth (discussed below), this thermodynamic advantage uniquely enables the growth of β-Na_0.33_V_2_O_5_.^87^

#### 2.2.3 Czochralski Growth

The Czochralski crystal growth enables the production of high-quality single crystals essential for electronic applications. The Czochralski method is a crucible-based crystal growth technique that relies on the controlled solidification of a melt. The process involves pulling a seed crystal from a melt pool within a crucible, with precise control of the temperature gradient, crystal/crucible rotation speed, and cooling rates to ensure optimal growth conditions. Rotation of the seed ensures uniform growth, whereas the pulling rate determines the crystal’s diameter and growth rate.^105–107^ Over time, advancements in crucible size and process parameters have necessitated a deeper understanding of melt convection dynamics, which critically influence crystal quality. Melt convection in Czochralski systems arises from three primary mechanisms: buoyancy-driven, forced, and thermocapillary (Marangoni) flows. Buoyancy-driven convection, governed by temperature and density gradients, dominates in large crucibles, forming toroidal flow patterns that modulate heat and species transport.^108^ Forced convection, induced by crystal and crucible rotation, alters flow symmetry and impurity distribution. High crystal rotation rates suppress turbulent buoyant flows, whereas crucible counter-rotation mitigates impurity incorporation.^109^ Marangoni convection, driven by surface tension gradients at the melt-gas interface, enhances radial flow toward the crystal, aiding oxygen transport control.^110^ Surface tension and convection dynamics, particularly thermocapillary forces from temperature-induced surface tension gradients, critically shape crystal growth by driving inward melt flows, stabilizing the melt/crystal interface, and promoting uniform growth through impurity redistribution;^111^ experimental evidence confirms that free melt surfaces enhance flow efficiency, whereas restrictive conditions can degrade interface quality and impurity control, ultimately underlining the complex interplay of parameters in producing high-quality crystals.

Several critical parameters must be controlled during a Czochralski growth to achieve high-quality crystals. Pulling rate directly influences the crystal’s diameter and growth rate. Faster pulling rates can produce larger crystal diameters, but they simultaneously risk introducing defects if not carefully managed. The crucible temperature is also crucial for maintaining the melt in a liquid state and preventing supercooling or premature solidification. As both pulling rate and crucible temperature play critical roles in the success of a Czochralski growth, these parameters are often empirically modeled to reveal optimal synthetic conditions.^107^ Another key parameter is the crystal rotation speed as it ensures radial symmetry and influences the melt/crystal interface, impurity distribution, and the generation of crystal stresses.^105,106^ The melt level and crucible geometry significantly impact heat and mass transport, with lower melt levels potentially enabling faster heat transfer and more stable growth processes, though they may simultaneously constrain the maximum crystal size that can be obtained.^106,107^

There are several significant challenges and limitations that must be carefully navigated to produce high-quality crystals using the Czochralski method. These limitations have inspired researchers to develop models that can inform experimental parameters to optimize crystal growth. Defects and dislocations pose a critical concern. Tsai and Subramanyam explored how growth parameters like orientation and ambient temperature influence dislocation density and proposed strategic optimization approaches to mitigate these structural imperfections.^112^ The melt/crystal interface also presents another crucial challenge, as highlighted by Grajower and co-workers, who examined the delicate balance of heat flow and derived critical expressions defining stable growth conditions and the smallest diameter for necking down.^107^ Additionally, one common pitfall in Czochralski growth is the volatilization of precursors. Wang and co-workers demonstrated the severity of this issue through their research on La_3_Ga_5.5_Ta_0.5_O_14_ (langatate) crystal growth, where they found that volatilization processes like Ga_2_O_3_ escape can introduce compositional changes and potentially compromise crystal quality.^113^ These interconnected challenges underscore the complexity of the Czochralski method, which requires precise control and deep understanding of thermal, structural, and compositional dynamics to successfully produce high-quality crystals.

The successful growth of single-crystalline β-Na_0.28_V_2_O_5_ by the Czochralski method (Figure 13) has been demonstrated by independent research efforts, each optimizing distinct aspects of the process. The melting–crystallization process is a reversible redox reaction given by eq 1.

1 $${\left[Na_{0.28}V_{12}O_{30\pm y}\right]}_{\mathrm{sol}}+\left(0.5x\pm \left(\frac{y}{2}\right)\right)O_{2}\left(g\right)↔{\left[Na_{0.28}V_{12}O_{30\pm 0.5x}\right]}_{\mathrm{liq}}$$

**Figure 13:**
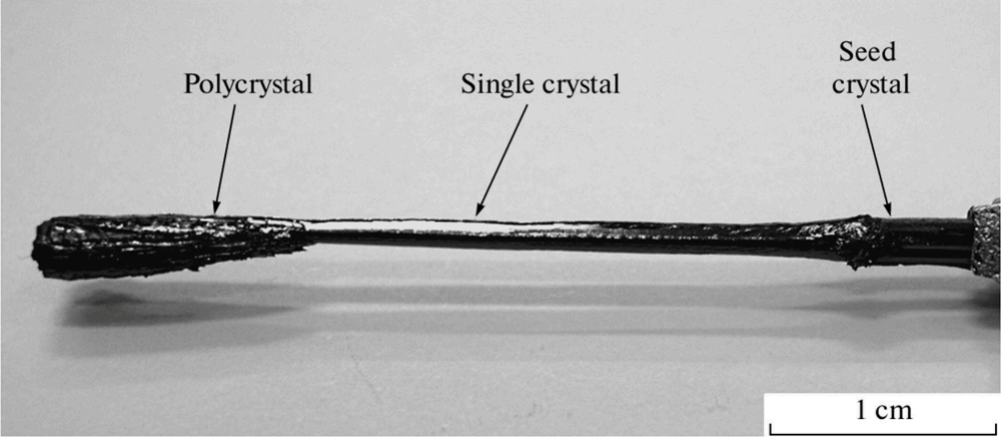
Crystal of β-Na_0.28_V_2_O_5_ grown from melt by Czochralski method. This image was adapted with permission from reference 88. Copyright 2013 Springer Nature.

One study achieved growth under ambient atmospheric conditions by employing a tailored temperature gradient (120 °C/cm) and oxygen exposure to stabilize the vanadium oxide melt, effectively addressing volatility and oxygen loss challenges. Another method focused on procedural refinements, utilizing taper growth techniques, small-diameter seed crystals, and controlled parameters (pulling speed: 1–2 mm/h; rotation: 7–10 rpm) to produce prismatic crystals (1–2 mm cross-section, 20–40 mm length) with a pronounced [100] elongation, underscoring the structural precision attainable through Czochralski. Together, these works illustrate the adaptability of the Czochralski method in tailoring β-Na_0.28_V_2_O_5_ crystals for both functional applications and controlled morphological outcomes.^88,114^

The Czochralski method has also been utilized to obtain meta- and orthovanadate single crystals. For example, LiVO_3_, NaVO_3_, and KVO_3_ were obtained from stoichiometric melts. The precursors were heated gradually over the course of 7 h to their melting points (LiVO_3_: 545 °C; NaVO_3_: 510 °C; KVO_3_: 520 °C) and rapidly heated to 1000 °C for homogenization. Growth was conducted in platinum crucibles under ambient atmosphere using a pull rate of 0.2 mm/h.^115^ BiVO_4_ single crystals that crystallized in *I*2/*b* were also grown by combining Bi_2_O_3_ and V_2_O_5_ in stoichiometric ratios, melting at 1214 K in a platinum crucible, and pulling at 1 mm/h with 8 rpm seed rotation. Slow cooling (12 h) yielded transparent brownish-yellow boules (∼55 mm diameter, 30 mm length). Twin domains formed from ferroelastic transitions during cooling, but twin-free <5 mm cubes were cleaved along [001] planes.^116^

#### 2.2.4 Floating Zone

Unlike conventional crystal growth methods requiring a crucible, the floating zone technique avoids potential contamination from a reaction container by suspending a molten zone between a polycrystalline feed rod and a seed crystal via optical or radio frequency heating.^117,118^ High-quality feed rods are typically synthesized through solid-state reactions of precursor powders, followed by isostatic pressing and sintering. The rod’s density, homogeneity, and chemical composition directly affect crystal quality; voids or compositional fluctuations can propagate as cracks or inclusions in the final crystal.^118,119^ This crucible-free approach ensures stoichiometric control and minimizes defects, making it essential for synthesizing pure, large-volume crystals needed to study material properties such as metal-to-insulator transitions or magnetic ordering such as through neutron scattering or ARPES.

In floating zone synthesis, the feed and seed rods fulfill distinct roles. The feed rod, positioned above the seed rod, acts as the material source for crystal growth. As the molten zone advances, the feed rod is gradually consumed. The purity and quality of the feed rod directly influences the ultimate purity of the obtained crystal. The seed rod initiates crystallization by dipping into the molten zone and establishing the crystal’s orientation and quality. Proper alignment between the rods is vital to ensure uniform growth and minimize defects.^118,119^ Their interaction stabilizes the molten zone, which depends on temperature gradients, rotation rates, and ambient atmosphere. For example, Behr et al. demonstrated that Ar/O_2_ gas mixtures enhance stability by aiding oxygen transport and suppressing bubble formation.^119,120^

Congruent melting—where a material solidifies without compositional changes—is critical in floating zone growth to preserve stoichiometry. For vanadium oxides, which exhibit complex phase behavior, congruent melting avoids secondary phases that degrade crystal quality.^118,121^ Materials like V_2_O_3_, which is one of the few vanadium oxides that melt congruently (i.e., the solid and liquid phases share the same composition), enable steady crystal growth without compositional segregation. In cases of incongruent melting (e.g., certain vanadium-based oxides like VO_2_, V_2_O_5_), the oxide can decompose and vaporize at different temperatures, which can lead to compositional variations, nonuniform crystal structure, and potential loss of volatile components.^118,122^

Two important parameters when performing a floating zone synthesis are the rotation rate and reaction atmosphere. Rotation rates of the rods significantly influence molten zone dynamics where low rates risk subgrain boundary formation, whereas high rates may introduce bubbles. Similarly, precise oxygen partial pressures (*p*O_2_) during growth are crucial for stabilizing the oxidation states of vanadium cations. For example, LuVO_4_ [V^5+^] forms in oxygen-rich atmospheres, Lu_2_V_2_O_7_ [V^4+^] under slight reducing conditions, and LuVO_3_ [V^3+^] under reducing conditions. Compared to traditional Czochralski growth, the floating zone method’s steep temperature gradient enables faster growth and higher purity, whereas the shallower gradient in the Czochralski method provides better diameter control.^123^ However, precise *p*O_2_ control, slow growth rates, careful feed rod preparation, and controlled postgrowth annealing in floating zone syntheses are essential to maintain the desired oxidation state, minimize volatilization, and reduce thermal stresses, in order to ensure high-quality crystal growth.^123^

Carefully optimized floating zone syntheses have had tremendous success in obtaining large, high-quality single crystals. For example, single crystals of rare-earth orthovanadates *R*VO_4_, where *R* = Y, La, Ce, Pr, etc., were successfully grown (Figure 14). Feed rods, prepared from rare-earth oxides and V_2_O_5_, were calcined at 800 °C for 15 h, sintered at 1200–1400 °C, and grown at 2–10 mm/h with 30 rpm counter-rotation. The obtained crystals (7 × 7 mm^2^ cross-section, 20–50 mm length) exhibited body colors reflecting trivalent rare-earth ions, except colorless YVO_4_, LaVO_4_, and LuVO_4_. Challenges arose with CeVO_4_ and NdVO_4_ where low surface tension yielded opaque polycrystals. However, further optimization of the surface tension of the melt eventually enabled the authors to grow deep purple NdVO_4_ crystals.^89^ This study emphasizes the critical role of addressing surface tension in the crystal growth process to achieve high-quality crystals of *R*VO_4_ materials.^4^

**Figure 14:**
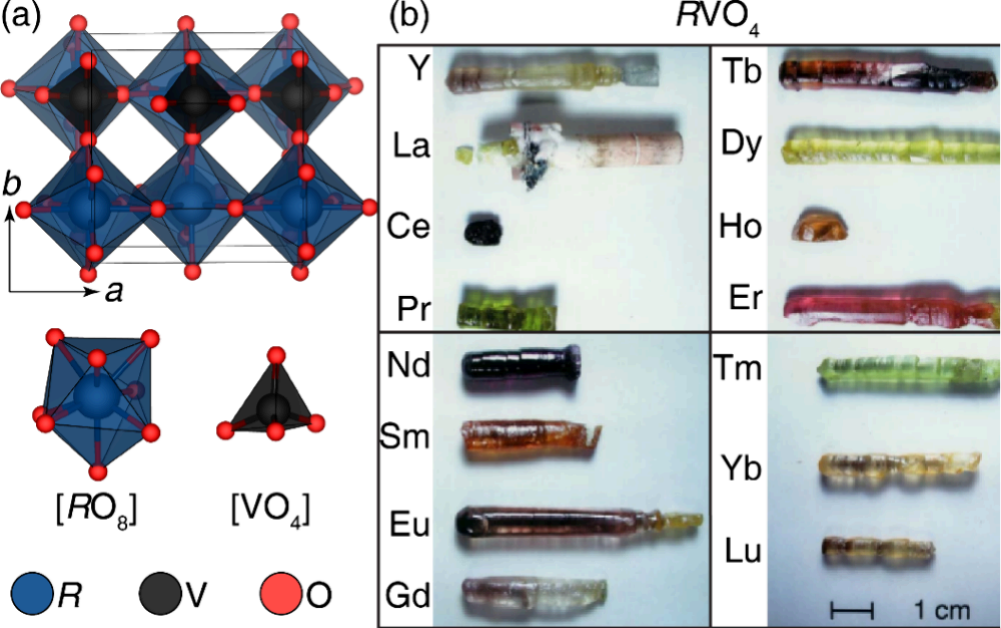
Crystal images of *R*VO_4_ grown crystals. Reproduced with permission from reference 89. Copyright 2006 Elsevier.

#### 2.2.5 Hydrothermal Synthesis and Growth

Hydrothermal synthesis refers to synthesis that is performed in an aqueous medium at temperatures and pressures greater than 100 °C and 1 bar, respectively.^124^ This synthetic technique involves combining precursors and an appropriate solvent in a poly­(tetrafluoroethylene) cup and placing the cup in a sealed reaction vessel, usually a bomb or autoclave, and heating (Figure 11e). In this reaction vessel, the solvent is brought to temperatures greater than their boiling point due to an increase in the autogenous pressure from heating.^125^ It is important to note that the reaction temperature does not have to be very high due to the high pressures achieved during the reaction. Therefore, most hydrothermal reactions are performed between 100 and 250 °C where the upper limit is usually governed by the thermal stability of poly­(tetrafluoroethylene) at elevated temperatures.^125^ Many parameters can affect the result of a hydrothermal synthesis (i.e., single vs polycrystalline, particle morphology, sample purity) especially the pH of the reaction medium (which governs the solution-phase speciation through the Pourbaix diagram^9^), the relative ratios of the precursors (i.e., concentrations of ions), potential reducing or oxidizing reagents (which further govern the redox potentials and modify solution-phase speciation based on the Pourbaix diagram), the type of ion present in solution (whether spectator, shape-directing, or templating ions), and the temperature of the reaction.^126^ Of these variables, pH is one of the most important for hydrothermal synthesis because it has a large influence over the species that are present within the aqueous media. As a result, hydrothermal reactions are usually typically designed based on consideration of a Pourbaix diagram.

Also known as a potential/pH diagram, Pourbaix diagrams plot the electrochemical stability of a metal at various oxidation states as a function of pH where solid lines represent regions where phases can coexist. As such, different species and specific oxidation states of elements can be targeted by simply shifting the pH of the initial reaction mixture through the addition of specific acids and bases like HClO_4_ and NaOH, respectively, where the addition of the Na^+^ and Cl^–^ ions do not participate in the resulting reaction and serve only to maintain charge neutrality. Similarly, the oxidation potential of the mixture can be fine-tuned through the addition of H_2_O_2_ or organic reducing/oxidizing reagents.^127^ Pourbaix diagrams are typically presented at room temperature, ambient atmospheric pressure, and a molar concentration of 1 μM and changing either of these parameters will change the appearance of the diagram.^127^ Similar to the vanadium–oxygen phase diagram given in Figure 9, a Pourbaix diagram does not yield any information on reaction-rate or kinetic effects but provides a means of modulation solution-phase speciation to converge on a desired synthetic outcome.

The Pourbaix diagram of vanadium oxide as a function of oxidation potential and ion concentration are provided in Figure 15a and Figure 15b, respectively. The Pourbaix diagrams reveal that the pervandyl cation, VO_2_^+^, with pentavalent V, is only stable at low pH (between 1–3) and a very narrow oxidation potential window between 1.0–1.4 V, but at a wide range of molar concentrations. As the pH increases, the most stable species are H_2_V_10_O_28_^4–^ (pH ≈ 2–3), HV_10_O_28_^5–^ (pH ≈ 3–5), V_10_O_28_^6–^ (pH ≈ 6–6.5), V_4_O_12_^4–^ (pH ≈ 6.5–9.5), V_2_O_7_^4–^ (pH ≈ 9.5–12.5) and finally VO_4_^3–^ (pH ≈ 12.5–14) where the oxidation potential window becomes incrementally larger with increasing pH. The regions of tetra-, tri-, and bivalent cations exist in a far narrower pH and oxidation potential range where higher pHs tend to stabilize the solid (shaded regions in Figure 15a).^127^

**Figure 15:**
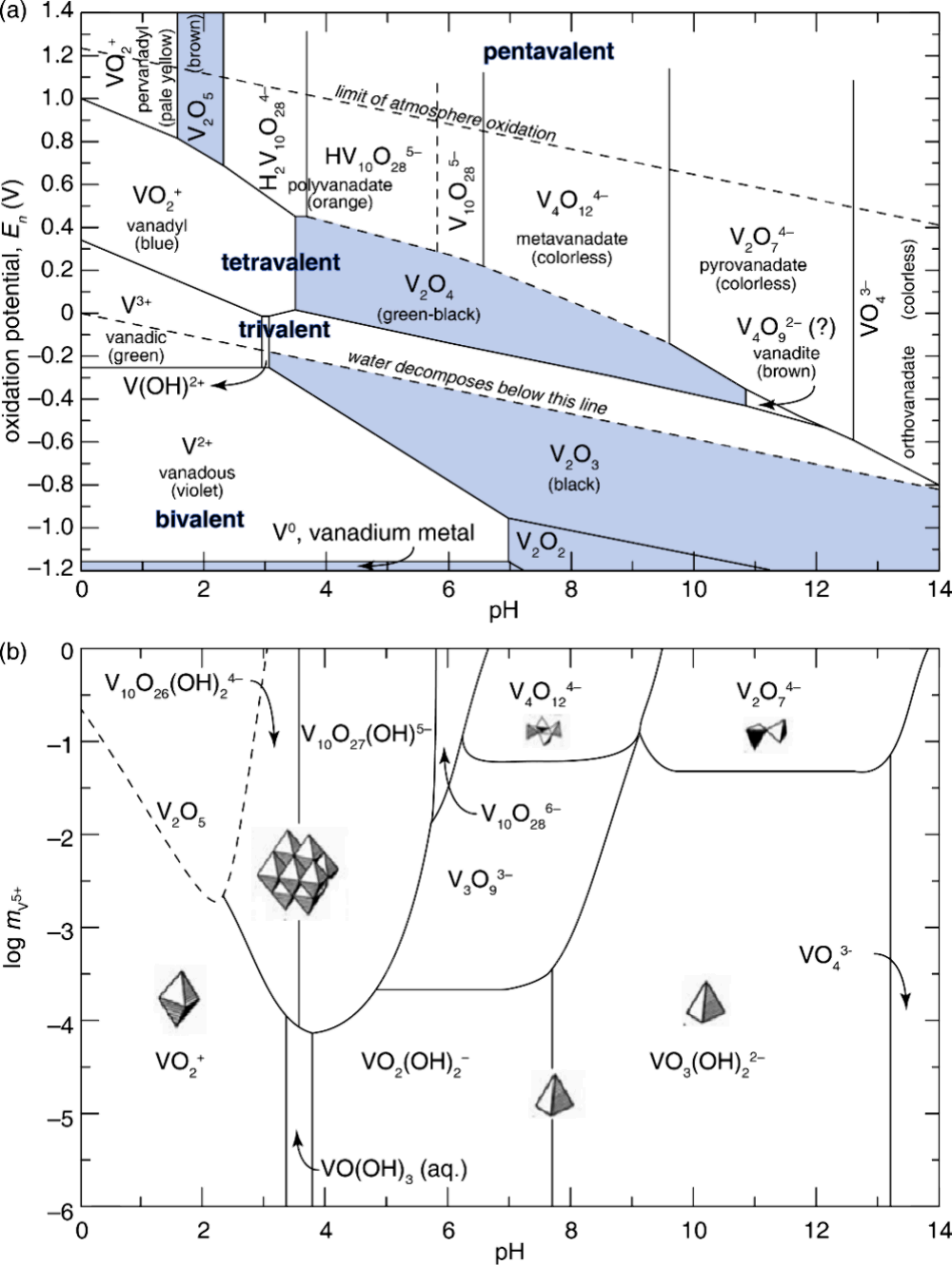
Vanadium Pourbaix diagram as a function of (a) oxidation potential and (b) ion concentration. (a) was adapted with permission from reference 127. Copyright 1958 Elsevier. Panel (b) was adapted from reference 128 under a CC BY license. Copyright Livage et al.

Pourbaix diagrams are clearly vital for ensuring that the desired ionic species are present within a hydro- (or solvo-) thermal reaction. The importance of carefully regulating the pH of a hydrothermal reaction has been demonstrated in many investigations where all reaction conditions are kept constant except for pH. For example, V_2_O_5_, tetramethylammonium (TMA) hydroxide, and LiOH were placed in a hydrothermal vessel in a molar ratio of 1:2:1 and reacted at 185 °C for 72 h where the pH of each vessel was tuned from 2 to 10 through the addition of acetic acid and showed 7 distinct phases that could be stabilized across the pH range (Figure 16a).^126^ These phases also adopted a variety of different particle morphologies ranging from single crystalline plates to fibers, and larger crystallites (Figure 16b–g).^126^ Many of the obtained phases are large enough to where a single crystal could be isolated from the agglomerates and used either as seed crystals for further Bridgman crystal growth discussed above or further average, local, or electronic structure experiments.

**Figure 16:**
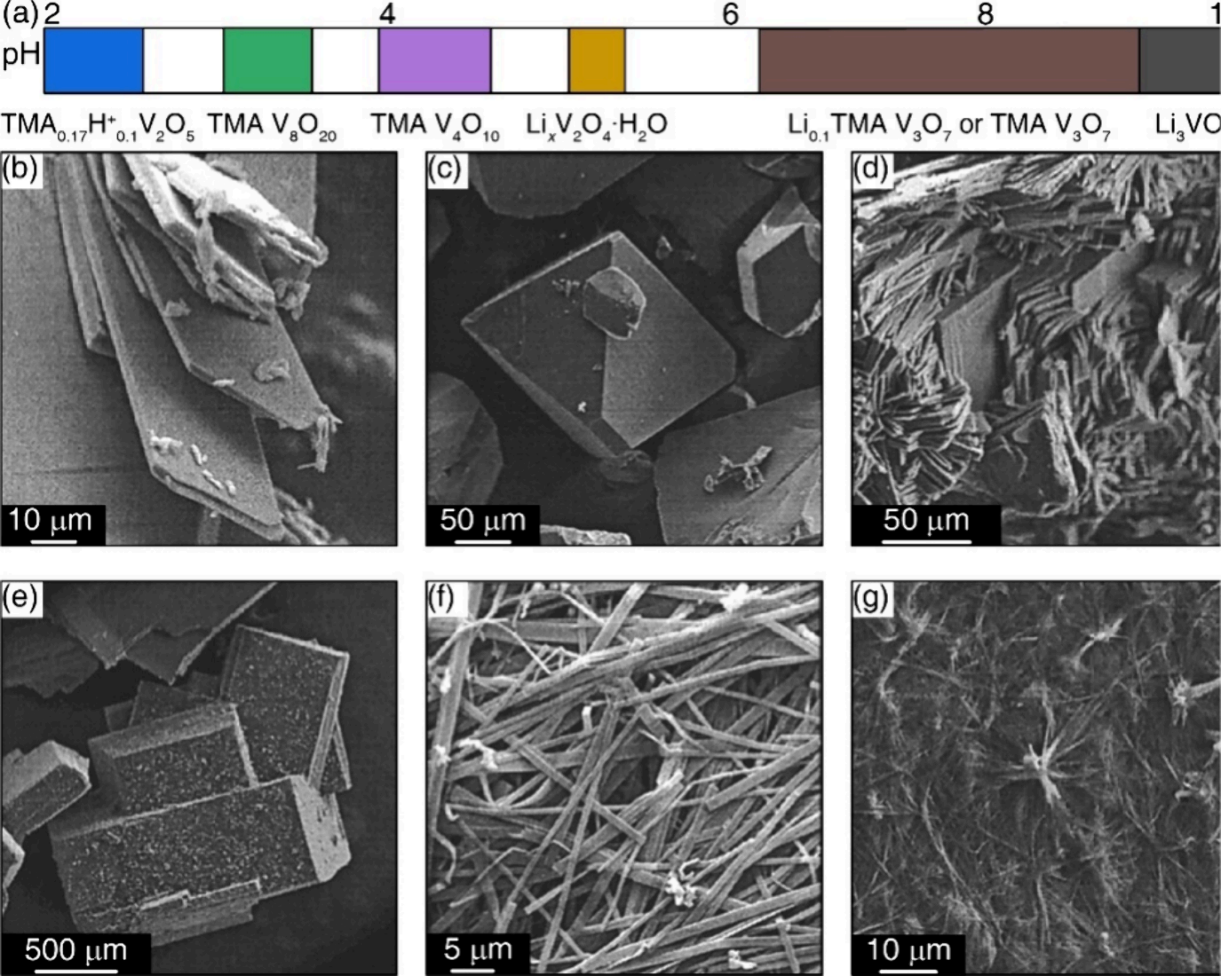
(a) Phases produced when reacting V_2_O_5_, tetramethylammonium hydroxide, and LiOH in a hydrothermal vessel and adjusting the pH to the indicated levels with acetic acid. Morphology of (b) TMAV_3_O_7_, (c) TMA_4_[V_22_O_54_(CH_3_COO)]·4H_2_O, (d) Li_*x*_V_2_O_4_·H_2_O, (e) TMAV_4_O_10_, (f) TMAV_8_O_10_, and (g) TMA_0.17_H_0.1_V_2_O_5_ at different pH levels. Tetramethylammonium is abbreviated here as TMA. This figure was reproduced with permission from reference 126. Copyright 1998 American Chemical Society.

The pH of a hydrothermal reaction can also be tuned to obtain different particle morphologies from a single phase. BiVO_4_, a popular photocatalyst, was synthesized from solutions from Bi­(NO_3_)_3_·5H_2_O and NH_4_VO_3_ mixed in a 1:1 molar ratio where the pH was adjusted to 1, 4, and 9 upon the addition of NH_3_.^129,130^ Each sample was transferred to a poly­(tetrafluoroethylene) cup and stainless-steel reactor and heated to 453 K in an oven for 48 h. BiVO_4_ can crystallize in three different polymorphs: tetragonal zircon (*I*4_1_/*amd*), monoclinic scheelite (*I*2/*b*), and tetragonal scheelite (*I*4_1_/*a*). For comparison, a set of standard samples were also prepared. This includes a sample where no pH adjustment was made (denoted as BiVO_4_ (u)), a sample that was prepared through a homogeneous precipitation (denoted as BiVO_4_ (h)), and an unreacted, amorphous sample of the dried precursors after the pH was adjusted to one (denoted as BiVO_4_ (a)). Despite a complicated phase space, monoclinic scheelite-type BiVO_4_ was the only phase observed in the powder X-ray diffractograms regardless of pH (Figure 17a). One discernible difference between the observed compounds was the full width at half-maximum of the diffraction reflections increased as a function of pH, indicating that the particle size of the obtained BiVO_4_ was becoming incrementally smaller under more basic conditions. Indeed, the crystal sizes, determined from the Scherrer formula, were 243 nm (pH = 1), 85 nm (pH = 4), and 49 nm (pH = 9).^129^ Field-emission scanning electron microscopy (FE-SEM) revealed significant differences in the particle morphology. The BiVO_4_ synthesized at a pH of 1 comprises large aggregates of particles of irregular morphologies (Figure 17b), whereas increasing the pH to 4 and 9 yielded rod-like grains (Figure 17c) and particles with longer rods, almost needle-like morphologies (Figure 17d-e), respectively. Ultimately, the different particle sizes and morphologies had a significant impact on the photocatalytic activity (Figure 17f) of the obtained BiVO_4_ where the initial rate of O_2_ evolution decreased from 92 μmol/h (pH = 1) to 63 μmol/h (pH = 4) and 19 μmol/h (pH = 9).^129^ This study underlines the importance of crystallinity on the photocatalytic activity of a material and highlights how a material’s properties can be tuned by modifying the particle morphology using hydrothermal synthesis.

**Figure 17:**
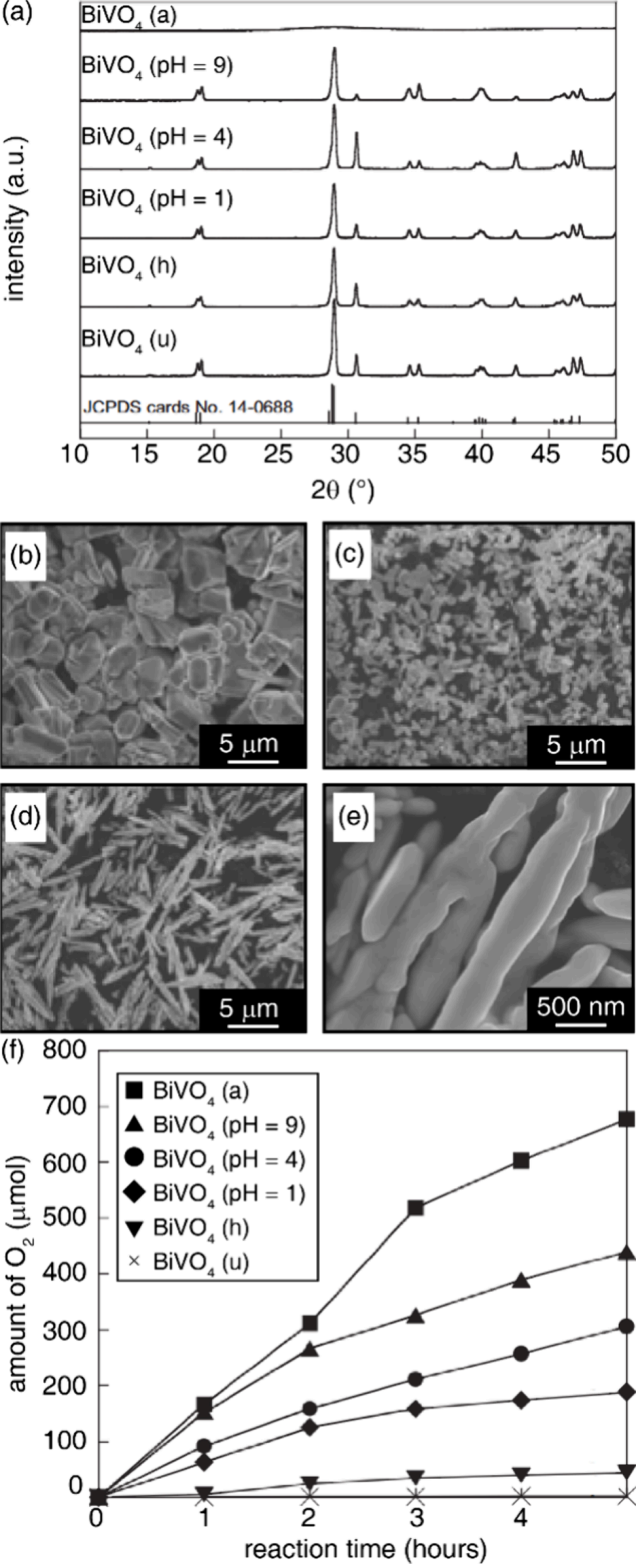
(a) Powder X-ray diffraction patterns of amorphous BiVO_4_ (BiVO_4_ (a)), BiVO_4_ that was synthesized through homogeneous precipitation (BiVO_4_ (h)), pH-dependent BiVO_4_, and an unreacted sample of BiVO_4_ (BiVO_4_ (u)). The morphology of BiVO_4_ when synthesized at a pH of (b) 1, (c) 4, and (d) 9. (e) A zoomed in image of (d) BiVO_4_ synthesized at a pH of 9. (f) Photocatalytic O_2_ evolution from a 150 mL 0.05 M AgNO_3_ solution under visible-light irradiation (λ ≥ 420 nm). This figure was reproduced with permission from reference 129. Copyright 2006 Wiley.

Finally, hydrothermal synthesis has the added advantage of yielding highly crystalline nanomaterials of varying particle morphologies that can be used to prepare vanadium oxide-based thin films. For example, different particle morphologies of VO_2_ (B) have been obtained using hydrothermal synthesis such as nanowires, nanobelts, nanorods, nanotubes, and 3D nanostructures. Nanowires of VO_2_ (B) with a diameter of 50–60 nm and a thickness of 6–10 nm can be obtained by reaction of V_2_O_5_ with water and ethylene glycol (Figure 18a).^131,132^ Figure 18c depicts free-standing nanosheets of VO_2_ (B), which was obtained by preparing a V_2_O_5_ suspension and dispersing in H_2_O_2_ and isopropanol.^133^ This suspension was then loaded into an autoclave and heated. Upon completion of the reaction, the suspension was washed in ethanol and vacuum filtered, which was peeled off to obtain the film. Optimizing the temperature and time of the reaction resulted in the free-standing film that stack evenly on the (001) plane. Nanorods of VO_2_ (B), like those shown in Figure 18d, can be obtained by dissolving V_2_O_5_ using a reducing agent such as lactose, maltose, glucose, pentaerythritol, ethanol, and glycerol in water and transferring the mixture to an autoclave and reacting at temperatures between 160 and 180 °C.^137^ It was found that when glycerol was used as the reducing agent, the crystallinity of the obtained VO_2_ (B) was higher and the obtained nanorods were more uniform with a diameter of ≈ 50 nm and a length of 0.5–2 μm. Finally, more unconventional particle morphologies of VO_2_ (B) have also been achieved. Flower-like micronanostructures of VO_2_ (B) were assembled from single crystalline nanosheets using polyvinylpyrrolidone as a capping reagent (Figure 18e).^135^ V­(IV)­Oacac_2_ and poly­(vinylpyrrolidone) were ultrasonicated, transferred to an autoclave, and heated at 200 °C for 24 h. Uniform, flower-like architectures, 1–1.5 μm in diameter, with “nanopetals” ≈ 20–30 nm thick have been obtained. Similarly, urchin-like VO_2_ (B) nanostructures composed of radially aligned nanobelts were synthesized upon reaction of V_2_O_5_ with peroxovanadic acid and oxalic acid under hydrothermal conditions (Figure 18f).^136^ The nanobelts were determined to be 20–30 nm thick and 50–150 nm wide and the diameter of the overall nanostructures were 2–6 μm. Through multiple time- and temperature-dependent studies, it was found that the VO_2_ (B) nanostructures form via a reduction-dehydration phase-transition and disassembly process following eqs 2–4.

2 $$V_{2}O_{5}\left(s\right)+4H_{2}O_{2}\left(l\right)\to 2{\left[\mathrm{VO}{\left(O_{2}\right)}_{2}\right]}^{-}\left(\mathrm{aq}.\right)+2H^{+}\left(\mathrm{aq}.\right)+3H_{2}O\left(l\right)$$

3 $${\left[\mathrm{VO}{\left(O_{2}\right)}_{2}\right]}^{-}\left(\mathrm{aq}.\right)+10H^{+}\left(\mathrm{aq}.\right)+C_{2}H_{2}O_{4}\left(l\right)+6H_{2}O\left(l\right)\to V_{10}O_{24}\cdot 12H_{2}O\left(s\right)+2{\mathrm{CO}}_{2}\left(g\right)+10O_{2}\left(g\right)$$

4 $$V_{10}O_{24}\cdot 12H_{2}O\left(s\right)+4C_{2}H_{2}O_{4}\left(l\right)\to 10{\mathrm{VO}}_{2}\left(B\right)\left(s\right)+8{\mathrm{CO}}_{2}\left(g\right)+16H_{2}O\left(l\right)$$

**Figure 18:**
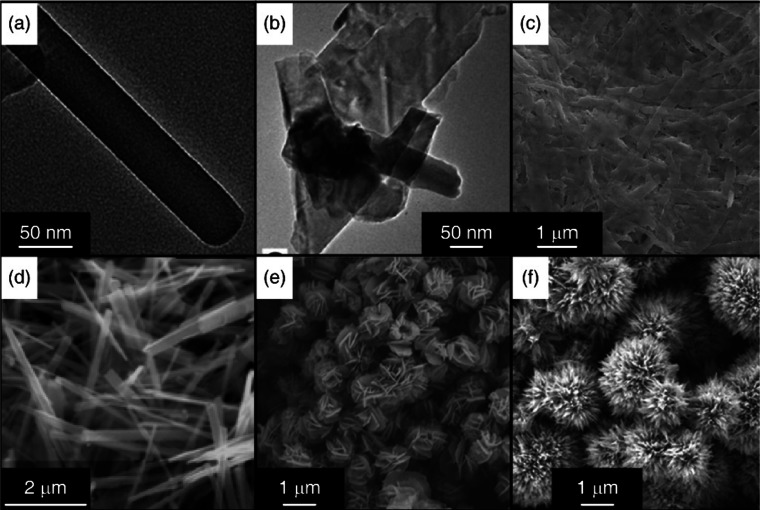
Hydrothermal synthesis of VO_2_ (B) with various morphologies including (a) nanowires, (b) nanosheets, (c) free-standing nanosheets, (d) nanorods, (e) flower-like micronano structures, and (f) urchin-like nanostructures. Panel (a) was reproduced with permission from reference 131. Copyright 2008 Elsevier. Panel (b) was reproduced with permission from reference 132. Copyright 2015 Elsevier. Panel (c) was reproduced with permission from reference 133. Copyright 2018 Elsevier. Panel (d) was reproduced with permission from reference 134. Copyright 2009 American Chemical Society. Panel (e) was reproduced with permission from reference 135. Copyright 2009 American Chemical Society. Panel (f) was reproduced with permission from reference 136. Copyright 2009 American Chemical Society.

First, V_2_O_5_ dissolves in hydrogen peroxide to form [VO­(O_2_)_2_]^−^ diperoxo anions, which become reduced through the addition of oxalic acid to from V_10_O_24_·12 H_2_O clusters that serve as the site for heterogeneous nucleation for urchin-like V_10_O_24_·12H_2_O nanostructures. Layered V_10_O_24_·12 H_2_O is then further reduced by the oxalic acid, which causes a reduction-dehydration topotactic transformation that yields the final urchin-like VO_2_ (B) nanostructures.

#### 2.2.6 Flux Growth

Flux growth describes a single crystal growth method in which the starting materials are dissolved in a flux (solvent) and the crystals are precipitated out of solution similar to recrystallization of molecular inorganic compounds. The flux lowers the melting point of the desired compound while also remaining agnostic of the crystallization event.^138,139^ The lowered melting point (and thus lower reaction temperatures) are vital for obtaining crystals of phases that melt incongruently as reaching the melting point of an incongruently melting phase will cause the phase to disproportionate.^140^ In typical flux growth experiments, the precursors dissolve in the flux and crystallization occurs once the solution becomes supersaturated. Supersaturation can be achieved by reducing the volume of the melt through evaporation or cooling the melt.^140^

A diverse range of fluxes have been used for the flux growth of single crystals.^139,141–143^ The type of flux largely depends on the desired phase. Indeed, the criteria for an “optimal” flux include: (i) the ability to dissolve most, if not all, of the precursors; (ii) a low melting point; (iii) a significant thermal coefficient of solubility; (iv) low volatility, (v) inertness to the precursors and the reaction container (crucible); and (vi) ease of removal after crystal growth through solvent dissolution or vacuum sublimation.^140^ For oxides (and thus vanadates), flux growth utilizes a high-temperature melt of simple inorganic oxides and fluorides including but not limited to PbO, PbF_2_, BaO, BaF_2_, Bi_2_O_3_, Li_2_O, Na_2_O, K_2_O, KF, B_2_O_3_, P_2_O_5_, and MoO_3_.^144^ Some reports have also reported success in obtaining single crystals using a mixture of these fluxes to obtain an even lower melting eutectic.^140^

Importantly, the flux should also not react with the reaction container. Intermetallics are often grown using metallic fluxes and ceramic crucibles such as those made of alumina, zirconia, and boron nitride. Oxides, on the other hand, typically utilize metallic crucibles such as those made from Pt, Ti, and Nb. Flux growths are typically performed in sealed ampules or in atmosphere-controlled furnaces for reactions involving air-sensitive precursors or where tight control of the oxidation state is required. While growing single crystals from a flux does not require specialized equipment and allows for relatively low reaction temperatures, thermal gradients within fluxes make it difficult to obtain large crystals and reactive flux constituents are often incorporated within crystals as inclusions or as substitutional or interstitial dopants.

Techniques for flux growth of single crystals can be differentiated based on reaction temperature and the method of extracting the crystal from the flux.^145^ At temperatures below 1200 °C, most polycrystalline powders and precursors are loaded into a crucible from highest melting point constituent at the bottom to lowest melting point at the top.^146^ This allows the low melting point precursor to melt first and flow over the higher melting compounds and homogenize the melt. Typically, a 10:1 ratio of flux to precursors is used for crystal growth and optimized depending on the crystal morphology.^140^ The crucible is then placed within an evacuated fused amorphous silica ampule that has already been filled with shards of silica to ensure that the crucible is not in contact with the bottom of the ampule. This lack of contact prevents the ampule from shattering due to the differential thermal expansion of the crucible and ampule. Finally, some quartz wool is placed above the crucible which acts as a filter during flux removal. Indeed, once the reaction has reached completion, the ampule is brought to temperatures still above the melting point of the flux so that when the ampule is inverted, the flux passes through the quartz wool filter but the newly formed crystals are retained on the filter.^145,146^ The ampule is then centrifuged to separate the flux from the crystals. If quartz wool cannot be used due to interference with the reaction then the flux may also be chemically etched from the obtained single crystals. The limitation of this method, however, is that the etchant should have a thermodynamic preference to attack the flux over the crystals. Common etchants include NaOH or HCl, which can dissolve most metal fluxes.^146^ Nonmetallic fluxes, which are often used to produce oxide single crystals, can be more difficult to remove since the flux can have a similar reactivity with the etchant as the obtained oxide single crystal. If an ideal etching solvent cannot be found, then the flux may also be manually removed from the crystals through careful mechanical separation or even focused ion beam milling. Reactions requiring temperatures higher than 1200 °C or greater than 5 mL of flux are not viable in fused silica ampules and a different secondary container must be considered. Introducing a seed crystal that is rotated or pulled has led to the development of flux growth derivative techniques such as slow-cooling bottom growth, top-seeded solution growth,^147^ top-seeded vertical temperature gradient techniques, growth by traveling solvent zone, and flux evaporation, among others.^144^

Flux growth is most often used to obtain single crystals of ternary vanadates. However, these reactions often require strict optimization and precise control of reaction conditions because of the increased complexity of ternary systems. For example, InVO_4_ melts incongruently at 1134 °C and In_2_O_3_ readily sublimes in air above 1100 °C and therefore is an excellent candidate for flux growth.^148^ One ideal flux to be used in this reaction is a self-flux, i.e., one comprising the precursors necessary for the formation of the target phase. The In_2_O_3_–V_2_O_5_ phase diagram manifests a low-temperature eutectic at 678 °C when In_2_O_3_ and V_2_O_5_ are mixed in a 1:1 ratio; however, experiments showed that the mixture did not melt, even when heated to 1000 °C. Instead, Cu_2_V_2_O_7_ was utilized as a flux since pyrovanadates have been shown to be good fluxes in the literature.^149^ Cu_2_V_2_O_7_ melts congruently at ≈785 °C and Cu^2+^ ions are unlikely to substitute for V^3+^ ions in InVO_4_. A 1:4 (w/w) ratio of the precursors and Cu_2_V_2_O_7_ was mixed and loaded into Al_2_O_3_, ZrO, and Pt crucibles and heated to 950 °C and dwelled for 12 h, slowly cooled to 800 °C (a temperature above the melting point of the flux) at a rate of 2.5 °C/h, and held at this temperature for 12 h to promote crystallization after which the flux was decanted.^148^ While the solution reacted with both the alumina and zirconia crucibles, the Pt crucible was found to be optimal for obtaining large single crystals of InVO_4_, as shown in Figure 19a.^148^ The crystals do present surface impurities of Cu_2_V_2_O_7_ with slight Pt inclusions (Figure 19b–d) but the Pt and Cu content was <0.01 and 5.13 wt %, respectively. While functional, it is clear that flux growth of vanadates requires careful consideration of the flux selection as well as the heating profile and compatibility of all components with the crucible.

**Figure 19:**
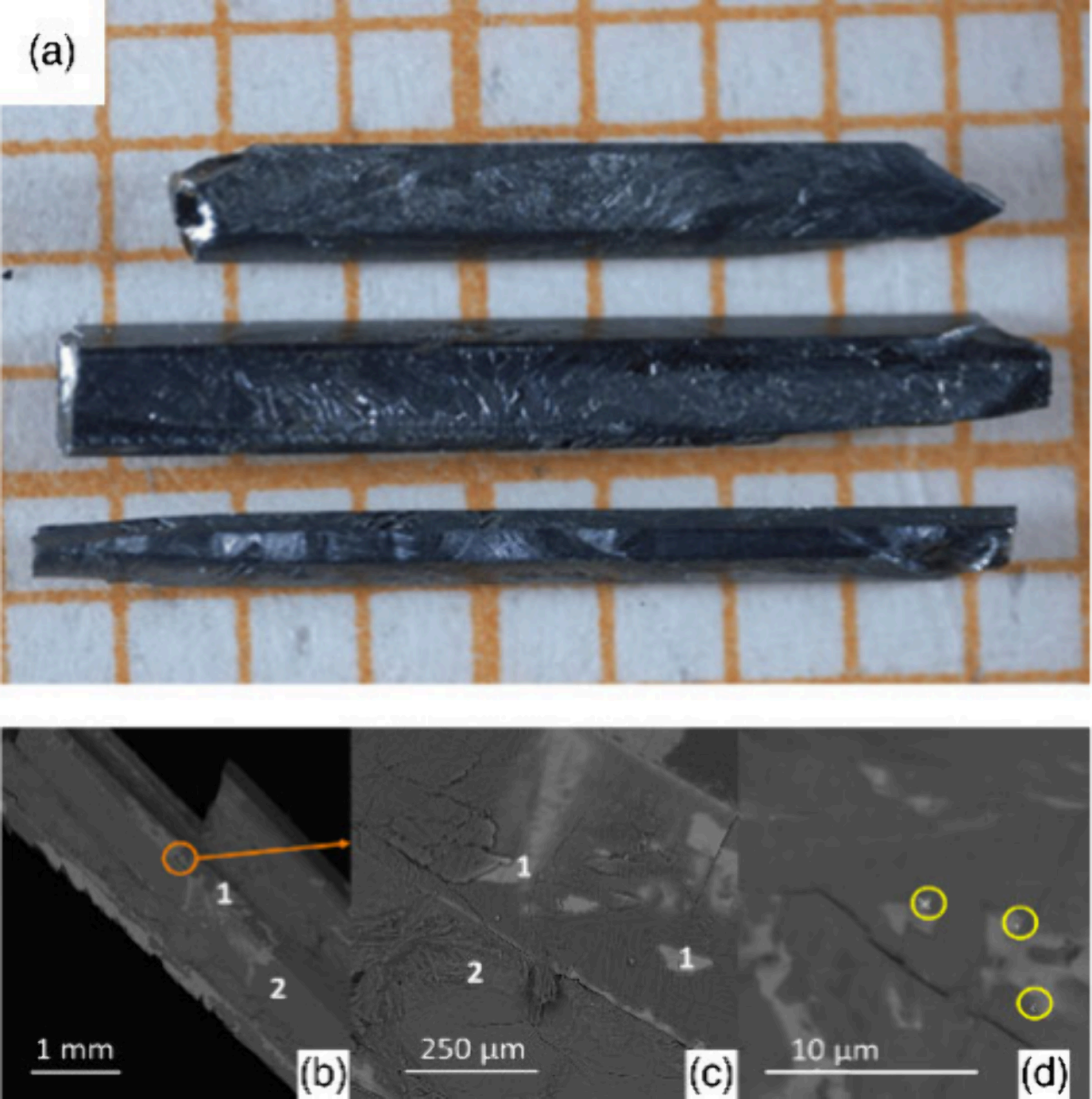
(a) Typical crystals of InVO_4_ obtained from flux growth after being extracted from a Pt crucible. A back scattering electron microscopy image of the (b) InVO_4_ crystal and (c) a zoomed in image of the crystal surface marked by the orange circle in panel (b) where 1 denotes regions of InVO_4_ and 2 denotes a region of the flux utilized—Cu_2_V_2_O_7_. (d) A scanning electron microscopy image of the InVO_4_ crystal where inclusions of Pt arising from contamination with the Pt crucible are highlighted by the yellow circles. This figure was reproduced from reference 148 under a CC BY license. Copyright 2023 Voloshyna et al.

Flux growth of vanadates has also been successful when a polycrystalline powder of the desired phase is loaded into the crucible with the flux instead of synthesizing the desired phase *in situ*. Presynthesized polycrystalline Li_2_VO_4_ and the chosen flux, a 69:26:5 molar ratio of LiCl:Li_2_MoO_4_:LiBO_2_, was loaded into a Pt crucible that was placed and sealed inside of an evacuated silica ampule that was carbon coated through the pyrolysis of acetone.^150^ The carbon coating was performed to prevent a reaction of lithium with the ampule. The flux was selected due to its low ternary eutectic point at 490 °C; the lithium-rich components serve to prevent lithium deficiency within the grown crystals and mitigate the high volatility of lithium. Moreover, the hexavalent Mo in Li_2_MoO_4_ prevents redox reactions that could result in mixed-valent vanadium in Li_2_VO_4_. Ratios of flux:Li_2_VO_4_ were attempted (10:1 to 1:10) for crystal growth where a 1:1 ratio was identified as the ratio that yielded the best solubility and crystal growth. The solubility was estimated from the weight ratios of crystals larger than 150 μm in the final product upon quenching at various temperatures between 600 and 740 °C.^150^ By 800 °C, Li_2_VO_4_ completely dissolves and as such, the ideal growth conditions were determined to be to heat the mixture at 800 °C for 10 h, cooling to 450 °C over 72 h and cooling to room temperature for 10 h. The grown Li_2_VO_4_ crystals were then separated from the flux by rinsing with a low concentration LiOH solution and acetone. The obtained crystals were lustrous and black with well-developed (111) faces.^150^ The obtained crystals had typical dimensions of 0.5 mm × 0.5 mm × 0.5 mm, which were large enough for subsequent analysis further depicting the utility of flux growth in obtaining high-quality vanadate single crystals.

#### 2.2.7 Chemical Vapor Transport

Chemical vapor transport is a linchpin for synthesizing high-quality single crystals of vanadium oxides for applications in electronics, optoelectronics, and energy-efficient coatings.^151^ This technique leverages reversible gas-phase reactions facilitated by transport agents to enable the dissolution and recrystallization of solid precursors within a temperature gradient.^152^ Vanadium oxides, characterized by their inherently low vapor pressures and complex polymorphism, are particularly amenable to chemical vapor transport, as the method circumvents challenges associated with conventional solid-state synthesis.^153^ A key advantage of chemical vapor transport lies in its ability to produce phase-pure, single-crystalline materials with well-defined morphologies, essential for probing structure–property relationships. Additionally, these methods produce single crystals with fewer defects at lower processing temperatures than traditional ceramic methods, while also enabling epitaxial control of growth for thin films—all essential to obtaining high-quality crystals to examine intrinsic strong electron correlation phenomena.

##### 2.2.7.1 Rationalizing the Choice of Transport Agent

A central factor in CVT crystal growth is the choice of transport agent, which directly shapes the oxygen chemical potential (mO_2_) and therefore controls the thermodynamic stability of different vanadium oxide phases during transport-driven recrystallization.^154^ Vanadium oxides are unusually sensitive to redox conditions because of their wide range of accessible oxidation states. As a result, fine-tuning the oxygen partial pressure (pO_2_) is critical, not just for achieving phase-pure products, but also for stabilizing distinctive VO_*x*_ synthetic targets under specific growth conditions. The phase outcome in CVT is not dictated by pO_2_ alone but is a function of interdependent variables: the chemical identity and concentration of the transport agent, the imposed temperature gradient, and the stoichiometry of the source material. Each transport reagent sets up a distinct chemical environment, governed by its own equilibrium reactions and vapor pressures, which in turn determine which vanadium oxide phase is favored.^155^ Understanding the particularities of each transport agent and controlling these parameters is key to navigating the complex phase space of vanadium oxides to obtain specific synthetic outcomes.

TeCl_4_ emerges as the most versatile and broadly effective transport agent. This reagent establishes a stable gas-phase equilibrium involving TeOCl_2_ and Cl_2_, affords moderate oxidative strength, and yields expansive control over pO_2_ ranging from 10^–25^ to 1 bar. As such, this reagenet has been used to grow varying vanadium oxide stoichiometries except VO.^155,156^ The phase boundaries and bidirectional transport limits of VO_2_ with TeCl_4_ have been well characterized,^157^ supported by the identification of dominant gas species such as VOCl_3_, TeOCl_2_, and TeO_2_, which vary with oxygen pressure.^156^ However, this versatility comes with a caveat: selectivity can decrease if experimental parameters are not strictly controlled. The partial pressure of TeCl_4_ itself influences phase formation as shown in the Figure 20; higher TeCl_4_ vapor pressures tend to stabilize volatile vanadium oxychlorides (e.g., VOCl_3_, VO_2_Cl), which thereby stabilizes higher oxidation states such as V_2_O_5_ or provides a means of selecting for specific Magnéli phases such as V_9_O_17_ over V_8_O_15_, based on subtle gas-phase dynamics.^158^ Lower TeCl_4_ pressures favor lower-valent or intermediate Magnéli phases such as V_4_O_7_ or V_6_O_13_, which are sensitive to relatively minor changes in oxygen fugacity.^156,158^ These observations underscore the importance of optimizing both the identity and concentration of the transport agent, as TeCl_4_ vapor pressure controls not only transport kinetics but also modulates phase boundaries by exerting some control over the local redox environment.

**Figure 20:**
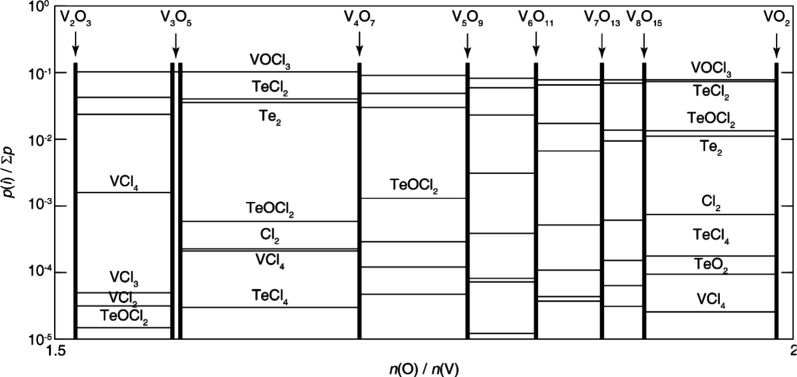
Gas-phase composition over different vanadium oxides in equilibrium with TeCl_4_ at 900 °C as produced by Oppermann et al.

Other transport agents offer varying degrees of utility and specificity. Cl_2_ is strongly oxidizing and often causes irreversible oxidation, restricting its use mainly to higher-valent phases such as V_2_O_5_. I_2_ offers milder oxidation conditions suited to VO_2_ synthesis but lacks the versatility of TeCl_4_ in stabilizing intermediate oxides. HCl, despite being less oxidizing, is moisture-sensitive and provides inconsistent redox control, which oftentimes leads to less predictable phase outcomes.^156,159^

Considering reducing transport reagents, NH_4_Br is perhaps the most prominent, and engenders partial or complete reduction of V_2_O_5_ to V_3_O_7_.^160^ When used alone, NH_4_Br selectively transports phase-pure V_3_O_7_ by lowering the oxygen partial pressure, demonstrating that transport agents can directly affect vanadium oxidation states. Consequently, NH_4_Br is unsuitable for high-valent oxides like V_2_O_5_ when phase purity is required but can be used to access reduced phases. In contrast, NH_4_Cl exhibits greater stability under oxidative conditions and enables phase-pure V_2_O_5_ transport when combined with Cl_2_ or H_2_O.^160^

A more recent approach involves organometallic precursors such as vanadium oxo-tri-isopropoxide, which can be used in chemical vapor deposition. These compounds contain preformed V–O bonds, which in principle allows finer control over oxidation states and coordination environments, which yields improved phase selectivity in VO_*x*_ synthesis.^92^ For instance, V_2_O_5_, VO_2_(M), V_3_O_7_, and V_7_O_13_ were selectively synthesized by tuning only the deposition temperature requiring neither postannealing nor redox adjustments. Mathur and co-workers grew nanocrystalline vanadium oxide films directly using this compound as a single-source precursor.

##### 2.2.7.2 Mechanistic Overview of a Chemical Vapor Transport Growth

The chemical vapor transport process begins with the sublimation of vanadium oxide precursors such as vanadium oxychloride (VOCl_3_) or vanadium tri-isopropoxide (VO­(O^*i*^Pr)_3_), which volatilize and migrate along a thermal gradient to a cooler deposition zone.^151,152^ These precursors undergo heterogeneous reactions, leading to nucleation and epitaxial growth on substrates.^161,162^ Precursor chemistry critically dictates phase selectivity and crystallinity. VOCl_3_ enables growth of VO_2_ and V_2_O_5_ because of its high volatility and oxygen-rich decomposition pathways. Conversely, VO­(O^*i*^Pr)_3_, with alkoxide ligands, favors reduced phases like V_2_O_3_ under inert atmospheres.^163^ Vanadyl acetylacetonate (VO­(acac)_2_) facilitates low-temperature (300–500 °C) synthesis of V_2_O_5_ nanostructures (e.g., nanowires, nanospheres) with high surface-to-volume ratios.^164^ The basic reaction for the growth of different morphologies is provided in eq 5.

5 $$\mathrm{VO}{\left(\mathrm{acac}\right)}_{2}+O_{2}\to V_{2}O_{5}+CO_{2}+H_{2}O$$

Wang and co-workers obtained different V_2_O_5_ morphologies—nanocluster films, nanowires, and nanospheres—by controlling the distance­(13–30 cm) from the precursor source and the growth pressure (1–456 Torr).^165^ Substrate choice has also been known to impact epitaxial alignment. For example, single-crystalline sapphire (Al_2_O) yields (010)-oriented VO_2_ films as a result of lattice matching,^166^ whereas nanoimprinted silicon enables site-specific nucleation of VO_2_ nanowires.^157^

The growth kinetics and final microstructure are governed by parameters such as the temperature gradient, reactor pressure, carrier gas flow rate, and precursor reactivity. For instance, temperature gradients (Δ*T*) critically influence phase formation. For VO_2_, a Δ*T* of ∼100 °C between source and deposition zones ensures monoclinic-to-rutile phase transitions, whereas deviations risk mixed-phase products. Atmospheric-pressure chemical vapor deposition yields VO_2_ films at 400–550 °C, whereas low-pressure CVT yields high-aspect-ratio V_2_O_5_ nanowires.^153^ Reactor atmosphere (inert versus reducing) modulates oxygen stoichiometry. The presence of hydrogen stabilizes lower oxidation states (e.g., V^3+^ in V_2_O_3_), whereas argon or nitrogen yields stoichiometric VO_2_.^151,167^ In general, higher temperatures (400–700 °C) favor VO_2_ thin film formation, whereas lower temperatures promote V_2_O_3_ growth.^168^

Gas-phase dissolution in chemical vapor transport hinges on redox reactions with halogens and oxide decomposition as per eq 6:

6 $$M_{a}O_{1/2a}\left(s\right)⇆M_{a}\left(s\right)+\frac{1}{4}aO_{2}\left(g\right)$$

where *M* is a metal and *a* is the oxidation state of the metal. Gas-phase dissolution can also utilize metal halogenation following eq 7

7 $$M_{a}\left(s\right)+\frac{1}{2}aX_{2}\left(g\right)⇆MX_{a}\left(g\right)$$

where *X* is either Cl, Br, or I. Chlorine is the preferred halogen in light of the thermodynamic stability of vanadium chlorides (e.g., VCl_4_) and favorable equilibria (10). However, chlorine’s tendency to overoxidize vanadium or form nonvolatile oxychlorides (e.g., VOCl) represents a significant constraint.^157^ To circumvent this, *in situ* halogen generation via decomposition of TeCl_4_ offers an additional degree of control as per eqs 8–13.^156,152,168^

8 $${\mathrm{TeCl}}_{4}\left(g\right)⇆{\mathrm{TeCl}}_{2}\left(g\right)+{\mathrm{Cl}}_{2}\left(g\right)$$

9 $${\mathrm{TeCl}}_{2}\left(g\right)+\frac{1}{2}O_{2}\left(g\right)⇆{\mathrm{TeOCl}}_{2}\left(g\right)$$

10 $${\mathrm{TeOCl}}_{2}\left(g\right)⇆\mathrm{TeO}\left(g\right)+{\mathrm{Cl}}_{2}\left(g\right)$$

11 $${\mathrm{TeO}}_{2}\left(g\right)⇆\frac{1}{2}{\mathrm{Te}}_{2}\left(g\right)+O_{2}\left(g\right)$$

12 $${\mathrm{Te}}_{2}\left(g\right)⇆2\mathrm{Te}\left(g\right)$$

13 $${\mathrm{Cl}}_{2}\left(g\right)⇆2\mathrm{Cl}\left(g\right)$$

These reactions that release Cl_2_ as a byproduct enable controlled transport of metastable phases such as the Magnéli series (V_*n*_O_2*n*–1_, 3 ≤ *n* ≤ 9) (Figure 21) where V_6_O_11_ can be made under precise oxygen partial pressures as can be seen in Figure 22a from the Barogram plot.^152,156,168^ In fact, chemical vapor transport is a preferred method for synthesizing Magnéli/Wadsley phases of vanadium oxides by dint of the exceptional compositional precision and phase selectivity that it affords. It is particularly effective for targeting specific VO compositions because halogen transport agents (like TeCl_4_ or VCl_4_) create volatile intermediates with different transport rates depending on oxidation states, enabling selective crystallization of specific phases. The temperature gradient in these techniques creates distinct thermodynamic conditions that enable precise control of the V:O ratio, which is crucial for accessing the narrow stability windows of specific Magnéli (V_*n*_O_2*n*–1_) or Wadsley (V_2*n*_O_5*n*–2_) phases. Oppermann and co-workers observed that elevated temperatures promote smoother Magnéli phase surfaces (Figure 21a), whereas prolonged transport results in distinct phase domains (e.g., V_2_O_3_/V_3_O_5_) due to structural mismatches: V_2_O_3_ (hexagonal), V_3_O_5_ (monoclinic), and V_4_O_7_ (triclinic). In contrast, monoclinic VO_2_ grows epitaxially on monoclinic V_6_O_13_ at lower temperatures (Figure 21b). Similarly, monoclinic V_6_O_13_ (Figure 21c), orthorhombic V_2_O_5_ (Figure 21d), and VO_2_ (Figure 21e) form isolated crystals, highlighting the role of lattice symmetry in phase segregation.^156^

**Figure 21:**
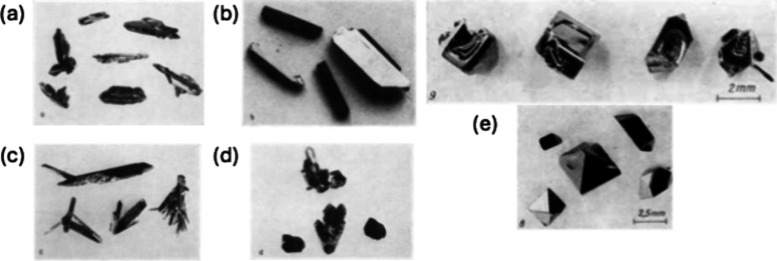
Single crystals of (a) Magnéli phases, (b) V_6_O_13_, (c) VO_2_ on V_6_O_13_, (d) V_2_O_5_, and (e) VO_2_ grown using TeCl_4_ as transport reagent. Panels (a)–(d) have been adapted from reference 156. Copyright 1977 Wiley. Panel (e) was adapted from reference 169. Copyright 1975 Wiley.

**Figure 22:**
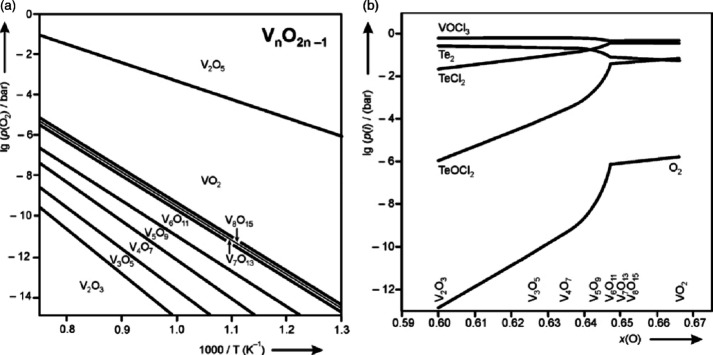
(a) Barogram of the vanadium–oxygen system showing phase equilibria of V_2_O_5_, VO_2_, V_2_O_3_, and the Magnéli phases as a function of oxygen partial pressure (pO_2_) and temperature. (b) Experimental gas-phase composition for TeCl_4_-mediated transport of V_2_O_3_ and V_6_O_11_ at 1000 °C. Reproduced with permission from reference 152. Copyright 2012 De Gruyter Bill.

The controlled synthesis of vanadium oxides such as VO_2_, V_3_O_5_, V_6_O_13_, V_3_O_7_ by chemical vapor transport hinges on the interplay of transport agent reactivity, temperature gradients, and thermodynamic equilibria.^156,169–171^ Schäfer’s foundational work and subsequent advancements in transport modeling provide critical tools to address challenges unique to these materials.^168^ Schäfer’s transport equation forms the backbone of chemical vapor transport kinetics, linking partial pressure gradients, temperature, and ampule geometry to the transport rate. For vanadium oxides, the equation is expressed in eq 14

14 $$ṅ=\frac{\Delta p\left(VOCl_{3}\right)}{\sum p}.\frac{T^{0.75}.q}{s}.0.6\times 10^{-4}$$

where Δ*p*(*VOCl*_3_) is the partial pressure difference of the dominant vanadium species (e.g., VOCl_3_) between the source ((*T*_1_) and sink (*T*2)), ∑_*p*_ is the total pressure, *T* is the mean temperature (K), and *q*/*s* represents the cross-sectional area-to-length ratio of the ampule.^156,172^ This equation underscores the importance of optimizing temperature gradients and ampule design to achieve efficient transport of phases such as VO_2_ or V_3_O_5_.

Vanadium oxides often exhibit homogeneity ranges (e.g., V_*n*_O_2*n*–1_ Magnéli phases), necessitating the extended transport model.^173^ This framework relates the component fluxes *J*(*V*), *J*(*O*) to the composition of the deposited phase (eq 15)­

15 $$\left(\frac{n\left(O\right)}{n\left(V\right)}\right)=\frac{J\left(O\right)}{J\left(V\right)}$$

where the flux ratio is governed by equilibrium partial pressures of gas species (e.g., VOCl_3_, TeCl_2_) at source and sink conditions. For example, Figure 22a^168^ illustrates the oxygen coexistence pressures (*p*(O_2_)) for V_*n*_O_2*n*–1_ phases, which dictate the O/V ratio in the deposited solid. By coupling this barogram with the extended model, the composition of mixed-valent oxides can be predicted.^156,168^

The gas-phase composition during the chemical vapor transport growth of vanadium oxides, as depicted in Figure 22b,^168^ reveals a shift in dominant tellurium species from Te_2_ (for oxygen-poor V_2_O_3_) to TeCl_2_ and TeOCl_2_ (for oxygen-rich VO_2_). This transition drives distinct transport mechanisms.

For V_2_O_3_, the transport mechanism involves eq 16

16 $$V_{2}O_{3}\left(s\right)+3\mathrm{TeC}l_{2}\left(g\right)\overset{\mathrm{yields}}{\to }2\mathrm{VOC}l_{3}\left(g\right)+Te_{2}\left(g\right)+O_{2}$$

where Te_2_ dominates, favoring low oxygen fugacity. For VO_2_, the mechanism can be understood following eq 17

17 $$4VO_{2}\left(s\right)+10\mathrm{TeC}l_{2}\left(g\right)\overset{\mathrm{yields}}{\to }4\mathrm{VOC}l_{3}\left(g\right)+\mathrm{TeOC}l_{2}\left(g\right)+3Te_{2}$$

where TeOCl_2_ is the dominant species at higher *p*(O_2_).^156,168^

TeCl_4_’s dual role as a chlorinating agent and oxygen buffer enables selective phase deposition. Computational tools^174^ have become available to simulate these equilibria, resolving challenges such as incongruent dissolution and nonstationary transport in multiphase systems. For instance, variations in O/V ratios during dissolution (“incongruent dissolution”) are addressed by the extended model’s stationarity condition provided in eq 18 which is derived from the fluxes *J*(*O*) and *J*(*V*) of the individual components of the system. Eq 18 is useful for predicting the composition of crystals with homogeneity range and multiphasic mixtures deposited at the sink in a quasi-stationary transport and the partial pressure difference between source and sink can be correlated to the barogram provided in Figure 22(b).^173^

18 $${\left(\frac{p^{*}\left(O\right)-x_{\mathrm{sink}}.p^{*}\left(V\right)}{p\left(\mathrm{TeC}l_{2}\right)}\right)}_{\mathrm{source}}={\left(\frac{p^{*}\left(O\right)-x_{\mathrm{sink}}.p^{*}\left(V\right)}{p\left(\mathrm{TeC}l_{2}\right)}\right)}_{\mathrm{sink}}$$

Next, the sequential deposition of phases (e.g., VO_2_ before V_6_O_13_) is managed by iteratively recalculating equilibrium pressures and fluxes,^174^ ensuring quasi-stationary transport.^175^

Integrating Schäfer’s transport equation with the extended transport model enables precise control over the chemical vapor transport of vanadium oxides, facilitating the synthesis of phase-pure high-quality single crystals for applications in energy storage and electronics. Future prospects include plasma-assisted chemical vapor transport to access metastable phases, further expanding the utility of these approaches.^176^

Ultimately, chemical vapor transport distinguishes itself through several critical advantages that have positioned it at the forefront of advanced materials synthesis. Unlike traditional high-temperature growth techniques, chemical vapor transport provides exceptional control over crystal formation, enabling the synthesis of materials with minimal defects and tailored structural characteristics. One of the most significant attributes of chemical vapor transport is its ability to produce high-quality single crystals with minimal defects and impurities. The method systematically suppresses excess nucleation, facilitating the growth of large, nearly defect-free crystals. This is particularly crucial for vanadium oxides like VO_2_, where structural perfection directly influences critical properties such as metal–insulator transitions and electronic behavior. Research has demonstrated that CVT-grown crystals exhibit sharp phase transitions, such as the M1–M2–R structural transformation in VO_2_,^161^ which serves as a testament to the method’s capability to generate materials with exceptional structural integrity. The chemical vapor transport method has proven to be remarkably versatile. For instance, studies have successfully produced molybdenum-doped VO_2_ single crystals with compositional variations up to *x* = 0.60 have been grown, demonstrating the method’s adaptability in creating complex, tailored material systems.^177^ This flexibility has enabled researchers to investigate structure–property relationships and develop materials with precisely tunable characteristics. The low defect density makes CVT-grown single crystals ideal for applications demanding high-precision substrates, including advanced thin-film technologies and quantum electronic devices.^178^

A distinctive feature of chemical vapor transport lies in its unprecedented control over material morphology and phase transition properties. Researchers have utilized this method to grow VO_2_ thin films with sharp semiconductor-to-metal transitions near 340 K, which makes them ideal for thermochromic applications.^179,180^ By strategically employing transport agents like iodine (I_2_) or vanadium trichloride (VCl_3_), the crystal growth can be precisely tuned.^181^ Chemical vapor transport has also been used to create complex composite structures, such as core–shell VO_2_@ZnO nanotetrapods.^182^ The continued refinement of chemical vapor transport techniques—through improved precursor selection, optimized deposition conditions, and sophisticated substrate engineering—will undoubtedly unlock new frontiers in navigating the vast design space of binary, ternary, and more complex vanadium oxides with precise site-selective modification.

#### 2.2.8 Molecular Beam Epitaxy

Recent efforts to develop alternatives to complementary metal–oxide–semiconductor (CMOS) circuitry with brain-inspired computation have focused attention on vanadium oxides that undergo metal-to-insulator transitions (discussed further in detail below in Sections 3 and 4) as potential active elements for brain-inspired non-von Neumann computing. Their development and eventual application, however, hinges on the ability to synthesize high-quality thin films with atomic-level precision that show tunable transformation characteristics such as threshold voltage, conductance contrast, thermal coefficient of resistivity, and hysteresis.^8,9^ Molecular beam epitaxy (MBE) has emerged as a state-of-the-art technique for the preparation of thin films with explicit control over the epitaxial layer thickness and uniformity over large surface areas. Recent improvements in the technique have also made it possible to produce films with minimal defects with electrical and optical properties that rival and even outperform materials obtained from traditional crystal growth techniques.^183^

Molecular beam epitaxy is the process of growing thin, epitaxial films by processing beams of atoms or molecules in an ultrahigh vacuum environment incident upon a heated crystal with a nearly atomically clean surface.^183^ The beam containing the constituent atoms/molecules strikes the crystal substrate at a moderate temperature, which provides the necessary thermal energy for the arriving atoms to migrate over the surface to the lattice sites. Here, the ultrahigh vacuum not only minimizes contamination (i.e., defects) of the growing surface, but also allows the atoms/molecules to travel in a nearly collision-free path until they reach the substrate surface.^183^ Constituents that do not reach the substrate typically collide with the chamber walls, which are chilled to condense the atoms and effectively remove them from the system (Figure 23a). An attached reflection high energy electron diffraction (RHEED) gun is typically used to monitor the crystal structure of the growing film in real-time. The constituent atoms then form a crystalline layer commensurate or incommensurate with the substrate to produce an epitaxial film. This growth process has the advantage of being able to finely tune composition to produce crystalline interfaces that are nearly atomically abrupt based on a shutter, which when closed, turns the beam off almost instantly. This allows the composition of the beam to be changed in times shorter than that needed to grow a single atom layer of the film enabling modulated growth of heterostructures.^186^

**Figure 23:**
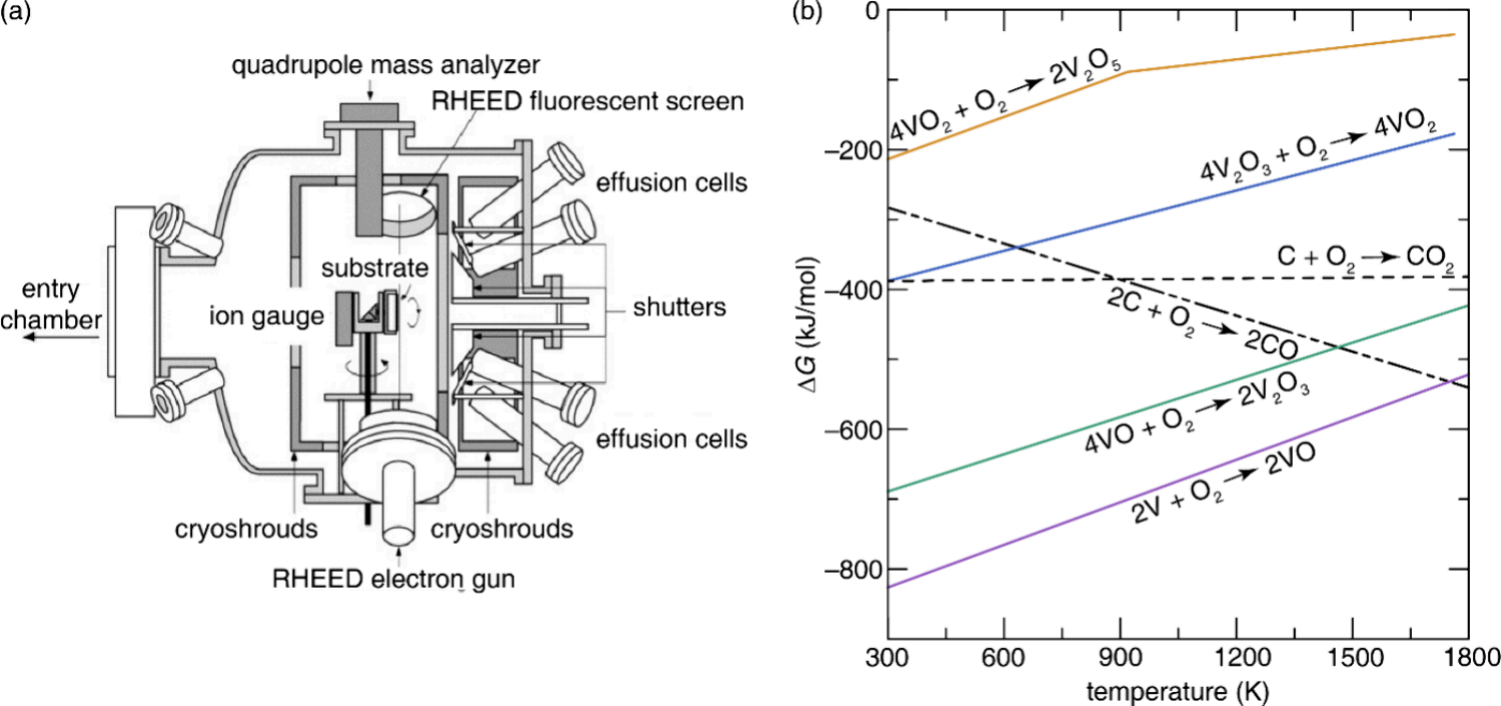
(a) A schematic of a typical molecular beam epitaxy system. (b) A vanadium–oxygen Ellingham diagram. Panel (a) was adapted with permission from reference 184. Copyright 2003 Elsevier. Panel (b) was reproduced with permission from reference 185. Copyright 2021 Elsevier.

One of greatest challenges associated with molecular beam epitaxial growth of oxide thin films is sourcing the highest purity precursor to minimize the number of defects while also balancing the temperatures required during synthesis. Many elemental precursors, while high in purity, have extremely low vapor pressures. This requires the reaction temperatures to be exceptionally high, typically >2000 °C, to produce enough vapor to engender feasible growth rates.^187^ Additionally, the elements require different partial pressures of oxygen in order to form the oxide. In molecular beam epitaxial growth, the oxygen partial pressure is inherently minimized to maintain ultrahigh vacuum conditions, meaning some elements with lower oxygen affinities may not fully oxidize, which renders compositional and stoichiometric control a substantial challenge.

Determining the correct partial pressure of oxygen required during synthesis becomes a particular challenge when growing vanadium oxide-based thin films through molecular beam epitaxy. Indeed, the partial pressure of oxygen must be precisely controlled to form the thin film with vanadium in the desired oxidation state. Fortunately, Ellingham diagrams for the vanadium–oxygen system have been developed to aid experimental design. Ellingham diagrams depict changes in the Gibbs free energy (Δ*G*) for the oxidation reaction of a particular species as a function of temperature. As the change in enthalpy (Δ*H*) and entropy (Δ*S*) essentially remain constant as a function of temperature (unless a phase change occurs), a plot of Δ*G* versus temperature can be drawn as a series of straight lines where Δ*H* is the slope and Δ*S* is the *y*-intercept.^188^ Typically, Ellingham diagrams are depicted with negative free energies of formation for metal oxides, an oxygen partial pressure of 1 atm, and all reactions are normalized to consume one mole of oxygen. The Ellingham diagram for different vanadium oxide species is provided in Figure 23b. This diagram is particularly helpful when deciding what temperature to anneal vanadium metal or a vanadium oxide to oxidize it to a particular oxidation state. For example, oxidizing VO to V_2_O_3_ will occur at temperatures below ≈1450 K since the Δ*G* of the oxidation reaction is more negative (i.e., favorable) than the oxidation of carbon to carbon monoxide at the same temperature region. These Ellingham diagrams can then be converted from units of Gibbs free energy to units of partial oxygen pressure, such as the diagram provided in Figure 22a, to help target specific oxygen pressures for molecular beam epitaxial growth of films.^189^ While it is clear that V_2_O_5_ is the thermodynamically stable phase at ambient conditions, the ultrahigh vacuum required for molecular beam epitaxy is better suited to grow thin films of VO_2_ and V_2_O_3_ because of the wider temperature and pressure regions where these phases are accessible.

Among the vanadium oxides, V_2_O_3_, is a promising candidate for neuromorphic devices in light of its metal-to-insulator transition (MIT) at 155 K and second-order MIT upon cooling from the paramagnetic insulting to paramagnetic metallic phase.^172^ Despite numerous efforts, however, there is an ongoing challenge of obtaining high-quality films of V_2_O_3_ with a resistivity ratio as high as single crystals. This performance mismatch largely stems from the formation of secondary impurity phases during growth or exposure to air, and the presence of bulk strain that originates from the mismatch between the V_2_O_3_ and substrate lattice and coefficient of thermal expansion. The most popular substrate for growing V_2_O_3_ thin films is sapphire (Al_2_O_3_) since they share the same *R* 3 *c* space group at room temperature but with a lattice mismatch of 4.1%. This mismatch induces strain where studies have shown that higher degrees of in-plane tensile strain results in a larger magnitude of change in the room-temperature resistivity.^190^

Recently, progress has been made with growing V_2_O_3_ on silicon substrates, which is more desirable for microelectronic applications and can still be utilized as an effective substrate given its 3-fold symmetry along the (111) planes. To identify the optimal growth conditions, films of V_2_O_3_ were grown on silicon at a variety of temperatures (60 °C, 100 °C, 400 °C, 700 °C, and 760 °C) and partial pressures of oxygen (1.1 × 10^–5^ Torr, 2.0 × 10^–5^ Torr, 2.2 × 10^–5^ Torr, 1.8 × 10^–6^ Torr, and 7.8 × 10^–8^ Torr).^172^ Unsurprisingly, films grown at temperatures below 100 °C remained amorphous, evidenced by featureless RHEED patterns (Figure 24a). Increasing the temperature to 400 °C yielded a RHEED pattern with streaks characteristic of thin film deposition and growth (Figure 24b). It can be deduced that reaction temperatures lower that 400 °C are insufficient to induce the formation of crystalline nuclei or accommodate the lattice mismatch with the Si (111) substrate; as such, the obtained films grow without discernible long-range order.^172^ Further increasing the temperature to 700 °C resulted in RHEED patterns with concentric rings indicative of a polycrystalline film (Figure 24c). Interestingly, *ex situ* grazing incident X-ray diffraction (GIXRD) and θ–2θ diffraction patterns of each of the thin films revealed that 400 °C and 1.1 × 10^–5^ Torr (sample #5) is optimal to obtain a film of V_2_O_3_, whereas the other growth conditions yielded films with impurities such as V_3_O_5_ and V_6_O_11_. High-resolution transmission electron microscopy (HR-TEM) of the V_2_O_3_ film (sample #5) reveals that the film comprises several crystalline domains with different in-plane orientations that are ≈40 nm wide (Figure 24d).^172^ The cross-sectional planes of two domains, the (1120) and the (3300), can be seen in the inset of Figure 24d, corresponding to two mutually orthogonal planes perpendicular to the (0006), which reveals a degree of degenerate epitaxial growth. Additionally, a 2 nm layer of amorphous SiO_*x*_ was also observed in the HR-TEM image, which is hypothesized to arise from the spontaneous oxidation of Si through the diffusion of oxygen atoms from the grown VO_*x*_ layer during deposition. Finally, the mosaicity in the thin film was determined to be 9°, which results in a broadening of the reciprocal lattice points (Figure 24e). This work shows that the growth of V_2_O_3_ films does not have to be limited to just sapphire substrates and that identifying the ideal substrate may help tune the electronic properties of the material and enable large-scale integration with wraparound Si circuitry.

**Figure 24:**
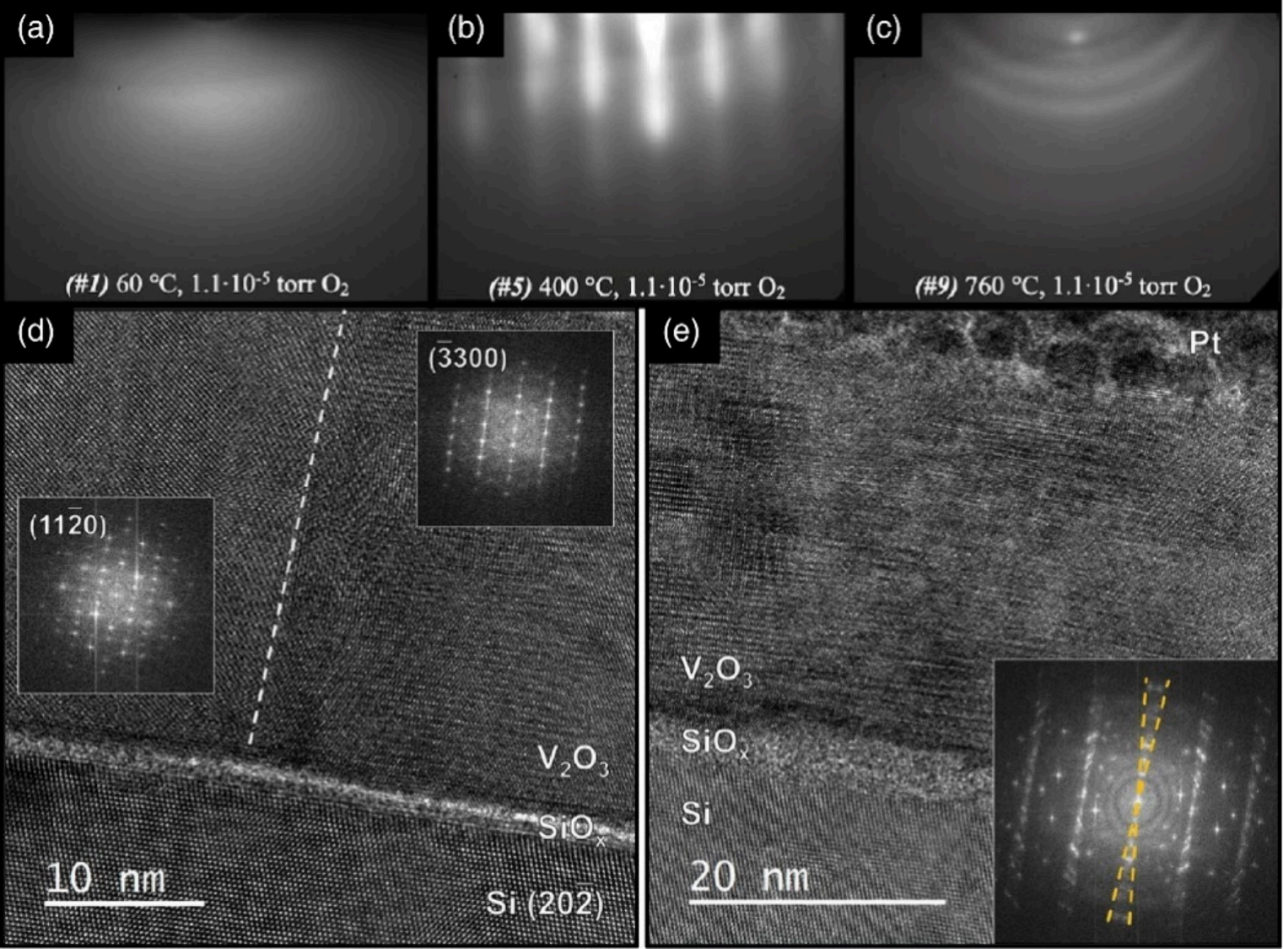
*In situ* reflection high-energy electron diffraction patterns of V_2_O_3_ grown at 1.1 × 10^–5^ Torr and (a) 60 °C, (b) 400 °C, and (c) 760 °C. (d) High-resolution transmission electron microscopy images of Si substrate with the deposited V_2_O_3_ film from (b) where the white line indicates the domain boundary. Insets are the fast Fourier transform patterns of the two epitaxial domains. (e) HRTEM image illustrating the mosaicity of the thin film where the FFT pattern is given in the inset. The yellow lines highlight the broadening of the reciprocal lattice of the V_2_O_3_ due to the mosaicity. This figure was reproduced with permission from reference 172. Copyright 2023 Elsevier.

Molecular beam epitaxy is a more challenging technique to prepare crystalline films of VO_2_ due to the precise control of the beam flux needed for the stoichiometric deposition of VO_2_ and the difficulty in isolating a single polymorph of VO_2_ from its complex phase space (Figure 9). Recently, two-inch films of VO_2_ (M) with uniform thickness were prepared through radio frequency plasma assisted molecular beam epitaxy using metallic vanadium powder on a Al_2_O_2_ (0001) substrate.^191^ Deposition occurred at 550 °C and a base pressure of <3 × 10^–9^ Torr and an oxygen flux with a precision of 0.1 standard cubic centimeters per minute (sscm). The influence of the oxygen flux was studied by varying the rate to 0.6 sscm, 1.3 sccm, 1.5 sscm, 1.8 sscm, and 2.1 sscm. At the lowest rate or 0.6 sscm, θ–2θ diffraction revealed the growth of hexagonal V_2_O_3_ films, whereas the highest rate of 2.1 sscm showed the presence of VO_2_ (M) as well as VO_2_ (B). The remaining intermediate rates all resulted in phase pure VO_2_ (M) films.

The thickness of the V_2_O_3_ and VO_2_ (M) films were determined through synchrotron-based X-ray reflectivity measurements (Figure 25b). This data depicts regular oscillations arising from interfacial interference. The thickness of the V_2_O_3_ and VO_2_ (M) film (*d*) was determined to be 63.2 and 113.8 nm consistent with^191^ thicknesses measured from SEM cross sections in Figure 25c,d for V_2_O_3_ and VO_2_, respectively.

**Figure 25:**
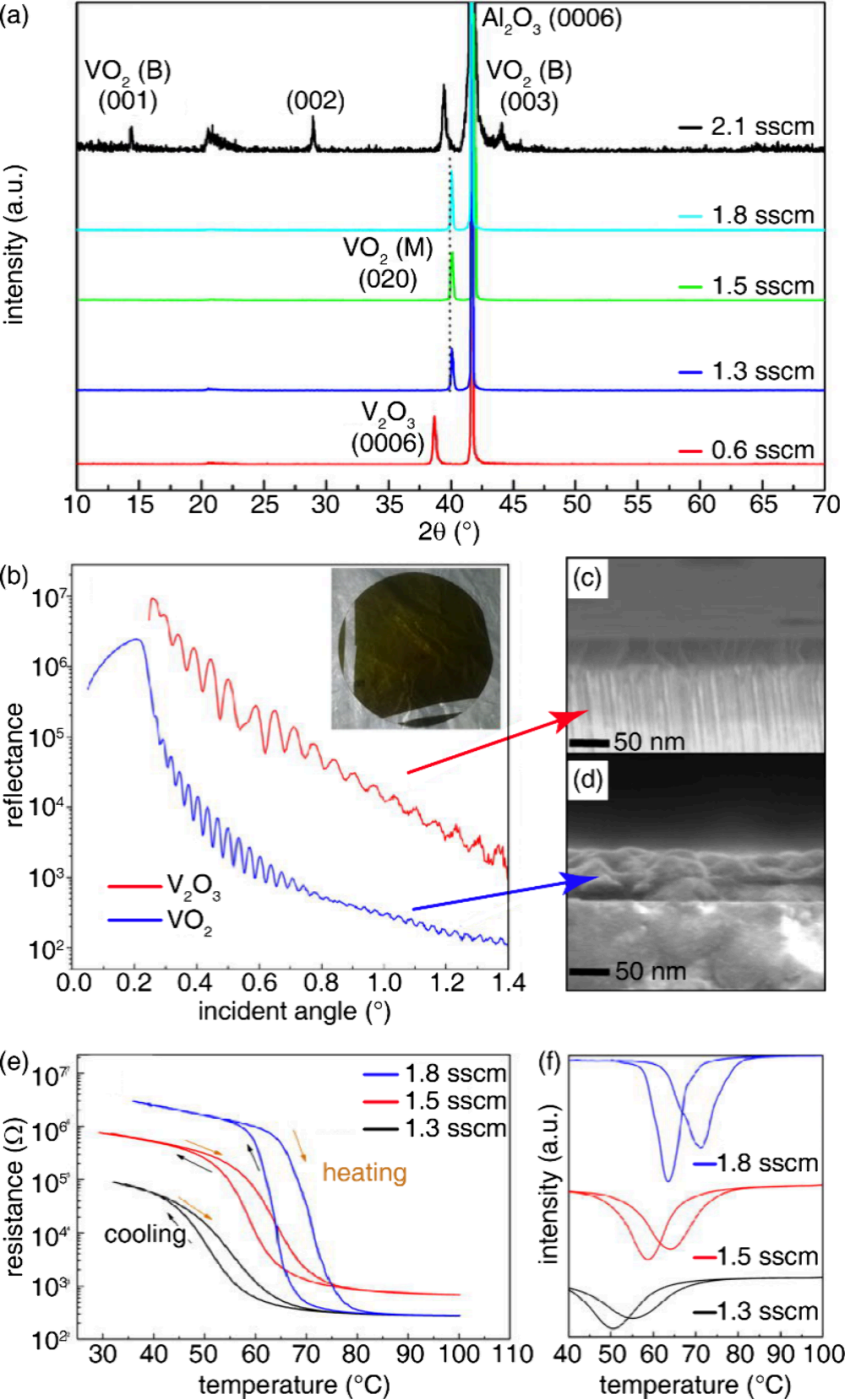
(a) Powder X-ray diffractograms of the prepared vanadium oxide thin films with different oxygen flux rates. (b) Synchrotron X-ray reflectivity measurements of V_2_O_3_ and VO_2_ with an inset depicting the VO_2_ epitaxial film. SEM cross sectional images of (c) V_2_O_3_ and (d) VO_2_. (e) Electric resistance curves for the VO_2_ films grown under different oxygen flux as a function of temperature. (f) The differential curves allow the transition temperature of the VO_2_ films to be determined. This figure was reproduced with permission from reference 191. Copyright 2013 AIP Publishing.

Finally, the different oxygen rates used in the growth of the VO_2_ (M) films can influence the number of defects present within the film, which, in this case, would be oxygen vacancies. Indeed, when measuring the resistance as a function of temperature, the VO_2_ (M) films clearly show that a decrease in the oxygen flux results in a decrease in the MIT temperature (Figure 25e,f).^191^ These results demonstrate that the oxygen partial pressure and flux needs to be precisely controlled to produce high-quality films with minimal defects and highly tunable electronic properties.

### 2.3 Modulating Synthetic Levers Toward Crystal Growth of Metastable Phases

The synthetic methods discussed above illustrate great success in the growth of vanadium oxide single crystals. However, it comes as no surprise that many of the phases discussed in Section 2.2. that were able to be isolated and stabilized are those that are thermodynamically stable (congruently melting) compounds. Much of the interest in these compounds arises from their complex free energy landscapes, populated with richly diverse metastable polymorphs. Such metastable polymorphs can be conceptualized as numerous shallow wells in proximity of a deep well that depicts the thermodynamically stable phase (Figure 3). A grand challenge in solid-state chemistry and materials science is to navigate the energy landscapes to arrive at a shallow well and to isolate a metastable polymorph under ambient conditions. Synthetic strategies to access metastable phases can be broadly classified along two axes: (a) achieving precise kinetic control over the path and angle of descent through the energy landscape by controlling synthetic parameters such as temperature, pressure, constrained topologies, structural homologies, and point defects to navigate “rugged” energy landscapes and to deposit the material in the shallow, metastable wells, or (b) through conditions of constrained equilibrium by accessing conditions wherein a metastable phase is the lowest energy configuration through the use of applied pressure, tensile or compressive stress, surface confinement, site-selective substitutional dopants, or other structural homologies, followed subsequently by quenching to trap the material even under conditions where it is no longer the lowest energy configuration.^10^ Beyond these traditional synthetic levers, one also must go beyond and consider other nonequilibrium conditions such as the role of particle sizes and surface confinement.^10^ This section describes some exemplary strategies that can be tuned during a crystal growth experiment to identify putative targets and to isolate single crystals of metastable polymorphs.

Isolating thermodynamically stable phases is relatively facile because their synthetic routes (i.e., high temperature sintering during conventional metallurgic and ceramic processing) are often predicated on providing an excess of energy that ensures that a system of atoms does not get trapped in shallow metastable wells. In contrast, “kinetically trapped” metastable phases are more commonly accessed through solution-phases syntheses or topochemical modifications, which enable more selective navigation of free energy landscapes and effectively utilize activation energy barriers inherent to structural transformations as a means of kinetic stabilization, as discussed more in depth in Section 5. As a case in point, the synthesis of single crystals of ζ-V_2_O_5_, a metastable polymorph of α-V_2_O_5_, first requires a hydrothermal reaction synthesis between α-V_2_O_5_ and AgC_2_H_3_O_2_ to prepare β-Ag_*x*_V_2_O_5_, which is isostructural to that of ζ-V_2_O_5_, but the Ag-ions arrayed along β interstitial sites along a quasi-1D tunnel defined by corner- and edge-sharing [VO_5_] and [VO_6_] polyhedra in ζ-V_2_O_5_.^192^ The Ag-ions are leached through topochemical treatment with either hydrochloric acid or sodium thiosulfate to obtain the metastable polymorph, ζ-V_2_O_5_. This topochemical treatment constrains the rugged energy landscape and directs the transformation toward ζ-V_2_O_5_ through the use of structural homologies in essence because the topochemical treatment removes Ag-ions but does not provide sufficient energy to the system to relax to its thermodynamic minimum.

Other strategies to entrap a metastable phase include constrained equilibrium where the metastable configuration is the most energetically favored polymorph such as through utilization of finite size effects (wherein surface energy differentials are sufficiently large to outweigh bulk free energy differentials), templated growth (utilizing differentials in nucleation energies), or applied temperature/pressure. As an example of the first mentioned effect, at nanoscale dimensions, surface and strain free energies are comparable in magnitude to bulk free energies. As such, for appropriate surface terminations, metastable polymorphs can be stabilized below a critical size as the energetically favored phase. This phenomenon has been observed such as in the stabilization of rutile TiO_2_ (*P*4_2_/*mnm*) under a critical diameter of ≈14 nm and tetragonal ZrO_2_ below a critical size of ca. 30–40 nm.^193^ Similarly, the use of LiFePO_4_ as a cathode material for Li-ion batteries is enabled by a metastable solid solution between the Li-poor and Li-rich phases of the olivine-type structure stabilized in nanometer-sized particles that ameliorates the extensive phase separation observed in larger particles.^194^

Templated growth typically refers to the nucleation and growth of materials utilizing epitaxially matched templates to favor nucleation of specific metastable phase rather than the most thermodynamically favored polymorph. This method utilizes lattice strain as a means to drive reaction trajectories such as to selectively suppress nucleation barriers for stabilization of metastable polymorphs. Thin films of VO_2_ (M) have been stabilized through coherent epitaxial strain where the *c*_*R*_ axis is elongated based on epitaxial matching with the M2 phase of TiO_2_.^195^ Judicious choice of substrates with epitaxial matching of in-plane lattice parameters provides one possible path to stabilizing metastable polymorphs.

Finally, the last method involves identifying the most stable synthetic parameters such as the appropriate temperature and pressure regimes required to stabilize the desired phase in a melt (in other words, conditions where the metastable phase represents the lowest energy configuration), and subsequently, quenching to ambient conditions as means to “lock” and kinetically trap the metastable phase and prevent it from converting to a thermodynamically stable phase. For example, the metastable β-V_2_O_5_ phase can be obtained by pressing a pellet of V_2_O_5_, wrapping it in tungsten foil and placing it in a high-pressure cell where the precursor is heated to 1073 K and 6.0 GPa of pressure.^33^ Once these conditions are obtained, the cell was quickly quenched at a rate of 6000 K/min.

An important question pertains on how to *a priori* access metastable phases. As a first point of consideration, possible metastable structures can be identified based on modern density functional theory calculations and genetic-algorithm- or ML-based explorations of structural design space. Methods such as particle swarm optimization, a computational approach based on optimization of a given problem through iterative improvements to a candidate solution with regard to a measurable quantity (energy), and resulting algorithms such as XtalOpt,^196^ USPEX, AlphaCrystal, and Crystal structure AnaLYsis by Particle Swarm Optimization (CALYPSO)^197^ enables the effective screening of structural design space using first-principles calculations to identify new metastable phases under conditions such as high pressure, which in turn can enable targeted approaches to the synthesis of these phases.^198^ For example, VH_2_ was identified as a phase that should exist at ambient temperatures and pressure. A high-pressure polymorph of VH_2_ was also predicted to exist at pressures above ≈47 GPa that is mechanically and dynamically stable as confirmed by elastic constant and phonon calculations and is metallic.^199^ Methods such as particle swarm optimization thus represent one possible approach for navigating rugged energy landscapes of vanadium oxides that may be able to significantly reduce the optimization time of synthetic pathways toward metastable phases by identifying target phases and their energetic and structural proximity to other accessible polymorphs.

As another example, density functional theory calculations have been combined with machine learning models to identify new thermodynamically stable and metastable phases. For instance, a support vector regression-based machine learning model was constructed to predict a compound’s formation energy as a proxy for their stability.^200^ The model was trained using calculated formation energies from databases such as The Materials Project^201^ and OQMD^202,203^ and had a resulting coefficient of determination, *r* ^2^ of 0.94 eV and a mean absolute error of 0.085 meV/atom.^200^ The trained machine learning model was then applied to perform formation energy predictions on a variety of composition spaces that was then cross-references with phases reported on the International Crystal Structure Database (ICSD). The model was not only able to successfully predict the existence of thermodynamically stable phases, but also metastable phases, by considering phases that had a predicted formation energy of between 0 and 50 meV/atom. The reliability of the model was investigated by generating a predicted phase diagram from the Y–Ag–In phase space, which suggested the existence of YAgIn that was not yet experimentally known. While synthesis of this 1:1:1 phase did not yield a phase pure compound, unindexed reflections belonging to a new phase were observed. Using the level rule to move away from known phases within the phase space led to the identification of a new phase, YAg_0.56_In_1.35_. As such, this first-principles driven machine learning model represents an exemplar for reducing the time and synthetic effort typically utilized in the identification of new phases. Machine learning informed tools such as *MatLearn* can generate binary and ternary phase diagrams, which contain predicted compositions that lie on or slightly above the convex hole and target synthesis of novel metastable compounds, such as those shown in Figure 26, that contain predicted compositions that lie on or slightly above the convex hole and target synthesis of novel metastable compounds.^204^ Combining first-principles calculations with advances in AI and machine learning will undoubtedly help the identification and isolation of new metastable phases as well as enable “synthesis design”—the planning of synthetic campaigns.

**Figure 26:**
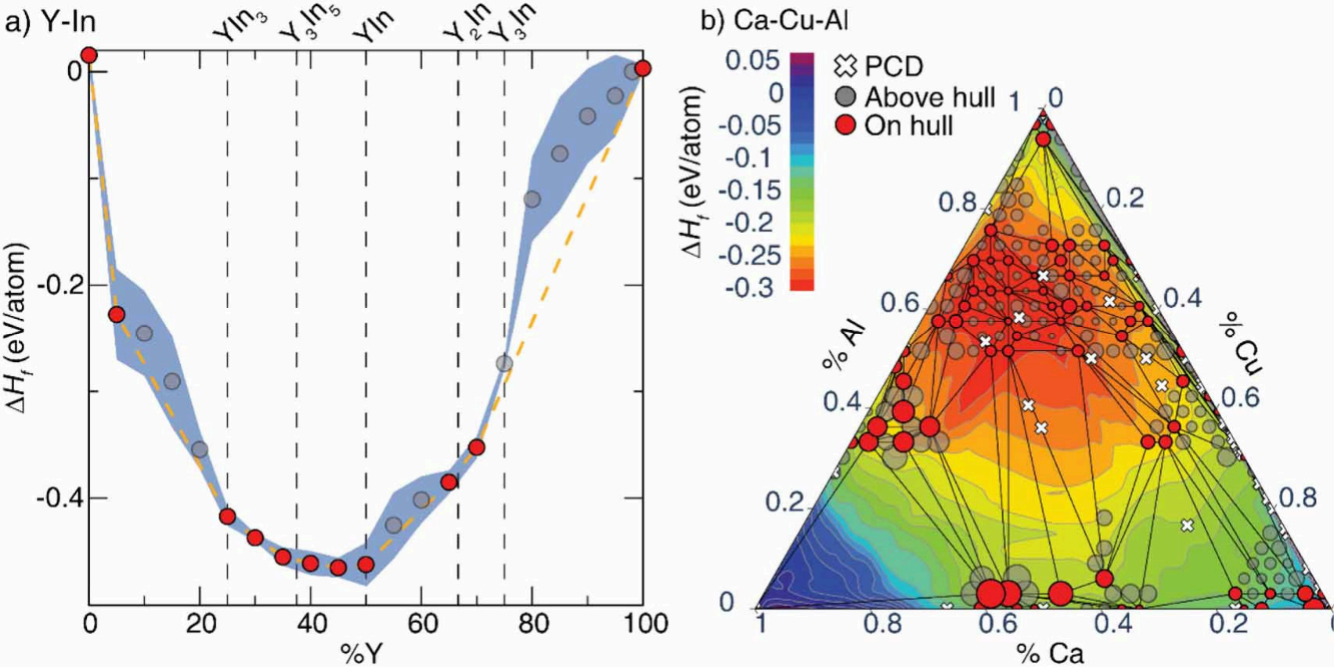
Phase diagrams that are predicted by *MatLearn* for the (a) Y–In binary system and (b) Ca–Cu–Al ternary system. The sizes of the circles in panel (b) correspond to the degree of uncertainty in the points on or near the convex hull.

## 3 The Electronic Structure and Electronic Instabilities of Vanadium Oxides

### 3.1 A Rugged Energy Landscape

The rugged free energy landscape illustrated in Figure 3 underpins the rich structural diversity and myriad solid–solid structural and electronic transformations manifested by vanadium oxides. Such transformations have elicited extensive attention as model systems for modulating and disentangling transformation characteristics through site-selective modification in condensed matter physics as well as for a broad range of applications spanning from neuromorphic devices alluded to in Section 1.1 to thermochromic films and infrared sensors. Vanadium oxides exhibit a vast and variegated array of transformation behaviors and in many cases descriptive models lag behind experimental observations. We begin our exploration of transformations by sorting these behaviors into two categories, diffusive transformations and diffusionless electronic instabilities, distinguished by the length scales of associated atomic displacements. Diffusive transformations involve intermediate- or long-range migration of ions, frequently coupled to chemical exchange between a crystal and its environment through an interface, and their kinetics are highly dependent on crystal size. They usually involve changes in composition and oxidation states either across the entire crystal or from one region to another such as with the propagation of an intercalation front.^205^ The involvement of diffusion processes across large length scales typically entails slow rates, albeit the initial distortions of the lattice to accommodate ion hopping can be at the time scale of shear waves. At the end of its trajectory, a diffusing ion rests in a site crystallographically equivalent to many others through which it migrated, so diffusive transformations can usually be halted at partial degrees of progress by removal of the driving stimulus.^206–208^

Electronic instabilities, on the other hand, involve small ionic displacements on the order of a unit cell length or less rather than long-range diffusion. This consideration forbids exchange of atoms with the environment, and thus the overall composition and connectivity of the crystal lattice is preserved. Individual atomic displacements are small and occur on the order of nanoseconds or shorter (often with ultrafast time scales),^209–211^ and their low activation energy ensures that they propagate rapidly once nucleated such as through transformation dislocations. Such transformations are thus generally abrupt and associated with a critical value of a state variable, such as temperature. First-order transitions can be rendered broader through weak disorder.^212,213^ Displacement trajectories involved in electronic transformations are unlikely to bridge symmetry-equivalent configurations but propagation beyond a few unit cells nevertheless requires propagation of a transformation dislocation;^214^ disrupted coordination and unit cell symmetry relationships are coupled to changes in the energy level, localization, orbital occupancy, spin alignment, and/or mobility of electrons. Displacement trajectories contain no degenerate points, so electronic transitions frequently display bistability, approximating electronic switches and underlying their promise as active elements in computing applications, which is the focus of Section 4.

We will first discuss the electronic states of vanadium oxides in terms of their structural elements and describe simple models of interactions between states which drive electronic structure transitions along a continuum from Peierls’-type or charge density wave transitions underpinned by one- or two-dimensional (un)­dimerization to electron-correlation-driven Mott transitions resulting from modulation of band filling or bandwidth.^8,9,215–219^ After a brief survey of phase transforming materials, we will examine transition mechanisms in binary oxides and strategies for modifying them, with an eye to the neuromorphic computing applications discussed in the next section. We finish with several examples of complex phase competition in ternary vanadium oxide bronzes studied using single-crystal techniques.

### 3.2 Electronic Structure of Vanadium Oxides: From Atoms to Bonds to Bands

Although the same quantum chemical principles underpin the formation both of bonds in molecules and of bands in extended solids, they are described respectively by chemists and physicists in somewhat different formalisms. In the spirit of Burdett^220^ and Hoffmann,^221^ we undertake in the first half of this section to develop concepts of electronic band structure starting from chemical bonding principles as a precursor to highlighting structural correlations to electronic instabilities. We will construct our electronic structure picture with examples pertaining to the vanadium oxide materials under consideration in this review (e.g., referring to V 3*d* orbitals), although the phenomena described here are not unique to this material family. For a more general and thorough introduction to this topic we refer the reader to two excellent textbooks that develop more generally applicable formalisms.^222,223^

#### 3.2.1 Local Electronic Structure: A Chemical Bonding Perspective

As is typical for dense periodic solids, the mobility of conduction electrons in vanadium oxides depends on the continuity of the filled and empty density of electron states (DOS) with energies spanning the Fermi level. In a typical strongly-electron-correlated binary or ternary vanadium oxide, these frontier states have substantial contributions from V 3*d* states, rendering their transport properties highly sensitive to the oxidation state of vanadium ions. In this review, we focus on materials with vanadium oxidation states ranging from 2+ to 5+ and electron configurations [Ar]­3*d* ^3^4s^0^ to [Ar]­3*d* ^0^4s^0^. The average vanadium oxidation state in a compound with the stoichiometry *M*_*x*_VO_*y*_ is given by 2*y-nx*, where *M* is a guest cation and *n* its formal positive charge. Selection of a guest ion and stoichiometric coefficients *x* and *y* thus allows direct control of frontier orbital filling.

Interaction of V 3*d* orbitals with a perfectly octahedral oxyanion crystal field splits the resulting antibonding orbitals into triply degenerate *t*_*2g*_ and doubly degenerate *e*_*g*_ manifolds (Figure 27a). Considering a simplified ionic bonding picture, these orbitals contain no O 2*p* character and they are conventionally named after the hydrogen-like 3*d* states they originate from *d*_*xy*_, *d*_*yz*_, and *d*_*xz*_, in the *t*_*2g*_ manifold and *d*_*x2–y2*_ and *d*_*z2*_ in the *e*_*g*_ manifold. We maintain this convention, although we shall see that V 3*d*–O 2*p* hybridization imparts significant O 2*p* character to these states (so that V–O bonds have substantial covalent character), which are further split by reduction of symmetry and partial filling.

**Figure 27:**
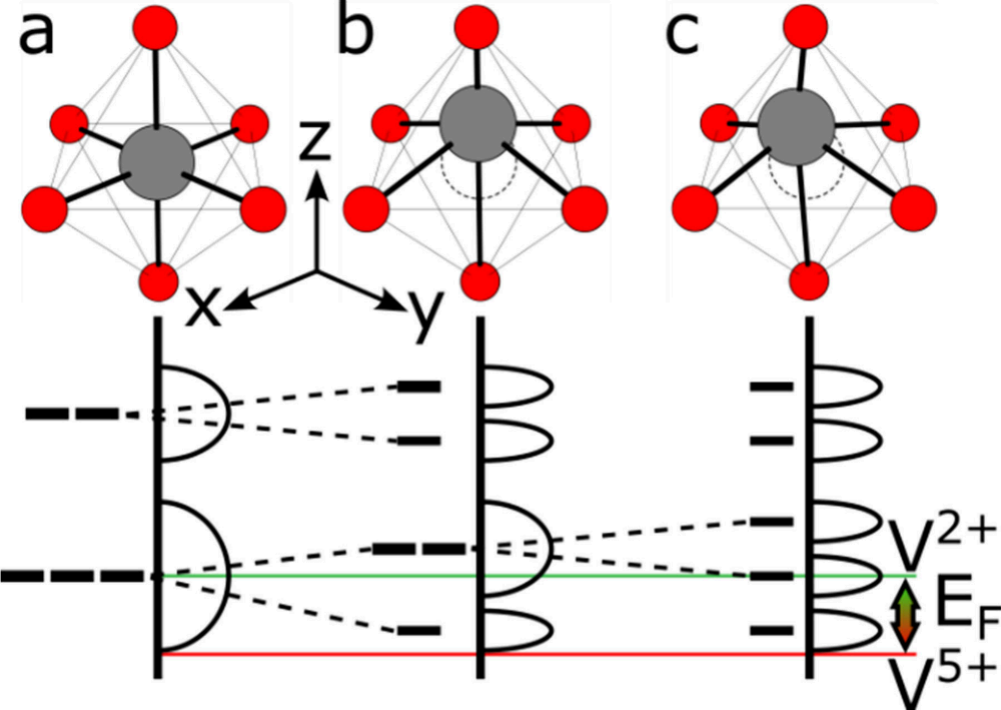
Crystal field splitting of atom-centered V 3d orbitals (left of energy axis) and bands (right) in (a) octahedral, (b) square pyramid, (c) equatorially distorted square pyramid coordination. Red and green lines respectively represent Fermi level for V^5+^ and V^2+^.

As discussed in preceding sections, the small size of V^5+^ (and occasionally V^4+^) ions frustrates perfect octahedral coordination by oxygen. Displacement of vanadium ions toward square pyramidal coordination lifts the degeneracy of the *t*_*2g*_ and *e*_*g*_ manifolds, respectively positioning the 3*d*_*xy*_ and 3*d*_*z2*_ orbitals at the lowest and highest energies (Figure 27b). In crystals where planar symmetry elements bisect VO_*x*_ basal planes diagonally, such as those featuring zigzag edge-shared chains, an additional equatorial displacement (along the *x* axis in Figure 27c) lifts the remaining degeneracy of the 3*d*_*yz*_ and 3*d*_*xz*_ orbitals. The sensitivity of vanadium’s 3*d* energy dispersion to its coordination environment strongly couples atomic and electronic degrees of freedom in oxides. For instance, the square pyramidal distortion in α-V_2_O_5_ stabilizes the *d*_*xy*_ state enough for it to split off from the rest of the conduction band to form a midgap state,^224–228^ rendering intercalated analogues susceptible to polaron formation.^229^

#### 3.2.2 V 3d Orbital Overlap and Structure Motifs

In addition to local effects, the development of extended electron states (bands) in vanadium oxides depends on the V–O bonding connectivity within the repeating structural motifs discussed in preceding sections. For simplicity we consider here states that arise from direct V 3*d*–3*d* interactions, with the caveat that, in reality, they are mediated by hybridization of O 2*p* states. The energy and dispersion relationships of these bands in a given material depend in detail on the connectivity of the vanadium oxide framework, as we will discuss below.

We will briefly consult a simplified tight-binding model, effectively a linear combination of electronic orbitals (LCAO) bonding picture, for a qualitative description of band formation. Following conventional derivations, we consider an infinite chain of evenly spaced hydrogen-like atoms bearing a single electron each, a sort of one-dimensional crystal. Bloch’s theorem stipulates that an electron in a periodic potential *V*(***r***) = *V*(***r*** + ***R***) (such as in a crystal where ***R*** is a lattice vector) must have a wave function of the form ψ = *e* ^*ik*^ ^•*r*^ *u*_*k*_ (***r***), where ***r*** and ***k*** are respectively the position vector and wavevector, and *u*_***k***_(***r***) = *u*_***k***_(***r*** + ***R***) shares the periodicity of *V*(***r***). From a set of atomic wave functions *φ*_*m*_ (***r***) of orbital character *m* and centered on a nucleus *j* separated from its neighbors by a translation vector ***T***, one can construct a basis of Bloch states with the form *φ*_*m*__, ***k***_ (***r***) = ∑_*j*_ *e* ^*i**k***^ ^·*j**T***^φ_m_ (***r*** – *j* ***T***). In general, electronic energy eigenfunctions in a crystal are linear combinations of these basis orbitals: ψ_***k***_ (***r***) = ∑_*m*_ *c*_*m*__, ***k***_ *φ*_*m*__, ***k***_ (***r***). For simplicity, we specify that the atoms in our chain are a uniform distance || ***T*** || = *a* apart, and that each bears a single *s*-like atomic orbital that interacts only with the nuclei of its immediate neighbors. We will also assume that direct overlap of the unperturbed atomic orbitals is negligible, ⟨*φ*_*m*_ (***r*** – *j* ***T***)|*φ*_*m*_ (***r*** – *l* ***T***)⟩ = *δ*_*j*__,*l*_. Despite its simplicity, this model captures the relevant aspects of band formation and serves as a starting point for describing the Hubbard and Peierls models below.

The Hamiltonian for this system contains two terms: *H* = *H_at_* + *V*_*nn*_, where *H_at_* contains the kinetic energy and “parent” nucleus potential energy terms of the isolated atom and *V*_*nn*_ contains nearest-neighbor nuclear potential terms. Application of this Hamiltonian to a Bloch basis state gives eqs 19–21:

19 $$\left\langle \varphi_{m,k}\left|H\right|\varphi_{m,k}\right\rangle =\overset{l+1}{\underset{j=l-1}{\sum }}\int d\tau e^{-ikja}\varphi_{m}^{*}\left(r-ja\right)\left[H_{at}+V_{nn}\right]e^{ikla}\varphi_{m}\left(r-la\right)$$

20 $$=E_{at}+E_{nn}+\left[e^{-ika}+e^{ika}\right]\int d\tau \varphi_{m}^{*}\left(r-a\right)V_{nn}\varphi_{m}\left(r\right)$$

21 $$=E_{0}-2\cos\left(ka\right)t\left(a\right)$$

where *E*_0_ = *E*_*at*_ + *E*_*nn*_ collects terms which do not vary with *k*. The “hopping integral” *t*(*a*) = −∫ *dτ φ*_m_^*^ (*x*)*V*_*nn*_ *φ*_*m*_ (*x* + *a*) determines the bandwidth, whose magnitude increases as the interatomic distance *a* decreases.

This model produces an effectively continuous band of energy eigenstates with a sinusoidal *E*(*k*) relationship and energy width 4*t* (Figure 28a), composed of bonding LCAOs at *k* = 0, nonbonding at *k* = π/2*a*, and antibonding at *k* = π/*a*. Band broadening due to strong overlap of *d*-orbitals on chains of vanadium atoms can thus close energy gaps induced by crystal field splitting or other effects, engendering collective electron behavior. In this highly simplified but instructive model, John Goodenough predicted that this localized-collective electron crossover occurs for V^*n*+^ ions below a critical V–V distance *R*_*c*_ = [3.17–0.05*n* – 0.04S­(*S* + 1)]­Å^230^ for total electron spin *S*, yielding 3.04, 3.02, 2.94, and 2.92 Å respectively for V^2+^, V^3+^, V^4+^, and V^5+^ in their low-spin states.

**Figure 28:**
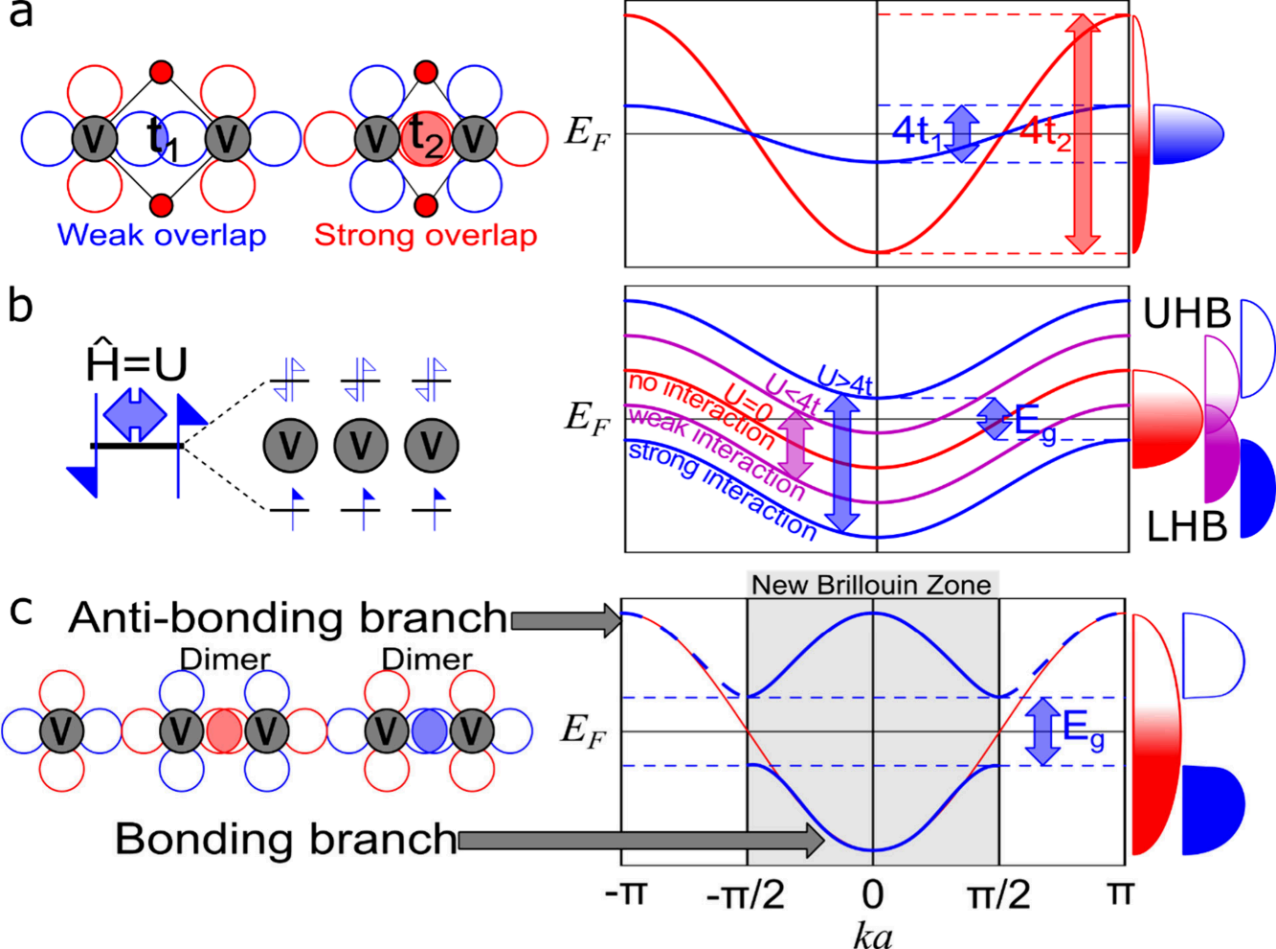
Band structure formation and MIT mechanisms in vanadium oxides. (a) Effect of direct V 3*d*–3*d* overlap on band dispersion. (b) On-site Coulombic repulsion U splits bands into upper Hubbard and lower Hubbard bands. (c) Peierls instability stabilizes dimerization of V chains, splitting bands into a filled branch with bonding character and an empty branch with antibonding character with respect to V–V pairs.

Octahedral corner- and face-sharing lead to values significantly above and below these values, respectively, and their degree of direct V3*d–*V3*d* overlap is relatively constant. Edge-sharing, however, tends to position vanadium ions at the cusp of Goodenough’s crossover distance so that the degree of V 3*d* de/localization along infinite edge-shared chains that span macroscopic crystalline domains is sensitive to relatively small, low-energy perturbations. Disruption of overlap by phonon coupling and thermal oscillations of vanadium ions, and in some cases lattice anharmonicity, contributes to the temperature sensitivity of electron states. Incorporation of ionic guest species introduces new mechanisms for thermal modulation of V 3*d* orbital overlap. Most known monovalent and divalent guest ions are significantly larger than vanadium ions in any oxidation state, necessitating significant distortions to, or the formation of interstices within, the host lattice. Electronic structure transitions in multiple materials are associated with order–disorder transitions of cations, accompanied inevitably by charge ordering along chains of edge-shared vanadium-centered polyhedra as discussed in further detail in Sections 3.4.1 and 3.4.2.

The structural anisotropy of extended edge-shared chains discussed in Section 1 is mirrored by a corresponding anisotropy in electronic structure. The structure of V 3*d*-derived bands in α-V_2_O_5_ serves as an illustrative example. Nominally empty V 3*d* states constitute the lower region of the material’s conduction band. As mentioned above, crystal field effects position the V 3*d*_*xy*_ orbital at the bottom of the V 3*d* manifold, perpendicular to the vanadyl bond. The orientation of the vanadyl bond along the layer stacking direction ensures that *xy* orbitals lie within the layer plane with lobes oriented toward those of their neighbors. The resulting σ bonding interactions create a split-off band separated from the rest of the conduction band by a pseudogap (visible at 2 eV above the valence band maximum in Figure 29a), whereas the antibonding states are elevated further into the conduction band. The presence of the split-off band is a consistent prediction of perturbative,^231^ density functional theory,^232,233^ and quasi-particle self-consistent GW calculations,^234^ and is well-attested by experimental evidence.^235–237^

**Figure 29:**
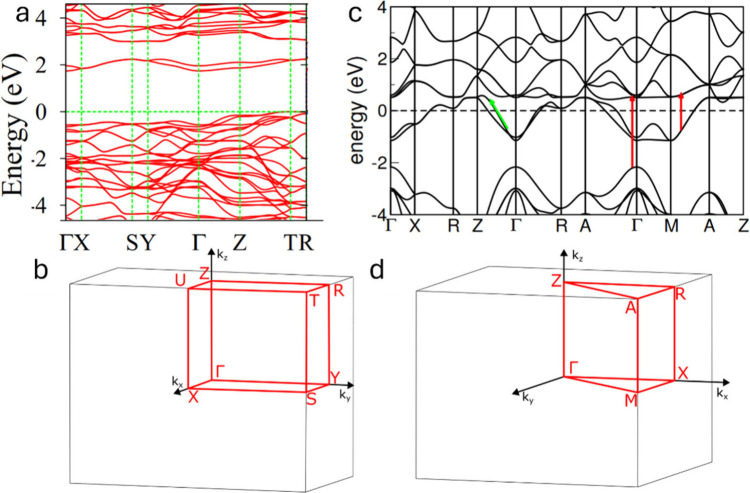
Band dispersion curves calculated for (a) α-V_2_O_5_ and (b) tetragonal rutile VO_2_. (c, d) Brillouin zone maps identifying K-points for (c) α-V_2_O_5_ and (d) VO_2_. Panel (a) was reproduced with permission from reference 234. Copyright 2015 American Physical Society. Panel (b) was reproduced with permission from ref 238. Copyright 2018 Optica Publishing Group.

The split-off band itself shows signatures of further dimensional confinement. In the calculated band structure of α-V_2_O_5_, V 3*d*_*xy*_ states (at 2 eV in Figure 29a) exhibit the greatest dispersion along the Γ–Y (along the zigzag chains, Figure 29b) and Γ–S (toward nearest neighbor) directions, suggesting that zigzag chains support metallic states only in the direction they extend.^234^ By contrast, the bands show minimal dispersion along Γ–X, indicating that the zigzag chains interact weakly with one another, an effect that arises from the antisymmetric nature of the bonding *d*_*xy*_ orbital combination with respect to the chain-bridging oxygen.^234^ A similar effect is visible in the high-temperature conductive phase of rutile VO_2_ (Figure 29c,d), where metallic states appear along the *Z* axis parallel to straight edge-shared chains.^238^ The split-off band in α-V_2_O_5_ has significant implications for the electronic structure of materials with a similar V_2_O_5_ framework, as we will discuss for the case of α-NaV_2_O_5_ below in Section 3.4.1. It has been shown to play a role in small polaron formation and stabilization of defects,^232,233,239^ which has substantial implications for the performance of α-V_2_O_5_ as a cathode material in intercalation batteries.^229^

#### 3.2.3 Electron–Electron Correlations: The Hubbard Model

So far our discussion has neglected the effects of Coulombic interactions between 3*d* electrons. The one-dimensional Hubbard model (Figure 28b) treats electron repulsion as an energy cost *U* incurred by situating two electrons on the same atom site, in our case the same V 3*d* orbital in an infinite chain.^240,241^ Such on-site Coulomb repulsion is particularly significant for 3*d* and 4*f* orbitals that have narrow spatial extent, resulting in strong localization and correspondingly high probability for intraorbital interaction when occupied by more than one electron. The effect of this repulsion on the electronic structure is greatest for half-filling of orbitals as in V^4+^, which we will assume going forward. As interacting electrons are not independent, their states can no longer be represented by single-particle wave functions and the Hubbard Hamiltonian must be written in second quantization notation as in eq 22:

22 $$H=-t\underset{\left(i=j\pm 1\right),\sigma }{\sum }c_{i,\sigma }^{†}c_{j,\sigma }+U\underset{i}{\sum }c_{i,↑}^{†}c_{i,↑}c_{i,↓}^{†}c_{i,↓}$$

where *t* is the familiar tight-binding overlap integral, *c*_*i*,σ_^†^ and *c*_*i*__,σ_ are creation and annihilation operators for an electron with spin state σ on site *i*, and *U* is the on-site electron interaction energy. The first term represents the tight-binding model discussed above, whereas the second term contributes *U* to the Hamiltonian for every pair of electrons in the same localized orbital. In the local view, this has the effect of reversing the energetic benefit of nearest-neighbor overlap and relocalizing electrons into singly filled atomic orbitals. In the reciprocal space view, repulsion splits the half-filled band into a filled lower Hubbard band (LHB) of unpaired electrons and empty upper Hubbard band (UHB) of paired electrons, shifted apart by an energy *U*. For *U* smaller than the Hubbard bandwidth 4*t′*, this separation creates a direct band gap of energy *U* (purple dispersion curve in Figure 28b) but no gap in the density of states. However, for *U* > 4*t′* (blue dispersion curve), an indirect band gap opens and the material exhibits insulating behavior. The band structure and transport behavior of so-called Mott insulators are thus sensitive to effects which modify *t* (via thermal motion, applied strain, local distortion, or *d*-orbital reorientation) or *U* (dielectric screening or charge carrier density). V 3*d* electron correlation effects contribute to the formation of insulating phases in many vanadium oxides that simple band theory—and even density functional theory approaches in underestimating bandgaps^242,243^ —predicts to be metals, such as VO_2_ and V_2_O_3_, as discussed below.

#### 3.2.4 Electron–Lattice Correlations: Peierls Transition

The one-dimensional chain of half-filled atomic orbitals we have considered thus far is susceptible to another instability: the Peierls transition. To illustrate, we recall our tight-binding chain of hydrogen-like atoms. Phase relationships between overlapping atomic orbitals in a tight-binding band are dictated by the magnitude of the plane wave electron’s wavevector ***k***. At *k* = 0 all phases are constant, so *s*-like atomic basis orbitals (or, for instance, V3*d*_*xy*_ orbitals oriented as portrayed in Figure 28c) will interact constructively and form bonds, whereas at *k* = ±π/*a* phases alternate at every atom to form nodes. At *k* = ±π/2*a*, the Fermi momentum of a half-filled band, the phases of the atomic basis orbitals alternate every other pair of atoms in the chain. The superposition | *k* = +π/2*a*⟩ + |*k* = −π/2*a*⟩ creates a standing wave which concentrates probability density between alternating pairs of atoms to effectively form new bonds. These states are stabilized by dimerization, which correspondingly destabilizes the empty, antibonding standing wave state formed by the opposite superposition |*k* = +π/2*a*⟩ – |*k* = −π/2*a*⟩, creating two new bands separated by an energy gap (*E*_*g*_ in Figure 28c). In the case a chain of hydrogen atoms, this dimerization collapses the chain completely into H_2_ molecules with electrons completely localized in H–H bonds. In real materials with strong bonding networks, partial dimerization instead results in a “Peierls distortion”, a doubling of the unit cell length and commensurate division of the Brillouin zone to position the gap at the new zone edge. The periodic localization of electron density in bonding states is referred to as a charge density wave (CDW), of interest for underpinning highly nonlinear dynamical switching of conductance.^244^

Rudolf Peierls proved that one-dimensional chains of atoms are necessarily unstable to such distortions.^244^ Peierls distortions in higher-dimensional systems require translation symmetry across significant regions of the Brillouin zone (“Fermi surface nesting”) and are relatively rare. However, several structure motifs in vanadium oxides sufficiently confine electronic states along a single direction of edge-shared polyhedra that their host materials undergo Peierls distortions and CDW formation. In the case of VO_2_, a pronounced dimerization is observed below 67 °C with alternating short and long bond lengths in monoclinic VO_2_ as compared to a single bond distance in the tetragonal rutile phase (*vide infra*). The metal–insulator transition in VO_2_ has contributions from both Peierls and Mott phenomena.^216^ As per mean field theory, the substantial superexchange between vanadium atoms in V–V dimers gives rise to dynamic intersite correlations. The Mott transition is thus adiabatically linked with the Peierls-like dimerization.

#### 3.2.5 Computational Approaches to Correlated Electronic Structure Prediction

The preceding sections are intended to familiarize the reader, in simple terms, with the origins and consequences of electron–electron and electron–lattice correlation effects which modulate the band structure of vanadium oxides. Modeling correlation effects to predict electronic structure with a useful degree of accuracy is substantially more difficult at the levels of both theory and implementation. As a complete treatment of computational methods is beyond the scope of this review, we intend the following descriptions to guide the reader toward the appropriate tool for their system of interest.

Density Functional Theory (DFT) is by far the most commonly used family of methods for computing band structures in extended solids. DFT approaches simplify many-body interacting systems by calculating observables (particularly the system’s energy) from a single functional of the many-body wave function, the electron density ρ­(***r***), which is decomposed into a basis of auxiliary, noninteracting Kohn–Sham orbitals. The Kohn–Sham DFT energy contributions consist of the kinetic energy, the Hartree energy (Coulombic repulsion), the exchange-correlation energy (*E*_XC_), and the external potential energy such as electron–nuclear Coulombic interaction energy. The exact form of the exchange-correlation energy functional is unknown for general interacting electron systems, and the accuracy and computational cost of a DFT calculation depend mainly on the complexity of the choice of approximated exchange-correlation energy functional, the system under study, the basis set, and the treatment of core electrons. The local density approximation (LDA) utilizes an *E*_XC_[ρ­(***r***)] which depends only the density as a function of position.^245^ Generalized gradient approximations (GGA) include the gradient of the density in *E*_XC_[ρ­(***r***),∇ρ­(***r***)], whereas meta-GGA incorporates the kinetic energy density τ­(***r***) in *E*_XC_[ρ­(***r***),∇ρ­(***r***),τ­(***r***)]. In addition, hybrid functionals with weighted sums of exact/screened Hartree–Fock exchange energy and other energy functionals often achieve better accuracy for various physical properties, although at increased computational cost.

By computing observables from the total electron density rather than individual single-electron wave functions, DFT drastically reduces the complexity of calculations on large systems. However, Coulomb repulsion calculated from the total electron density will erroneously include an individual electron’s interaction with its own density, which exchange-correlation functionals fail to cancel exactly.^245,246^ This “self-interaction error” is most severe for systems containing localized electrons and leads to DFT’s well-known tendency to delocalize electrons and underestimate band gaps in insulators. It is understandable that conventional DFT with LDA and GGA functionals fail to predict the electronic structure of strongly correlated systems. Due to use of exact Hartree–Fock exchange terms, hybrid functional approaches fare better but their computational cost renders them impractical for large systems. DFT+U methods account for on-site electron repulsion by incorporating the Hubbard U term discussed in Section 3.2.3 into the Kohn–Sham Hamiltonian, where the occupation number *n* at each atom site is computed by projecting the Kohn–Sham orbitals onto an atomic basis set.^247^ Actual values of *U* may be calculated at several levels of theory, but are often determined semiempirically to match predicted properties to experimental data.^248–250^

Green’s function (GF) methods are intermediate between wave function-based approaches and DFT in terms of both accuracy and computation cost.^251^ Like DFT, GF methods define a quasi-particle self-energy akin to *E*_*XC*_, but which is nonlocal (i.e., a function of two spatial coordinates) and frequency dependent. In many-body perturbation theory within the *GW* approximation,^252,253^ the self-energy can be approximated by the product of the dressed Green’s function *G* and screened Coulomb interaction *W*. In the simplest *G*_0_ *W*_0_ approximation, the dressed Green’s function *G* and the screened Coulomb interaction *W* are approximated by *G*_0_ and *W*_0_ directly calculated from eigenenergies, wave functions, and bare Coulomb interaction from a prior calculation, such as DFT. The quasi-particle self-consistent GW method (QSGW) allows for the iterative calculation and update of *G* and *W* in a self-consistent manner.^254^ One significant advantage of GW methods over DFT is their ability to treat excited state electronic structure, where the imaginary part of the self-energy naturally reflects the inverse of lifetime of excited states. In contrast, Kohn–Sham eigenvalues from DFT are auxiliary quantities which have no formal interpretation except that the highest occupied Kohn–Sham eigenvalues with the exact exchange-correlation functional is equal to the ionization potential. Although GW methods typically predict band gaps with higher fidelity than DFT, they struggle with materials which display strong excitonic effects as in the case of α-V_2_O_5_, the band gap of which even sophisticated QSGW methods tend to overestimate.^234,255,256^

Dynamical mean field theory (DMFT)^217,257^ is an electronic structure method for studying strongly correlated systems where electron–electron local interaction U is very strong, such as VO_2_ and V_2_O_3_. DMFT often starts from DFT eigenstates which are projected onto the relevant correlated space near the Fermi level and then transformed into a set of localized orbitals such as maximally localized Wannier functions^258^ that reproduce the low-energy electronic structure of DFT. Hubbard *U* and Hund’s exchange coupling *J* parameters can be determined for the correlated orbitals by using constrained random phase approximation,^259,260^ the linear response approach,^261^ or by fitting to experimental results. Local interaction *U* and *J* terms are added to the effective DFT Hamiltonian, resulting in a many-body lattice Hamiltonian. As solving this Hamiltonian is challenging for realistic materials, DMFT transforms the many-body lattice problem into a single quantum impurity problem for each correlated site embedded in a noninteracting electron bath from the remaining electrons in the crystal, which is then self-consistently solved. Compared to DFT and GW, which are often more suitable for weakly correlated materials, DMFT explicitly includes strong dynamic local correlations, making it particularly suited for studying systems with localized *d* or *f* electrons, such as Mott insulators.

#### 3.2.6 Electronic Instabilities in Single Crystals

Anisotropic electronic structures and transport phenomena are difficult to measure in powders. Electron transport through powder samples involves conduction through many particle–particle interfaces whose structure, composition, purity, and resistivity may differ significantly from the bulk material with substantial contributions observed from scattering at grain boundaries. The percolative nature of conductive pathways formed by particle contacts exacerbates the localization of current density and Joule heating, which reduces the volume of material modified by the probe bias and often establishes thermal gradients. As discussed at the end of Section 1, anisotropy in physical measurements is also hidden in powder samples by the random orientation of individual particles. Probe fields are applied along a random distribution of directions relative to crystallite orientation, and the measured response is aggregated similarly. Single crystals are thus ideal platforms for investigating the often-subtle mechanisms of electronic structure transformations in vanadium oxides. Modulating their transformation characteristics such as threshold voltage or temperature, thermal coefficient of resistivity, and hysteresis is critical for several applications, such as the design of electro-thermal neurons as we discuss in Section 4. In this section, we focus on studies that exploit the advantages offered by single crystal specimens. A broader perspective of metal–insulator transitions in general can be found elsewhere.^8,9,262–267^

### 3.3 Specific Examples: Binary Vanadium Oxides

#### 3.3.1 Temperature-Induced MIT behavior in vanadium oxide single crystals

Temperature-driven MITs occur in a variety of material classes, including transition metal oxides (TMOs), chalcogenides, and organic conductors, each governed by distinct underlying mechanisms.^268,21,269^ In this review, we focus on single-crystal vanadium oxides, which provide enhanced structural integrity, and a well-defined phase space. Five vanadium oxide material classes are discussed here (Figure 30a). The massive phase space occupied by strongly correlated oxide materials is reflected in Figure 30a, which provides an illustration of the MIT transition temperatures (*T*_C_) and the changes in resistivity of representative V–O crystals as compiled from an extensive search of the literature.^189,270–281^ Many crystals exhibit a *T*_C_ below 300 K, and fewer above the 300 K threshold imperative for applications in computing, sensing, and thermochromics.

**Figure 30:**
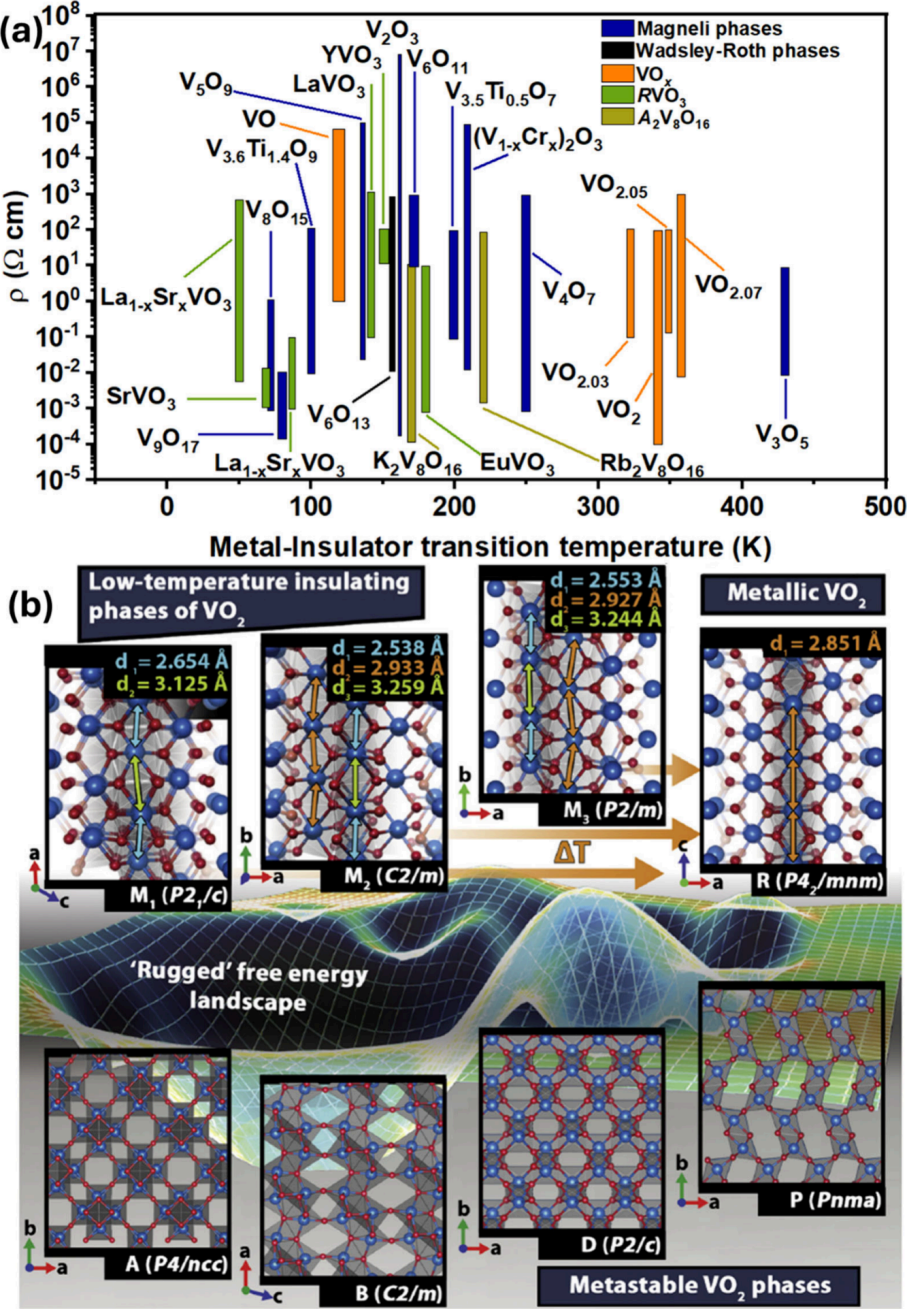
(a) Graphical representation of the MIT behavior compiled for several vanadium oxide-based crystals. The horizontal axis represents MIT temperatures (*T*_C_), whereas the vertical axes show reported changes in resistivity and conductivity. The height of each floating bar corresponds to the change in resistivity/conductivity between the metallic (bottom) and insulating (top) phases. (b) The structural relationships between the various reported polymorphs of VO_2_ as a model representation of MIT in binary vanadium oxide systems. Panel (b) was reproduced with permission from reference 8. Copyright 2019 Cell Press.

#### 3.3.2 VO_2_ as a Canonical Model of MITs in Binary Vanadium Oxides

VO_2_ serves as a model system for MIT materials, spanning the Mott–Peierls spectrum with distinct transition mechanisms and exhibiting widely varying transition temperatures (Figure 30a).^282^ Several different polymorphs of VO_2_ have been reported, thus defining a complex phase space.^283,10,284^ Among the known binary vanadium oxide MIT materials, correlated VO_2_ exhibits the most abrupt metal–insulator transition near room temperature (340 K). At *T*_C_ = 340 K, VO_2_ undergoes a diffusionless first-order phase transformation from a high-symmetry rutile-type phase (>340 K) to either a stable monoclinic (M_1_) phase or metastable M_2_/M_3_ phases (<340 K), as illustrated in Figure 30b. In the high-temperature rutile phase, vanadium atoms are evenly spaced with a V–V bond distance of 2.851 Å. Upon cooling, the system transitions into a low-temperature phase characterized by V–V dimers, where the M_1_ phase exhibits alternating long (3.125 Å) and short (2.654 Å) bonds, whereas the M_2_/M_3_ phases feature alternating dimerized and undimerized V–V chains (Figure 30b).^285^ In the M_1_ phase, all vanadium ions form a fully dimerized zigzag chain structure, giving rise to strong electron correlation, which induces strong Peierls distortion and opens a robust bandgap, resulting in an insulating ground state.^286^ The M_2_ phase consists of alternating chains: one made up of zigzag dimerized V–V pairs, and the other a linear, undimerized V–V chain. This combination introduces localized electronic states in the undimerized chains, emphasizing the role of electron correlation and giving the M_2_ phase a mixed Mott–Peierls character.^286^ The M_3_ phase consists of one nearly linear V–V chain and another that is slightly zigzagged, resulting in partial and irregular dimerization.^286,287^ This structural arrangement supports delocalized electronic states and facilitates spectral weight transfer that enhances Peierls interactions, thereby stabilizing a metallic ground state via a Peierls–Mott mechanism.^287^ These variations in V–V alignment are sensitive to anisotropic stresses, affect orbital overlap, bandgap size, and lattice dynamics, underpin spatially inhomogeneous electronic behavior (as a result of local strain gradients) and add to the complexity of the insulator–metal transition.

The interplay between the electronic Mott transition and the Peierls-type structural transformation remains a topic of ongoing debate in condensed matter physics. The modern discourse began with Morin et al., who used electrical resistivity and structural measurements to document a dramatic first-order MIT coincident with a rutile-to-monoclinic lattice change, thereby establishing VO_2_ as the prototypical MIT material, albeit without substantial commentary on the underlying mechanism.^153^ Subsequently, Goodenough proposed a molecular orbital picture based on the one-dimensional V–V correlations in rutile and monoclinic structures. He argued that in the high-temperature rutile phase, the V–V chains allow for metallic conductivity because of partially filled π* orbitals, whereas upon cooling, structural distortion (V–V pairing) in the monoclinic M_1_ phase results in Peierls-like bandgap opening via bonding–antibonding splitting of the d_∥_ orbitals. Thus, Goodenough’s local structure and bonding model attributed the insulating behavior of low-temperature VO_2_ to a Peierls distortion alone, without invoking strong electronic correlations.^288^ Zylbersztejn et al. challenged this view using temperature-dependent conductivity and Hall measurements, which concluded that the chemical bonding picture, as outlined by Goodenough, could not fully explain the transition, particularly the persistence of the insulating phase in the M_2_ structure (where half the V atoms are not dimerized). These authors posited strong on-site electron–electron interactions (Mott correlation) as being essential to stabilization of the insulating state.^289^ Wentzcovitch et al. subsequently performed density functional theory (DFT)-Local Density Approximation (LDA) band-structure calculations and showed that a ∼0.6 eV gap emerges from orbital overlap in dimerized vanadium chains, which is supportive of a Peierls-driven mechanism.^290^ This was countered by Pouget et al., who argued from symmetry and magnetic considerations that correlation effects must be included, even when structural calculations reproduce the bandgap.^291^ Huang et al., using LDA+U simulations, concluded that both the lattice distortion and on-site Coulomb repulsion were needed to reproduce the insulating gap, advocating a hybrid Peierls–Mott framework.^292^ Biermann et al. applied cluster-dynamical mean field theory (DMFT) to a V–V dimer model and found that the M_1_ phase is a correlated singlet insulator consistent with a Mott-driven gap, whereas the M_2_ phase behaves like a classical Mott insulator with undimerized chains, introducing the concept of a correlation-assisted Peierls transition.^293^ Tomczak and Biermann extended this framework with spectroscopic measurements and concluded that both lattice distortion and strong correlations are required to describe VO_2_’s excitation spectrum accurately.^294^ Weber et al., using orbital-resolved cluster DMFT, found that the a_1g_ orbital undergoes an orbital-selective Mott transition, with Peierls distortions modulating, rather than driving, the MIT.^295^ In parallel, Kim et al. used DFT+U phonon-dispersion calculations and demonstrated that lattice instabilities only occur when Coulomb interactions are included, suggesting that Peierls distortion is assisted by electron correlations, not independent.^296^ Later, Budai et al. combined inelastic neutron and X-ray scattering with anharmonic phonon calculations and showed that vibrational entropy, especially from soft phonons, accounts for nearly two-thirds of the entropy change across the transition, which points to lattice dynamics being critical in stabilizing the phase change thermodynamically but perhaps not being the initiating mechanism.^297^ Huffman et al. carried out broadband optical spectroscopy on M_1_, M_2_, and triclinic phases and found virtually identical ∼0.6 eV optical gaps, despite differing lattice structures, thus emphasizing that the gap is primarily controlled by intra-atomic Coulomb interactions (i.e., Mott correlation).^298^ Brito et al. and Nájera et al. recently argued based on charge self-consistent DFT + DMFT calculations for the rutile, M_1_, and M_2_ phases that the insulating gap in the M_1_ phase originates from intersite singlet formation, a Mott-like mechanism, whereas M_2_ behaves as a classical Mott insulator, which points to a continuum of correlation-driven behavior with cooperative structural coupling.^216,299^

In recent experimental studies, Kumar et al., using optical transmission, resistivity, and X-ray spectroscopy on VO_2_ thin films, observed that electronic switching precedes the structural transition, which they used to posit the primacy of correlations in driving the transition.^300^ D’Elia et al. using temperature-dependent X-ray Absorption near Edge Structure (XANES) and Resonant Photoemission Spectroscopy (ResPES) on tensile-strained VO_2_ films and demonstrated that the transition can still occur when the structural distortion is suppressed. This led them to conclude that Mott correlations alone are sufficient to induce the MIT.^301^ Similarly, Sahu et al. combining time-resolved resistivity and Raman spectroscopy during rapid thermal cycling reported that changes in electrical resistance occurred prior to any observable Raman-active structural changes. They argued that this temporal decoupling indicates that the MIT is electronically driven.^302^ Rischau et al. took a different approach, using Raman and inelastic X-ray scattering with isotope substitution, and discovered phonon anomalies and shifts in the transition temperature. While they acknowledged the role of lattice dynamics, they suggested that these effects modulate but do not initiate the MIT, pointing instead to a correlation-assisted lattice mechanism.^303^ Díaz et al. explored the dynamics of the transition through nonequilibrium cluster DMFT simulations of a dimer-Hubbard model under both thermal and electric field excitation. Their findings indicated that electronic rearrangements precede structural changes across all conditions studied, reinforcing the hypothesis of a Mott-first transition mechanism.^304^ Most recently, Mlkvik et al. developed a bond-centered orbital single-site DFT+DMFT framework that permits continuous interpolation between the rutile and M_1_ phases. Their results showed that structural dimerization and electronic correlations evolve in a cooperative and synergistic fashion, supporting a unified Peierls–Mott transition picture.^305^ Taken together, this extensive body of experimental and theoretical work illustrates the considerable complexity: the MIT in VO_2_ appears to be fundamentally driven by strong electron correlations (Mott physics), whereas Peierls-type lattice distortions and phonon dynamics act in concert to stabilize the insulating state and shape the thermodynamics of the transition.

#### 3.3.3 Temperature Driven Nonlinear Dynamical Transitions in Other Binary Vanadium Oxides

Beyond stoichiometric VO_2_, the oxygen-deficient Magnéli phases (V_*n*_O_2*n*–1_) and Wadsley phases (V_*n*_O_2*n*+1_) discussed in Section 1.2.1 and sketched in Figure 2b,c show one-dimensional chains of edge-shared VO_6_ octahedra that can underpin electronic instabilities..^189^ These two families of compounds exhibit distinct MIT behaviors in their single-crystal forms. For example, the Magnéli phases, with corundum- and rutile-like layers as shown in Figure 5a, exhibit thermally induced MITs at temperatures ranging from 70 K (V_8_O_15_) to 430 K (V_3_O_5_), often accompanied by anomalies in magnetic susceptibility.^270,271^ In contrast, of the Wadsley–Roth phases, only V_6_O_13_ undergoes an MIT at 150 K, characterized by a sharp conductivity change and structural reorganization within the vanadium oxide layers due to mixed V^4+^/V^5+^ valence states.^272^

### 3.4 Modulating Metal–Insulator Transformation Characteristics in Binary and Ternary Vanadium Oxides

Multiple attributes of metal–insulator transformations must be considered when integrating MIT materials into electronic circuits, such as threshold temperature or voltage, conductance differential, sharpness of onset, and hysteresis. These characteristics couple directly into eqs 23 and 24 (see Section 4 below) governing memristive behavior and edge-of-chaos required for the construction of electrothermal neurons. Building electronic circuits to emulate neurons and synapses requires careful modulation of state variables in a manner that expresses the right kind of nonlinear control over electrical conductance. The MITs of binary and ternary vanadium oxides are capable of systematic modulation through modification of structure, composition, and application of external fields that modify different state variables such as effective temperature. The vast structural and compositional diversity of these compounds further holds promise for additional state variables beyond temperature to modulate spiking or oscillatory behavior. For instance, in ternary, quaternary, and quinary vanadium oxide bronzes that have been recently reported, independent modulation of ion concentrations can serve as state variables akin to Na- and K-ion channels used in biological neurons.^25,306^ Figure 31a schematically illustrates several approaches for modifying MIT characteristics encompassing site-selective modification, defect engineering such as through irradiation exposure, strain engineering, size variation, and applied voltage and pressure. We begin by discussing the impact of doping/alloying in binary vanadium oxides and then review the particular cases of VO_2_, V_2_O_3_, V_4_O_7_, and V_5_O_9_ single crystals. We then explore the role of stoichiometric variation, strain engineering, irradiation exposure and the case of size variation in modulating transformation characteristics.

**Figure 31:**
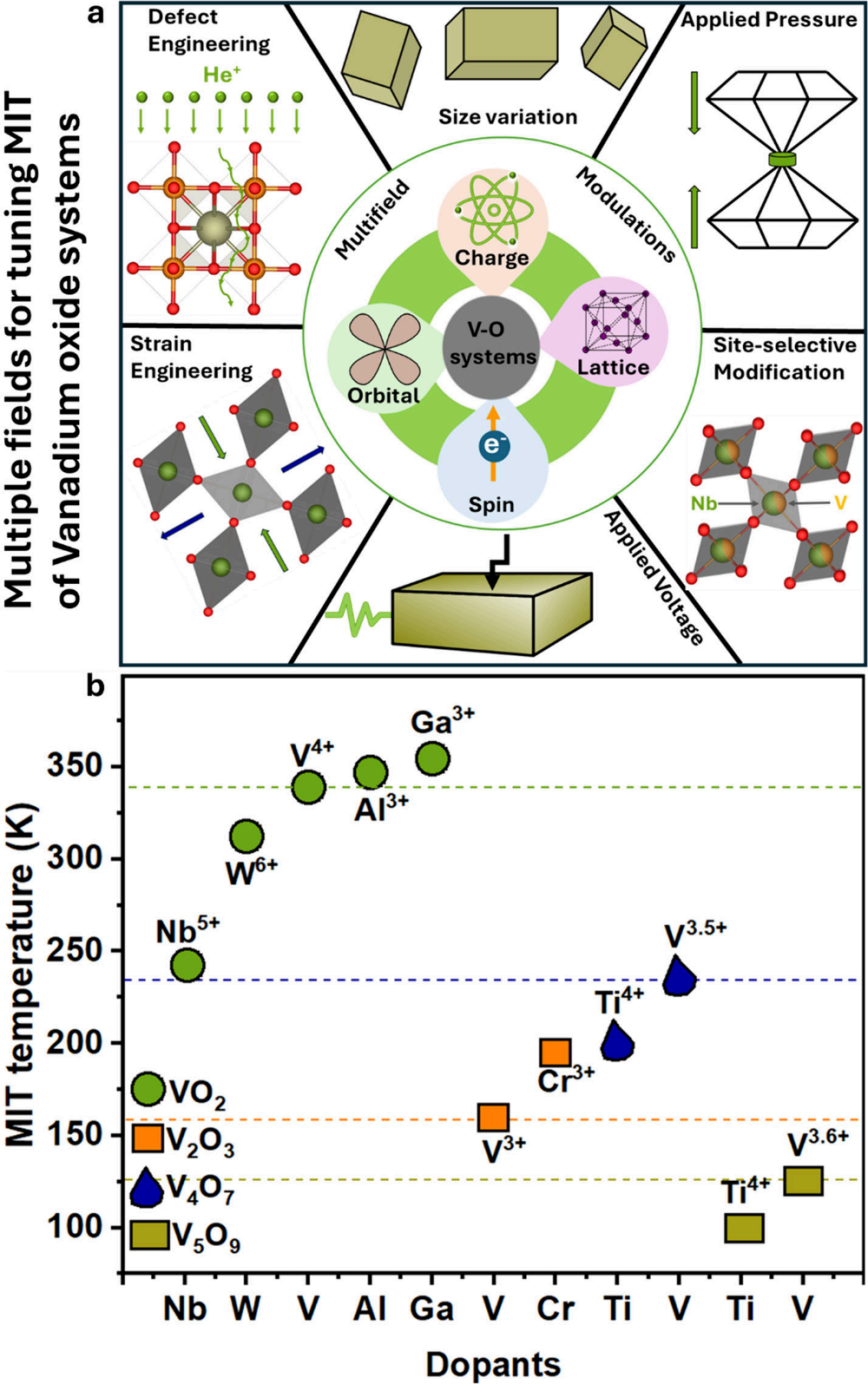
(a) Schematic overview of modulating the metal–insulator transition (MIT) in vanadium oxide systems through various external fields. (b) Effect of dopant ion charge and size on the MIT temperatures in V–O systems in this review. Dopant ions larger and higher-valent than V^4+^ (VO_2_), V^3.5+^ (V_4_O_7_), and V^3.6+^ (V_5_O_9_) typically cause decreased MIT temperatures, whereas smaller, lower, and equivalent dopants tend to increase MIT temperatures. The horizontal dashed lines represent the MIT temperatures of unalloyed VO_*x*_ crystals.

#### 3.4.1 Site-Selective Modification

Considering that the MIT *T*_C_ for most unalloyed binary vanadium oxides is below 300 K, considerable attention has focused on incorporation of transition metals on the vanadium sublattice to modulate the *T*_C_. The incorporation of dopants modifies the “rugged” free-energy landscape of vanadium oxides (Figure 30b) by altering relative phase stabilities and transformation barriers.^8^ This in turn intricately modifies the coupling of charge, lattice, orbital, and spin degrees of freedom (Figure 31a).^8^ Aliovalent substitution has the effect of introducing charge-compensating vacancies or interstitials and altering the effective concentration of charge carriers. Small substitutional dopants with high-valent transition metals (e.g., Ti^4+^, Nb^5+^, W^6+^, and Mo^6+^) result in electron carrier donation to the low-energy *t_2g_* orbital of vanadium oxides, reducing its *T*_C_. For instance, doping V_4_O_7_ (V^3.5+^) and V_5_O_9_ (V^3.6+^) with Ti^4+^ and VO_2_ (V^4+^) with Nb^5+^ and W^6+^ decreases the *T*_C_ (Figure 31b).^278,307,308^ In contrast, doping with low-valent and homovalent cations (e.g., Cr^3+^, Ga^3+^, and Al^3+^) tends to stabilize metastable insulating phases of vanadium oxides, which elevates the resulting *T*_C_. For example, Cr^3+^ doping in V_2_O_3_ (V^3+^) single crystals elevates *T*_C_ by nearly 35 °C (Figure 31b),^309,279^ whereas Al^3+^ and Ga^3+^ doping in VO_2_ (V^4+^) significantly elevates the *T*_C_ to 348 and 355 K from 340 K, respectively.^310,311^ A comprehensive account of dopant effects on the MIT of VO_2_ can be found in a recent review by Schofield et al. that explores the distinctive effects of individual dopants on transformation characteristics based on modulation of local structure (V–V dimer bonds lengths), electronic structure, concomitant defects, and local stress gradients.^9^

#### 3.4.2 Defect Engineering

Irradiation is a powerful tool for manipulating the metal–insulator transition (MIT) in correlated oxides by introducing structural disorder, carrier localization, lattice distortions, and modifying carrier and defect densities.^281,312–314^ Helium ion (He^+^) irradiation, in particular, has been shown to induce and modulate MIT temperature and drive an insulating phase as exemplified by Wang et al. in epitaxial SrVO_3_ films. The as-deposited thin film shows metallic behavior, but as irradiation fluence increases, resistivity rises, leading to an MIT at ∼40 K (1.75 × 10^15^ He/cm^2^) and ∼68 K (2.5 × 10^15^ He/cm^2^). At ≥3 × 10^15^ He/cm^2^, the films become fully insulating.^315^ This behavior is likely due to He^+^-induced lattice expansion and creation of vacancy and interstitial defects. Lattice expansion disrupts V–O hybridization, which reduces bandwidth and increases electron correlation strength. In contrast, ion collisions introduce point defects and scattering centers, further localizing charge carriers.^316^ As fluence increases, cumulative disorder further degrades metallic pathways, requiring higher thermal energy to induce conduction, thereby shifting the MIT to higher temperatures. Eventually, at high fluences, free carriers are trapped completely, yielding a fully insulating state.

#### 3.4.3 Stoichiometric Variation

Oxygen nonstoichiometry can effectively serve as an electron acceptor, depleting conduction electrons and promoting the stabilization of higher vanadium oxidation states.^317,318^ This increases electron localization and strengthens V–O hybridization, which favors the insulating state over the metallic state, thereby shifting the MIT to a higher temperature.^317,318^ For instance, the electrical conductance of VO_2_ single crystals varies significantly with oxygen content. Stoichiometric VO_2_ (VO_2.00_) exhibits a resistivity jump of ∼10^7^ at 63–64 °C with significant hysteresis; the hysteresis reflects challenges with nucleating the low-symmetry monoclinic phase in the tetragonal lattice.^283,319^ Increasing oxygen content progressively raises the *T*_C_ and widens the resistivity gap, with VO_2.03_ transitioning at 62–63 °C, VO_2.05_ at 65–66 °C, and VO_2.07_ at 71–72 °C.^320,280^ More nonstoichiometric samples exhibit reduced hysteresis and an intermediate monoclinic-tetragonal phase, attributed to V^5+^ ions inducing lattice distortions. These distortions modify orbital occupancy and reduce the density of itinerant carriers, further stabilizing the insulating phase.

#### 3.4.4 Thin Film Thickness

Thin film thickness plays a crucial role in manipulating MIT by altering quantum confinement, strain, defect density, and electron correlation effects.^264,321^ For instance, considering epitaxial SrVO_3_ thin films, Gu et al. demonstrated dimensional-crossover-driven MITs in high-quality epitaxial SrVO_3_ thin films grown by pulsed electron-beam deposition. Their findings reveal that thick SrVO_3_ films (∼25 nm) exhibit metallic behavior, whereas a temperature-driven MIT is induced in SrVO_3_ ultrathin films with thicknesses below 6.5 nm. The transition temperature *T*_C_ varies with thickness, 50 K for a 6.5 nm film, 120 K for a 5.7 nm film, and 205 K for a 3 nm film. The emergence of the MIT induced in SrVO_3_ ultrathin films with thicknesses below 6.5 nm is attributed to a dimensional crossover from a three-dimensional metal to a two-dimensional Mott insulator.^322^

#### 3.4.5 Strain and Pressure Effects on MITs

Considering the coupling among the lattice, orbital and charge degrees of freedom within correlated vanadium oxide materials, strain engineering has been utilized as an effective strategy to regulate the MIT functionality of V_2_O_3_ and VO_2_ by manipulating the band structure and orbital occupancy.^323,324^ Applying uniaxial compressive stress along the *c* axis alters the V–V bond lengths, which can influence the *T*_C_ by disrupting the V–V dimerization.^323,325^ For instance, epitaxial strain has been used to either induce or suppress the metallic state.^326,327^ For instance, Yonezawa et al. achieved controlled-MIT by growing V_2_O_3_ films on α-Al_2_O_3_ (0001) (+3.9% biaxial strain), which stabilizes the metallic phase down to 2 K from 170 K, whereas growth on LaTaO_3_ (0001) (−4.0% biaxial strain) raises the *T*_C_ to 190 K.^328^ Similarly, Gu et al. have shown that in VO_2_ thin films, compression of the *c* axis when grown on TiO_2_ (001) (+0.86% biaxial strain) shifts the transition temperature down to ∼300 K, which makes this system attractive for room-temperature applications.^325,327^

Beyond VO_2_, Kageyama et al. reported the growth of single-crystal EuVO_3_ thin films on (001)-oriented SrTiO_3_ substrates using pulsed laser deposition (PLD) and MBE with transitions observed at about 180 and 140 K.^329^ These transitions are attributed to changes in orbital ordering, as discussed below; however, the transition temperatures are slightly lower than that of bulk EuVO_3_ (∼200 K), likely due to the biaxial strain from the STO substrate convoluted with the presence of oxygen vacancies in the samples.^329^ An intriguing question from the perspective of electrothermal neurons and more broadly for neuromorphic computing is whether strain can be used as a mechanical state variable. Strain can provide positive and negative feedback and can couple to thermally induced stress and thus satisfies conditions for manifesting EOC. Systematically modulating the strain state of a device and obtaining a feedback mechanism based on applied voltage or current represents an active area of research.^330^ One can envision the application of a “bias strain”, like an ambient temperature, to place a device at a desired steady state, which is then perturbed through electrothermal mechanisms or ion transport to obtain semistability and feedback-responsible behavior emulative of biological neurons. Section 7.4 provides a detailed discussion of the use of single crystals to interrogate mechanical properties and examine chemo-mechanical coupling.

Considering a sign inversion, the application of external pressure can drive and modulate MIT in vanadium oxide materials. Pressure serves as an effective tool for modifying the electronic structures of materials and accessing different regimes in the rugged free energy landscape. Generally, exerting pressure alters the crystal structure, resulting in Fermi surface shifts and changes in carrier concentration.^331–333^ In strongly correlated electron systems, high pressure is particularly valuable, as lattice compression enhances orbital overlap and expands the electronic bandwidth, offering a direct means to explore the interplay between electronic interactions and material properties.^334^ Jayaraman and McWhan et al. demonstrated that substituting V with Cr in V_2_O_3_ can control the MIT of V_2_O_3_. For instance, Cr-doped V_2_O_3_ exhibits a pressure-induced MIT, where the critical pressure varies with Cr concentration. For *x* = 0.0375, the MIT occurs at 12.5 kbar (390 K). With decreasing Cr stoichiometry, the pressure at which the MIT occurs is reduced. For example, at *x* = 0.0187 and 0.0135, the transition shifts to 5.5 kbar (433 K) and 3.5 kbar (443 K), respectively. A linear extrapolation to unalloyed V_2_O_3_ suggests a critical pressure of −1.5 kbar (474 K), indicating a thermally driven transition at high temperatures.^309,279^

Similarly, Ueda et al. have shown that single crystals of V_7_O_13_ and V_8_O_15_ undergo pressure-induced MITs, accompanied by the suppression of their antiferromagnetic (AFM) phases under high pressure. In V_7_O_13_, resistivity measurements reveal a low-temperature anomaly at ambient pressure, corresponding to antiferromagnetic ordering at *T*_N_ ≈ 43 K. As pressure increases, *T*_N_ systematically decreases, and above 3 GPa, the anomaly broadens significantly. At 3.45 GPa, it vanishes entirely, marking the antiferromagnetic quantum critical point (QCP) and representing the complete suppression of long-range magnetic order.^335^

In V_8_O_15_, a first-order MIT occurs at *T*_C_ ≈ 69 K, followed by a transition into an AFM state at *T*_N_ ≈ 38 K. Under pressure, *T*_C_ abruptly disappears at 1.3 GPa, and the material remains metallic down to 0 K, confirming a first-order pressure-induced MIT.^335^ A resistivity anomaly, previously reported, suggests the persistence of AFM ordering in the pressure-induced metallic phase. Similar to V_7_O_13_, the AFM transition in V_8_O_15_ shifts to lower temperatures with increasing pressure and disappears entirely above 3.3 GPa, confirming the presence of an AFM QCP.

#### 3.4.6 Voltage-Induced MITs in Vanadium Oxide Single Crystals

As sketched in Figure 42 in Section 4.1 below, voltage- or current-controlled electrothermal neurons exhibit Joule self-heating, which modulates a state variable, the internal temperature, when current flows through active elements. Beyond this Joule heating picture, the role of the application of an external voltage or electric field in modulating the MIT by modifying charge distribution, carrier dynamics, and electronic correlation strength has further been examined for vanadium oxide single crystals.^336^ Electrical transport measurements reveal that V_3_O_5_ single crystals exhibit nonlinear *I*–*V* characteristics, where the system initially follows an insulating state before transitioning into a negative differential resistance (NDR) regime at higher voltages.^337^ Such NDR behavior is a telltale experimental signature of EOC and represents a system capable of oscillating (if the rates of heating and cooling are properly balanced either for the intrinsic element alone or with external resistive and capacitive circuit elements. This transition ultimately stabilizes in a metallic phase, indicating the onset of an electrically driven MIT. The resistive switching mechanism is influenced by self-heating effects, leading to variations in transition dynamics between different sample forms. Single-crystal V_3_O_5_ displays faster and more reproducible switching, whereas polycrystalline samples show slower transitions and extensive memory effects, suggesting a dependence on structural homogeneity.^337^

At higher currents, the oxygen stoichiometry of V_3_O_5_ is modified, leading to modulations of the MIT characteristics. These variations highlight the interplay between thermal and electrical effects in influencing the material’s transition dynamics and demonstrates the promise of using oxygen stoichiometry or defect dynamics in general as a means of synaptic emulation (wherein the oxygen vacancy concentration toggles the system between two distinctive neuronal regimes). The ability to induce and control an MIT in V_3_O_5_ through applied voltage presents promising opportunities for resistive switching devices and neuromorphic computing applications, where materials with high transition temperatures, EOC, and capable of sustaining prolonged periodic oscillations are in demand.

As another example, Jiang et al. observed a voltage-induced MIT in V_2_O_3_ single crystals characterized by an abrupt change in current at 160 K under an applied voltage of ∼2 V. The voltage-induced resistance change observed is triggered by Joule heating.^338^ Furthermore, Chudnovskii et al. studied the effect of Cr doping on the voltage-induced MIT behavior of V_2_O_3_ and observed that the Cr-doped V_2_O_3_ ((V_1–*x*_Cr_*x*_)_2_O_3_, *x* = 0.012) single crystals exhibit voltage-driven MIT behavior with both S-type and Z-type negative differential resistance (NDR) effects.^339^ The *I*–*V* characteristics reveal multiple switching states, where an applied voltage first induces a transition from a high-resistance state to a low-resistance state at a threshold voltage. Further increasing the current leads to a second transition state, corresponding to the paramagnetic insulator (PI) phase. The transition thresholds are temperature-dependent, aligning with the AFI–PM–PI phase transitions of Cr-doped V_2_O_3_. Unlike thermally driven transitions, which often cause material degradation, the voltage-induced MIT remains stable under repeated cycling, likely due to localized filamentary conduction rather than bulk heating. This suggests that Cr-doped V_2_O_3_, like V_3_O_5_, is a promising material for switching devices and resistive memory applications, offering tunable electronic properties through electric field control and with the promise of modulating spin states in addition to temperature as a state variable.

### 3.5 Case Studies of MIT Mechanisms in Multinary Vanadium Oxides

Metal–insulator transitions in binary vanadium oxides arise from V 3*d* states populated by incomplete oxidation of vanadium by oxygen, so that large changes in band filling necessitate changes in VO connectivity. The *M*_*x*_V_2_O_5_ ternary vanadium oxide bronzes, named after the bronze color of analogous ternary tungsten oxides, provide a platform for altering *d*-orbital populations independently of overall material structure. The chemical and structural properties which allow the incorporation of guest ions are discussed in Section 5 below. Here, we will discuss several electronic structure transformations in vanadium oxide bronzes in terms of the common structural motifs which support them.

#### 3.5.1 Competing Correlated States in α-NaV_2_O_5_

α-NaV_2_O_5_ stands as an excellent example of structural anisotropy giving rise to complex electronic states. α-NaV_2_O_5_ shares its vanadium oxide framework with the thermodynamic polymorph α-V_2_O_5_, consisting of edge-shared chains of VO_5_ octahedra square pyramids linked by corner-sharing to form sheets as described in Section 1 and illustrated in Figure 32a. From the stoichiometry, an average V^4.5+^ oxidation state and quarter-filled V 3*d*_*xy*_ band predicts metallic conductivity; however, single crystals of α-NaV_2_O_5_ exhibit a semiconducting resistance–temperature relationship above *ca*. 40 K, which fits well with a one-dimensional variable-range hopping model.^340^ From 40 to 35 K, resistivity decreases rapidly before recovering its semiconducting behavior below ca. 30 K.^340^ This is accompanied by a near-complete loss of magnetic susceptibility at 34 K, visible in Figure 32b.^341^

**Figure 32:**
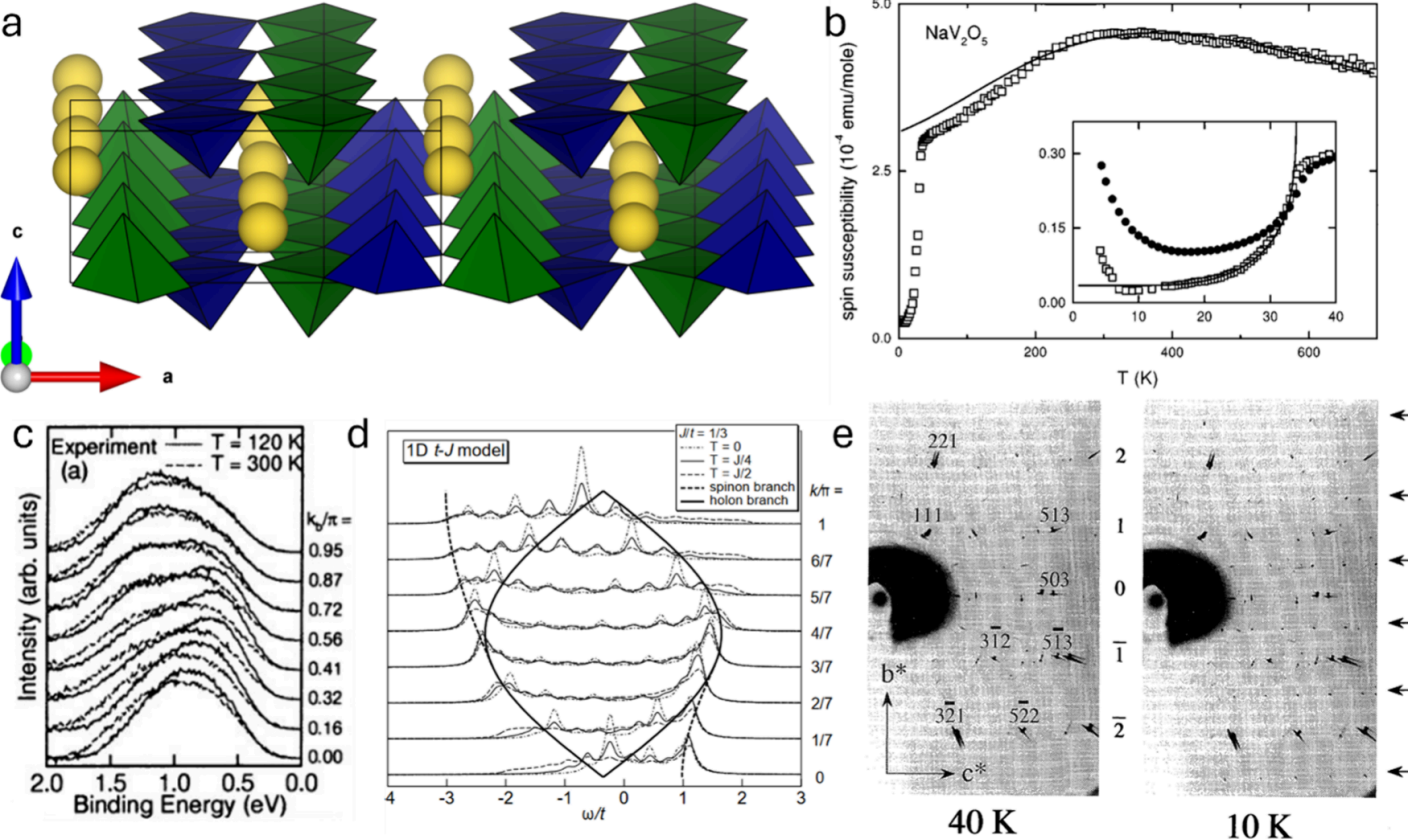
Crystal structure and electronic structure measurements of α-NaV_2_O_5_. (a) Crystal structure of α-NaV_2_O_5_, with crystallographically independent VO_5_ pyramids in green and blue and sodium ions in yellow. (b) Magnetic susceptibility vs temperature of α-NaV_2_O_5_ single crystals (open squares) and powder (filled circles) and Bonner–Fisher model fit (solid line). (c, d) Measured and simulated ARPES spectra of V 3*d* bands showing asymmetric dispersion about ***k*_b_** = π/2, matching asymmetry of spinon branch, dashed line in panel (d). (e) Single-crystal diffraction frames with rows of ***q*** = [1/2,1/2,1/4] satellites indicated by arrows. Panel (b) was reproduced with permission from ref 340. Panels (c) and (d) were reproduced with permission from reference 344. Copyright 1999 by the American Physical Society. Panel (e) was reproduced with permission from reference 346. Copyright 1997 Journal of the Physical Society of Japan.

The nature of the electronic phases, and particularly of the apparent half-filled behavior of the high-temperature insulating state, has been the subject of some controversy. Initial magnetic measurements^342^ revealed a broad susceptibility maximum around a Néel temperature of 320 K, suggesting the existence of (half-filled) one-dimensional Heisenberg chains in the high-temperature phase.^341^ Early single-crystal diffraction studies^343^ reported two inequivalent vanadium sites, consistent with charge disproportionation into separate half-filled V^4+^ 3*d* ^1^ and empty V^5+^ 3*d* ^0^ systems, colored green and yellow in Figure 32a, respectively. Based on this interpretation, α-NaV_2_O_5_’s high-temperature semiconducting behavior arises from Hubbard-like correlation effects within the half-filled 3*d*_*xy*_ chain. ARPES spectra (Figure 32c,d)^344^ show V 3*d* states forming a pair of features dispersive only along the *b* crystallographic direction in the 0–2 eV binding energy range, interpreted as spinon and holon excitations indicative of one-dimensional Hubbard band formation.^345^

Isobe and Ueda identified the sharp loss of magnetic susceptibility at 34 K as a spin-Peierls (SP) transition, whereby a collapse of antiferromagnetic ordering into localized spin-singlet pairs dimerizes the vanadium oxide lattice akin to the (conventional) Peierls transition.^341^ The appearance of ***q*** = [1/2,1/2,1/4] satellite reflections in single-crystal diffraction at low temperature, depicted in Figure 32e, was interpreted as additional evidence of a SP transition.^346^

More recent high-quality structure refinement of single-crystal X-ray diffraction data, however, revealed that all vanadium sites in α-NaV_2_O_5_ are equivalent, with an average V^4.5+^ valence.^347^ This finding suggested splitting of the V3*d*_*xy*_ states into (split-off) bonding and antibonding bands via strong interactions between zigzag chains (*t*_⊥_ in Figure 33a) as the source of the half-filling necessary for Hubbard band formation. This conclusion is supported by recent band structure calculations, as discussed for α-V_2_O_5_ in Section 3.2.2 above.^348^ This result also threw into doubt the spin-Peierls interpretation of the 34 K transition,^349^ and subsequent work has focused on determining the nature of the state below 34 K. Angle-resolved ^51^V and ^23^Na nuclear magnetic resonance^350,351^ and far-IR^352^ spectroscopy studies confirm the uniform V^4.5+^ valence at high temperature and strongly implicate zigzag V^4+^/V^5+^ charge ordering as the source of the supercell evidenced in Figure 32e, although several charge ordering patterns are possible. Single-crystal X-ray anomalous scattering experiments highlighted in Figure 33b^353^ support the pattern shown in Figure 33c, although this is incompatible with the previously established *Fmm2* space group in the low-temperature state, which necessitates an intermediate V^4.5+^ valence on alternating ladders (Figure 33d).

**Figure 33:**
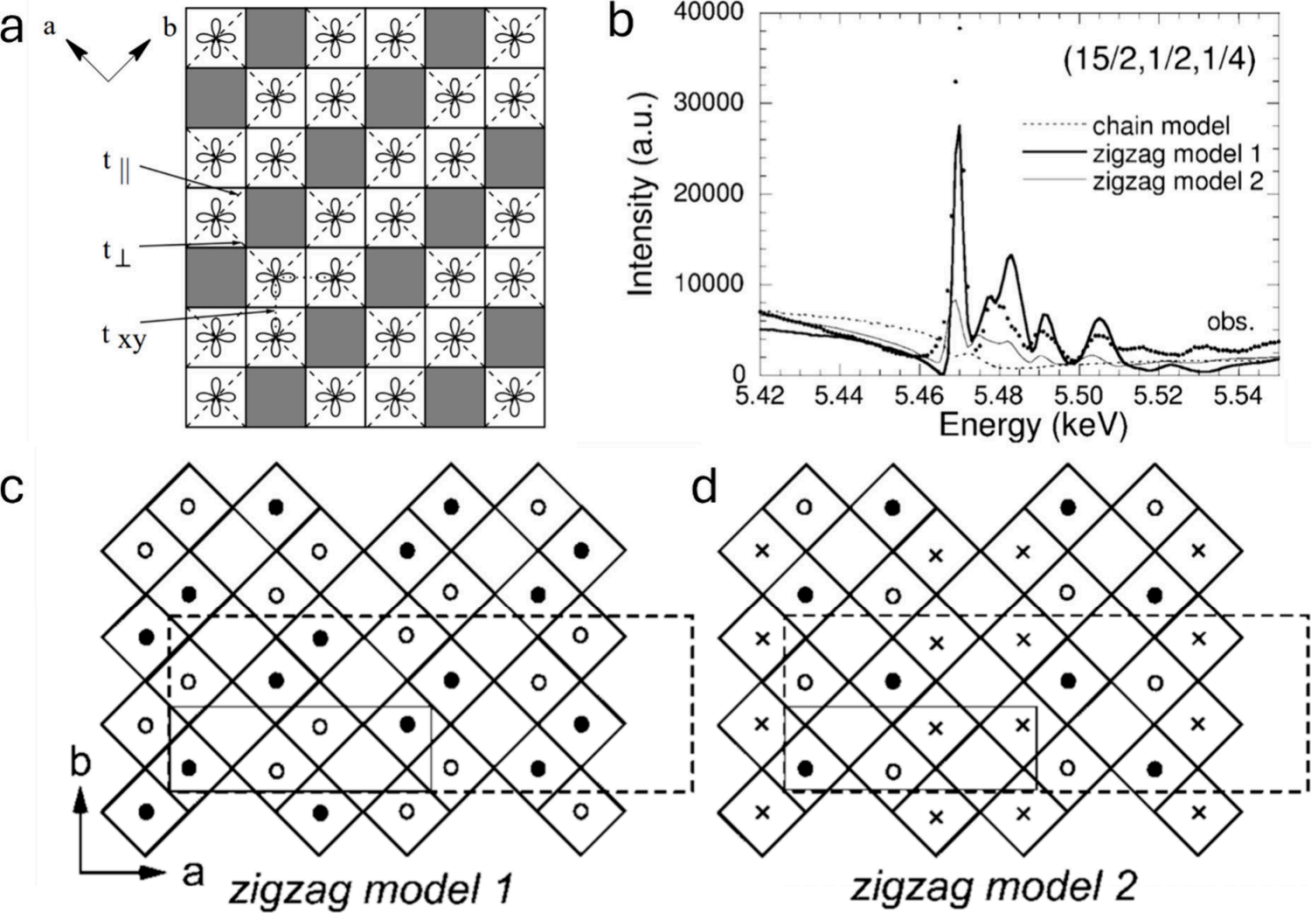
Evidence of charge ordering below 40K in α-NaV_2_O_5_. (a) V3*d*_*xy*_ orbital alignment and resulting orbital overlaps *t*_⊥_, *t*_∥_, and *t*_*xy*_. (b) Scattering intensity vs photon energy for the (^15^/_2_, ^1^/_2_, ^1^/_4_) reflection. (c,d) Two possible charge ordering patterns corresponding to panel (b). Filled and empty circles indicate V^4+^ and V^5+^ sites. Panel (a) was reproduced with permission from ref 349. Copyright 1999 *Solid State Communications*. Panels (b)–(d) were reproduced with permission from ref 353. Copyright 2000 *Physical Review Letters*.

α-NaV_2_O_5_ has generated significant interest in the solid-state physics community owing to its anisotropic structure, dimensional confinement of electronic states, and strong electron–electron and electron–lattice correlations. As we shall see in the following sections, it is an illustrative example of many other vanadium oxide bronzes.

#### 3.5.2 Peierls Distortions, Charge Density Waves, and Superconductivity in β-Na_0.33_V_2_O_5_

β-*M*_*x*_V_2_O_5_ phases feature guest cations *M* within tunnel-shaped interstices in the ζ-V_2_O_5_ host structure. The ions *M* = (Li^+^, Na^+^, Ag^+^, Ca^2+^, Pb^2+^, Sr^2+^) occupy paired seven-coordinate β sites within the tunnels, forming chains that run parallel to chains of edge-shared V_4_O_16_ subunits (Figure 34a,b). The proximity of adjacent pairs of sites prevents them from being occupied simultaneously and imposes a nominal maximum concentration *x* = 1/3, corresponding to half-occupied sites. Coulombic repulsion is minimized by adoption of a zigzag ordering of β-site occupancy, which only attains long-range order near *x* = 1/3. Ca^2+^, Pb^2+^, and Sr^2+^ exhibit such ordering at ambient temperature, whereas Na^+^ and Ag^+^ undergo a transition from disordered to ordered states at ca. 250 and 220 K, respectively.

**Figure 34:**
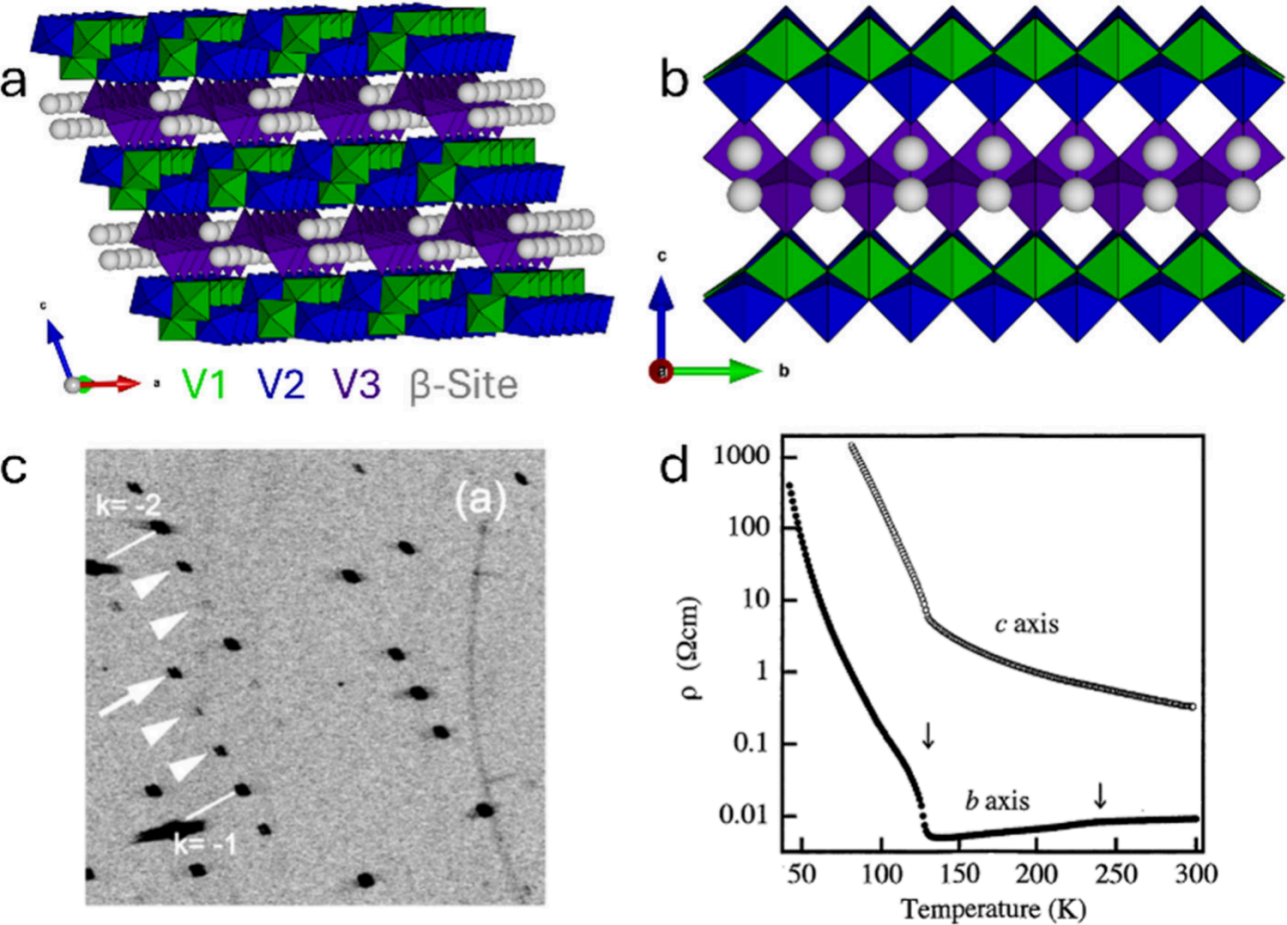
β-M_*x*_V_2_O_5_ structure. (a) pseudo-1D morphology and (b) arrangement of β sites along *b*. (c) Single-crystal X-ray diffraction pattern of β-Na_0.33_V_2_O_5_, with 1/2- and 1/6-order satellite reflections respectively indicated by long and short arrows. (d) Electrical resistivity of a single crystal vs temperature along *b* and *c* axes. Arrows indicate phase transition temperatures. Panel (c) was reproduced with permission from reference 354. Copyright 2002 Elsevier. Panel (d) was reproduced with permission from reference 355. Copyright 1999 *Journal of the Physical Society of Japan*.

Nearly all known β-*M*_*x*_V_2_O_5_’s exhibit transport nonlinearities associated with electronic structure transitions. Among those, the transitions in *M* = (Li^+^, Na^+^, Ag^+^, Ca^2+^, Sr^2+^) occur independently of guest ion ordering, suggesting that the underlying phenomena arise primarily on the V_2_O_5_ lattice. β-Na_0.33_V_2_O_5_ is by far the best-studied member of this family, thanks to the prolific single-crystal studies of Yutaka Ueda and collaborators.

β-Na_0.33_V_2_O_5_ undergoes three phase transitions when cooled from 300 K to below 27K. Below ca. 250 K, a doubling of the unit cell along *b* (parallel to the tunnels) occurs as sodium ions adopt long-range order, visible in single-crystal diffraction as satellite reflections with 1/2-order Miller indices (long arrows in Figure 34c).^354^ A 3D-ΔPDF experiment performed on single crystals reveals that zigzag ordering along *b* persists for individual Na^+^ chains above the ordering temperature, and that supercell formation results from correlation of the zigzag pattern phases between adjacent tunnels along *a*.^356^ However, the small effect this has on the material’s resistivity (visible in Figure 34d) supports the conclusion that conductive electron states are localized on the host lattice.

The reduced dimensionality of β-Na_0.33_V_2_O_5_’s electronic structure is evident in the difference in resistivity along the *b* and *c* directions in Figure 34d, and arises from the weak interactions between V_4_O_16_ units connected along *c* by V3 square pyramid chains. Additional support is provided by ARPES spectra collected from single crystals, which show significant band dispersity only along the *b* axis.^357^ The same study observed a maximum band energy at *k*_*b*_ *b* = π/4 at 160 K, while a value of π/12 is expected given the [V^4+^]/([V^4+^] + [V^5+^]) ratio of 1/6 presumed from the material’s stoichiometry. This can be accounted for by 1) the doubling of the unit cell (and thus division of the Brillouin zone) along *b* induced by sodium-ion ordering and 2) preference for occupying V_4_O_16_ chains (containing V1 and V2 sites) at the expense of V3 sites. Bond valence sums calculated from a single-crystal diffraction structure solution (Figure 35a) at 170 K confirm the latter conclusion.

**Figure 35:**
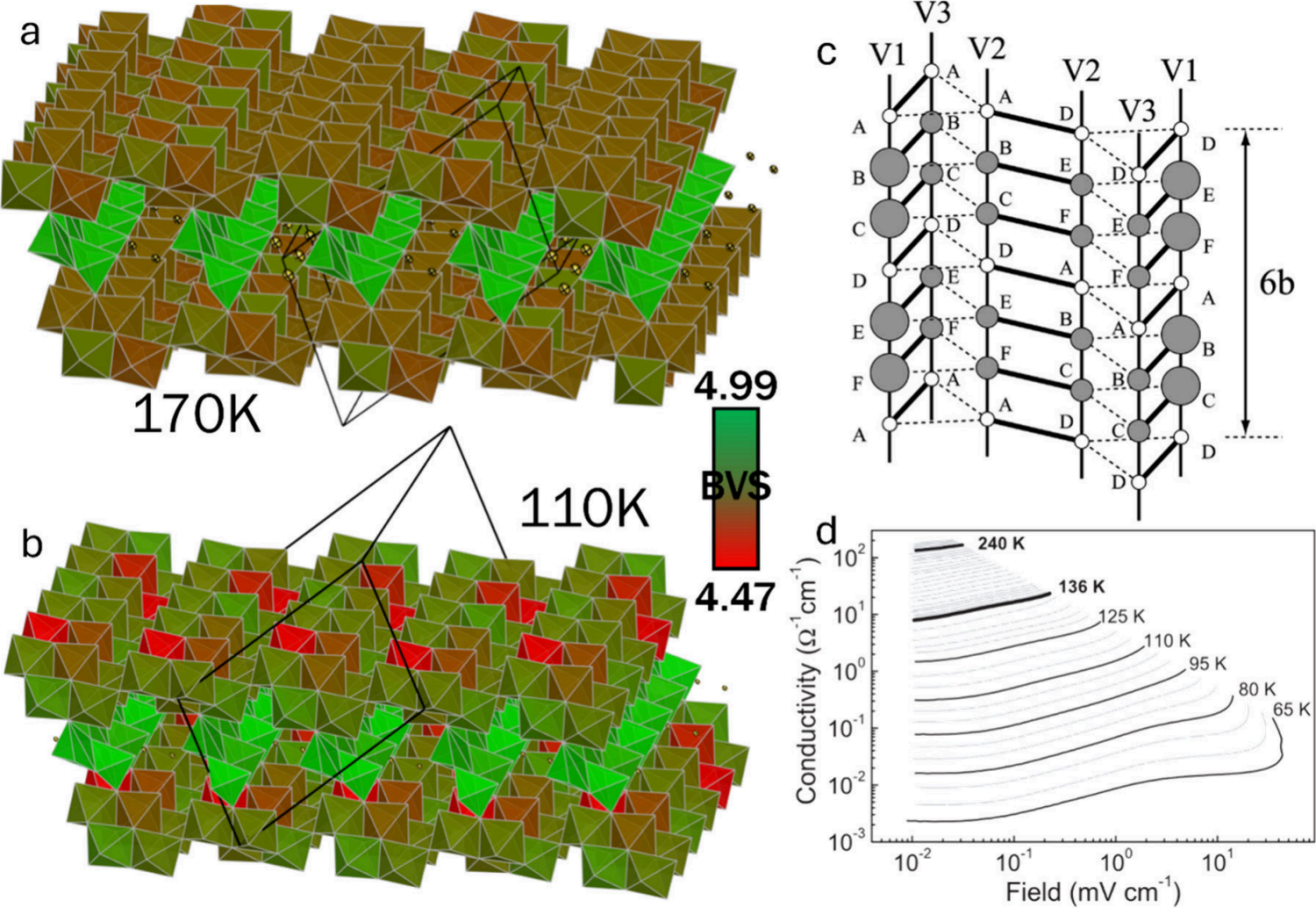
Evidence of charge localization in β-Na_0.33_V_2_O_5_. (a, b) Crystal structures solved from single-crystal diffraction data collected at (a) 170 K and (b) 110 K, with unit cells drawn in black and vanadium-centered polyhedra color-coded according to oxidation state as determined from bond valence sum calculations. (c) Electron occupancy determined from angle-resolved NMR and neutron scattering data. (d) Field- and temperature-dependent conductivity measured along *b*. Panel (c) was reproduced with permission from reference 80. Copyright 2006 American Physical Society. Panel (d) was reproduced with permission from reference 358. Copyright 2005 *Journal de Physique*.

Figure 35b shows bond valence sums at 110 K, suggesting that the insulating state adopted below ca. 135 K (see Figure 34d) involves a further localization of electrons within each V_4_O_16_ chain with a period of 3*b* (to give a total supercell length of 6*b*). This pattern essentially matches that determined from fitting electron localization models to single-crystal neutron scattering and angle-dependent NMR data presented in Figure 35c, constituting strong evidence for Peierls behavior and charge density wave formation.^80,359^ Field-dependent conductivity measurements exhibit three conduction regimes at 65 K, characteristic of CDWs in one-dimensional materials.^358^ According to this interpretation, conduction up to ca. 0.6 mV/cm (as shown in Figure 35d) occurs by migration of small polarons. From 0.6 to 30 mV/cm, conduction is dominated by depinning of incommensurate CDWs from defects. Above 30 mV/cm, commensurate CDWs depin from the lattice, freeing the associated electrons for conduction and generating an enormous increase in current. The low threshold voltages for CDW depinning and relatively small Peierls distortions in the insulating state suggest significant interactions between adjacent one-dimensional electron states, so that β-Na_0.33_V_2_O_5_ can be considered a 1D-2D crossover system.

The magnetic behavior of β-Na_0.33_V_2_O_5_ is marked by a transition at *T*_C_ = 24 K, indicating the onset of long-range magnetic ordering. The system exhibits strong antiferromagnetic interactions along the *b* axis and weaker couplings in the *ac* plane, consistent with the one-dimensional nature of electron interactions. The magnetic transition is highly sensitive to Na nonstoichiometry, with deviations from *x* = 0.33 leading to a significant suppression in both *T*_C_ and magnitude of the anomaly. The transition becomes undetectable when *x* = 0.30 or *x* = 0.345, indicating that even slight variations in Na concentration disrupt the magnetic ordering. Notably, the suppression effect is more pronounced for Na excess (*x* > 0.33) than for Na deficiency (*x* < 0.33), highlighting the critical role of Na ordering in stabilizing the magnetic state.^355^

The decrease in MIT temperature observed in this material with applied isostatic pressure agrees with the proposed origins outlined above, as increased pressure will magnify interactions between 1D states and destabilize the CDW.^360^ The effect of pressure on the electronic structure of β-*M*_*x*_V_2_O_5_ materials is of particular interest due to the presence of superconductivity at sufficiently low temperatures and high pressures, particularly in β-Li_0.33_V_2_O_5_, β-Na_0.33_V_2_O_5_, and β-Ag_0.33_V_2_O_5_.^361,362^ Figure 36 shows P-T electronic phase diagrams for each compound. The trend that pressure suppresses the MIT temperature in each material is consistent with the contingent one-dimensionality of the shared V_2_O_5_ framework, whereas the large dependence of the transition temperature on the ion identity implies that guest ion–V_2_O_5_ interactions affect the stability of the 1D CDW states. The stability of the CDW state in β-Li_0.33_V_2_O_5_ is also reflected in the high pressure needed to induce its superconductivity, and in conjunction with other evidence^362^ strongly supports dimensionality crossover as the basis for superconducting state formation.

**Figure 36:**
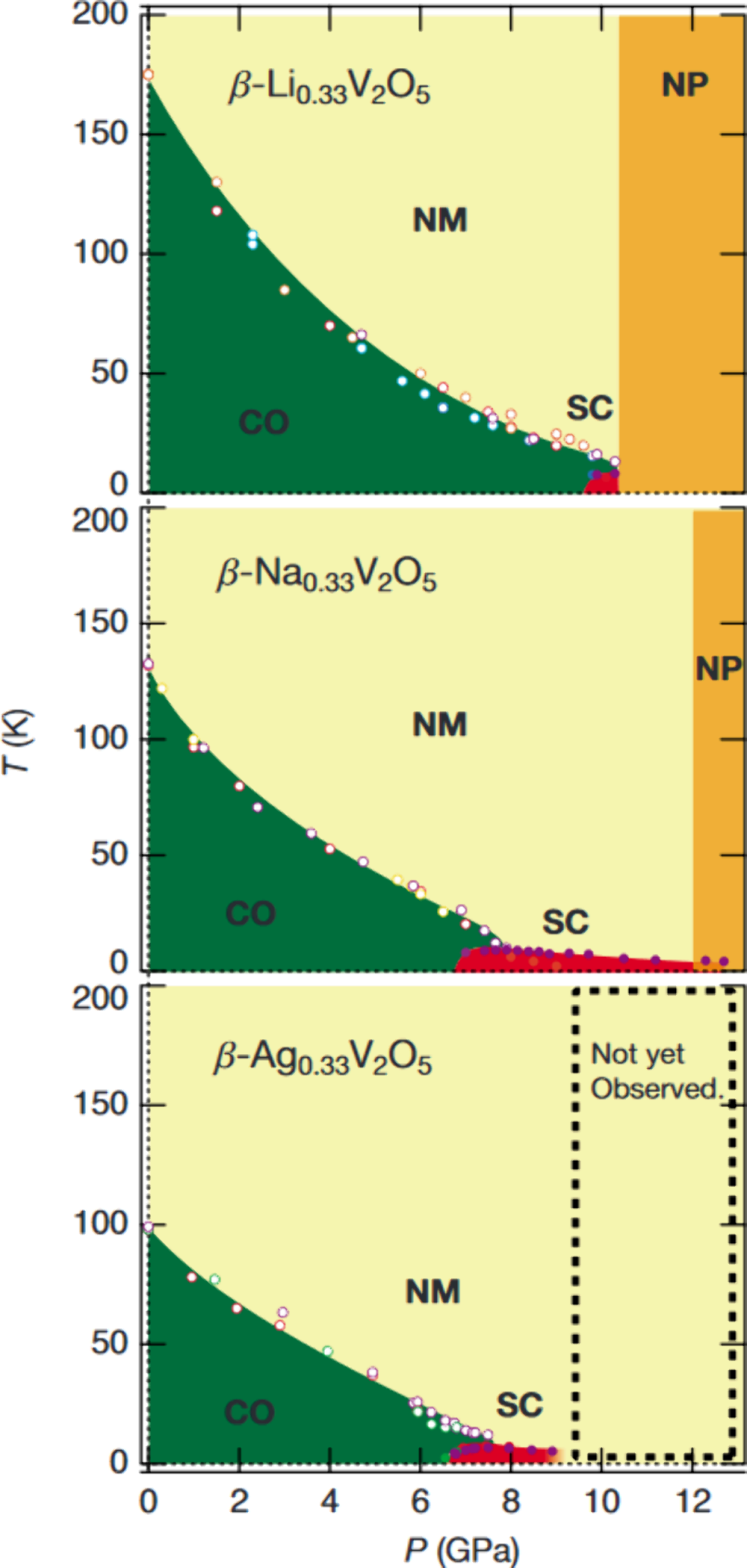
Electronic *P*–*T* phase diagrams for the indicated materials. NM = “normal metal”, CO = “Charge ordering” (CDW), SC = “Superconducting”, NP = “New Phase”. This figure was reproduced with permission from reference 362. Copyright 2008 American Physical Society.

#### 3.5.3 Coupled Order–Disorder and Metal–Insulator Transitions in Cu_*x*_V_2_O_5_ Bronzes

The electronic structure transitions we have discussed so far have arisen from electronic structures located primarily on the V_2_O_5_ framework with minimal or no mechanistic involvement of guest ions. Two related materials, β′-Cu_*x*_V_2_O_5_ and ε-Cu_0.9_V_2_O_5_, stand out as counterexamples, wherein guest ion ordering is strongly entangled with electron localization. A comparison of the electronic and structural transition behaviors of these materials will prove fruitful for understanding, and eventually controlling, host–guest interactions in *M*_*x*_V_2_O_5_ compounds.

β′-Cu_*x*_V_2_O_5_ is isostructural with the β-*M*_*x*_V_2_O_5_ phases discussed in the previous section, albeit with copper ions occupying β′ sites—offset from β sites by *b*/2—and exhibiting significant split-site disorder (Figure 37a). Unlike β sites, neighboring β′ sites are far enough from each other to allow simultaneous occupancy, reflected in a much wider solubility range, 0.26 ≤ *x* ≤ 0.65.^35^ β′-Cu_*x*_V_2_O_5_ materials exhibit an resistive anomaly upon cooling below a critical temperature (Figure 37b) that depends closely on the stoichiometry *x*. This is in marked contrast to the β-*M*_*x*_V_2_O_5_ compounds noted above, where even small compositional deviation from *x* = 1/3 suppress phase transitions. Superstructure formation attributed to Cu ion ordering along *b* has been observed in single-crystal diffraction at several stoichiometries *x* < 0.5, coinciding with the electronic structure transition.^363^ This structure modulation was found to be incommensurate (***q*** *<* 3 ***b***) with the crystal lattice for *x* < 0.4, including for *x* = 0.33, and commensurate (***q*** = 3 ***b***) for *x* = 0.4.^364^ The presence of electronic transitions at compositions which nominally give incommensurate V 3*d* band filling excludes a simple Peierls mechanism, whereas the coincidence of the electronic and atomic structure transitions implies that electron localization on the V_2_O_5_ framework is highly sensitive to Cu-ion motion. Indeed, a strong relationship between temperature, composition, Cu split-site behavior and electronic structure has been established, evident in the change in Cu-ion distribution between the central and split sites plotted in Figure 37c.^365^

**Figure 37:**
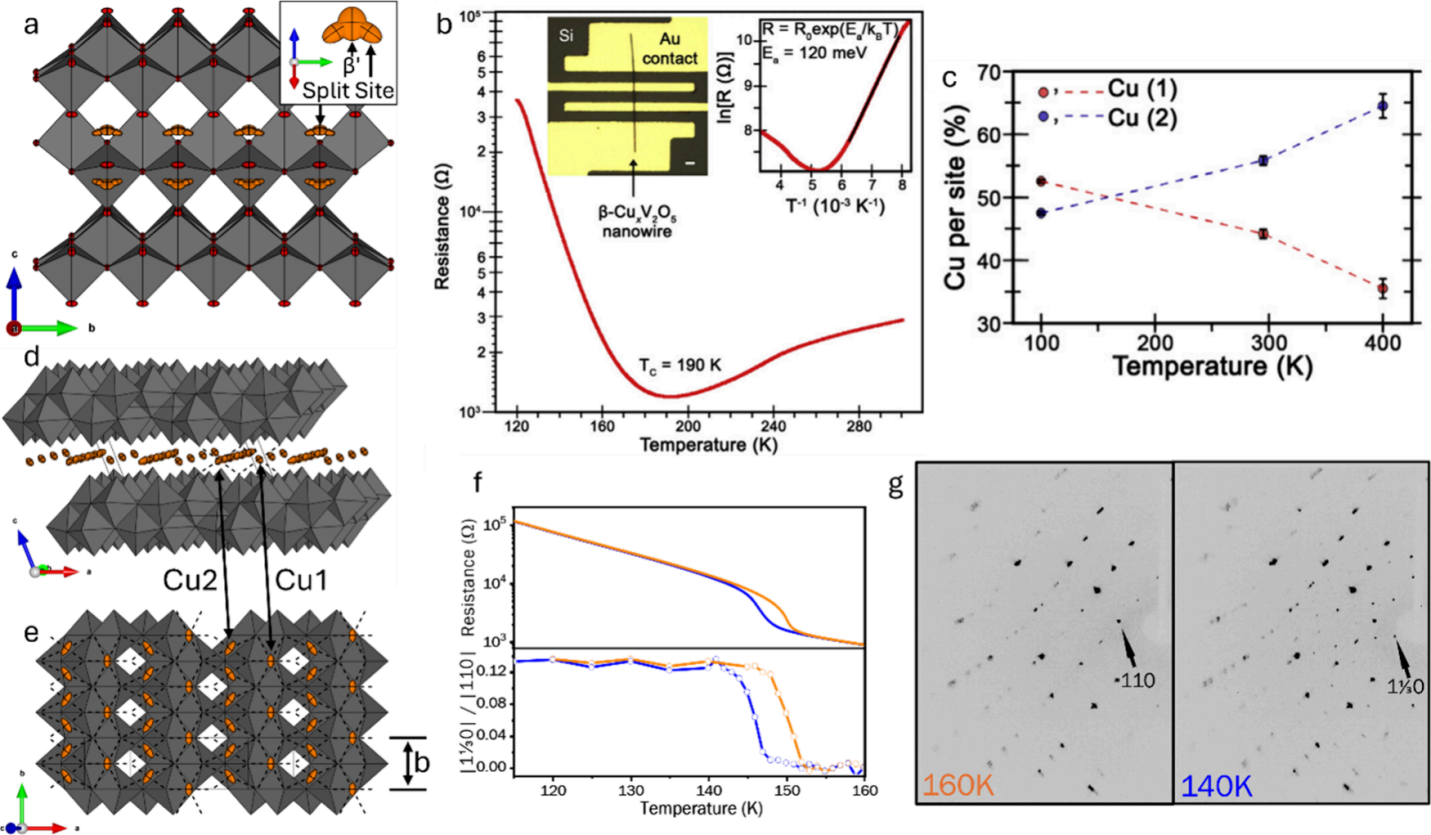
Structure and MIT behavior of β′-Cu_*x*_V_2_O_5_ and ε-Cu_0.9_V_2_O_5_. (a) Side view of tunnel in single-crystal β′-Cu_*x*_V_2_O_5_ structure at 110 K. Inset shows detail of Cu split sites. (b) Resistance vs temperature for a single β′-Cu_*x*_V_2_O_5_ nanowire. Inset shows activated conduction fit. (c) distribution of Cu-ion occupancy between the central Cu (1) site and Cu (2) split site, the latter indicative of Cu-ion disorder. (d, e) Two views of ε-Cu_0.9_V_2_O_5_, Cu1 and Cu2 chains. (f) Electrical resistance (top) and normalized 1 1/3 0 reflection intensity (bottom) vs temperature for ε-Cu_0.9_V_2_O_5_. (g) Single-crystal diffraction patterns taken above (160 K) and below (140 K) the MIT temperature, showing supercell reflections in the low-temperature state. Panels (b) and (c) were reproduced with permission from reference 365. Copyright 2020 Cell Press. Panels (f) and (g) were reproduced from reference 366 under a CC BY 4.0 license. Copyright 2024 Ponis et al.

ε-Cu_0.9_V_2_O_5_ similarly features extended parallel chains of Cu ions along *b*. Rather than being isolated from each other within tunnels, octahedral Cu1 and square-planar Cu2 sites occupy a two-dimensional interstitial space sandwiched between V_2_O_5_ double-layers (Figure 37d,e). Each Cu ion thus interacts with neighbors along multiple axes, and simulations show facile hopping between adjacent sites at ambient temperature.^366^ This is facilitated by nominal half-filling of Cu2 sites, whose proximity precludes complete occupancy. Beyond this, Cu1 and Cu2 sites show a strong preference for slight substoichiometric filling (hence the compositional deviation from one Cu per V_2_O_5_). ε-Cu_0.9_V_2_O_5_ was recently found to undergo a sharp conductivity transition (Figure 37f) coupled to a nearly commensurate Cu ion occupancy ordering transition resulting in the formation of an approximate 1 × 3 × 1 supercell (Figure 37g), a phenomenon which has been exploited to fashion oscillator circuits from entire single crystals, as shown in Figure 38a,b.^366^ In addition to the expected supercell reflections, single-crystal diffraction patterns collected in the insulating state (Figure 38c) contain significant diffuse scattering and long-range modulation satellites, although the modulated structure has yet to be solved. A similar behavior (Figure 38d,e) has been partially characterized in the related τ-Cu_*x*_Ag_*y*_V_2_O_5_ ^45^ and highlights the complex ordering behavior that may arise from two-dimensional cation–cation interactions.

**Figure 38:**
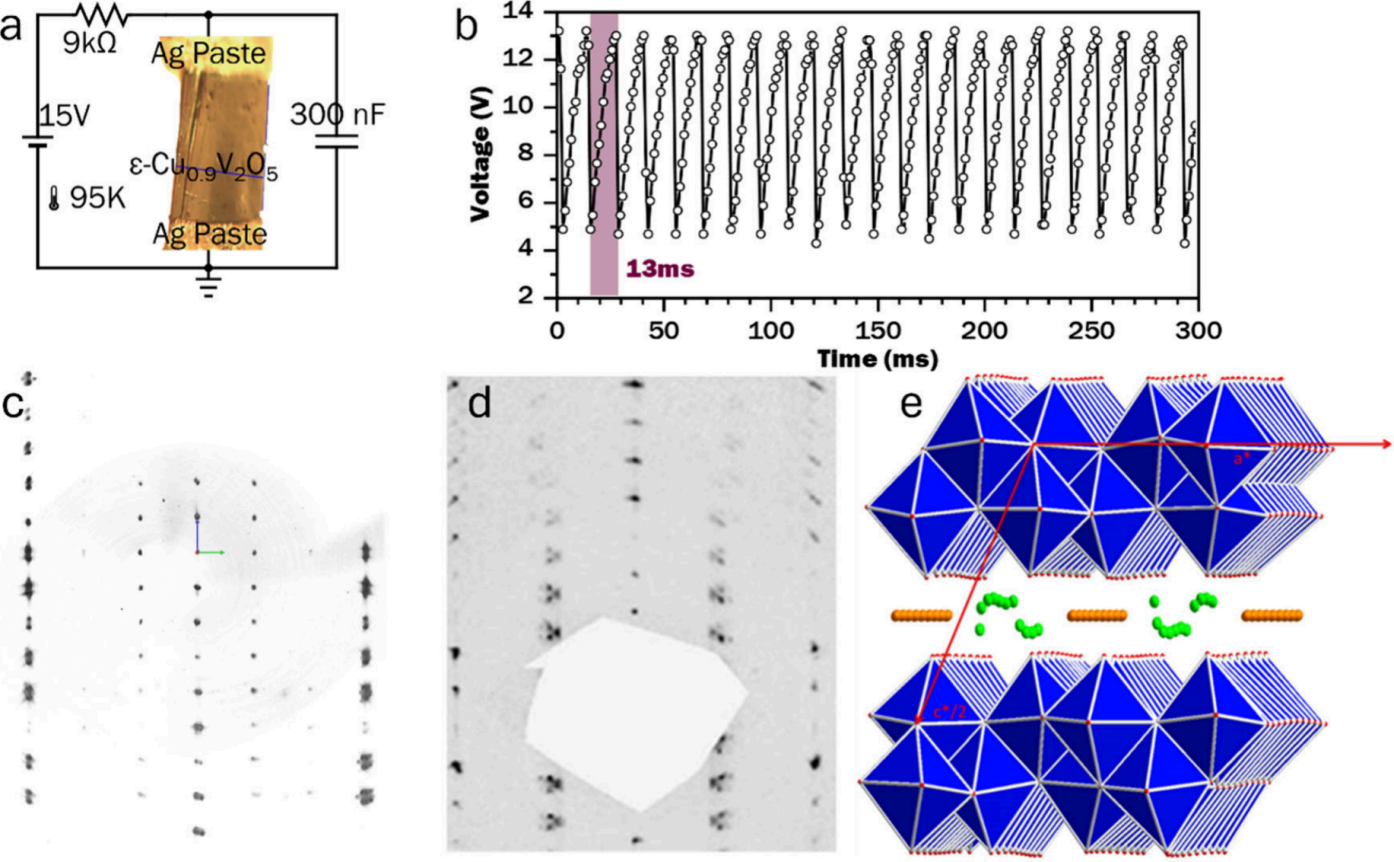
Evidence of structure modulation in layered M_*x*_V_2_O_5_. (a) Schematic of ε-Cu_0.9_V_2_O_5_ single-crystal oscillator circuit and (b) oscillations produced at the specified conditions. (c) 2KL scattering plane of ε-Cu_0.9_V_2_O_5_ collected at 110 K. (d) 2KL scattering plane of τ-Cu_*x*_Ag_*y*_V_2_O_5_ at 100 K. (e) Modulated structure of τ-Cu_*x*_Ag_*y*_V_2_O_5_, Cu in orange, Ag in green. Panels (a) and (b) were reproduced from reference 366 under a CC BY license. Copyright 2024 Ponis et al. Panels (d) and (e) were reproduced with permission from reference 45. Copyright 2013 Elsevier.

In both ε-Cu_0.9_V_2_O_5_ and β′-Cu_*x*_V_2_O_5_, the coupling of metal–insulator and cation order–disorder transitions suggests relatively strong coupling of Cu ions to electron states on the V_2_O_5_ guest structure. As is typical for vanadium oxide bronzes, the guest Cu ions coordinate to vanadyl oxygens that interact covalently with vanadium ions. The short vanadyl bond dictates the orientation of the low-energy V 3*d*_*xy*_ orbitals, so distortions due to disorder on coordinated cation sites can interrupt overlap along infinite chains. The unique strength of Cu–V_2_O_5_ interactions likely results from a combination of factors. The Cu^+^ ion is a considerably stronger Lewis acid than alkali or alkaline earth ions. The small size of Cu^+^ ions positions them close to the vanadium oxide lattice, evident in the spacing of β′ vs β sites in the tunnel bronzes and in the narrow interlayer distance in ε-Cu_0.9_V_2_O_5_ compared to analogs.^32^ The copper ion exhibits the lowest coordination number of any ion in both structure types, concentrating the effects of disorder on fewer bonds. Finally, Cu 3*d* states reside at the top of the valence band and contribute significantly to shaping the Fermi surface.

The magnetic behavior of β-Cu_*x*_V_2_O_5_ varies with copper stoichiometry, *x*, revealing distinct magnetic transitions at specific temperatures. For *x* ≈ 0.60, the magnetic susceptibility remains nearly temperature-independent over a wide range, deviating from Curie–Weiss behavior. In contrast, for β-Cu_0_._29_V_2_O_5_, a slight increase in susceptibility occurs at *T*_t_ ≈ 210 K, whereas for β-Cu_0_._40_V_2_O_5_, a minor decrease is observed at *T*_R_ ≈ 180 K. Further studies indicate that the transition at *T*_R_ occurs within a narrow composition range (0.38 ≤ *x* ≤ 0.42), and below this temperature, χ­(*T*) becomes sensitive to measurement conditions. After slow cooling, only a slight decrease is noted at *T*_R_, resembling the behavior seen in electrical resistivity. At 5 K, the susceptibility increases to 2 × 10^–3^ emu per vanadium mole due to Curie-like behavior, suggesting the presence of localized magnetic moments. The magnetic transitions observed at 180 and 210 K could be attributed to V^4+^–O–V^4+^ superexchange interactions arising from the cooperative ordering of Cu^+^ and V^4+^ ions.^363^

#### 3.5.4 Spin–Orbital Ordering in *RE*VO_3_ Perovskites

The rare-earth orthovanadites *RE*VO_3_, *RE* = (La–Lu, Y), adopt an orthorhombically distorted (here *Pnma*, the equivalent alternate setting *Pbnm* is frequently used in literature) perovskite structure with a V^3+^ (*d* ^*2*^ *)* oxidation state shown in Figure 39a. Rare-earth ions occupy the cubic *A* site, while vanadium ions occupy the octahedral *B* site of the idealized perovskite. Geometric distortion and competition between Jahn–Teller and superexchange effects ensure that, despite the high symmetry of the parent structure, the rare-earth orthovanadites each undergo a series of transitions between atomic and electronic phases whose structure and physical properties exhibit various degrees of anisotropy. YVO_3_ is the best-studied member of this family owing to its strange magnetic behavior.

**Figure 39:**
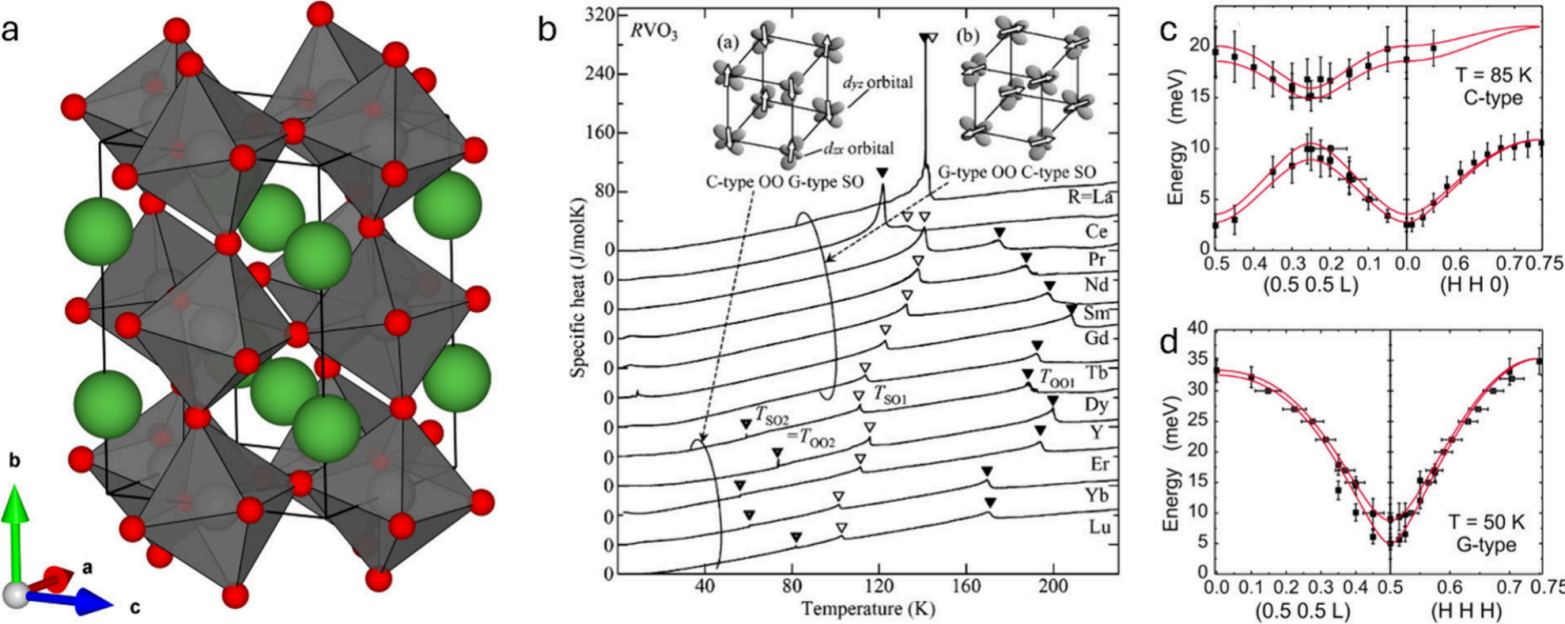
REVO_3_ structure and phase transition behavior. (a) Distorted perovskite structure of REVO_3_, RE ions in green. (b) Specific heat curves for REVO_3_s. Peaks associated with onsets of orbital and spin ordering regimes (depicted in inset) marked with triangles, as described in main text. (c, d) YVO_3_ magnon dispersion curves determined from inelastic neutron scattering at 85 and 50 K. H and L reciprocal axes respectively correspond to a* and b* directions for the structure in panel (a). Panel (b) was reproduced with permission from reference 367. Panels (c) and (d) were reproduced with permission from reference 368. Panels (b)–(d) Copyright 2003 American Physical Society.

At room temperature, all members are paramagnetic Mott–Hubbard insulators.^367^ The small size of rare earth ions relative to the size of *A* site induces a compression and tilting of the VO_6_ octahedra, an effect which significantly increases across the La–Lu series as a result of lanthanide contraction.^369^ Octahedral compression stabilizes V 3*d*_*xy*_ states but preserves the degeneracy of 3*d*_*xz* */yz*_ states which host the remaining *d* electron. This tilting also increases the size of the unit cell to √2 × 2 × √2 times the values of the cubic perovskite, with the *a* and *c* axes rotated 45°. Cooling below a temperature indicated by black triangles in Figure 39b introduces a “G-type” orbital ordering (G-OO), that is with occupation of V 3*d*_*xz*_ and 3*d*_*yz*_ orbitals (and accompanying Jahn–Teller elongation) alternating between nearest-neighbors in all three directions as shown in the inset. A monoclinic transition results from the broken mirror symmetry perpendicular to *b*, as evidenced by X-ray diffraction and Raman spectroscopy on single crystals of YVO_3_ ^370–372^ and electron diffraction on crystals of LaVO_3_.^373^

Further cooling (white triangles in Figure 39b) triggers a transition to a “C-type” spin ordering (C-SO, antiferromagnetic in the *ac* plane, and ferromagnetic along *b*) overlaid upon the G-OO structure. Magnon dispersion relations in YVO_3_ measured using single-crystal inelastic neutron diffraction (Figure 39c) are characteristic of canted ferromagnetic ordering along the *b* axis, and suggest two superexchange coupling strengths possibly due to partial orbital disorder in the *ac* plane in the G-OO/C-SO state.^368^ Synchrotron diffraction shows continuous increase in the monoclinic distortion of YVO_3_ with cooling, which supports the gradual onset of G-OO structure (and thus orbital disorder) even below the C-SO transition.^372^

Below a temperature indicated by double-triangles in Figure 39b, several REVO_3_ compounds with small *A*-site ions (Gd–Lu, Y) undergo a simultaneous orbital-spin reordering. The ferromagnetic coupling along *b* converts to an antiferromagnetic ordering (G-SO), whereas V 3*d*_*xz* */yz*_ orbitals cease to alternate along *b* and the monoclinic Jahn–Teller distortion disappears (C-OO), as evidenced by single-crystal Raman spectroscopy and X-ray diffraction.^367,370^ The magnon dispersion curves as measured by single-crystal inelastic neutron scattering (Figure 39d) in YVO_3_ become nearly isotropic, whereas optical conductivity features assigned to G-OO ordering are suppressed.^374^

Trends in ordering temperature between rare earth ions have been attributed to the decreasing ionic radius from La to Lu and the distorting effect this has on the VO_3_ framework.^367^ Decreased superexchange across bent V–O–V bonds weakens magnetic interactions and suppresses the C-SO transition temperature. The distortion is, however, cooperative with the Jahn–Teller distortion caused by orbital ordering. Competition between stabilization of Jahn–Teller distortions and weaking V–O–V interactions is likely responsible for the reversal of the trend in the G-OO transition temperature at RE = Gd. This sensitivity to rare-earth element identity is exemplary of the tunability of ternary vanadium oxides by composition modification.

Furthermore, doped perovskite vanadates such as La_1–*x*_Sr_*x*_VO_3_, Pr_1–*x*_Ca_*x*_VO_3_, Nd_1–*x*_Sr_*x*_VO_3_, and Y_1–*x*_Ca_*x*_VO_3_ show conductance nonlinearities depending on the extent of alloying (*x*) and the specific rare-earth element.^273^ At room temperature, all the pristine members exhibit a paramagnetic Mott–Hubbard insulator behavior as stated above.^367^ Partially substituting the rare-earth metals with alkaline-earth metals introduces hole doping (decreasing band filling), and generally induces an insulator-to-metal transition, where the *x*_c_ (the critical doping level) influenced by the tolerance factor, which governs structural distortions and electronic bandwidth. The *x*_c_ for the MIT occurs at 0.178 for La_1–*x*_Sr_*x*_VO_3_, 0.23 for Nd_1–*x*_Sr_*x*_VO_3_, 0.25 for Pr_1–*x*_Ca_*x*_VO_3_, and 0.5 for Y_1–*x*_Ca_*x*_VO_3_ with the corresponding MIT temperatures being 60 K (La), 50 K (Nd), 80 K (Pr), 100 K (Y).^273–275^ These variations arise from differences in ionic radii and electronic configurations, which directly influence the strength of electron correlation.^273^

#### 3.5.5 Charge/Orbital Ordering and Metal–Insulator Transitions in Hollandite Vanadates

Hollandite vanadates represent a fascinating class of transition metal oxides with the general formula *A*_*x*_ *M*_8_O_16_, where *A* is a monovalent, divalent, or trivalent cation and *M* is typically a transition metal (Figure 40). These compounds consist of double chains of edge-sharing MO_6_ octahedra that form one-dimensional (1D) tunnels along the *c* axis (Figure 40b,c), which host interstitial cations.^376^ The resulting low-dimensional framework supports strong anisotropic electronic interactions and correlated electron phenomena, making these systems ideal for exploring MITs. For instance, in Bi_1.8_V_8_O_16_, the MIT occurs at ∼62.5 K and is associated with a structural transition combined with charge ordering between V^3+^ and V^4+^ (Figure 40b) cations.^377,378,375^ Pb_1.6_V_8_O_16_ undergoes a similar transition near 140 K, also accompanied by structural distortions and charge/orbital ordering (Figure 40b,c).^379,380^ In K_2_V_8_O_16_ the MIT occurs at 170 K and is marked by a two-step resistivity jump spanning 3 orders of magnitude underpinned by a structural transformation from tetragonal to monoclinic symmetry upon heating and is further accompanied by a decrease in magnetic susceptibility.^276^ The low-temperature insulating phase exhibits charge ordering of V^3+^ and V^4+^, wherein V^4+^–V^4+^ and V^3+^–V^3+^ dimers form spin singlets. In Rb_2_V_8_O_16_, the MIT occurs at 220 K, but the resistivity jump is less pronounced.^277^ As in K_2_V_8_O_16_, a pronounced decrease in magnetic susceptibility is observed upon heating suggesting V^3+^ and V^4+^ charge pairing. However, no clear superlattice ordering is observed, likely as a result of Rb nonstoichiometry. All the compounds undergo first-order MITs with volume expansion in the insulating phase; their high-temperature metallic states are expected to be suppressed under high pressure. Based on the filling level within the (1D) tunnels, the formal oxidation state of vanadium exceeds 3.5 in K_2_V_8_O_16_ (3.75), Rb_2_V_8_O_16_ (3.75), and Pb_1.6_V_8_O_16_ (3.60), but is lower in Bi_1.8_V_8_O_16_ (3.325). This reduced vanadium oxidation state in the bismuth hollandite correlates with its lower metal–insulator transition temperature.^377^

**Figure 40:**
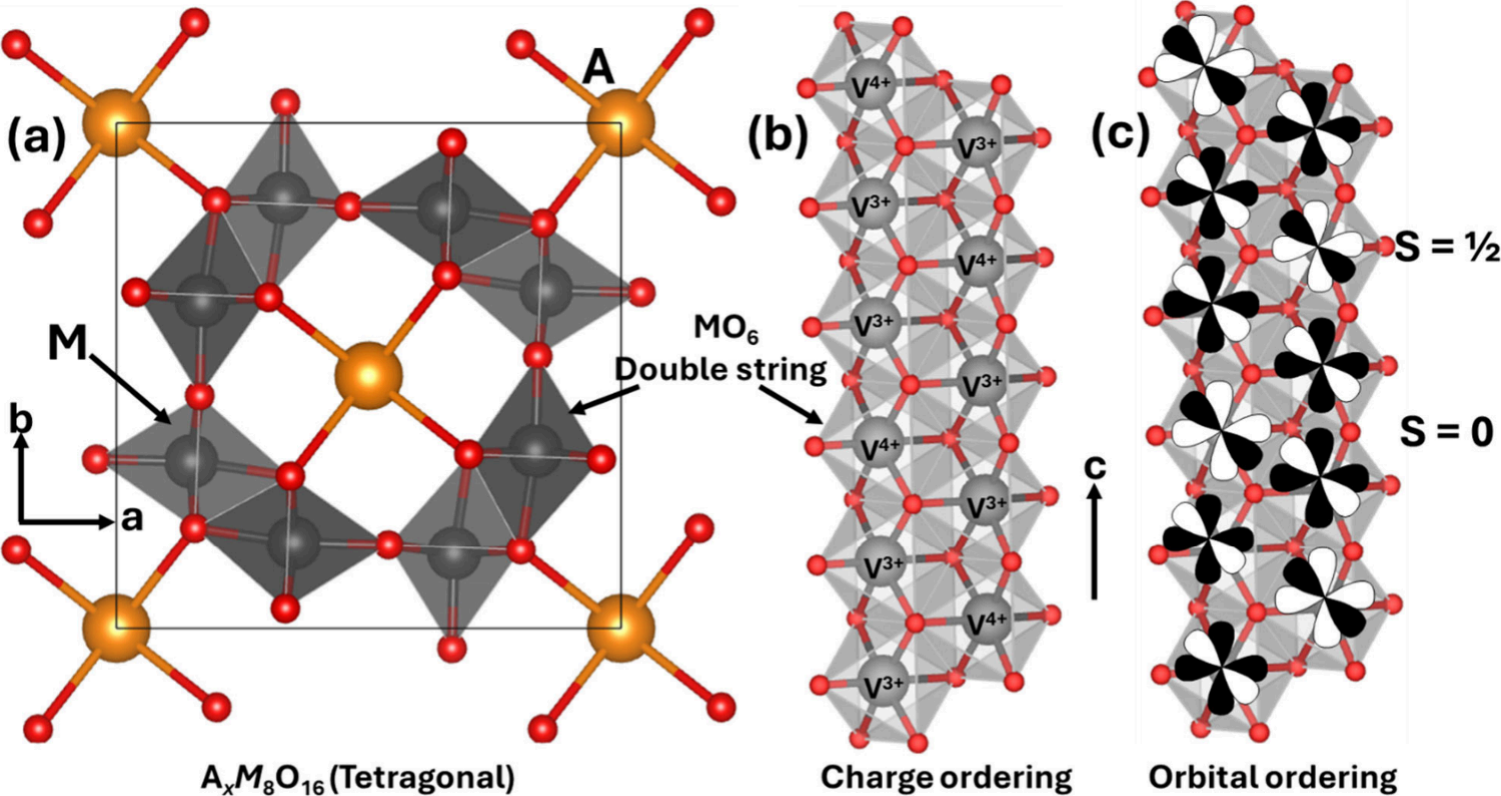
(a) Structure of tetragonal (*I*4/*m*) hollandite *A_x_M*_8_O_16_ compound, where *A* is a monovalent, divalent, or trivalent cation and *M* is typically a transition metal. The model of the (b) charge ordering and (c) orbital ordering. The solid and open lobes represent occupied and unoccupied *t_2g_* orbitals, respectively. V^3+^–V^3+^ forms *S* = 0 below *T*_MIT_ and the remaining V^4+^ forms *S* = 1/2 and contribute to C–W behavior.^375^

The preceding sections illustrate that binary, ternary, and more complex vanadium oxides exhibit a diverse range of mechanisms underpinning electronic instabilities that can be systematically modulated. The rich and tunable range of electronic structure and its implications for strongly nonlinear electrical conductance upon the application of power (voltage or current) to a device where it is an active element has generated considerable excitement for emulation of information processing architectures in mammalian brains. Vanadium oxides exhibiting MITs, spin ordering transitions, and Peierls transitions are among the frontrunners for neuronal emulation such as the design of oscillators and amplifiers.

## 4 Application Highlight: Neuromorphic Computing

### 4.1 Neuronal and Synaptic Information Processing Based on Nonlinear Dynamical Modulation of Conductivity

The arrival of the information age, shepherded by Moore’s Law that saw the aggressive scaling of transistor integration, has seen the incorporation of digital technology into nearly every aspect of our information economies. Artificial intelligence (AI) is poised to revolutionize our relationship to technology once again, but current implementations demand enormous amounts of computing power.^381,382^ Processing, storing, and transmitting information currently accounts for approximately 10% of global energy consumption, and projections indicate that by 2040, computational energy demands could exceed the global energy supply by a factor of 10.^383^ As the initial footfalls of AI are felt across our information economies, energy consumption for computing is on an altogether unsustainable trajectory. The emergence of AI further implies that much of future computing will need to be performed at the edge under severe power constraints. Given that digital complementary metal oxide semiconductor (CMOS) technology is fundamentally constrained by the Fermi–Dirac distribution of electron energies, and many of the thermal scaling and speed characteristics of CMOS have already saturated, the traditional exponential improvements that have accrued by scaling CMOS are no longer available. Traditional von Neumann architectures physically separate data storage and processing tasks, and the constant shuttling of data between logic and memory units is exceedingly energy intensive. With the global demand for computation set to strain available energy supply within the next 20 years,^384^ the future of computing will be defined not by continued miniaturization of silicon circuitry but by the development of new, fundamentally different energy efficient paradigms such as those emulating the sparsity and energy efficiency of mammalian brains.

Mammalian brains provide a powerful alternative example of energy-efficient information processing, leveraging closely intertwined synapses and neurons to perform transmission, memory, and logic functions within a singular fabric.^385–388^ In biological neural circuitry, underpinned by ion transport mediated by ion channels and through release and uptake of molecular neurotransmitters, neurons integrate signals from multiple synaptic connections, process information across dendritic networks, and generate outputs through precisely timed trans-synaptic pulses.^389^ Collectively, neurons give rise to emergent behavior such as adaptive event-based learning and enable processing of large data sets with real-time updating. Synapses (Figure 41a) individually weight spike signals and mediate transmission from presynaptic neurons (1) to postsynaptic neurons (2) across the synaptic cleft (3). Transmission begins when voltage-gated calcium channels (4) open, inducing synaptic vesicles (5) to release neurotransmitter molecules into the cleft. Neurotransmitters in turn open sodium channels (6) and enable influx of sodium ions. The spike in sodium concentration perturbs the neuron’s resting state membrane potential (1 in Figure 41b). Transient membrane depolarizations (2) decay to the resting voltage unless they accumulate in sufficient numbers (3) to surpass a threshold voltage and initiate an action potential (4), which propagates along the axon to downstream neurons. Potassium efflux repolarizes the membrane (5), and a refractory period (6) ensues while ion pumps re-establish the resting state. Neuron properties exhibit multiple orders of complexity,^390^ exemplified by the existence of excitability classes (Figure 41c).^391^ The spiking frequency of Class 1 neurons shows a continuous relationship to an applied DC current, Class 2 neurons only begin periodic spiking (and Class 3 neurons only emit a single spike) above a threshold current.

**Figure 41:**
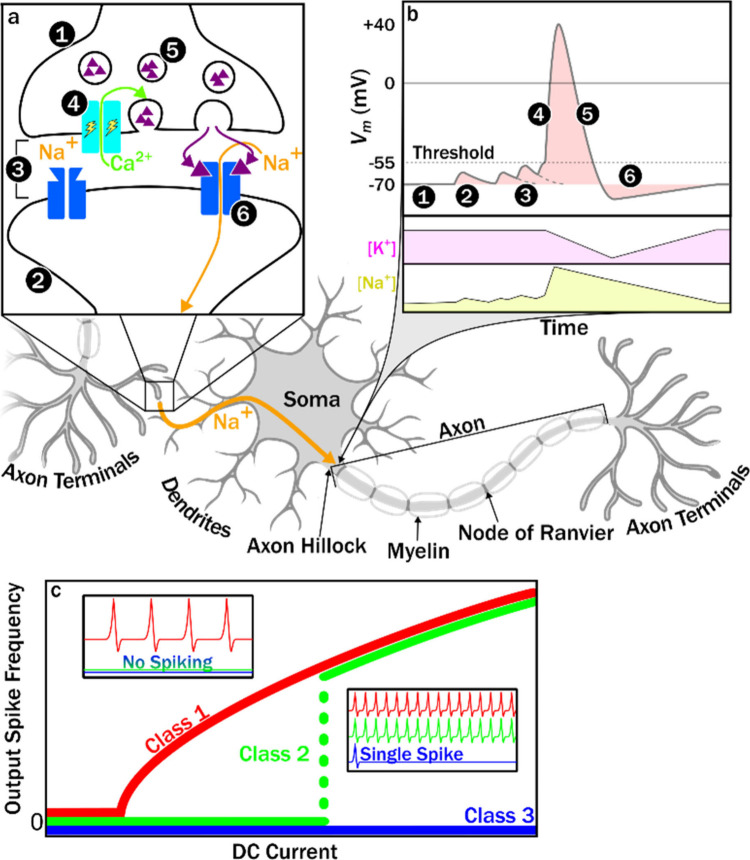
Biological neuron and synapse function. (a) Schematic cross-section of a synaptic junction showing signal transmission mechanism. (b) Membrane voltage vs time for single action potential, generated by transmembrane ion concentration gradients (bottom). Numbered elements identified in main text. (c) Spiking behavior of the three excitability classes under applied DC current.

A neuron maintains a charge imbalance across an impermeable membrane, which is bridged by sodium and potassium channels. Each channel contains voltage-activated “gates”, which all must be open for ions to flow through the channel. The probability that a given gate is open or closed depends on the voltage across the membrane. There are three different kinds of gates in the classic Hodgkin–Huxley model,^392^ but the potassium (K) channels are made of just one kind of gate. This model essentially provides state variables whose modulation can position the system at what Chua described as the “edge of chaos” ^393–396^ where controllable semistable oscillations and action potentials can be obtained as encapsulated in Figure 41.

Neuromorphic computing aims to mimic, and even surpass, the energy efficiency of the human brain, but is predicated on the design and realization of a whole new set of neuron- and synapse-like materials that can serve as building blocks for device architectures emulative of neuronal circuitry.^397,398^ Recent studies have demonstrated that strongly correlated materials can be engineered to replicate biological neuronal behavior, leveraging their nonlinear dynamical modulation of conductance with temperature or voltage such as metal–insulator transitions (MITs) manifested in binary and ternary vanadium oxides in response to external stimuli such as temperature (*T*_C_), electric fields, external pressure, and chemical doping.^8,386,399,400,309,401,402^ Indeed, just a few nonlinear dynamical memristive neuromorphic elements can replace thousands of MOSFETs in computing operations and provide a direct means of encoding efficient signal amplification.^403^

Recent analytical frameworks and compact models have begun to establish the foundational basis for how the edge-of-chaos expressed by biological neurons with sodium- and potassium ions can be emulated in physical materials leveraging nonlinear dynamical modulation of conductance as a function of state variables, which as shown in the rugged energy landscape of Figure 3 is abundant in vanadium oxides.^404,405^ Manifesting edge-of-chaos behavior to underpin self-sustaining oscillations, action potentials and bursting requires foremost the right kind of state variables expressing the right kind of nonlinear control over electrical conductance, as has been tuned by evolution over 400 million years in biological neurons. It is important that the set of state variables be reduced to only the minimal subset that are mutually independent, i.e. there should be no invertible function that maps any two state variables onto each other at all times. In analog or digital circuits, voltages across capacitors and currents through inductors are state variables. State variables of relevance usually describe the energy stored within a circuit element, capture its current state, and reliably predict its response to specific perturbations. A memristive element,^406^ which is critical to manifesting edge-of-chaos, has a variable resistance that depends on one or more internal degrees of freedom used to “store” information. The first solid-state memristors to be accurately described were based on ion migration (or migration of charged oxygen vacancies)^407^ where applied voltages shuttled ions back and forth in dielectric matrices such as TiO_2_,^408^ HfO_2_,^409^ or Ta_2_O_5_.^410^ The state variable therein is a measure of the position of the cloud of vacancies, e.g. width of the depletion region, in the device. When the power to this type of memristor was turned off, the vacancies remained frozen in place and the resistance state was retained. The diffusion constant for the vacancies is so low at room temperature that in theory the resistance of such systems would be stable for decades or even centuries, so this closely approximated a “nonvolatile memory” type of memristor. Such a system does not oscillate in the presence of a DC current or voltage bias, so it does not express EOC.

The next class of solid state memristors to be recognized were “electro-thermal” in nature, such as NbO_2_, VO_2_, LaCoO_3_, and *M*_*x*_V_2_O_5_ that exhibited nonlinear dynamical conductance modulation underpinned by strong electron correlation.^8,11,290,411–414^ For this class of memristor based on Peierls–Mott transitions (see Section 3), the material heats up by Joule self-heating when electrical power is applied, which in turn causes the electrical resistance of the material to decrease (or conductance to increase). This produces a feedback effect that causes the temperature, which is a thermodynamic state variable for the memristor, to increase rapidly, thus causing the conductance to increase rapidly^415,416^ (Figure 42a).

**Figure 42:**
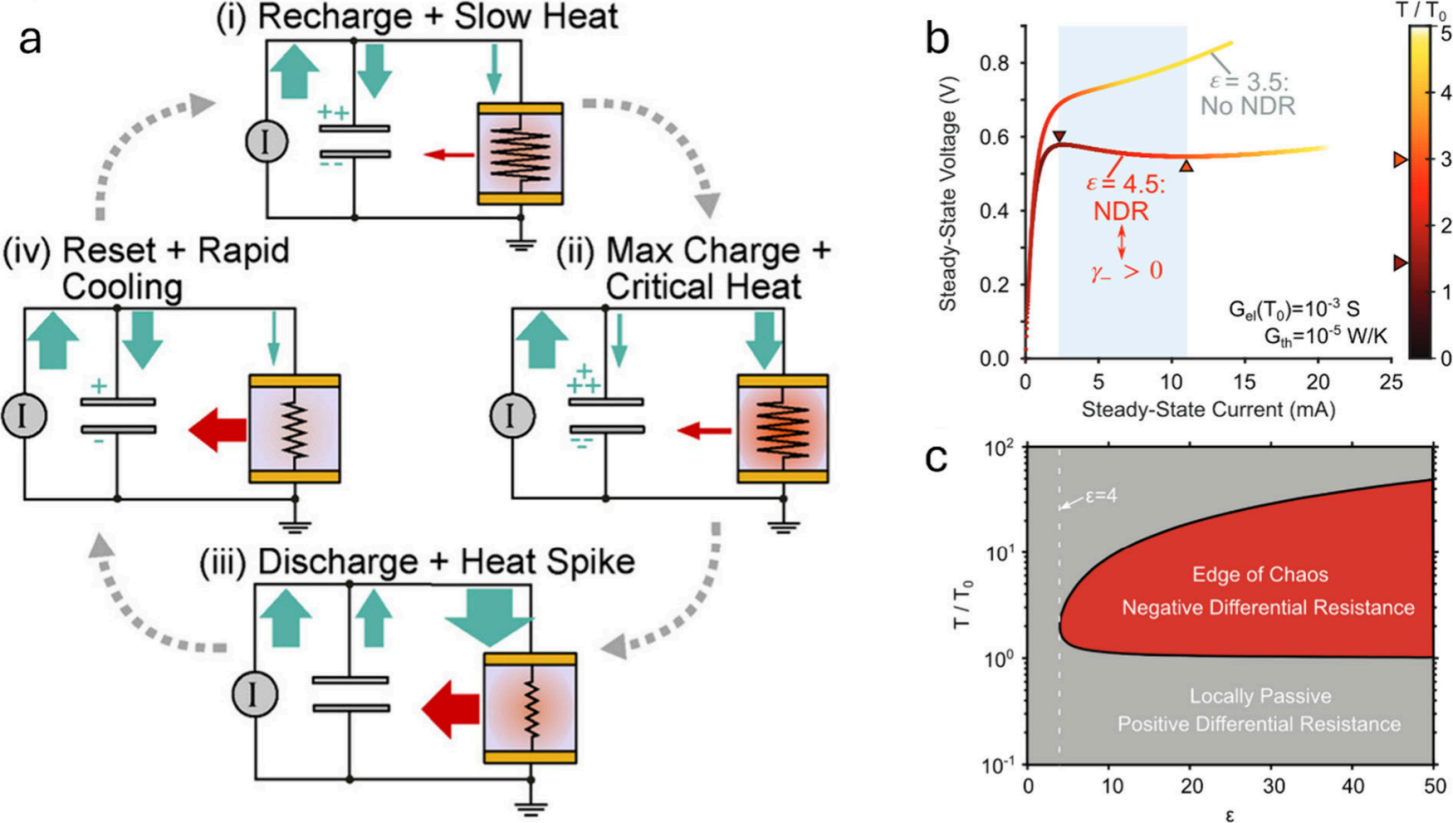
Electro-thermal oscillator operation and dynamics. (a) Four “phases” of a single electro-thermal oscillator cycle: (i) with the active element in the high-resistance state, current flows preferentially through the capacitor and increases its voltage, (ii) at a high capacitor voltage, sufficient current flows through the active element to heat it into its conductive state (iii) after the capacitor has discharged though the active element, cooling overtakes joule heating, and (iv) once the active element cools into its insulating state, the capacitor begins to recharge. (b) The presence of a negative differential resistance depends on the temperature dependence of the electrical and thermal conductances, with the most sensitive factor the ratio of the band gap energy to the ambient temperature at which the device will be operated, $\frac{\varepsilon }{kT_{0}}$ (c) The edge of chaos exists within a region of parameter space defined by the bandgap of the material *ε* and the internal temperature of the device driven by Joule heating T. Panel (a) reproduced from reference 416 under a CC BY license. Copyright 2025 Jardali et al. Panels (b) and (c) reproduced from reference 404 under a CC BY license. Copyright 2022 the Brown et al.

However, the heating of the memristor is balanced by “Newton’s Law of Cooling”,^417^ which states that the rate of heat flow out of a system by thermal conductance is proportional to the difference in temperature between the heated material and the surroundings. When the power is turned off, the memristor will cool down at some rate determined by the thermal conductance and the heat capacity of the system. This is a volatile or leaky “memory”. The time dependence, and thus the dynamics, of the memristor are determined by the conductance equation and a first order differential state equation:^404^

23 $$i=G_{\mathrm{el}}\left(T\right)v$$

24 $$\frac{dT}{dt}=\frac{1}{C_{\mathrm{th}}\left(T\right)}\left(iv-G_{\mathrm{th}}\left(T\right)\left(T-T_{0}\right)\right)$$

where *i* is the current, *v* is the voltage, *G*_el_ (*T*) is the temperature-dependent electronic conductance of the material, *T* is the internal temperature of the memristor, *T*_0_ is the ambient temperature, *C*_th_ (*T*) is the thermal capacitance (volumetric heat capacity), and *G*_th_ (*T*) is the thermal conductance. If the applied current for a current-controlled system is increased slowly enough or held constant, $\frac{dT}{dt}$ is close to zero and the system is essentially in a steady state that may be stable, unstable or “semistable”, i.e. EOC. For appropriate values of the electronic and thermal conductances (and to a lesser extent the thermal capacitance), the voltage vs current sweep can have a region in which the voltage decreases as the current is increased, i.e. a region of negative differential resistance (Figure 42b). This is the usual experimental signature of EOC. In combination with a parallel capacitor with its own state variable and differential state equation, which can be the intrinsic capacitance of the memristor itself, such a system is capable of oscillating (two coupled state equations are necessary for an oscillator) if the rates of heating and cooling are properly balanced, which may also be a signature of EOC if it is “controllable”. Figure 42a sketches the continual oscillation in response to an external voltage as current is split between a parallel capacitor and the active nonlinear dynamical element.

Neuron-emulative circuits leveraging the MIT in VO_2_ have been shown to emulate a large number of complex neuron-like behaviors such as spike frequency adaptation, accommodation, and threshold variability^418^ Achieving this level of complexity requires precise control over multiple functional attributes of electronic phase transitions, including transformation characteristics such as conductance switching magnitude, energy thresholds, heat dissipation, hysteresis, relaxation dynamics, and the number of accessible internal states, a subset of which are depicted in Figure 43a.^386,283,419–422^ These design rules are in turn derived from a mechanistic understanding of the interrelationship between atomistic, electronic, mesoscale, and interfacial structure and how changes in each give rise to conductivity transitions (Figure 43b).^299,413,419^

**Figure 43:**
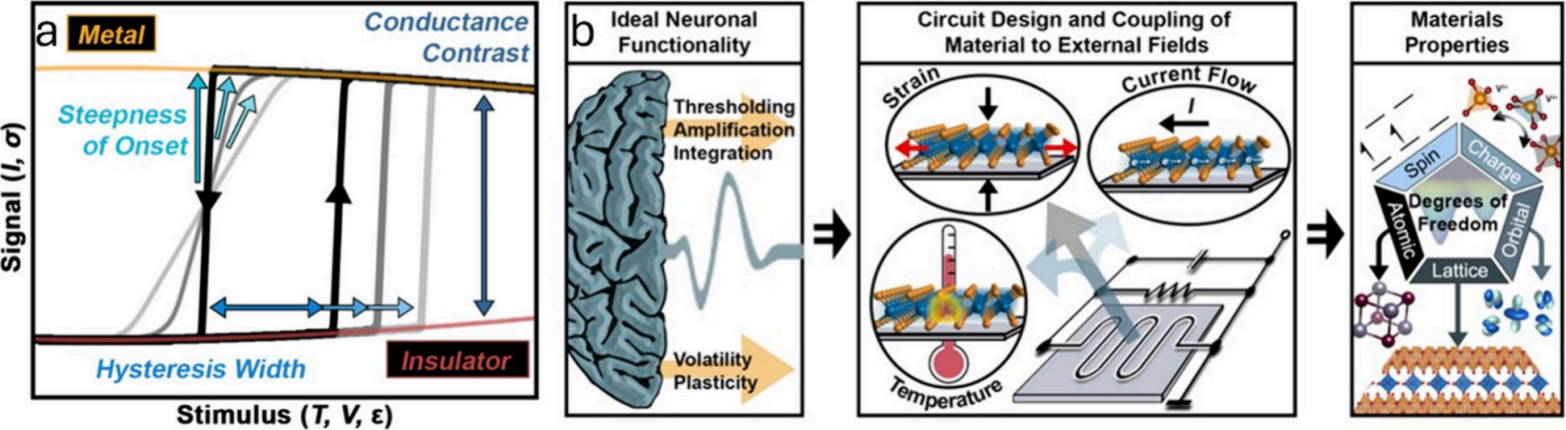
From compact models to material properties. (a) Transformation characteristics of metal–insulator transitions (MITs), which must be tuned in order to precisely control the properties of circuits emulating the function of neurons and synapses. (b) Designing physical neurons and synapses requires identifying desired circuit behaviors, deciphering modes of field coupling to material properties, and mapping to structure and composition of materials exhibiting strong coupling of spin, charge, orbital, atomic, and lattice degrees of freedom.

The key aspect of vanadium oxides that renders them viable and versatile electrothermal neurons is their strong temperature-dependent conductance,^300,423,424^ manifested in metal–insulator transitions, superconductivity, and a host of electronic phase transformations examined in Section 3 above. In general, for a physical signal to be able to act as a state variable capable of EOC and to exhibit dynamical complexity, several minimum criteria must be met: (i) the state variable needs to have a power-off negative feedback mechanism: The −*G*_th_ (*T*)­(*T* – *T*_0_) term in eq 24 recools the system when the power is OFF; (ii) the state variable needs to have a power-on positive feedback mechanism: The +*iv* term in eq 24 heats the system when the power is ON; (iii) the positive feedback mechanism needs to be controlled somehow by current, voltage, or power, e.g., +*iv* term in eq 24; (iv) the dependence of electrical conductivity on the state variable needs to be substantially nonlinear based on eq 23. As sketched in Figure 42a, for vanadium oxide actively exhibiting electronic phase transformations, as additional power is input into the device, the active element undergoes Joule heating, thereby increasing the temperature, and increasing the conductivity based on its abrupt phase transformation. If the power to the device is turned off, the temperature returns to the ambient temperature (*T*_0_) due to Newton’s Law of Cooling. More power generates greater temperature increases and the power input provides a means of positive feedback. For appropriate extents of nonlinearity manifested in vanadium oxides, such as recently demonstrated for both β′-Cu_*x*_V_2_O_5_ and ε′-Cu_*x*_V_2_O_5_, an EOC regime can be identified where oscillations can be sustained.^365,366^

As discussed in the previous section, MITs in vanadium oxides that enable EOC behavior are complex phenomena, arising from competition between electron and/or spin localization and delocalization. These transitions are typically first-order and are often accompanied by crystallographic distortions that lower the material’s symmetry below the transition temperature (*T*_C_). In correlated electron materials, this competition between electronic states arises from the strong interactions between spin, lattice, charge, and orbital degree of freedom sketched in Figure 43b. These interactions are very sensitive to application of external stimuli, where small perturbations can disrupt their delicate balance, transform a material along the rugged free energy landscape, and trigger phase transitions. External fields such as temperature, voltage, and pressure are commonly used to induce transitions in correlated electronic systems. Continued development of vanadium oxide neuromorphic active materials depends closely on improving our mechanistic understanding of these electronic structure instabilities and the exploration of the vanadium oxide materials design space to discover new candidates. Section 3 addresses the former subject, while Section 5 below describes a set of strategies for accessing new, metastable structures and compositions.

## 5 Topochemical Transformations of Vanadium Oxides

Traditional ceramic/solid-state synthesis provides a large amount of energy; as such, a system is efficiently able to sample the free energy landscape without being trapped in local minima, thereby often finding efficient ways of converging on equilibrium phases. In contrast, synthetic routes to carefully “deposit” a system within a local minimum wherein metastable structural forms exhibiting unusual chemical bonding motifs can be stabilized requires careful consideration of starting atomic arrangements and reaction trajectories.^10^ Owing to this difficulty, the possible range of metastable phase space remains underexplored and underutilized.^425^ *Chimie douce* or soft chemistry/topochemical methods have the potential to carefully “deposit” the material in a local minimum based on well-defined crystallographic relationships with precursor compounds and have found extensive use in the preparation of metastable compounds.

Topochemistry was first defined by Kohlschütter to describe the reactivity of species held in fixed orientations by a solid lattice.^426^ In conjunction with the related concept of topotaxy (the preservation of crystallographic relationships through solid-state reactions), topochemistry describes solid-state reactions which typically involve small atomic displacements and preserve structural elements of the reactants in the products.^427^ This use of the term topochemistry as related to the compounds under consideration—preservation of a host framework’s bond structure during a solid-state reaction—was introduced several decades ago by pioneers such as Livage and Figlarz, and it is in this sense that we use the term in the discussion that follows. Their innovative approach established new preparative routes in solid-state chemistry, enabling researchers to explore previously uncharted regimes of chemical reactivity, which enabled access to unprecedented structural diversity.^428^ Indeed, these topotactic reactions utilize low-temperature, kinetically controlled processes that involve the substitution of a highly mobile guest species (typically ions or molecules) within a host compound that contains a rigid framework with vacant interstitial sites (typically layered or tunneled structures) with minimal atomic rearrangement of the host lattice. Once inserted, these guest ions can be removed or even exchanged with relatively minor disruption of bonding framework of the host lattice. Whittingham coupled topochemical reactions with electrochemistry enabling the discovery of ion-inserted ternary phases in molybdenum trioxide, titanium disulfide, and vanadium pentoxide, which set the stage for modern Li-ion batteries.^53^ Concurrently, Thomas played a crucial role by exploring topochemical control in both organic and inorganic solids,^429^ thereby establishing a foundation for subsequent advances in intercalation chemistry. Whittingham and co-workers spearheaded the efforts into investigating the topochemical lithiation and sodiation of V_2_O_5_ for electrochemical applications,^53,163^ whereas investigations led by Murphy and co-workers provided key insights into the thermodynamics of (de)­lithiation of VO_2_, V_3_O_7_, V_4_O_9_, and V_6_O_13_.^430^ The specific structural motifs of vanadium oxides reviewed above such as abundant interstitial sites manifests a rich diversity of solid-state transformations and provides access through topochemical and electrochemical means to multinary vanadium oxides, whose distinctive electronic and structural properties have since become a focal point of contemporary research.

In this section we seek to discuss the topochemical and electrochemical transformations of single-crystalline and polycrystalline binary vanadium oxides. We will first outline experimental considerations applicable to the design of topochemical reactions. We will explore these concepts in more detail by way of specific example systems, through the lens of their crystal structures, the available vacant sites for guest ion/molecule (de)­insertion, and the resulting changes in crystal framework.

### 5.1 Experimental Considerations for Topochemical Reactions

The key challenge in a topochemical single-crystal-to-single-crystal transformation is maintaining the integrity of the crystals. Ion insertion inevitably distorts the host material lattice, and accumulation of chemical potential and strain gradients may cause plastic deformation and disintegration of the crystal.^431^ This problem is exacerbated in systems where ion insertion induces sharply discontinuous phase transformations involving significant bond rearrangement, as in lithiation of α-V_2_O_5_ (see Section 5.2.1 below).^432^ Three factors influencing the success of a single-crystal topochemical transformation in this regard are thus (1) whether the host structure distorts continuously upon insertion or removal of cations; (2) whether the insertion-induced transformation takes place without collapse of the host lattice; and (3) whether the kinetics of the reaction are slow enough that deleterious chemical potential and strain gradients are minimized.^36^ Each of these depends on the particular choice of host material, guest ion to be inserted or removed, redox-active reagent, solvent, temperature, etc. Establishing kinetic control over a single-crystal topochemical synthesis involves searching for optimal conditions within a large space of reaction parameters.

Topochemical transformation kinetics can be limited either by surface reactions or solid-state diffusion.^205,433^ For instance, Fraggedakis et al., derived a generalizable scaling law wherein in a diffusion-limited regime, when the distribution of inserted Li-ions lags behind surface reaction kinetics, a “core–shell” morphology is observed, whereas in a surface-reaction-limited regime, “intercalation waves” deriving from a limited number of surface nucleation sites propagate through the crystalline lattice of the particle.^205^ Factors such as reaction time and concentration of the topochemical or electrochemical reagent control the extent and driving force for diffusion by determining the chemical flux at surfaces. The host lattice’s structural flexibility and dimensionality plays a paramount role in determining its ability to accommodate ions without undergoing irreversible changes. Finally, the surface orientations and topologies play a pivotal role in governing whether the incoming ion flux is oriented along specific insertion directions and if there exist defective sites such as with coordinative unsaturation or vacancies that are particularly amenable to ion insertion at low overpotentials and that can effectively initiate nucleation of ion insertion reactions. Careful tuning of each of these conditions is essential for achieving controlled and efficient topochemical transformations.

#### 5.1.1 Host Material

The topochemical reactions which we will discuss in vanadium oxides involve the long-range migration of ions through networks of interconnected vacant interstitial sites within the tunnel or layered structures outlined in Section 1.2. The dimensionality of the interstitial space plays a role in the kinetics of ion insertion. The intercalation of ions within layered compounds is frequently described using the staging model,^434–436^ which breaks down structures by assigning an index. Lower staging indices indicating higher guest-ion concentrations within the host lattice (Figure 44). Stage 1 features uniform, alternate guest and host layers, whereas Stage 2 involves a guest layer separated by two distinct host layers. Higher staging indices, such as 3 and above, correspond to a lower degree of intercalation and are likely to involve interior host layers that are not adjacent to the intercalated layers. The intercalation process requires energy to separate the host layers and create vacant sites for the intercalant as well as a reduction in entropy because of the eventual ordered arrangement of the guest species. This becomes more difficult when utilizing guest ions with larger ionic radii, which requires accommodation through greater lattice dilatation.^437^

**Figure 44:**
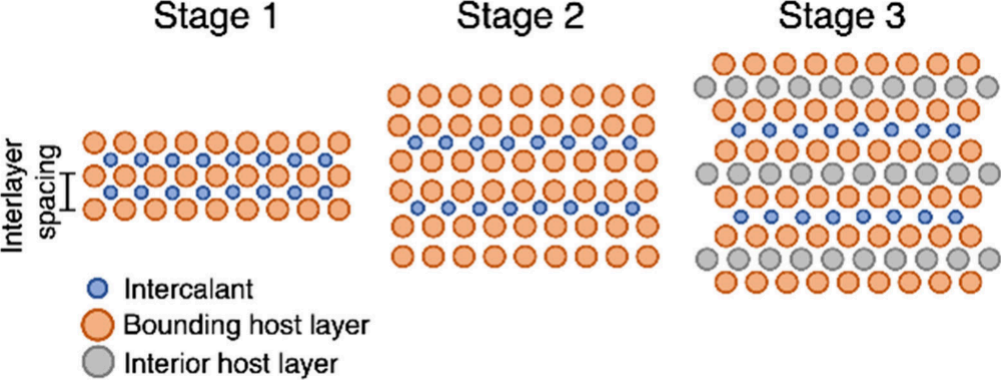
A schematic representing the process of staging that occurs when ions are intercalated within layered host compounds. Stage 1 compounds (left) correspond to alternating layers of the bounding host (orange) and intercalant (blue). Stage 2 describes two consecutive layers of the host compound followed by a layer of the intercalant. Higher order staging models, like Stage 3, involve a lower degree of intercalation and involve layers or intercalants that are separated by different layers within the host like bounding layers and interior layers (gray). This figure was reproduced with permission from reference 434. Copyright 2019 Wiley.

In tunnel-structured oxide frameworks, the ion insertion process is similarly governed by a delicate balance between structural deformation and chemical driving forces. Rather than requiring expansive layer separation as in lamellar structures, tunneled frameworks accommodate guest ions within rigid, one-dimensional channels. While this minimizes large-scale lattice dilatation, it introduces steric and electrostatic constraints within confined diffusion pathways. Enthalpic stabilization is limited by the accessible coordination number of the guest cation with limited local framework distortions, while entropic stabilization is governed by the relatively constrained positional disorder within the tunnels.^438–440^ As with layered materials, ion insertion into tunnel-structured oxides navigate the free energy landscape through kinetically controlled topochemical pathways.

Insertion or intercalation within tunnel-structured or layered hosts is thermodynamically driven by the reduction of a transition metal center to accommodate the charge of inserted species. eq 25 represents a generalized, redox-mediated topochemical insertion or removal reaction involving a vanadium oxide:

25 $$xA+V^{2m+}O_{m}^{2-}↔A_{x}^{n+}V^{\left[2m-xn\right]+}O_{m}^{2-}$$

When the inserting species (*A*) is a small, highly electropositive atom (like lithium), the host vanadium exists in a high oxidation state, and the framework anion is both small and strongly electronegative (as in the case of oxygen), the redox insertion reaction has a strong thermodynamic driving force, which indeed is harnessed in battery electrode materials. The thermodynamics of the reaction reflects the specific redox half-reactions involved, which makes oxides particularly suitable as high-voltage cathode materials. These redox-mediated Coulombic interactions lower the energetic cost of accommodating inserted ions, enabling reversible ion storage even in the case of high structural rigidity.^434,436,441,442^

#### 5.1.2 Guest Ions and Reagents

A variety of cations monovalent, divalent, and polyvalent ions can be accommodated within interstitial sites of vanadium oxides (Figure 45). A primary consideration governing the kinetics and thermodynamics (and ultimately, the success) of a topochemical reaction is the charge density—defined as the ratio of ionic charge to radius—of the chosen ion to be inserted or removed.^163^ Considering monovalent ions, lithium ions with a small Shannon ionic radius^68^ of 0.59–0.76 Å (for coordination numbers typical in vanadium oxides^32^) can be readily inserted within a variety of vanadium oxide frameworks. Li ions can be rapidly diffused between layers with a broad range of interlayer spacings as well as along quasi-one-dimensional tunnels with only relatively minor structural perturbations of the host framework.^26,27,36^ In contrast, larger monovalent ions such as sodium (1.02–1.18 Å) and potassium (1.46–1.51 Å) require larger interstitial sites; in light of the lower solid-state diffusion coefficients of Na-ions, topochemical sodiation typically necessitates higher temperatures and longer times. The larger dimensions of Na-ions furthermore result in extensive sliding of layers and insertion-induced structural transformations;^32^ potassium is inserted in relatively low stoichiometries and engenders substantial lattice expansion.^35^

**Figure 45:**
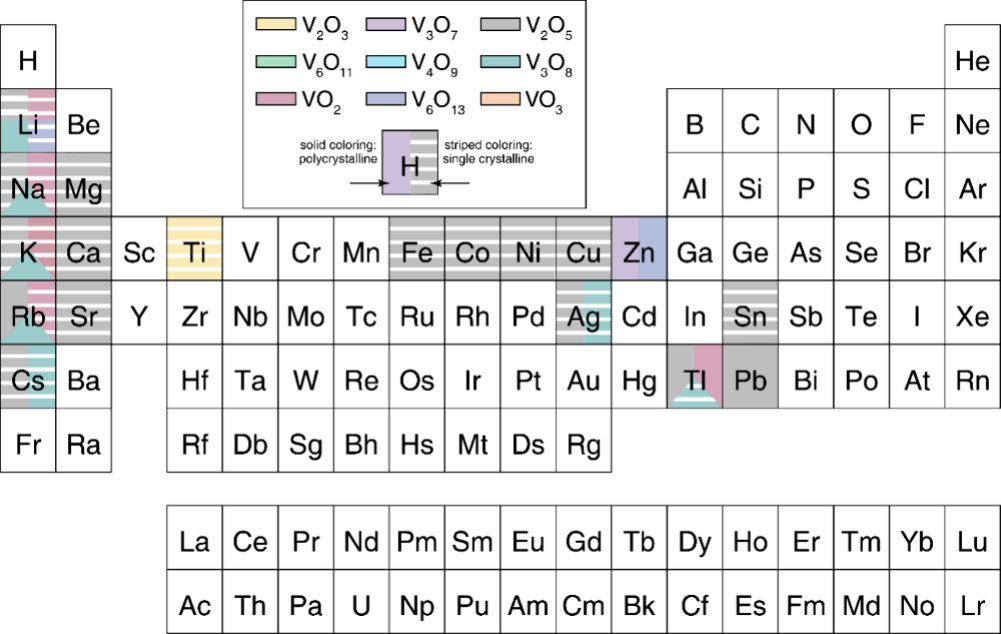
A periodic table indicating the identity of ions that have been inserted within vanadium oxides. The dashed white lines represent instances where the substitution was performed on single crystals. Solid shaded elements indicate ion doping within polycrystalline samples. For example, dark blue shading for Li and Zn indicates that these cations have been successfully intercalated within single crystalline V_6_O_13_ (striped) and polycrystalline V_6_O_13_ (solid), respectively.

Divalent cations, such as magnesium and calcium can also be inserted into vanadium oxide frameworks. Although magnesium has a small ionic radius of 0.66–0.72 Å, its divalent oxidation state leads to stronger electrostatic interactions with the anion lattice and indeed “hard” cations are generally more challenging to insert.^443,444^ Calcium, with a larger radius of 1.00–1.12 Å, is “softer” but further induces lattice distortions.

A determinative factor for both topochemical and electrochemical reactions is the competition between redox insertion and conversion or corrosion processes, which requires precise control of the effective potential windows and reaction kinetics to preserve structural integrity while enabling accommodation of controllable stoichiometries of inserted ions. In both topochemical and electrochemical ion insertion, elementary steps involve (i) ions diffusing to the surface of the host framework, (ii) adsorption of ions onto surfaces (in the case of electroabsorption, ions are bound at the Stern layer extending out to the Debye length constituting a somewhat mobile layer of adsorbed species activating capacitive and pseudocapacitive processes^445,446^ ); (iii) ion diffusion to a site where insertion can be initiated; (iv) desolvation and insertion of the ion within the host framework, which involves a concomitant redox event; (v) solid-state diffusion through layers or tunnels along interstitial sites;^447^ and (vi) either continuous incorporation of ions such as through a staging process or insertion-induced phase transformations.^54,448^ Electrochemical ion insertion potentially affords greater control over kinetics and stoichiometries of ion insertion based on selection of the applied potential.

Murphy and co-workers established a redox equivalent scale shown in Figure 46 that provides a chemical equivalent of specific reagents that can be used to drive redox intercalation or deintercalation.^449^ For instance, the low reduction potential (≈1 V vs. Li/Li^+^) of *n*-butyllithium (C_4_H_9_Li) allows it to lithiate TiS_2_ in heptane according to eq 26. Lithium can then be removed from the LiTiS_2_ product using I_2_ in acetonitrile (eq 27) or 2,3-dichloro-5,6-dicyano-1,4-benzoquinone (DDQ), owing to the high reduction potentials of their respective products.^428^

26 $$\mathrm{Ti}S_{2}\left(s\right)+C_{4}H_{9}\mathrm{Li}\left(\mathrm{heptane}\right)\to \mathrm{LiTi}S_{2}\left(s\right)+\frac{1}{2}C_{8}H_{18}\left(\mathrm{hexane}\right)$$

27 $$\mathrm{LiTi}S_{2}\left(s\right)+\frac{1}{2}I_{2}\left(\mathrm{MeCN}\right)\to \mathrm{Ti}S_{2}\left(s\right)+\mathrm{LiI}\left(\mathrm{MeCN}\right)$$

While benzophenone readily forms its characteristic ketyl radical anion upon reduction, the electron transfer from LiTiS_2_ to bzph and subsequent delithiation (eq 28) are not spontaneous due the benzophenyl radical’s (bzph^•–^) low reduction potential

28 $$\mathrm{LiTi}S_{2}\left(s\right)+\mathrm{bzph}\left(CH_{3}\mathrm{CN}\right)\to ̷\mathrm{Ti}S_{2}\left(s\right)+Li^{+}\left(CH_{3}\mathrm{CN}\right)+\mathrm{bzp}h^{•-}\left(CH_{3}\mathrm{CN}\right)$$

**Figure 46:**
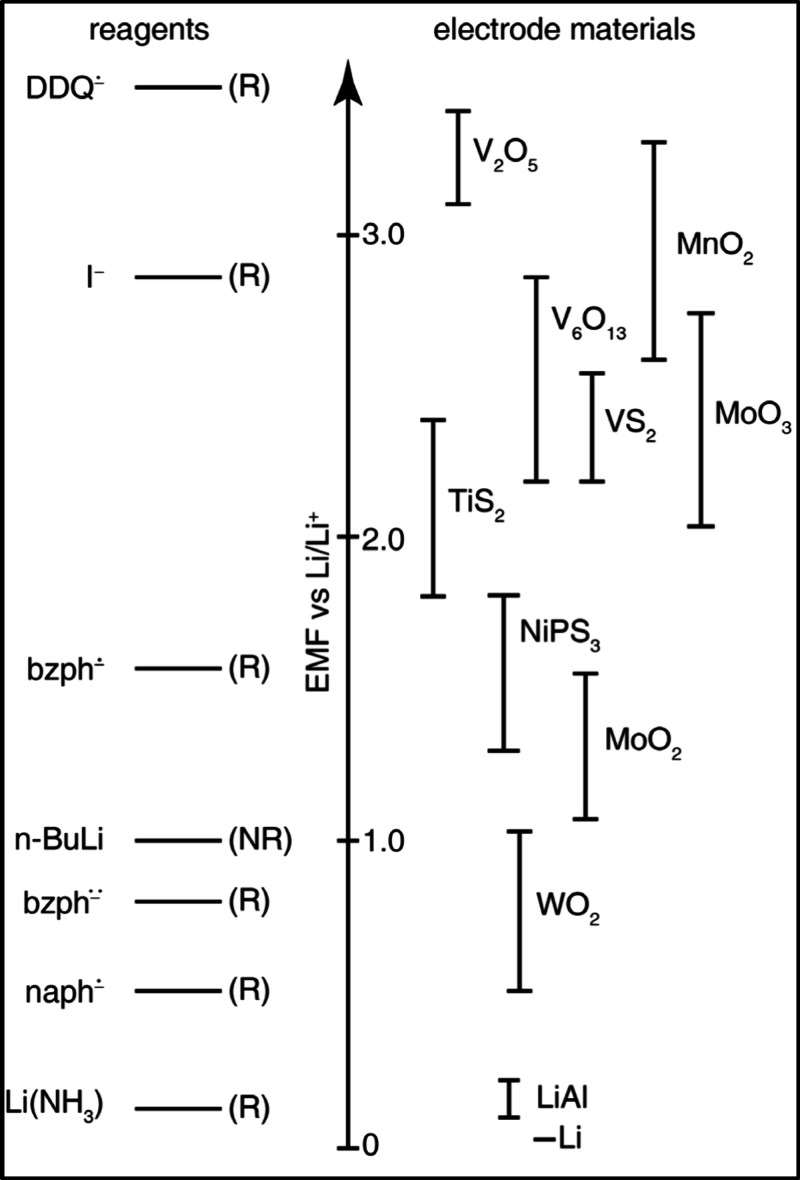
Potentials of a variety of reagents (left) and electrode materials (right) for modeling cell reactions relative to Li/Li^+^. It is important to note that the potential of each reagent varies depending upon the solvent, concentrations, and electrolyte utilized. (R) denotes reversible reagents while (NR) refers to irreversible reagents. The abbreviations for the reagents are as follows: DDQ is 2,3-dichloro-4,5dicyanobenzoquinone, bzph is benzophenone, naph is napthalene, and *n*-BuLi is *n*-butyllithium. This figure was reproduced from reference 449. Copyright 1979 Springer Nature.

In principle, reagents that are closely matched with a host lattice’s reduction potential allow reversibility, i.e. the same reagent can be used to insert or remove an ion by the application of Le Chatelier’s principle. For instance, lithium iodide will spontaneously insert Li^+^ into V_2_O_5_ (eq 29), and this reaction can be reversed by the application of large concentrations of I_2_.

29 $$V_{2}O_{5}\left(s\right)+x\mathrm{LiI}\left(\mathrm{MeCN}\right)↔{\mathrm{Li}}_{x}V_{2}O_{5}\left(s\right)+\frac{x}{2}I_{2}\left(\mathrm{MeCN}\right)$$

Continued action of this sort of dynamic equilibrium allows for the gradual homogenization of initially inhomogeneous ion concentration, while the low overpotential of the redox-matched reaction limits unwanted side-reactions.^450^ By contrast, the stability of the octane produced by *n*-butyllithium precludes the reverse reaction and more readily leads to the accumulation of composition inhomogeneities and chemical potential gradients the implications of which are discussed in further detail in Section 7. Similar considerations pertain to the use of irreversible oxidizers, and maintaining controlled reaction rates under mild oxidizing conditions is essential to avoid structural collapse. Oxidizing agents commonly used to deintercalate *M*_*x*_V_2_O_5_ materials follow the order of decreasing of oxidation strength: NO_2_BF_4_ > Na_2_S_2_O_8_ > NOBF_4_ > Br_2_ > I_2_.^451^

#### 5.1.3 Solvent

Solvents play a crucial role in influencing topochemical reactions owing in part to the thermodynamics of guest ion desolvation. Loss of surrounding solvent molecules must occur order for ionic species to incorporate into the host lattice. The strength of guest species solvation, solvent molecule size, and coordination characteristics not only influence the structure of the solvation shell but also determine whether desolvation is required at the electrode interface or whether solvent molecule cointercalation occurs. This, in turn, impacts the reaction kinetics, interfacial resistance, and structural evolution of the electrode.^452^ Two illustrative examples from recent studies highlight how solvent choice modulates these interactions.

In the first case, sodium ions in diglyme-based electrolytes exhibit cointercalation into graphite, bypassing the typical desolvation step required in conventional carbonate solvent systems. Diglyme effectively stabilizes the Na^+^ ion and acts as an electrostatic shield, reducing the energy barrier for charge transfer and enabling fast, reversible insertion of solvated Na^+^ into the graphite lattice.^453^

In a second system, TiS_2_ electrodes demonstrate starkly different electrochemical behavior depending on the solvent used. When diglyme is the solvent, cointercalation of Na+ occurs, accompanied by notable interlayer expansion.^452,454^ In contrast, traditional carbonate solvents suppress solvent cointercalation due to the stronger binding of the solvent shell and higher desolvation energy, resulting in conventional ion insertion without structural swelling. These results underscore the dynamic role solvents play not only in stabilizing ions but also in dictating whether and how they can enter host structures.

The choice of solvent can also significantly alter the properties of the host material by modifying surface and interlayer energetics and thereby enhance or inhibit guest species accommodation. Liu and co-workers discerned that different solvents modify the effective internal pressure (*P*_eff_) within the host through variations in surface energy and surface tension. These solvent–host interactions directly impact intercalation kinetics as demonstrated by the varying reaction rates of ferrocene insertion into VOPO_4_•2H_2_O across different solvent environments.^455^ The complexity of these solvent effects extends beyond simple parameters like desolvation energies or redox potentials, highlighting the need for comprehensive understanding of solvent–host–guest interaction networks.

#### 5.1.4 Pillaring and Preintercalation to Enhance Insertion Selectivity

Preintercalation, or “pillaring,” involves intentionally introducing guest cations into electrode host structures prior to the insertion of the desired ion. Pillaring is particularly useful for host structures with well differentiated distinct crystallographic sites for ion insertion. Specifically, preintercalation can be used as a means of expanding crystal lattice frameworks such as to mitigate spatially constrained bottle-necks that impede ion diffusion and to thereby expand the transition states.^443,456–458^ In more rigid hosts, preintercalation can inadvertently block favorable diffusion pathways or promote framework anion migration, potentially resulting in structural collapse by stabilizing under-coordinated transition sites. Several examples where a straightforward chemical preintercalation strategy grants electrode materials enhanced structural stability are discussed in Section 5.2.3.

#### 5.1.5 Tracking Topochemical Transformations with Single-Crystal Diffraction

Single-crystal X-ray diffraction (SCXRD) has emerged as a pivotal tool for elucidating the atomic-scale mechanisms governing topochemical transformations in energy storage materials. Unlike conventional powder X-ray diffraction (PXRD), which averages signals across polycrystalline grains and relies on sparse reflections (one score or a few scores of indexed reflections), SCXRD leverages thousands of reflections to achieve angstrom-level spatial resolution. Moreover, the crystal structure solution also provides high-resolution three-dimensional electron density maps providing insights into the sequence of interstitial sites occupied upon topochemical ion insertion/deinsertion, a high-resolution view of framework distortions induced as a result of ion insertion; and mapping of ion diffusion pathways—aspects often obscured in ensemble studies. However, crystallographic mosaicity, arising from misoriented domains, complicates analysis, particularly in topochemically modified systems where kinetic inhomogeneities (e.g., nonuniform cation insertion and strain gradients) generate distinct domains. To mitigate this, studies prioritize single-domain crystals with low mosaicity, which yield coherent snapshots of kinetically trapped intermediates. These structure solutions reveal partial occupancies and dynamic averaging effects of the inserted ion, which provide insight into metastable configurations accessed in electrochemical processes. While underexplored, diffuse scattering holds promise for revealing local structure correlations in ion disordered samples.

Complementing SCXRD, first-principles DFT calculations can be used to rationalize site-filling preferences and local perturbations observed in experiment. This synergy between SCXRD and computational modeling bridges atomic-scale observations with macroscopic electrochemical performance, clarifying the origins of phenomena such as voltage hysteresis and ion mobility barriers. By mapping kinetic bottlenecks (e.g., constrained sites ions need to hope through between sites) and by exploring relative energetics of diffusion pathways through molecular dynamics simulations, these insights can guide the design of robust electrodes with enhanced ionic conductivity and structural resilience. As such, SCXRD-driven studies not only advance our fundamental understanding of energy storage mechanisms but also provide a blueprint for optimizing next-generation battery technologies.

### 5.2 The Rugged Free Energy Landscape of Binary and Multinary V_2_O_5_ Phases

A huge variety of materials feature a V_2_O_5_ framework composition, including at least six binary polymorphs^33,36,47,459,460^ and countless ternary M_*x*_V_2_O_5_ and multinary phases (which inherit the nickname “bronzes” from the analogous family of tungstates that exhibit a bronze-like metallic luster). The binary materials contain vanadium in its maximum nominal oxidation state, V^5+^, charge-balanced by a low vanadium/oxygen stoichiometric ratio. The high density of vanadium vacancies compared to their shared VO parent structure (see Figure 4 and related discussion in Section 1.2) generates one-dimensional (tunnel) or two-dimensional (planar) networks of interconnected interstitial sites. The open nature of V_2_O_5_ structures facilitates the incorporation of relatively mobile guest ion species, while strong V^5+^–O^2–^ interactions stabilize the host lattice against collapse. V_2_O_5_ is indeed an attractive insertion host because of its multiple accessible redox couples and its limited proclivity for oxygen evolution.^461,462^ Migration of mobile guest ions between sites is dependent on the geometry and connectivity of interstitial site networks, which vary between framework structures. This endows each polymorph with a unique chemistry with respect to a given guest species. By way of example, Table 3 summarizes the sensitivity of Li- and Mg-ion diffusion kinetics to the sequence of coordination geometries adopted during migration through several host structures. The diffusion energy barrier of the hard Mg^2+^ ion is lowest for the uniform coordination trajectory offered by λ-V_2_O_5_, while the softer Li^+^ ion shows much less preference. Such considerations highlight the value of V_2_O_5_’s structural diversity for engendering desired physical and chemical properties.

**Table 3:** Calculated Open-Circuit Voltages, Energies above the Thermodynamic Hull, Diffusion Energy Barriers, and Coordination Number Sequence along Diffusion Trajectories for Mg and Li in Several V_2_O_5_ Polymorphs^10,443^ ^a^

	OCV-Li (V)	OCV-Mg (V)	E_hull_-V_2_O_5_ (meV)	E_hull_-Li_***x***_V_2_O_5_ (meV)	E_hull_-Mg_***x***_V_2_O_5_ (meV)	Li-ion Diffusion barrier (eV)	Mg-ion diffusion barrier (eV)	coordination number sequence during migration
**α-V**_**2**_ **O**_**5**_	3.35	2.59	0	45	118	0.11–0.16	1.15–1.23	8→3→8
**γ′-V**_**2**_ **O**_**5**_	3.44	2.73	6	27	50	0.18–0.15	0.71–1.16	4→3→5→3→4
**ζ-V**_**2**_ **O**_**5**_	3.71	3.28	24	23	–17	0.13–0.14	0.62–0.86	4→3→5→3→4
**ρ′-V**_**2**_ **O**_**5**_	3.74	2.86	30	38	85	0.07–0.07	0.28–0.46	4→3→5→3→4
**δ′-V**_**2**_ **O**_**5**_	3.59	2.80	32	53	149	0.07–0.19	0.54–1.07	4→3→5→3→4
**λ-V**_**2**_ **O**_**5**_	3.72	2.92	32	47	83	0.10–0.11	0.21–0.24	4→4→4→4→4

a Note that λ-V_2_O_5_ here refers to ε′-V_2_O_5_ in the original work.

*Chimie douce* methods, as discussed in the introduction to this section, are crucially important for the preparation of metastable V_2_O_5_ polymorphs. A typical synthesis strategy begins with the preparation of a thermodynamically stable ternary *M*_*x*_V_2_O_5_ bronze by direct solid-state, sol–gel, or solution-phase reaction between the stable α-V_2_O_5_ polymorph and a guest ion *M*. Incorporation of the *M* ion templates the V_2_O_5_ structure, with linear tunnels preferred by small ions at low concentrations and planar interstices between compact double-layered slabs by large ions at high concentrations. An oxidative topochemical reaction then removes the templating ion while preserving the host structure to produce the empty metastable polymorph. Figure 47 schematically depicts the sequences of solution-phase, solid-state, and topochemical reactions used to synthesize single-layered γ′-V_2_O_5_, double-layered λ-V_2_O_5_, and tunnel-structured ζ-V_2_O_5_. The broad range of polymorphs accessible by facile topochemical methods makes vanadium pentoxides a distinctive platform for decoupling composition from structure, by dint of its rugged energy landscape as discussed more generally in Section 1.1. In this section, we discuss synthetic strategies starting from thermodynamically stable α-V_2_O_5_ to metastable phases such as γ′-, ζ-, and λ-V_2_O_5_ based on topochemical extraction of cations (Li^+^, Ag^+^, Cu^+^) from M_*x*_V_2_O_5_ bronzes discussed above, and the subsequent ion exchange reactions their open framework structures enable.

**Figure 47:**
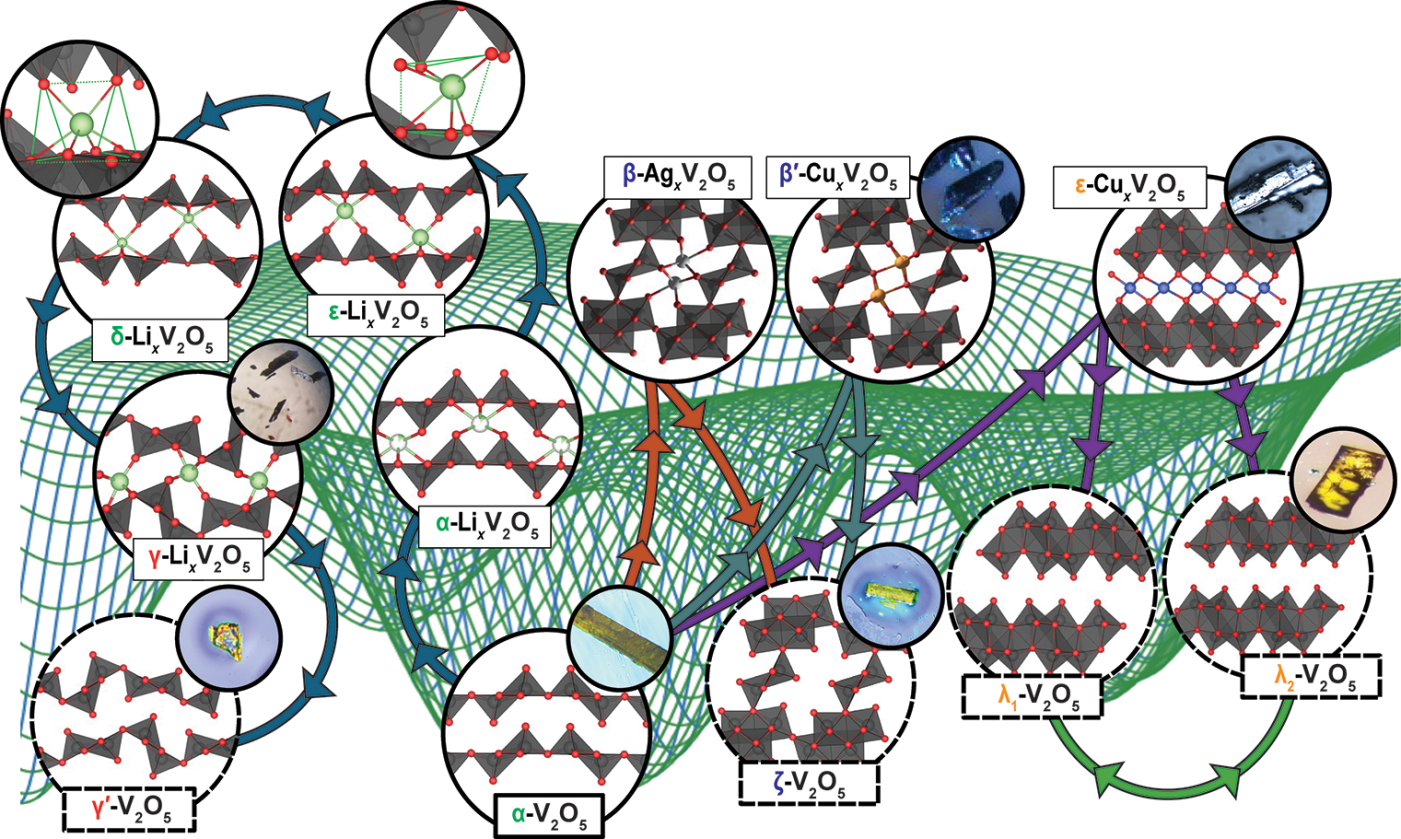
A rugged energy landscape showing several topochemically accessible phases within the V_2_O_5_ phase space. The crystal structure of each phase is depicted in the larger circles. Solid borders indicate thermodynamically stable phases, whereas dashed lines indicate metastable phases. Insets depict optical images of single crystals of each phase. The arrows represent reactions to synthesize each phase starting from α-V_2_O_5_, while Greek letter color indicates V_2_O_5_ framework type: green for single-layered, red for puckered single-layered, blue for tunnel, orange for double-layered host structures. The blue arrows indicate the lithiation of α-V_2_O_5_, which first yields α-Li_*x*_V_2_O_5_ and upon further lithium intercalation results in phase transformations to ε-Li_*x*_V_2_O_5_, δ-Li_*x*_V_2_O_5_, and γ-Li_*x*_V_2_O_5_. Magnified views of the ε-Li_*x*_V_2_O_5_ and δ-Li_*x*_V_2_O_5_ structures shown in the insets illustrate the shearing of the layers and the differences in the local Li-ion coordination environment (also shown in Figure 48f). Delithiation results in the empty γ′-V_2_O_5_ polymorph. Orange and green arrows indicate two separate pathways to achieve ζ-V_2_O_5_, which involve the successive insertion and removal of Ag and Cu, respectively. The purple arrows depict the cupration of α-V_2_O_5_ to obtain ε-Cu_*x*_V_2_O_5_. Decupration yields double-layered λ-V_2_O_5_.

#### 5.2.1 The Evolving Phases of Lithiated α-V_2_O_5_

In addition to its role as the starting point for metastable phase synthesis, α-V_2_O_5_ itself accommodates several intercalant species via chemical or electrochemical insertion. By far the best studied intercalant is lithium, owing to V_2_O_5_’s promise as a lithium-ion battery cathode material. In α-V_2_O_5_, inserted lithium migrates through the interlayer space along zigzag edge-shared VO_5_ chains extended along *b.* The host lattice distorts with continued lithiation as depicted in Figure 48a due lithium’s uncharacteristically large 8-coordinate site and the flexibility of the corner-shared V–O–V bonds along *a*, eventually accumulating in a series of structure transitions. In the initial solid-solution, α-Li_*x*_V_2_O_5_ (0 < *x* < 0.13, Figure 48b,c), lithium inserts into the pristine α-phase structure without significantly disrupting its layered framework. In this phase, vanadium atoms are coordinated by five oxygen atoms forming VO_5_ square pyramids, with an apical V=O bond length of approximately 1.577 Å and equatorial V–O bonds ranging from ∼1.779 Å to ∼2.017 Å. The V–O–V bond angles are about 125°, maintaining a relatively undistorted layered arrangement.^432,463,432,464^

**Figure 48:**
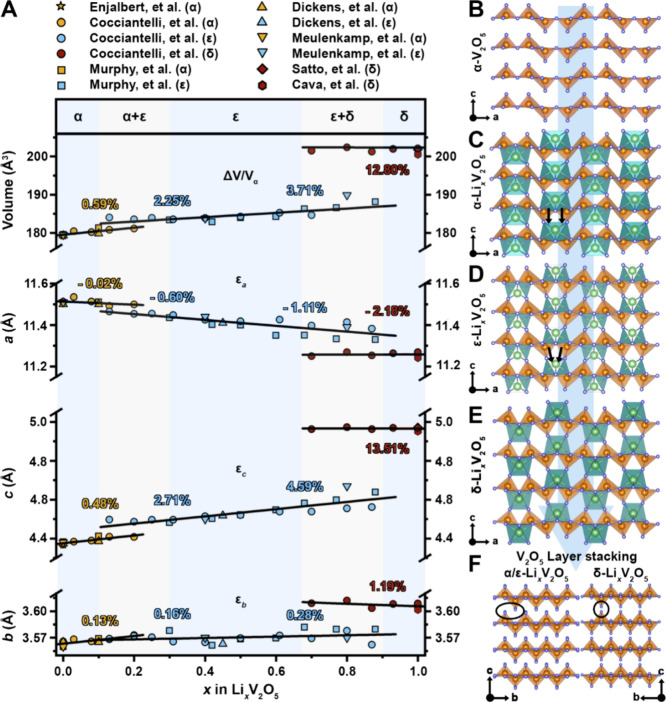
(a) Li_*x*_V_2_O_5_ system lattice parameters and phase identity vs. lithium stoichiometry *x*. Percentages indicate lattice parameter change relative to pristine α-V_2_O_5_. (b–e) Crystal structures of α-, ε-, δ-Li_*x*_V_2_O_5_ phases, with vanadium, oxygen, and lithium ions rendered respectively in yellow, blue, and green. (f) Detail of the layer shift accompanying the ε→δ structure transformation, visible in the change in alignment along *b* of opposing vanadyl oxygens. Figure reproduced with permission from ref 468. Copyright 2020 Cell.

As lithium content increases, the structure transforms into the ε-phase (ε-Li_*x*_V_2_O_5_, 0.32 < *x* < 0.80, Figure 48d), which features subtle puckering of the VO_5_ layers and increased interlayer separation along the *c* axis to accommodate the inserted ions. Continued lithiation leads to the δ-phase (δ-Li_*x*_V_2_O_5_, 0.88 < *x* < 1.00, Figure 48e), where more pronounced shifts in the layers occur (Figure 48f), leading to the sliding of the layers by half-a-unit-cell length along the *b* axis to better accommodate the significantly increased concentration of Li-ions. Upon further lithiation beyond *x* ≈ 1.0, the structure undergoes irreversible distortive phase transformation to γ- and ω-Li_*x*_V_2_O_5_ phases. In the γ-Li_*x*_V_2_O_5_ phase, the structure evolves through intermediate disordered states with highly puckered “up–down–up–down” arrangement of VO_5_ square pyramids, and exhibits a wider range of bond angles and lengths.^432,443,463^

At full lithiation, the ω-phase emerges, characterized by a complete rearrangement into a rock-salt-like disordered structure.^432,463^ These transformations from α to ε to δ to ω highlight the dynamic nature of the V_2_O_5_ lattice and are critical to its function as a cathode material, influencing both ion transport and structural stability during battery operation. To understand the origin of the observed modulation in Li-ion diffusivity, detailed ensemble and single-particle characterizations were conducted. Phase evolution as a function of Li-ion concentration was examined using *operando* XRD measurements.^465,466^

Lithiation of α-V_2_O_5_ single crystals is often accompanied by significant stress accumulation and phase inhomogeneity, presenting a key challenge to achieving controlled topochemical lithiation. These effects manifest as compositional and structural heterogeneities both within and between grains. Scanning transmission X-ray microscopy (STXM) (Figure 49) was employed to examine chemically lithiated α- and ζ-V_2_O_5_ nanowires. STXM hyperspectral maps (Figure 49a) captured X-ray absorption near-edge structure (XANES) spectra at the V L_3_-, V L_2_-, and O K-edges, enabling spatially resolved insight into changes in electronic and atomistic structure as lithium is inserted. These changes reflect vanadium reduction and accompanying lattice distortions, which affect both V L_3_-edge spectra and O K-edge spectra. Region-of-interest analysis in Figure 46b,c reveals significant lithiation heterogeneity across particles, even under identical conditions. Spectral differences between the ROIs mapped to varying lithiation levels are supported by reference standards and DFT simulations. Figure 49d highlights coexistence of multiple lithiated phases within single α-V_2_O_5_ nanowires. Finally, Figure 49e shows a progressive decrease in the *t*_*2g*_/*e*_g_* intensity ratio across different regions, providing further evidence of heterogeneity in lithium distribution and phase evolution.^431^

**Figure 49:**
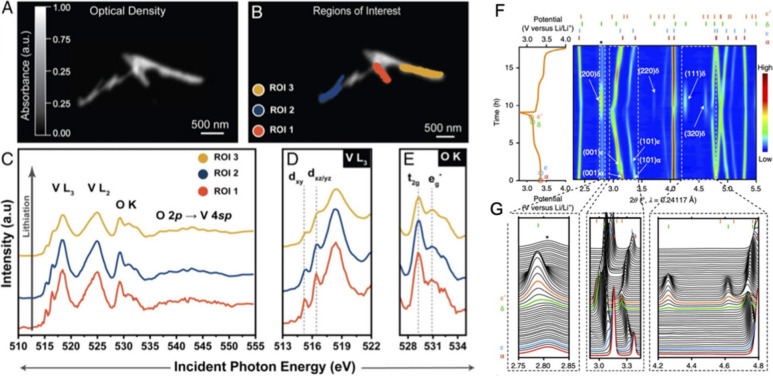
(a) Optical-density images of α-V_2_O_5_ nanowires obtained using STXM. The differences in the absorbance are due to the attenuation of X-rays that is dependent upon the linear absorption coefficient and sample thickness. (b) Regions of interest (ROIs) defined across the imaged sample. Three different regions, colored yellow, blue and orange, arise from the inhomogeneous lithiation of the α-V_2_O_5_ nanowires. The X-ray absorption near-edge spectra of (c) the V L_3_- and O K-edge, (d) a zoomed in view of the V L_3_-edge, and (e) a zoomed in view of the O K-edge of each of the three regions of interest identified in panel (b). The data is arranged in order of increasing lithiation from bottom to top, which was determined through changes in the relative intensity and peak positions of the V L_3_- and O-K spectra. (f) Galvanostatic discharge/charge profiles (left) and contour plots of *operando* synchrotron X-ray diffraction patterns (right) of bulk α-V_2_O_5_. These data reveal phase transformations that occur during the lithiation of α-V_2_O_5_ to ε-Li_*x*_V_2_O_5_, δ-Li_*x*_V_2_O_5_, and ε′-Li_*x*_V_2_O_5_ based on the emergence of plateaus in the charge/discharge profiles and new reflections within the powder X-ray diffraction. (g) Magnified views of representative X-ray diffraction patterns in selected 2θ regions. Panels (a)–(e) are reproduced from reference 192 under a CC BY license. Copyright 2022 Luo et al. Panels (f)–(g) are reproduced with permission from reference 431. Copyright 2022 Springer Nature.

On the other hand, electrochemical lithium insertion also triggers phase transformations that are clearly observed as plateaus in the charge/discharge curves at the left of Figure 49f.^467,431^ *Operando* synchrotron X-ray diffraction further shows different lithiated V_2_O_5_ phases during electrochemical cycling (Figure 49f,g). Subtle shifts in the (001) and (101) reflections mark the emergence of the slightly lithiated α-Li_*x*_V_2_O_5_ phase at Li_0.08_V_2_O_5_, which retains the orthorhombic unit cell of V_2_O_5_. At 3.32 V (Li_0.30_V_2_O_5_), the ε-Li_*x*_V_2_O_5_ phase appears, characterized by increased interlayer spacing and canting of the apical vanadyl moieties. A coexistence regime between α-Li_*x*_V_2_O_5_ and ε-Li_*x*_V_2_O_5_ exists from *x* ≈ 0.08–0.30, with the ε-Li_*x*_V_2_O_5_ phase growing with further lithiation. The appearance of new reflections at Li_0.88_V_2_O_5_ marks the onset of the highly lithiated ε′-Li_*x*_V_2_O_5_ phase. Further lithiation to Li_0.92_V_2_O_5_ (2.80 V) leads to the formation of the δ-Li_*x*_V_2_O_5_ phase, which shows a rearrangement of layers along the *b* axis. The phase coexistence regime spans ε-, ε′-, and δ-Li_*x*_V_2_O_5_ phases between 3.16 and 2.80 V. During charging, the reverse phase transitions occur, but δ-Li_*x*_V_2_O_5_ persists down to Li_0.47_V_2_O_5_ at 3.38 V, indicating the overpotential required for delithiation of micrometer-sized particles. These significant phase transformations that occur upon the lithiation of α-V_2_O_5_ introduce intergranular and intragranular compositional and phase heterogeneities during electrochemical cycling, which in turn induces considerable stress gradients and leads to capacity loss upon prolonged electrochemical cycling.

#### 5.2.2 γ′-V_2_O_5_

When the Li concentration exceeds 1 mol following Li_1–*x*_V_2_O_5_, there is an irreversible phase transformation to γ-Li_*x*_V_2_O_5_ (1 < *x* < 2), which exhibits a layered architecture composed of [VO_5_] distorted, corner connected square pyramids that form infinite double ribbons along *b* interconnected and create puckered layers perpendicular to *c* (Figure 50a). Temperature-dependent powder X-ray diffraction from 100–240 °C reveals the δ→ε→γ transition in a polycrystalline sample in Figure 50b.^469^ Notably, the vanadium sites in this polymorph mirror the structural complexity of δ-Li_*x*_V_2_O_5_, with distinct distortions in V–O bond lengths compared to α-V_2_O_5_.

**Figure 50:**
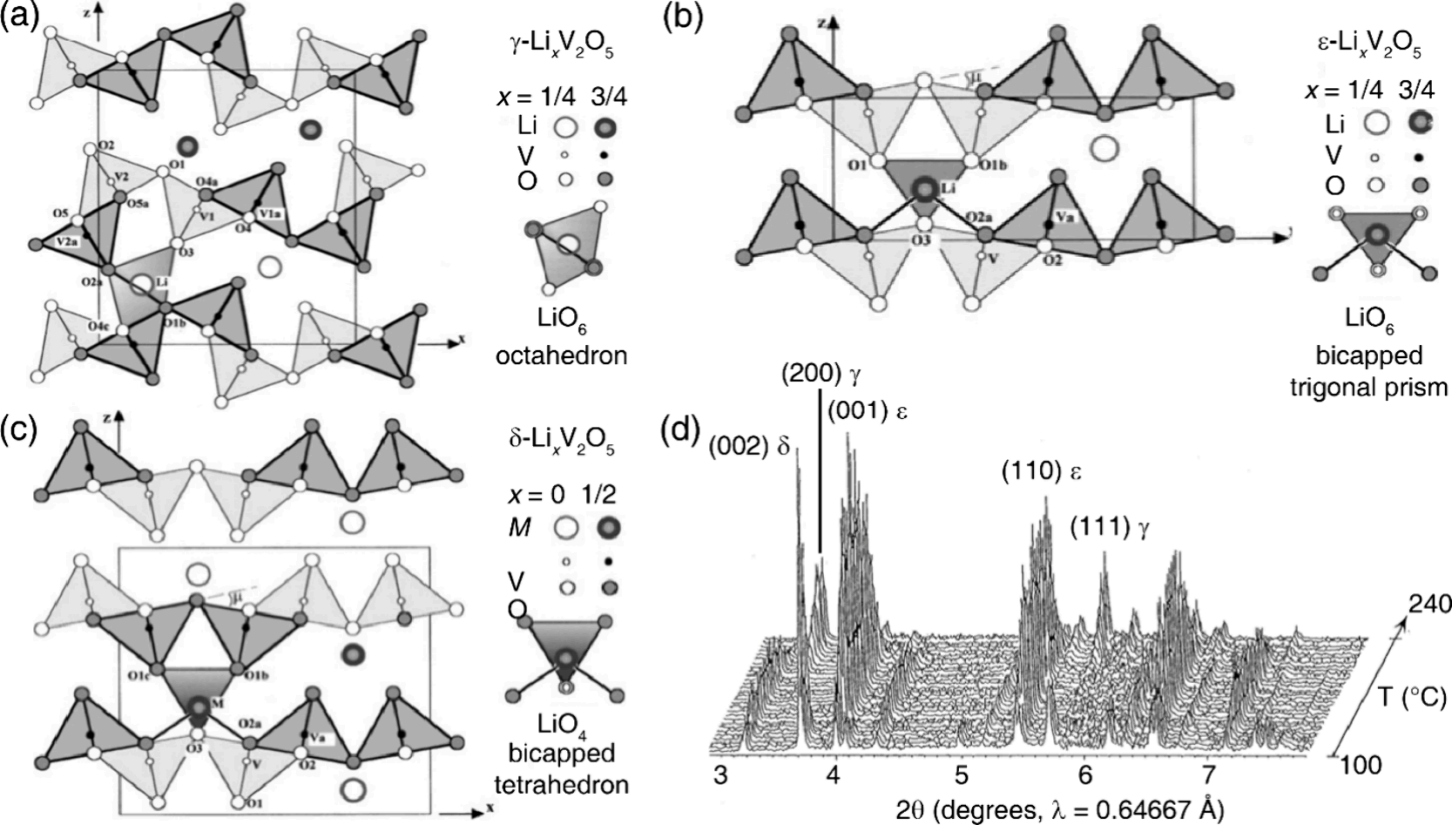
Crystal structures and lithium coordination environments of (a) γ-Li_*x*_V_2_O_5_, (b) ε-Li_*x*_V_2_O_5_, and (c) δ-Li_*x*_V_2_O_5_. (d) Temperature-dependent powder X-ray diffractogram showing δ→ε→γ transitions in LiV_2_O_5_ heated from 100 to 240 °C. This figure was reproduced with permission from reference 469. Copyright 1999 Elsevier.

Single crystal X-ray diffraction was able to establish that the Li cations are octahedrally coordinated by oxygen. Since one of the six bonds is significantly weaker than the remaining Li–O bonds, the Li local environment is also considered to be pseudo-octahedral.^27^ Satto et al. have also seen this phase transition to γ-Li_*x*_V_2_O_5_ using topochemistry.

Among the known metastable phases shown in Figure 47, γ′-V_2_O_5_ is readily accessible from topochemical transformations. Cocciantelli et al. first synthesized γ′-V_2_O_5_ from the topochemical delithiation from γ-LiV_2_O_5_,^459^ which preserves the V–O structural framework while removing Li ions from interstitial sites. We demonstrated the similar delithiation of macroscopic single crystals of γ-LiV_2_O_5_, which stabilized single crystalline γ′-V_2_O_5_ and enabled unprecedented atomic-level insights.^27^ The crystal stricture of γ′-V_2_O_5_ provides insights into interstitial sites accessible for lithium ions. The residual electron density surface (F_o_–F_c_ structure factor map) shows the electron density at a tetrahedral “B” position in-between the principle 6-coordinate Li “A” sites, mirrored along the *b* direction as shown in Figure 51a. Single-crystal X-ray diffraction supported by DFT calculations revealed that Li ions preferentially occupy octahedral *A* sites at higher lithiation levels, as shown in Figure 51b, whereas the tetrahedral *B* sites serve as transient positions during site-to-site ion migration. In this compound, rather than ordered staging of the inserted cations, the Li ions randomly distribute throughout the structure, which allows the structure to maintain an optimal layer separation to minimize local structural perturbations. A solid solution lithiation mechanism is evidenced from *x* = 0 to at least 1 in Li_*x*_V_2_O_5_; the change in local coordination of Li-ions follows 4–3–5–3–4 along the diffusion path.^443^

**Figure 51:**
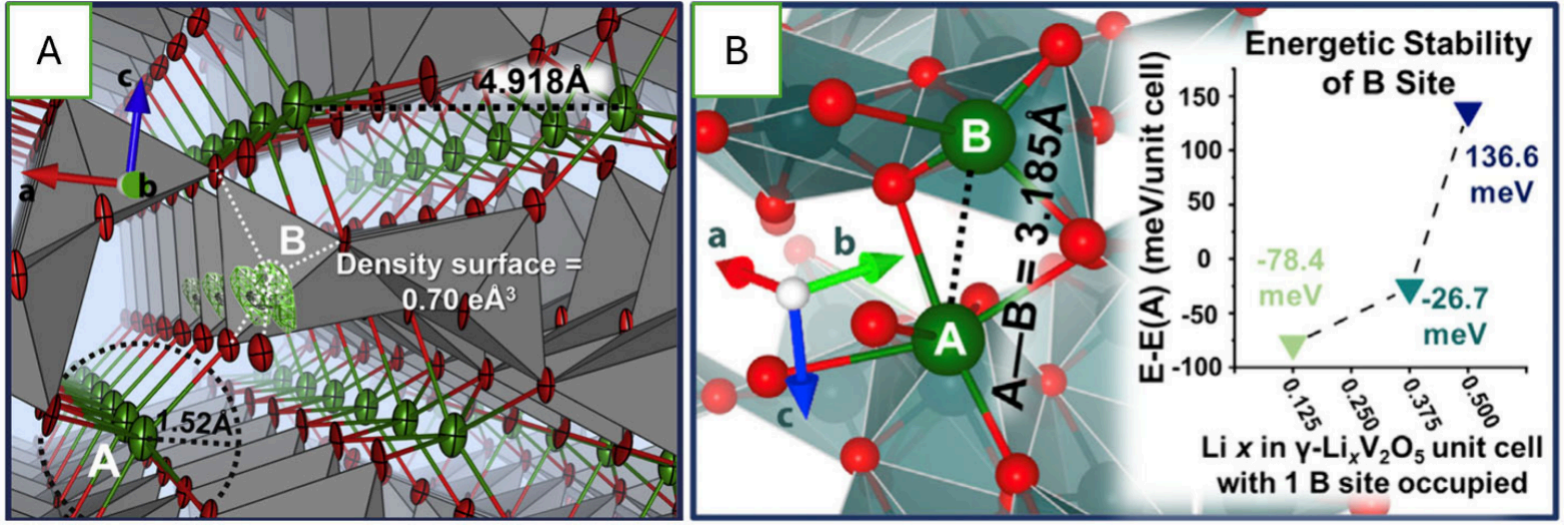
Diffusion pathway and local coordination sites accessed from topochemical treatment of γ′-V_2_O_5_ single crystals exemplifying the *A* and *B* sites from (a) single crystal X-ray diffraction and (b) first-principles density functional theory calculations. This figure was reproduced from reference 27 under a CC BY license. Copyright 2022 Handy et al.

The electrochemical insertion of various cations (lithium, sodium, potassium, and zinc ions) into γ′-V_2_O_5_ has been systematically investigated by Pereira-Ramos and co-workers to correlate the observed structural evolution with electrochemical cycling. Metastable γ′-V_2_O_5_ was stabilized through the deintercalation of γ′-LiV_2_O_5_ using NO_2_BF_4_ as the oxidizing agent. This empty γ′-V_2_O_5_ phase can then be utilized as a host for the insertion of different cations such as Na, K, and Zn. γ-Na_*x*_V_2_O_5_ can be obtained through the electrochemical sodiation of γ-Na_*x*_V_2_O_5_ (0 ≤ *x* ≤ 0.97) within a potential window of 1.75 to 4.00 V, using a galvanostatic discharge rate of C/10 to investigate the sodium-driven structural changes (Figure 52a). The sodiation of γ-Na_*x*_V_2_O_5_ was analyzed at different points of discharge using *ex situ* X-ray diffraction (Figure 52b). When sodium content *x* = 0.05, new (002) reflections were observed that coexisted with reflections corresponding to pristine γ′-V_2_O_5_. These reflections indicate an expansion of the interlayer spacing along *c* to 11.94 Å compared to 10.04 Å from γ′-V_2_O_5_ as a result of Na-ion insertion on Wyckoff 4*c* sites. Further Na-ion insertion yields biphasic domains in the composition range 0.05 ≤ *x* ≤ 0.7. Simultaneously, the intensities of characteristic reflections of γ′-V_2_O_5_ are gradually diminished, indicating conversion to the sodiated phase. At Na concentrations of 0.8 ≤ *x* ≤ 1, a single orthorhombic phase is observed with final refined lattice parameters of *a* = 9.76 Å, *b* = 3.63 Å, and *c* = 11.94 Å (Figure 52c).

**Figure 52:**
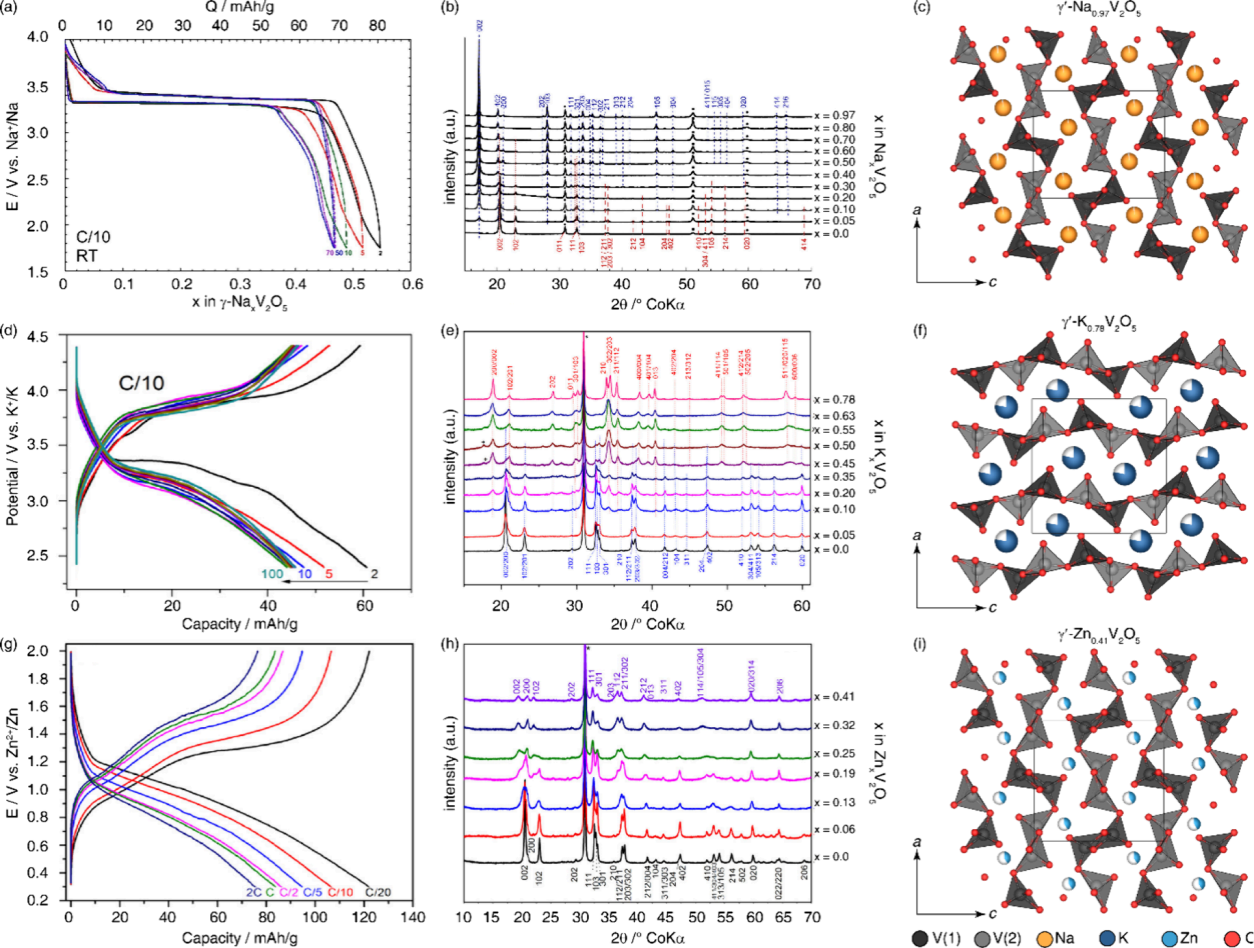
(a) The charge/discharge curves and (b) *ex situ* powder X-ray diffraction of Na_*x*_V_2_O_5_ of depict the emergence of a new sodiated γ′-V_2_O_5_. (c) γ′-Na_0.97_V_2_O_5_ crystallizes in orthorhombic *Pnma*. (d) The charge/discharge curves and (e) *ex situ* powder X-ray diffraction of K_*x*_V_2_O_5_ reveal three distinct regimes that must be progressed through to obtain the final (f) γ′-Na_0.78_V_2_O_5_. Zn-ion insertion within γ′-V_2_O_5_ was also evidenced through (g) cycling experiments at various C rates and (h) powder X-ray diffraction, which revealed the emergence of (i) γ′-Zn_0.41_V_2_O_5_. Panels (a)–(c) were reproduced with permission from reference 471. Copyright 2017 Elsevier. Panels (d)–(f) were reproduced with permission from reference 43. Copyright 2021 American Chemical Society. Panel (g)–(i) were reproduced with permission from reference 44. Copyright 2022 American Chemical Society.

Potassium has also successfully been electrochemically substituted within γ′-V_2_O_5_. Bhatia et al. constructed two-electrode coin cells utilizing γ′-V_2_O_5_ as the positive electrode, potassium metal as the negative electrode, and 0.5 M KPF_6_ in a 1:1 mixture of ethylene carbonate (EC) and propylene carbonate (PC) with 2 vol % fluoroethylene carbonates (FEC) as the electrolyte. They investigated its (de) potassiation following K_*x*_V_2_O_5_ (0 ≤ *x* ≤ 0.78). The charge/discharge curves (Figure 52d) and subsequent *ex situ* powder X-ray diffraction (Figure 52e) of K_*x*_V_2_O_5_ within a potential range of 4.4–2.4 V vs K^+^/K revealed three distinct regimes: (i) a solid-solution region in the range 0 ≤ *x* ≤ 0.05 within γ-K_*x*_V_2_O_5_ during initial K-ion insertion; (ii) a two-phase domain between 0.05 < *x* ≤ 0.5 along the voltage plateau at 3.3 V, corresponding to coexistence of γ-K_0.05_V_2_O_5_ and a new phase of K_0.5_V_2_O_5_; and (iii) a solid-solution region between 0.5 < *x* ≤ 0.78 that favors the formation of K_0.78_V_2_O_5_ at the end of the discharge potential at 2.4 V. Figure 52f illustrates the crystal structure of γ-K_0.78_V_2_O_5_, which retains the *Pnma* space group of γ′-V_2_O_5_. The structure features a layered arrangement along *b* and *c*, with potassium ions occupying Wyckoff 4*a* sites in 9-fold coordination with oxygen atoms. The K ions generally prefer sitting in 8-, 9-, or 10-fold coordinated configurations.^470^ The chains of [VO_5_] square pyramids along the *c* axis include two types of linkages: one formed by corner-sharing O(3) oxygen atoms, and the other by edge-sharing between adjacent [VO_5_] units through V–O(2) bonds. that alternate in orientation, in direct contrast with the pristine γ′-V_2_O_5_ structure, where edge-sharing [VO_5_] pyramids uniformly point upward or downward. This arrangement resembles the alternating VO_5_ geometry in α-V_2_O_5_. Finally, Bhatia et al. also studied zinc-ion insertion into γ′-V_2_O_5_ using a nonaqueous Zn metal cell with Zn­(CF_3_SO_3_)_2_ (Zn­(OTf)_2_) in acetonitrile as the liquid electrolyte (Figure 52g).^44^ *Ex situ* X-ray diffraction at different points of discharge revealed the reversible formation of γ-Zn_0.41_V_2_O_5_ upon cycling at C/20 rate (potential range: 0.3–2.0 V vs Zn^2+^/Zn). Zn_0.41_V_2_O_5_ crystallizes in the *Pnma* space group where Zn insertion results in a 6.4% volume expansion as compared to pristine γ′-V_2_O_5_ as a result of expansion of the interlayer spacing (leftward shift of the 002 reflection in Figure 52h). Rietveld refinements indicate that Zn^2+^ is located in the interlayer space, occupying the same 4c position as Na-ions in γ-Na_0.96_V_2_O_5_ and Li-ions in γ-LiV_2_O_5_, with a distorted octahedral coordination (average Zn–O distance: 2.238(3) Å). Edge-sharing [ZnO_6_] chains are stabilized along the [010] direction (Figure 52i), in contrast to K^+^ ions, which have a larger radius and occupy 4a sites with 9-fold coordination.

#### 5.2.3 ζ-V_2_O_5_

The recent discovery of a 1D tunnel-structured polymorph of V_2_O_5_, named ζ-V_2_O_5_, has afforded compelling platform for probing ion insertion mechanisms and lattice adaptability.^460,472,473^ ζ-V_2_O_5_ is prepared by removal of Ag- or Cu-ions from β-Ag_*x*_V_2_O_5_ or β′-Cu_*x*_V_2_O_5_, respectively.^26^ β-Ag_*x*_V_2_O_5_ is prepared by reacting AgCOOCH_3_ with α-V_2_O_5_ hydrothermally, followed by the leaching of Ag^+^ ions using dilute HCl to access ζ-V_2_O_5_. The AgCl solid byproducts formed during the leaching process are then removed by treatment with sodium thiosulfate (Na_2_S_2_O_3_).^460^ An alternative approach involves the synthesis of β′-Cu_0.64_V_2_O_5_ by a solid-state or hydrothermal reaction of Cu metal with α-V_2_O_5_. Leaching the Cu ions from this host using Na_2_S_2_O_8_ yields ζ-V_2_O_5_, a translucent orange phase with oxidized V^5+^. Single-crystal structure solutions of metastable tunnel-structured ζ-V_2_O_5_ ^26^ enable identification of distinctive interstitial positions as shown in Figure 53a–c. Studies on single crystals reveal that the 5-fold coordinated β′ site is at a half-unit-cell distance along the *b* axis from the 7-fold coordinated β site. The β and β′ sites have a Wyckoff position of 4i, although Cu in the β′ site occupies an additional 8j position owing to its split site behavior, discussed in Section 3.5.3.^26^ Particular quaternary compositions situate Li ions in 2-coordinate C site (tunnel center, Wyckoff position 2c) or 6-coordinate I site (intralayer octahedral site between tunnels, 2d).

**Figure 53:**
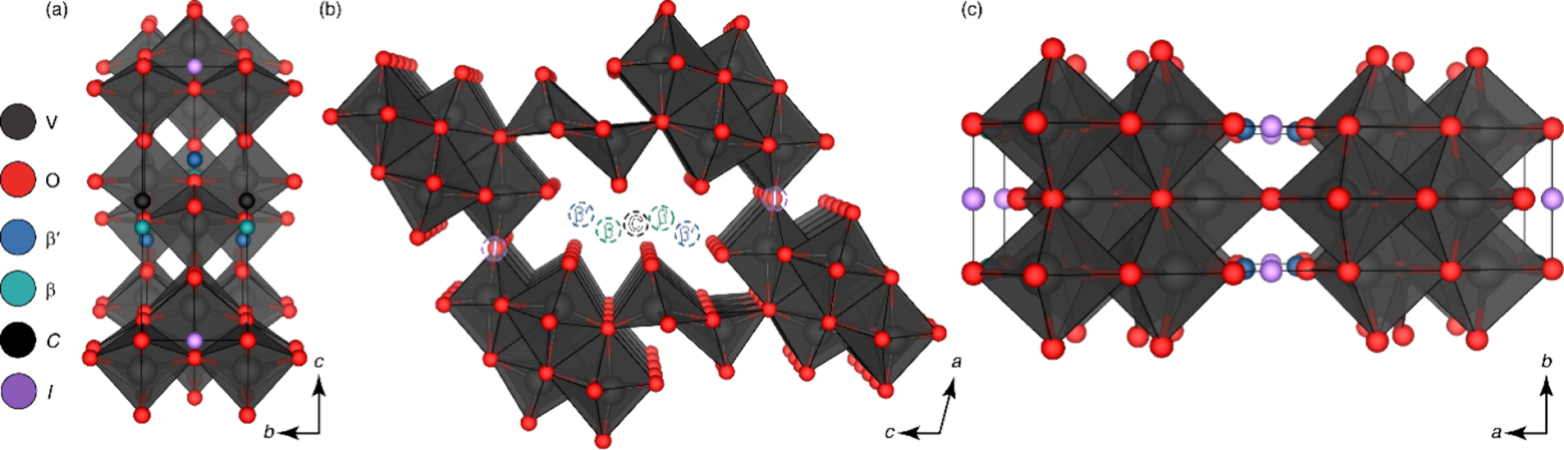
Crystal structure of ζ-V_2_O_5_ which is composed of 1D tunnels. The various vacant interstitial positions within these 1D tunnels are β at Wyckoff 4*i* (*x*, 0.5, *z*), β′ at Wyckoff 4*i* (*x*, 0, *z*), *C* at Wyckoff 2*c* (0.5, 0.5, 0.5), and *I* at Wyckoff 2*b* (0.5, 1,0, 1.0). The structure is shown here along (a) the *a* direction, (b) the *b* direction, and (c) the *c* direction to showcase the vacant positions. For clarity in panels (a) and (c), the vacant β, β′, *C*, and *I* sites are represented by blue, green, black, and purple spheres, respectively. It is important to note that the size of these spheres are equal and therefore does not represent the size of the cation that may substitute on each interstitial position.

The lithiation of ζ-V_2_O_5_ single crystals revealed significant site-selectivity driven by the concentration of the inserted Li-ions. Treating ζ-V_2_O_5_ with 0.001 M *n*-BuLi up to Li_0.39_V_2_O_5_ demonstrated preferential Li-ion occupancy of the β site accompanied by relatively subtle changes of the monoclinic angle (β = 110.0° to 110.7°) and modest unit cell expansion from ≈518 to ≈521 Å^3^. Increasing the Li concentration beyond 0.33 mol of Li results in cation reordering with Li-ions migrating to the β′ site. This causes a slight reduction in the β angle to 106.7° and results in a further expansion of the lattice (to a unit cell volume of 531 Å^3^), which is indicative of the distortion of the tunnel to accommodate the higher degrees of electrostatic repulsion between inserted Li-ions. The Li concentration can be further increased to Li_1.2_V_2_O_5_, which engenders the Li ions to occupy a novel central C site with a 2-fold coordination at the center of the tunnel. This is accompanied by a dramatic contraction of the long V–O bond (2.41 Å vs 2.64–2.77 Å), which alters the local vanadium coordination environment from square pyramidal to octahedral. Delithiation with NOBF_4_ restores the framework, underscoring the reversibility of these structural changes. Table 3 shows the relatively modest changes in local coordination environment, which engenders a lower barrier to Li-ion diffusion.^443^

Topochemical lithiation of ζ-V_2_O_5_ single crystals provided precise insights into the different interstitial sites available within the host framework. Pillaring experiments were subsequently used to identify interstitial sites accessed at still higher extents of lithiation. Figure 54 demonstrates a site-selective positioning approach based on preinsertion of ζ-V_2_O_5_ with cations that serve as analogs of “protecting groups” in organic chemistry. Such ions selectively occlude specific interstitial sites and are thus able to redirect Li-ion migration.^306^ For example, inserting Ag cations in β sites, redirects Li ions to β′ or C sites. Similarly, positioning Cu ions in β′ sites redirects Li-ions to an entirely new site between tunnels, known as the I site. Li-ions occupying this site engender structural distortions in the form of unit cell expansion (unit cell volume ≈ 542.5 Å^3^) and a reduction of the β-angle (104.7°). Table 4 lists site preferences and occupancies for different combinations of pillaring ions.

**Table 4:** Inserted Cations within ζ-V_2_O_5_, Chemical Formula, and the Resulting Site That Is Occupied by Li^a^

Guest ions	Formula	Site occupied by Li	Reference
Li	β-Li_0.39_V_2_O_5_	β	^26^
Li	β′-Li_0.66_V_2_O_5_	β′	^26^
Li	β′/C-Li_1.2_V_2_O_5_	β, β′, and C	^26^
Li/Ag	β′-Li_0.26_/β-Ag_0.27_V_2_O_5_	β′	^306^
Cu/Li	β′-Cu_0.29_/C-Li_0.08_V_2_O_5_	C	^306^
Cu/Li	β′-Cu_0.49_/I-Li_0.33_V_2_O_5_	I	^306^
Cu/Ag/Li	β′-Cu_0.17_/β-Ag_0.29_/C-Li_0.12_V_2_O_5_	C	^306^
Li/Na	β′-Li_0.26_/β-Na_0.32_V_2_O_5_	β′	^25^
Li/K	β′-Li_0.26_/β-K_0.27_V_2_O_5_	β′	^25^

a Data from refs 25, 26, 306 .

**Figure 54:**
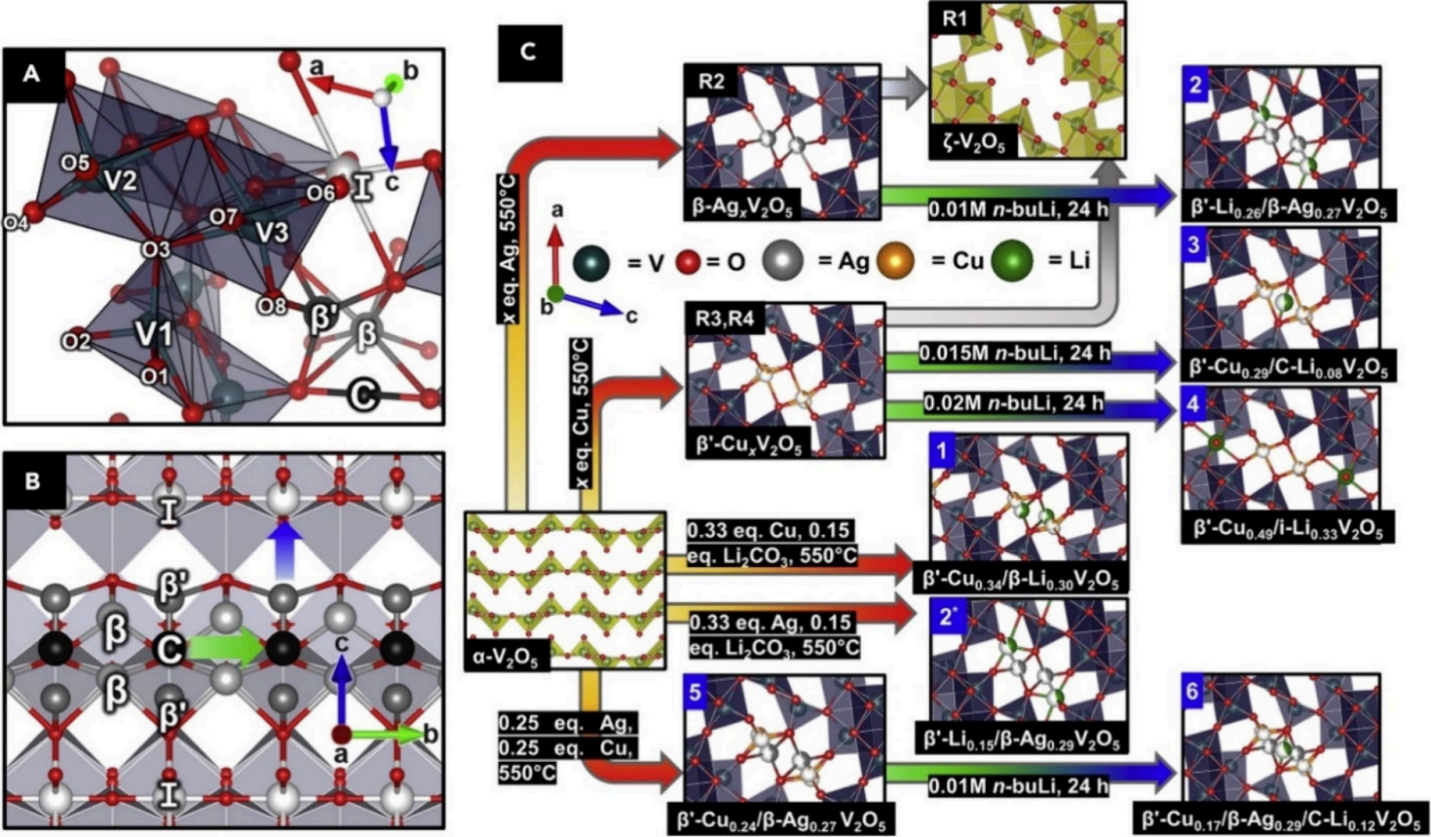
(a) The anisotropic unit of ζ-V_2_O_5_ where the V-centered polyhedra that surround the vacant interstitial sites (β, β′, C, and I). (b) A view along *a* of the tunnels present in ζ-V_2_O_5_ where the vacant interstitial positions are highlighted in greyscale and labeled. When all of the sites are occupied, the structure is nearly close packed. The green arrow depicts the direction of diffusion along *b* whereas the blue arrow depicts the direction of diffusion along *c*. (c) A schematic depicting the synthetic transformation accessible when starting from α-V_2_O_5_. When using the appropriate metals or salts, the β, β′-*M*_*x*_V_2_O_5_ and *M*_*x*_ *M′*_*y*_V_2_O_5_ (*M*/*M′* = Ag, Cu, Li) structures can be accessed where cations of different stoichiometries can selectively occupy the available sites. Topochemical lithiation routes are shown as green and blue arrows and the blue labels of 1–6 are used to indicate the new crystal structures that can be obtained upon selective positioning of the Li ions. The gray arrows indicate the multiple topochemical pathways toward obtaining ζ-V_2_O_5_. R1 is used to denote ζ-V_2_O_5_, R2 refers to β-Ag_0.30_V_2_O_5_, R3 refers to β′-Cu_0.38_V_2_O_5_, and R4 refers to β′-Cu_0.50_V_2_O_5_. This figure was reproduced with permission from reference 306. Copyright 2023 Cell Press.

Figures 51 and 54 exemplify the utility of single-crystal X-ray diffraction to yield high-resolution atomic positions, unit cell volumes, and monoclinic β angles across a series of topochemical transformations. Single crystal diffraction reveals dynamically adaptations of structure such as anisotropic expansion of the tunnel induced in response to ion insertion, polyhedral distortions, and/split-site disorder.^365^ Indeed, single crystal X-ray diffraction is key to delineating the site preferences for different inserted cations. Ag-ions preferentially occupy the 7-coordinated β sites because of their larger ionic radius of 1.15 Å, whereas Cu-ions prefer 5-coordinated β′ sites owing to their smaller ionic radius (0.77 Å). The high-resolution structure solutions further provide a means of interpreting *operando* powder XRD data. For instance, the monoclinic β angle serves as a sensitive probe of the depth of discharge. The β angle contracts most strongly upon filling of β′ sites. Finally, performing topochemical transformations on single crystals along a continuum of filled-and-emptied phases while retaining the 1D atomic connectivity of ζ-V_2_O_5_ enables the construction of electron density maps from single-crystal diffraction that provides an atomic resolution view of the paths traversed by Li-ions through the crystal lattice. A 5 →4→→2→4→5 coordination environment sequence is mapped based on SCXRD.

Understanding the dynamic evolution of ζ-V_2_O_5_ upon electrochemical ion insertion requires *operando* synchrotron X-ray diffraction, interpretation of which is greatly aided by high-resolution structure solutions available from SCXRD. *Operando* synchrotron X-ray diffraction was used to track Li-ion reordering within ζ-V_2_O_5_ interstitial sites during electrochemical lithiation/delithiation.^431^ These studies enable identification of the site-filling sequence as a function of potential and depth of discharge (Figure 55a). Across the entire range of electrochemical lithiation/delithiation, the monoclinic *C*2/*m* space group is retained, and the 1D tunnel structure of ζ-V_2_O_5_ remains fully preserved. During initial lithium-ion insertion (Regime I, 0 < *x* < 0.3 in Li_*x*_V_2_O_5_), a slight expansion of the tunnel structure occurs up to 3.38 V, corresponding to the filling of the β site along the tunnel. As the cell is discharged further to 2.89 V (Regime II, 0.3 < *x* < 0.66), the coexistence of two distinct Li-ion occupied sites (β and β′) is observed. Filling of the β′ site is increasingly favored with increasing concentration of Li-ions. At deeper depths of discharge (Regime III, down to 2.0 V), additional Li-ions are accommodated through the filling of β′ sites and C sites aligned along the tunnel center. This process engenders significant structural expansion along the *b* axis to enable simultaneous occupation of β′, C, and β sites.

**Figure 55:**
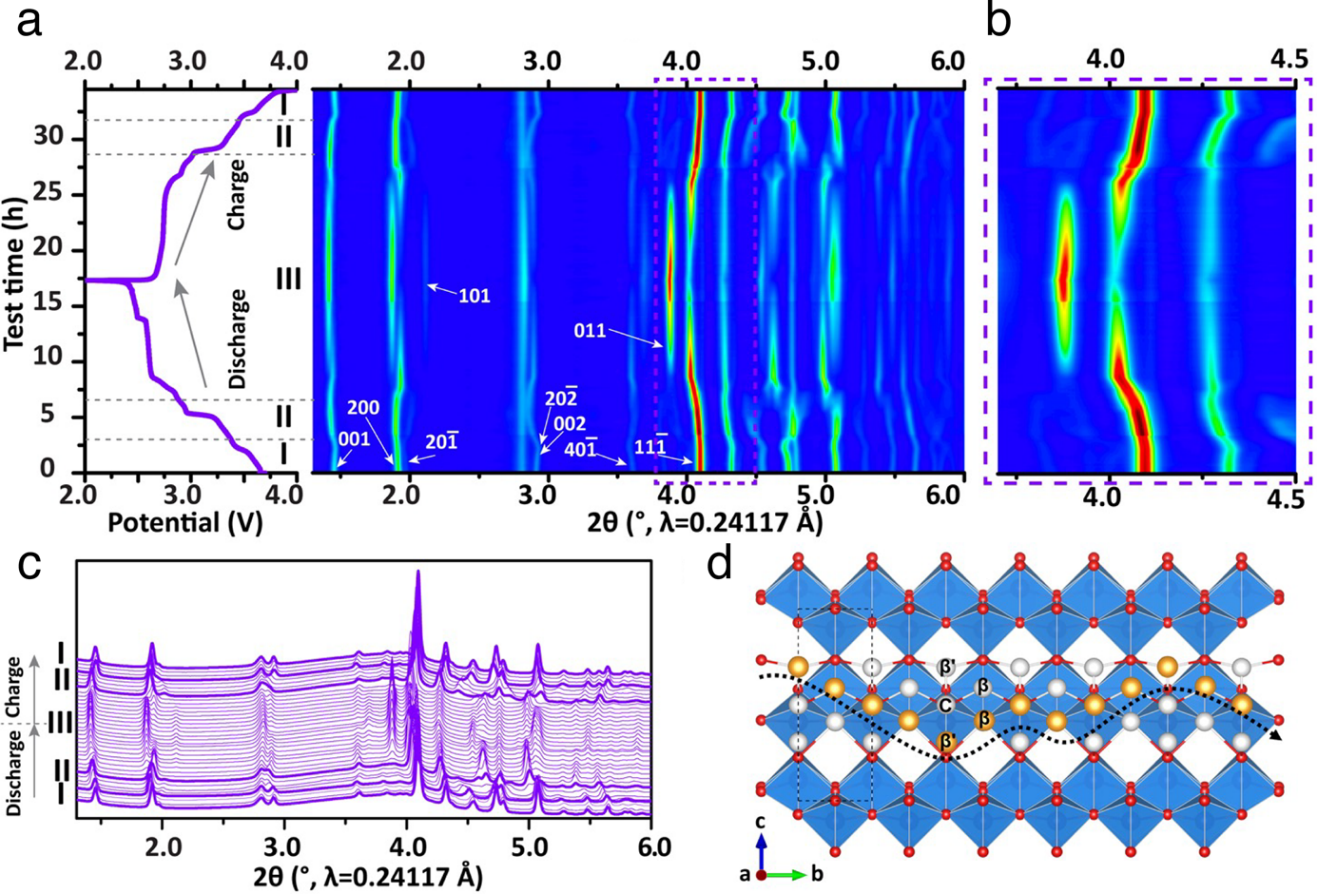
(a) Discharge/charge profile (Left) ζ-V_2_O_5_ at a C-rate of C/20 and contour plot of intensities of corresponding reflections (right). (b) Magnified view of diffraction intensity in the 2θ range from 3.7 to 4.5°. (c) Waterfall plot of select diffraction patterns acquired during lithiation/delithiation of ζ-V_2_O_5_. (d) Cut-away view of the ζ-V_2_O_5_ tunnel viewed down the *a* axis. White spheres indicate possible Li positions (β, β′, and C, respectively) in a highly lithiated structure, with orange spheres used to highlight the positions of an Li-ion along a proposed diffusion pathway. This figure was reproduced from reference 192 under a CC BY license. Copyright 2022 Luo et al.

During the charging process, as shown in Figure 55b, this highly lithiated regime undergoes a stepwise delithiation to stabilize Regimes III, II, and I, ultimately recovering the empty tunnel structure of ζ-V_2_O_5_ at the end of the charge cycle (4.0 V). Figure 55c shows a succession of reflections in a waterfall plot of selected diffraction patterns, which is indicative of Li-ion reordering within the tunnels. Such cation reordering with preservation of the tunnel-structured framework stands in stark contrast to the sequence of distortive structural transformations evidenced for α-V_2_O_5_ as plotted in Figure 48.

To further capture how pillaring ions modify lithium-ion diffusion pathways *operando* synchrotron powder X-ray diffraction was performed to understand the dynamic evolution of preintercalated compounds during (de)­lithiation.^25^ Here, the pillaring ions of interest were Na and K, whose positions were determined using SCXRD (Figure 56). Single-crystal structure analysis reveals that preintercalated β-Na_0.32_V_2_O_5_ (Figure 56a) and β-K_0.22_V_2_O_5_ (Figure 56b) maintain the same *C*2/*m* space group as the empty ζ-V_2_O_5_ polymorph (Figure 56c), but with distinctive structural modifications. Both Na^+^ (Figure 56d) and K^+^ ions (Figure 56e) occupy β interstitial sites, exhibiting 7-fold distorted pentagonal-bipyramidal coordination environments. This is in direct contrast with Li^+^ ions that occupy a five-coordinated β′ site (Figure 56f). The key difference between Na and K preintercalation lies in their modulation of the tunnel structure and the resulting implications for Li-ion diffusivity. K-ion preinsertion causes a more significant volume expansion (2.5%) compared to Na-ion preinsertion (0.76%) with respect to ζ-V_2_O_5_, which is consistent with potassium’s larger ionic radius (1.60 Å vs 1.26 Å for Na^+^ in 7-fold coordination). Lithiation brings about starkly distinctive behavior in the two materials. Na-ions undergo pronounced reordering by segregating to one side of the tunnel while Li ions occupy β′ sites on the opposite side (adjacent tunnels are inversions of each other), thereby transforming the material to a *P*2_1_/*m* space group. In contrast, K-preintercalated structures maintain the *C*2*/m* symmetry after lithiation, with K^+^ and Li^+^ showing high site selectivity but with random distribution across their preferred sites β and β′ sites, respectively. This difference in ordering behavior stems from the lower mobility of K-ions.

**Figure 56:**
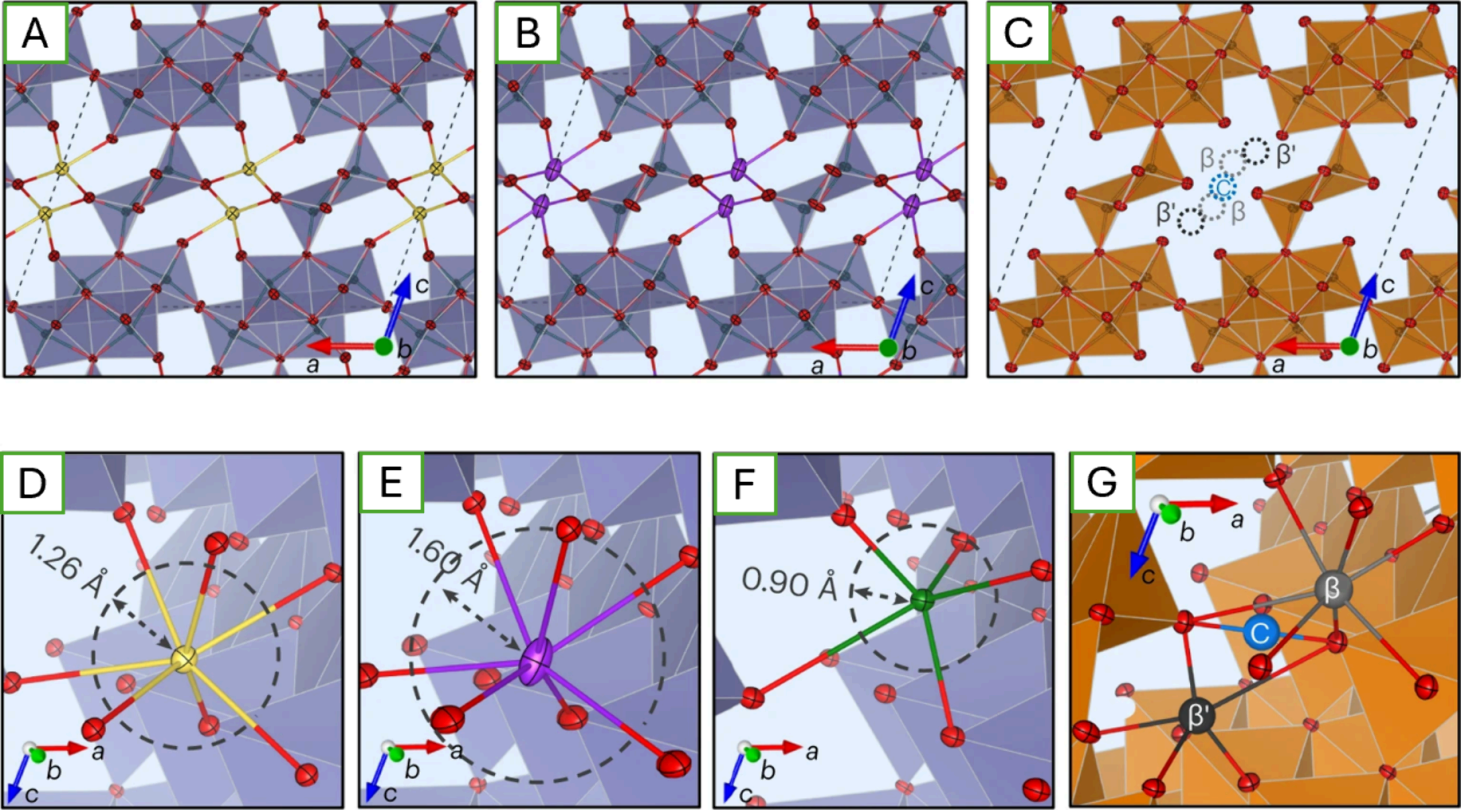
Crystal structures of (a) β-Na_0.32_V_2_O_5_, (b) K_0.22_V_2_O_5_, and (c) ζ-V_2_O_5_ shown along *b*. (d) Na^+^ and (e) K^+^ occupy seven-coordinated β sites whereas (f) Li^+^ ions occupy the five-coordinated β′ site. (g) A perspective view of ζ-V_2_O_5_ with the interstitial sites highlighted. This figure was reproduced with permission from reference 25. Copyright 2024 Springer Nature.

The charge/discharge profiles of β-Na_0.25_V_2_O_5_ (Figure 57a, left) and β-K_0.27_V_2_O_5_ (Figure 57c, left) exhibit several distinctive plateaus upon discharge to 2.0 V and charge to 4.0 V vs Li/Li^+^.^25^ The corresponding contour plots in Figure 57a, right and Figure 57c, right reveal reversible features for both compounds. The incorporation of these pillaring ions modifies the lattice symmetry of the host framework. For both β-Na_0.25_V_2_O_5_ and β-K_0.27_V_2_O_5_, initial Li-ion insertion (0 < *x* ≤ 0.4 in in Li_*x*_Na_0.25_V_2_O_5_ and 0 < *x* ≤ 0.3 in Li_*x*_K_0.27_V_2_O_5_) causes a slight expansion of the tunnel structure (Figure 57b). Upon further lithiation of Li_*x*_Na_0.25_V_2_O_5_ within a range of 0.4 < *x* ≤ 0.8, the tunnel further expands, yielding a unit cell volume of 537.73 Å^3^ and a monoclinic β-angle of 112.218°. These cell parameters are analogous to highly lithiated ζ-V_2_O_5_ where the β and β′ sites are occupied, causing the C sites to begin filling. Further lithiation to 2.0 V (0.8 < *x* ≤ 1.7 in Li_*x*_Na_0.25_V_2_O_5_) expands the unit-cell volume to 570.097 Å^3^ and reduces symmetry to *P*2/*m* with a β angle of 105.14°, corresponding to Li^+^ occupying the remaining β sites. The lithiation of β-K_0.27_V_2_O_5_ (Figure 57d) follows a similar trend. Initial lithiation expands the tunnel structure; Within the range (0.3 < *x* ≤ 0.8 in Li_*x*_K_0.27_V_2_O_5_) results in a splitting of new (001) reflections (Figure 57c, right) and a symmetry reduction to *P*2/*m*. Here, the behavior of β-K_0.27_V_2_O_5_ stands in contrast to β-Na_*x*_V_2_O_5_ as the inserted cations causes the unit cell volume to contract to 555.402 Å^3^. Moreover, the symmetry further reduces to *P*2_1_ as the electrochemically inserted Li-ions fill β′, C, and β sites that become accessible upon tunnel expansion. Despite these changes in the symmetry of the host lattice, Li-ion insertion is completely reversible demonstrating full preservation of the 1D tunnel framework.

**Figure 57:**
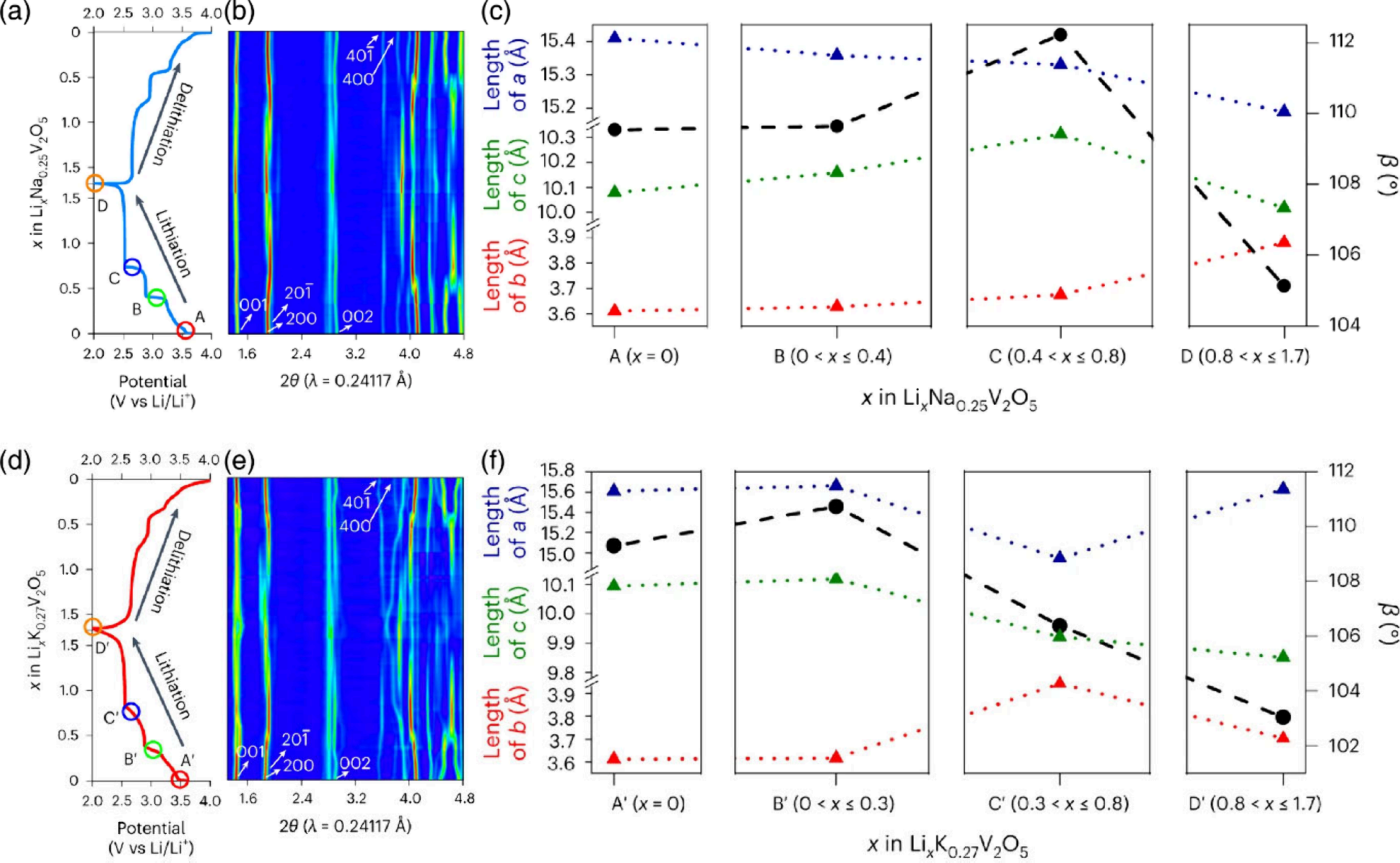
(a) Galvanostatic discharge/charge profiles acquired during *operando* powder XRD measurements (b) and corresponding XRD contour plots for β-Na_0.25_V_2_O_5_. (c) The change in lattice parameters for β-Na_0.25_V_2_O_5_ across different lithiation regimes. (d) Galvanostatic discharge/charge profiles acquired during *operando* powder XRD measurements (e) and corresponding XRD contour plots for β-K_0.27_V_2_O_5_. (f) The change in lattice parameters for β-K_0.27_V_2_O_5_ across different lithiation regimes. This figure was reproduced with permission from reference 25. Copyright 2024 Springer Nature.

#### 5.2.4 λ-V_2_O_5_

As discussed in Section 1.2 in ternary vanadium oxide bronzes, the V_2_O_5_ framework adapts to both the size and hardness of inserted cations as well as their stoichiometry. For example, β′-Cu_*x*_V_2_O_5_ reflects occupation of βˈ sites up to a stoichiometry of *x* ∼ 0.67 along the tunnels of the ζ-V_2_O_5_ framework as discussed above in Section 3.5.3. Increasing the Cu concentration to 0.67 < *x* < 0.9 induces a structural reorganization to ε-Cu_*x*_V_2_O_5_. This phase is a double-layered bronze and comprises slabs of edge-sharing [VO_6_] octahedra (Figure 58a). The electronegativity of the formally pentavalent V and the resulting V–O covalency induces an off-centering of the vanadium ion to form short V(1)–O(1) and V(2)–O(2) vanadyl bonds oriented toward interlayer interstitial sites occupied by inserted ions.^41,67^ In ε-Cu_*x*_V_2_O_5_, SCXRD reveals two distinct Cu coordination environments where Cu (1) occupies an octahedral site and Cu(2) occupies a square pyramidal site, as shown in Figure 58a.

**Figure 58:**
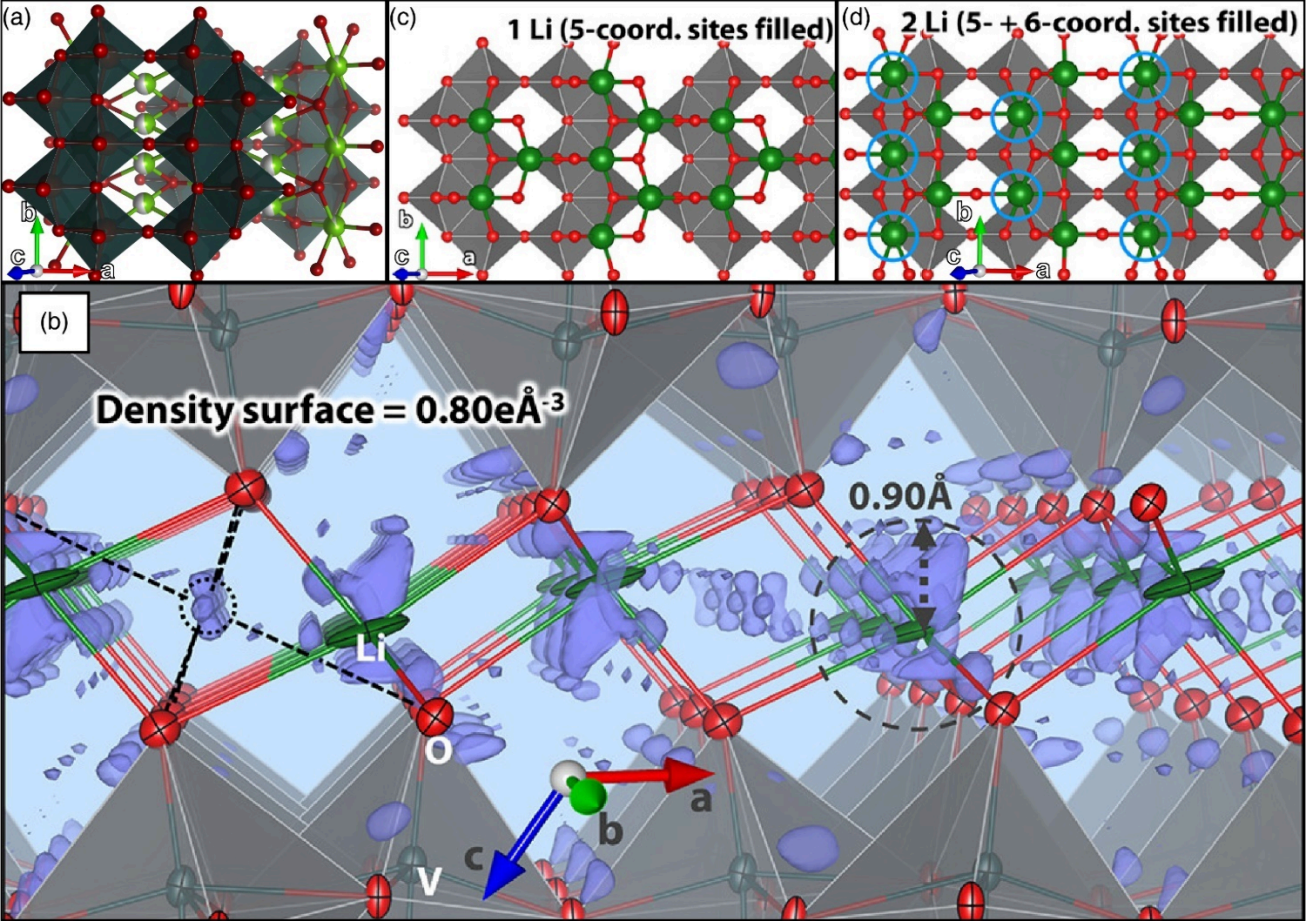
(a) Insights into the local coordination of ε-Cu_0.85_V_2_O_5_. (b) A “Li-eye” view of λ-LiV_2_O_5_ obtained from a single crystal to single crystal transformation from λ-V_2_O_5_, which shows a 5-fold coordinated Li surrounded by residual electron density. First-principles calculations of λ-LiV_2_O_5_ exhibiting (c) one and (d) two Li per V_2_O_5_. This figure was reproduced with permission from reference 36. Copyright 2022 American Chemical Society.

Garcia-Alvaro et al. pioneered topochemical copper deintercalation from ε-Cu_*x*_V_2_O_5_ powders with a variety of reagents such as Br_2_, I_2_, and NO_2_BF_4_.^37^ It was found that that concentration of the residual Cu remaining within the host framework was inversely correlated with the oxidizer strength (I_2_ < Br_2_ < NO_2_BF_4_). In past work, we stabilized single crystals of metastable λ-V_2_O_5_ by topochemical decupration of ε-Cu_*x*_V_2_O_5_ using NOBF_4_ in acetonitrile.^36^ As described in Section 1.2.2, this structure consists of V_4_O_20_ units edge-shared along the *a*- and *b*-axes and separated by a van der Waals gap.

The layered structure of λ-V_2_O_5_ allows for the insertion of ions within the host framework. Topochemical lithiation of λ-V_2_O_5_ enables a similar study to that of ζ-V_2_O_5_ where the lithium diffusion pathway can be elucidated. Single-crystal-to-single-crystal transformations enables the direct visualization of Li-ion migration paths based on the residual electron density around the 5-coordinated Li-ions as illustrated in Figure 58b. Figure 58c shows that Li-ions initially occupy 5-coordinate sites and, once these are filled, migrate to 6-coordinate sites (Figure 58d). This sequential site occupancy, coupled with anisotropic diffusion primarily along the *b* axis and secondarily the *a* axis is corroborated by DFT calculations.

### 5.3 Topochemistry of VO_2_

Vanadium­(IV) oxide, similar to its vanadium­(V) counterpart, exhibits a rich landscape of accessible metastable polymorphs including VO_2_ (B), VO_2_ (A), VO_2_(M), VO_2_ (R), VO_2_ (D), and VO_2_ (P), which can be synthesized based on conventional solvothermal or solid-state methods (Figure 59).^8,474,475^ Several open-framework VO_2_ phases serve as effective insertion hosts for lithium ions, accommodating Li-ions through topochemical and electrochemical methods. For example, Theobald et al. reported that the disordered VO_2_(B) (*C*2/*m*) polymorph can be topochemically lithiated through treatment with *n*-butyllithium and lithium iodide, and subsequently delithiated with iodine.^476^ This lithiation process yields a Li_*x*_VO_2_ bronze with a lithium stoichiometry ranging from approximately 0.5 to 0.85; the delithiation reaction can extract up to ∼80% of the inserted lithium-ions. In this polymorph, lithium ions are accommodated within interlayer sites. While these results confirm the feasibility of lithium insertion, they do not fully clarify the site occupancies or lithium-ion diffusion pathways—some insights on site occupancies have been obtained through electrochemical insertion studies.^477,478^

**Figure 59:**
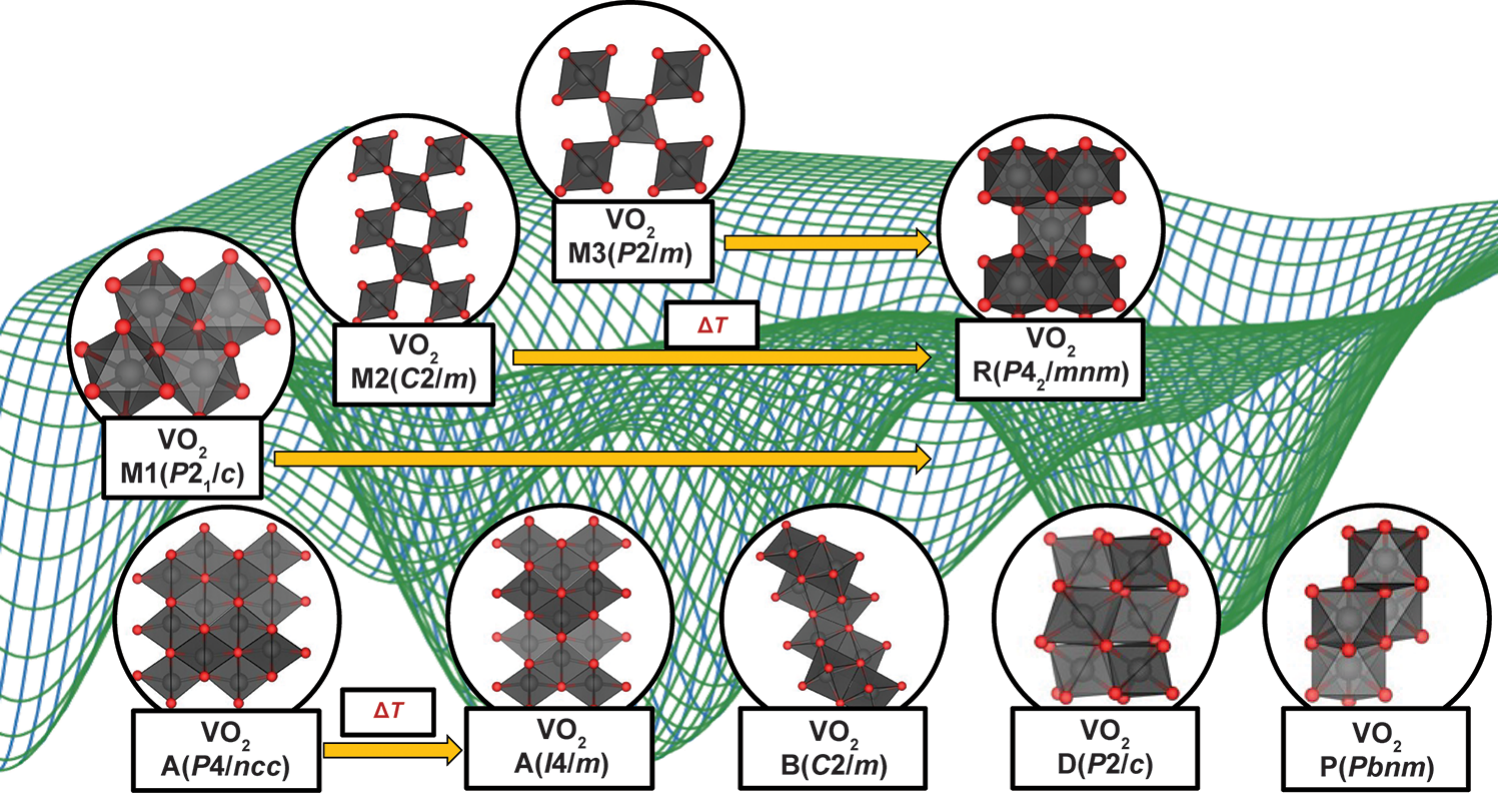
Crystal structure representation of accessible vanadium­(IV) oxide metastable polymorphs such as VO_2_ (B), VO_2_ (A), VO_2_(M), VO_2_ (R), VO_2_ (D), and VO_2_ (P). The various monoclinic VO_2_ polymorphs can undergo a reversible metal–insulator transition (ca. 67 °C) to the tetragonal VO_2_ (R) phase. VO_2_ (A) also has a structural transformation from *P*4/*ncc* to *I*4/*m* at high temperatures (∼162 °C). This figure was reproduced with permission from reference 8.

Beyond conventional topochemical cation intercalation into VO_2_, hydrogenation has emerged as a new strategy for tuning functional properties of VO_2_, based on insertion of protons within interstitial sites where they can be readily accommodated.^479^ For example, Chen et al. demonstrated a simple yet effective approach to insert hydrogen into the monoclinic VO_2_(M) polymorph, achieving controlled modification of its insulator-to-metal transition behavior.^480^ They explored the mechanism of proton insertion into multiple sites on the surface of VO_2_ by computing adsorption energies (Figure 60a). Hydrogen incorporation alters the electronic band structure, as revealed by partial density of states calculations (Figure 60b), and induces a reversible two-step insulator–metal–insulator phase transition. These authors posit an interface between doped and undoped regions of the crystal lattice (Figure 60c). Notably, the process can be further facilitated by introducing low work function metals such as Cu or Al, which facilitate a codoping mechanism reminiscent of proton-coupled electron transfer (Figure 60d).^480^ In this process, the metal first donates electrons to the VO_2_ interface, which subsequently attract protons from the surrounding electrolyte to maintain charge neutrality. Hydrogen insertion initiates at the surface, inducing the initial phase transition, and progressively propagates through the bulk, resulting in uniform hydrogen interstitial occupancy throughout the material.

**Figure 60:**
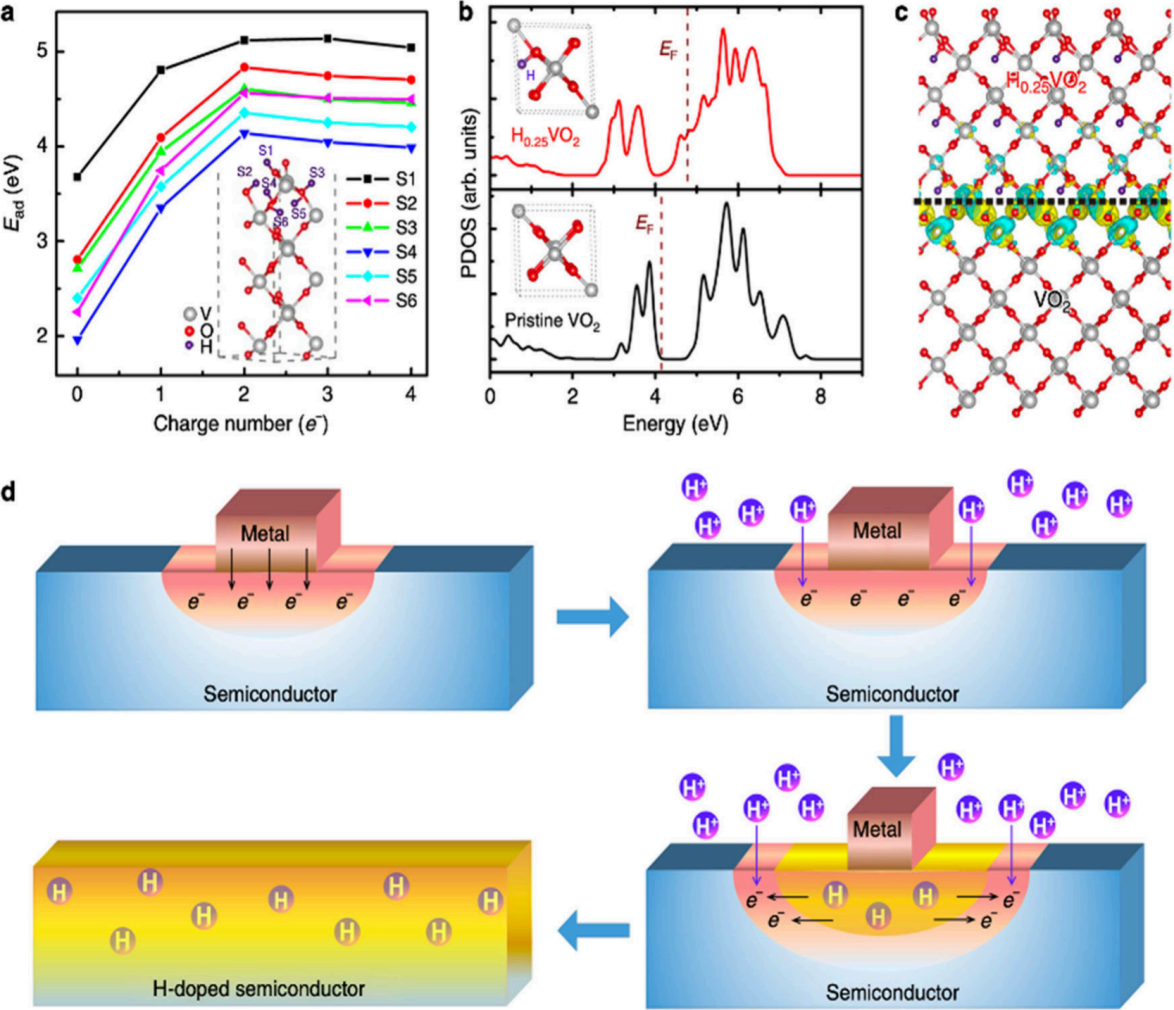
Description of the electron–proton codoping mechanism of proton insertion in VO_2_ (M). (a) Computed adsorption energies for a proton to six adsorption sites of the VO_2_ (020) surface, showing a trend of increasing energy with increasing doped electrons. (b) V-3*d* partial density of states (pDOS) showcasing the change in the semiconductor bandgap to a zero-energy gap state in the H-doped H_0.25_VO_2_ as compared to pristine VO_2_. (c) Computed differential charge distribution at the H_0.25_VO_2_/VO_2_ interface which highlights an ∼2.06e^–^ donation from each H_0.25_VO_2_ supercell to undoped VO_2_. Green and yellow bubbles represent hole and electron charges, respectively. (d) Schematic illustration of the electron–proton codoping mechanism with the low work function metal-acid treatment. This figure was reproduced with permission from reference 480. Copyright 2018 Springer Nature.

Vanadium­(IV) oxide, VO_2_(B) has been employed as a cathode material for Li-ion insertion and affords a specific capacity of 325 mA h g^–1^. However, its performance particularly in terms of capacity and cycling stability—lags behind that of other vanadium oxides such as V_2_O_5_ and V_6_O_13_.^476^ In a study by Liu et al., single-crystalline VO_2_(B) nanowires were used to investigate lithium-ion insertion pathways within the layered structure (Figure 61).^481^ The authors find a reversible phase transition to a lithium-rich Li_*y*_VO_2_ phase during discharge (Figure 61a,b). However, VO_2_(B) is known to undergo an irreversible phase transition at elevated temperatures, which contributes to cathode degradation. This limitation has prompted interest in alternative VO_2_ polymorphs, such as VO_2_(M) (*P*2_1_/*c*), which undergoes a reversible phase transition to VO_2_(R) (*P*4_2_/*mnm*) at high temperatures.^8,266,475,482^ Consequently, several studies have investigated the electrochemical lithiation performance of both phases.^476,481,482^ The VO_2_(R) phase demonstrates approximately 70% higher lithium-ion capacity accommodating up to 0.67 Li per formula unit compared to 0.42 Li per formula unit in VO_2_(M).^482^ Figure 62 presents simulations of the interstitial Li-ion sites within each crystal structure. Figure 62a,b shows the simulated pathways for Li-ion diffusion within VO_2_(M) and VO_2_(R) respectively, highlighting the interstitial sites within which Li can be accommodated. Figure 62c,e shows the density of states result for VO_2_(M) and VO_2_(R), respectively, which highlight the bandgap observed in the VO_2_(M) phase and the lack thereof in VO_2_(R). Figure 62d,f shows the evolution of the lattice parameters as Li-ions are incorporated into the structure. Finally, in Figure 62g,h there are two unique Li sites highlighted in each structure, denoted as 4c and 4d.

**Figure 61:**
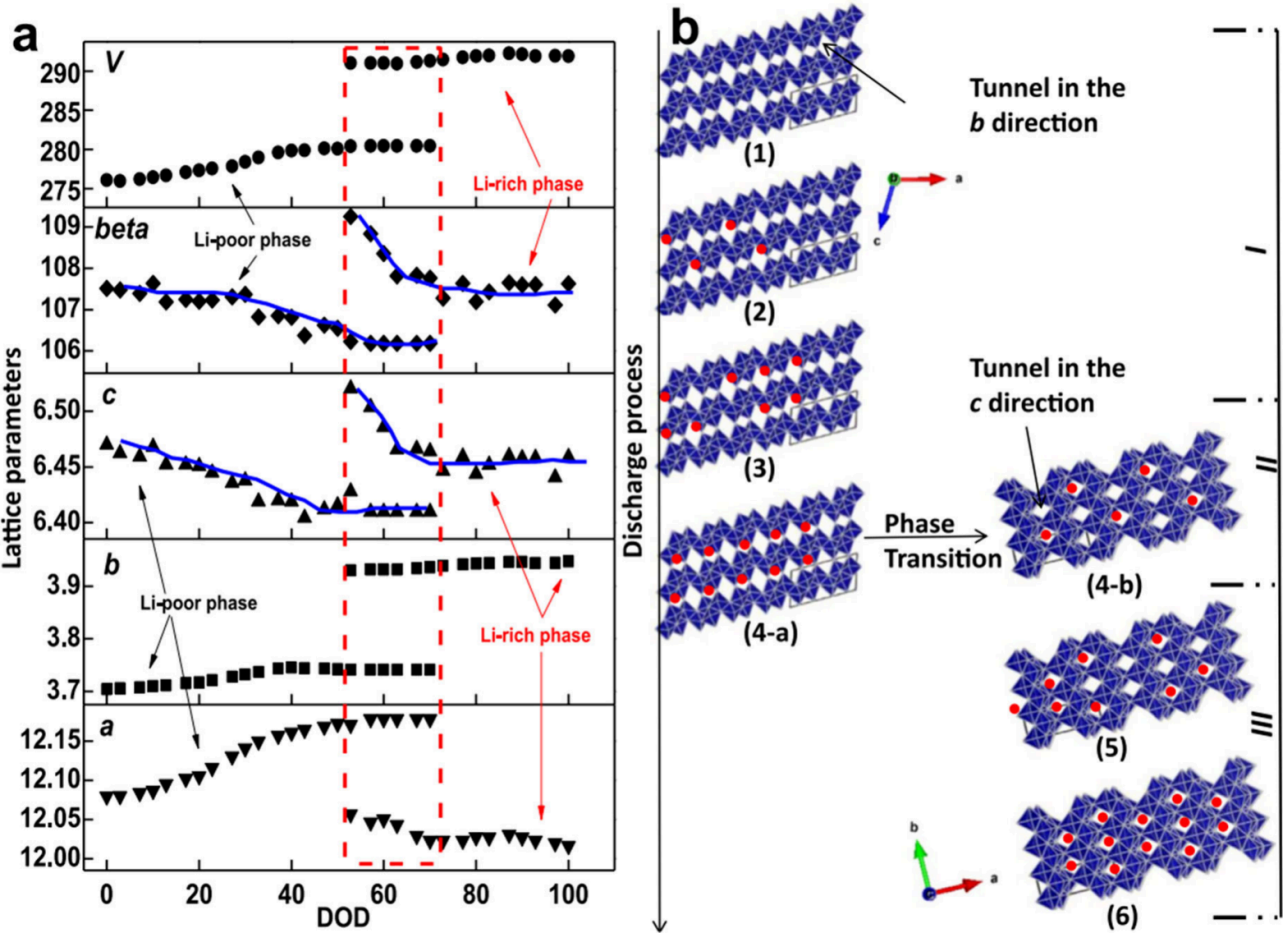
*In situ* synchrotron high energy X-ray diffraction (HEXRD) study on the evolution of VO_2_ phases upon lithiation/discharge. (a) Lattice parameters as a function of depth of discharge (DOD) on the first discharge cycle for cells prepared with VO_2_ (B), determined from refinements of *in situ* diffraction data. (b) Schematic illustration of Li-ion insertion in a single crystal VO_2_ (B) lattice. This figure was reproduced with permission from reference 481. Copyright 2017 Elsevier.

**Figure 62:**
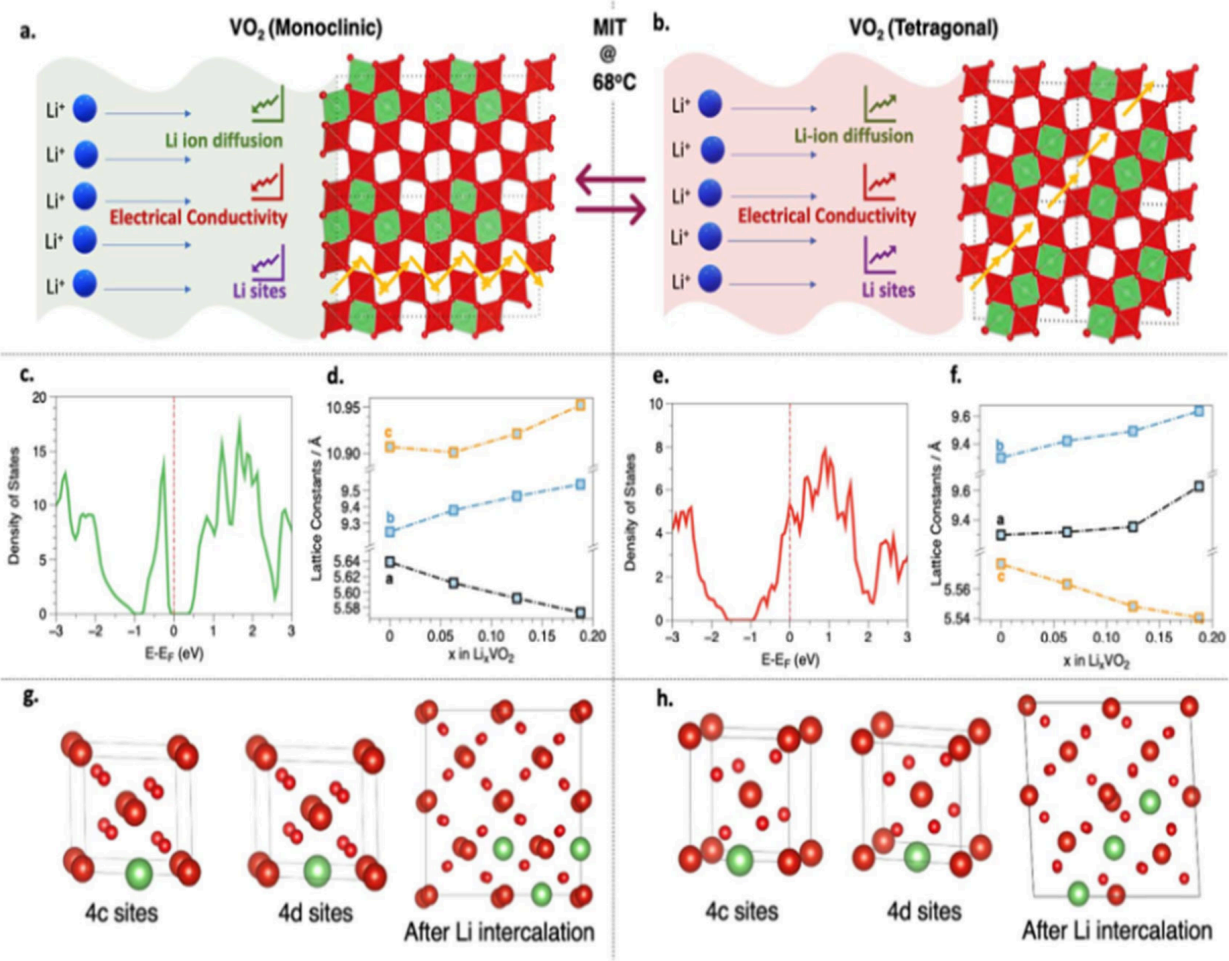
Crystal structure simulations for (a) VO_2_(M) and (b) VO_2_(R) with Li-ion diffusion pathways. Panels (c) and (e) show density of states for VO_2_(M) and VO_2_ (R) respectively. Panels (d) and (f) show the variation in the lattice constants as a function of Li concentration. Panels (g) and (h) show potential sites to accommodate Li-ions in the crystal lattice of VO_2_(M) and VO_2_(R), respectively. Figure was reproduced with permission from reference 482. Copyright 2024 Royal Society of Chemistry.

### 5.4 V_6_O_13_

Within the Wadsley–Roth family of compounds, structural complexity has limited the exploration of many vanadium oxide bronzes in both single-crystalline and polycrystalline forms. A notable exception is V_6_O_13_, which has emerged as a model system for studying ion intercalation and redox behavior due to its well-resolved and functionally rich structure.^155,483–485^ V_6_O_13_ crystallizes in the monoclinic system (space group *C2/m*) and adopts a perovskite-derived framework with distorted VO_6_ octahedra as the fundamental building block. These octahedra are connected through both edge- and corner-sharing, forming alternating single and double octahedral layers (Figure 63a). Within the single layers, vanadium atoms (V1) reside in asymmetric coordination environments, whereas the double layers contain crystallographically inequivalent vanadium sites (V2 and V3). These layers are stacked along the *c* axis and are further interconnected by corner-sharing oxygen atoms, forming a fully three-dimensional (3D) framework. Notably, the structure incorporates well-defined channels aligned along the *b* axis, which facilitate the diffusion and accommodation of guest ions. The presence of mixed-valence (V^4+^/V^5+^) vanadium centers and the open, tunnel-like architecture enable efficient topochemical insertion of various cations. These characteristics collectively render V_6_O_13_ a highly attractive candidate as an energy storage material where ion transport and redox activity are critical to performance.^486,487^

**Figure 63:**
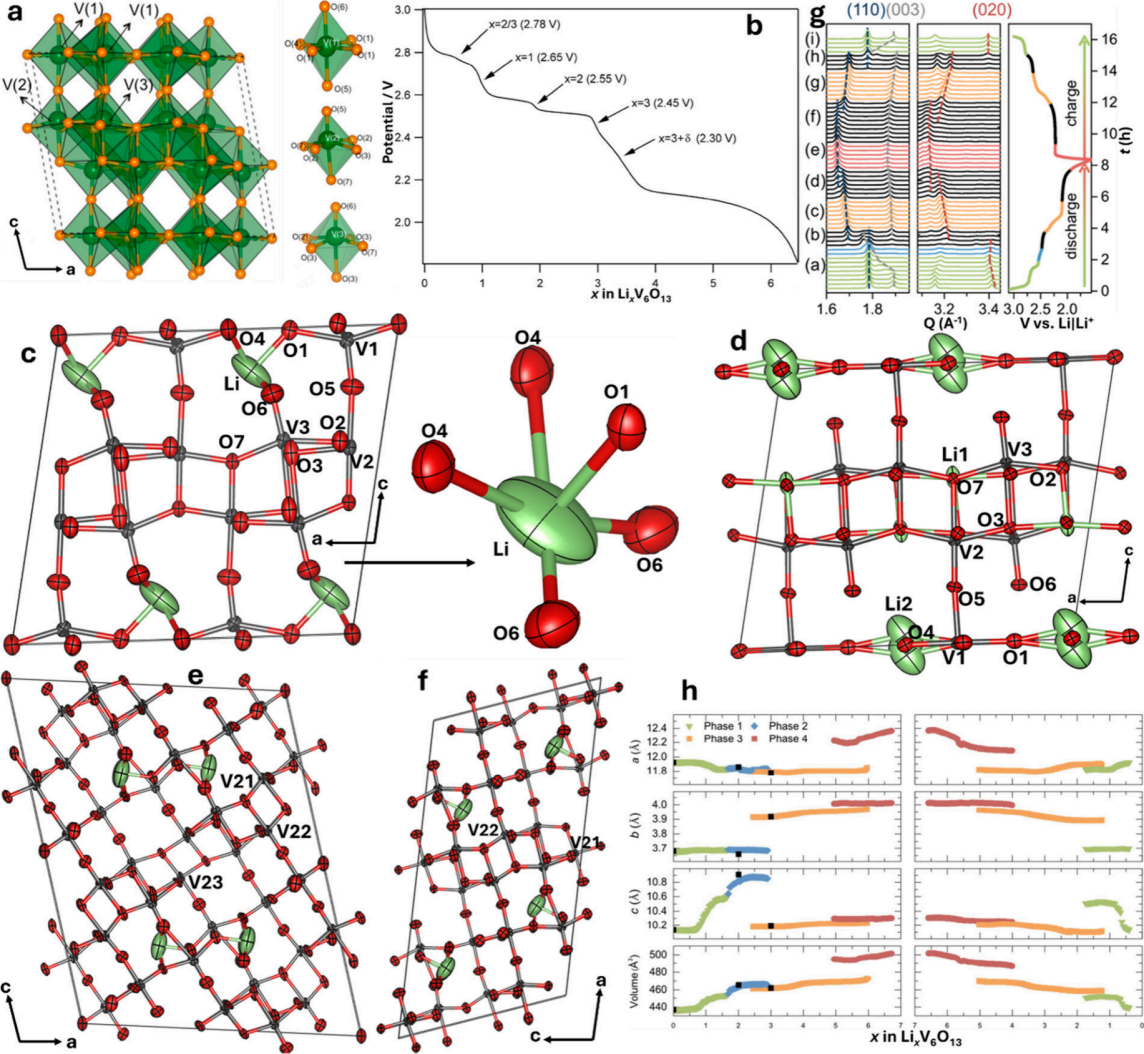
(a) Crystal structure of monoclinic V_6_O_13_ (*C*2/*m*). (b) Discharge curve for V_6_O_13_ versus Li/Li^+^. (c) Structure of Li_2_V_6_O_13_ and the square-pyramidal coordination of the lithium ion. (d) Structure of Li_3_V_6_O_13_. (e) Crystal structure of Li_2/3_V_6_O_13_viewed along the *b* axis, showing three VO_6_ octahedra in Li_2/3_V_6_O_13_. (f) Crystal structure of LiV_6_O_13_ viewed along the *b* axis. (g) *In situ* synchrotron X-ray diffraction of the first discharge and charge cycle of V_6_O_13_. (h) Evolution of unit cell volumes and phase fractions (χ_i_) of the four observed phases during the first discharge (left) and charge (right) cycle of V_6_O_13_. Panel (a) was reproduced with permission from reference 495. Copyright 2017 American Chemical Society. Panel (b) was reproduced with permission from reference 493. Copyright 2004 International Union of Crystallography. Panel (c) was reproduced with permission from reference 492. Copyright 1997 International Union of Crystallography. Panel (d) was reproduced with permission from reference 491. Copyright 1998 International Union of Crystallography. Panels (e) and (f) were reproduced with permission from reference 494. Copyright 2001 International Union of Crystallography. Panels (g) and (h) were reproduced with permission from reference 495. Copyright 2017 American Chemical Society.

The lithium-ion insertion behavior of V_6_O_13_ has been extensively studied, with early systematic work by Murphy et al. employing *n*-butyllithium as a lithiation agent alongside electrochemical cells to investigate lithiation-induced structural transformations in polycrystalline V_6_O_13_.^488–490^ Building on these foundational insights, later research extended these efforts to single-crystalline forms of V_6_O_13_. Investigations on single-crystals of V_6_O_13_, by Bergström et al. using electrochemical insertion, revealed crystallographic anisotropy in lithium-ion diffusion, underscoring the critical influence of structural orientation on Li-ion transport kinetics.

Bergström et al. provided critical insight into the lithium intercalation mechanism in electrochemically lithiated V_6_O_13_ single crystals through single-crystal diffraction studies, elucidating the structural evolution of lithium-rich phases such as Li_2_V_6_O_13_ (Figure 63b) and Li_3_V_6_O_13_ (Figure 63c).^491–493^ In Li_2_V_6_O_13_, lithium ions occupy a five-coordinated square-pyramidal site between layers upon lithiation. This results in a 7.6% expansion along the *c* axis, perpendicular to the layers, accompanied by elongation of the V(1)–O(6) bond and contraction of the V(1)–O(5) bond, which reflects displacement of the V1 atom as a result of specific Li–O–V interactions. In the case of Li_3_V_6_O_13_, a contraction along the *c* axis is observed, concomitant with a 5.6% expansion along the *b* axis. Two distinct lithium sites can be discerned: the first is a fully occupied five-coordinate square pyramidal site with square-pyramidal oxygen coordination within the double layer, akin to that in Li_2_V_6_O_13_, whereas the second is a 4i site within the single layer, which exhibits a distorted planar quadratic coordination geometry.

In contrast, Björk et al. employed a coffee-bag-type electrochemical cell to achieve controlled lithiation of V_6_O_13_ single crystals, accessing low-lithiation phases, Li_0.67_V_6_O_13_ (Figure 63e) and LiV_6_O_13_ (Figure 63f) by carefully controlling temperature to modulate intercalation kinetics.^494^ The V_6_O_13_ framework is largely preserved, retaining its original single and double edge-sharing VO_6_ layers connected by corner-sharing VO_6_ octahedra. Li-ions occupy 5-fold-coordinated tetragonal pyramidal sites in this structure. Structural analysis reveals an increase in V–O bond length associated with vanadium reduction, particularly in chemically equivalent but crystallographically distinct vanadium atoms. For instance, in Li_2/3_V_6_O_13_, V(23)–O(53) shows elongation as a result of vanadium reduction, whereas in LiV_6_O_13_, V21 retains the same elongation. In contrast, V13 and V11 in Li_2/3_V_6_O_13_ and LiV_6_O_13_, respectively, transform into a square pyramid as a result of coordination changes and reduction of the V atom.

Furthermore, Meng et al. employed in situ synchrotron X-ray diffraction in conjunction with electrochemical charge/discharge profiling to elucidate the structural transformations associated with lithium-ion (de)­insertion in V_6_O_13_.^495^ Their results revealed an asymmetric six-step discharge and five-step charge process (Figure 63g, right). *In situ* XRD patterns collected during a C/10 galvanostatic cycle (Figure 63g, left) highlight the behavior of key reflections, (110), (003), and (020), which serve as markers of structural transformations. Continuous shifts of the reflections observed in regions (a), (c), (e), (g), and (i) correspond to a single-phase interstitial solid solution mechanism. In contrast, regions (b), (d), (f), and (h) manifest the disappearance and appearance of reflections, which is characteristic of a two-phase nucleation and growth mechanism. This alternating pattern of phase evolution delineates a sequence of transitions through four distinct phases as shown in Figure 63h. Upon lithium insertion into Phase 1 (V_6_O_13_), the material undergoes a 3.9% volume expansion, reaching approximately Li_1.7_V_6_O_13_, accompanied by a voltage drop from 2.7 to 2.5 V. At *x* ≈ 1.7, a sharp 2.7% volume increase indicates a second-order phase transition between Phase 1 (V_6_O_13_) and Phase 2 (Li_1.7_V_6_O_13_). This is followed by a first-order transition from Phase 2 (Li_1.7_V_6_O_13_to Phase 3 (Li_2.1–3.0_V_6_O_13_) over the composition range *x* = 2.1–3.0, characterized by a voltage plateau at ∼2.45 V, indicative of a two-phase coexistence. Phase 3 (Li_2.1–3.0_V_6_O_13_) remains volumetrically stable until *x* ≈ 5, beyond which a second first-order transition is initiated. Between *x* = 4.8 and 6.2, the emergence of Phase 4 (Li_4.8–6.2_V_6_O_13_) is marked by a pronounced volume discontinuity and a corresponding flat voltage plateau at ∼2.10 V. Upon full delithiation, Li_6.7_V_6_O_13_ reversibly transforms to Li_0.4_V_6_O_13_, traversing three single-phase solid solution regimes.

### 5.5 Other Wadsley–Roth/Magnéli Phases

V_3_O_5_, as shown in Figure 64a, has a 3D open-framework structure comprising two types of VO_6_ octahedral chains along the *c* axis: one chain formed by edge-sharing octahedra along the *b* direction and the other by corner-sharing octahedra in the *ac* plane.^496^ The interstitial sites available for cation intercalation are mainly located within the open framework created by the VO_6_ octahedra.^487^ The extended 3D chain structure of V_3_O_5_ facilitates effective cation intercalation and deintercalation. Despite its favorable architecture, stabilizing the V_3_O_5_ phase has proven challenging, whether through traditional solid-state routes or by carefully tuning vanadium and oxygen stoichiometry in solution-based methods.^496^ Nagasawa et al. reported that V_3_O_5_ often coexists with V_4_O_7_ in single crystals grown using TeCl_4_.^155^ Nevertheless, Yu et al. successfully synthesized V_3_O_5_ microcrystals via vacuum calcination and were the first to demonstrate their application as an anode material for lithium-ion batteries.^487^ Importantly, they observed no significant structural changes upon Li-ion intercalation, which was attributed to be the key factor underpinning the excellent rate capability and cycling stability observed for this material.

**Figure 64:**
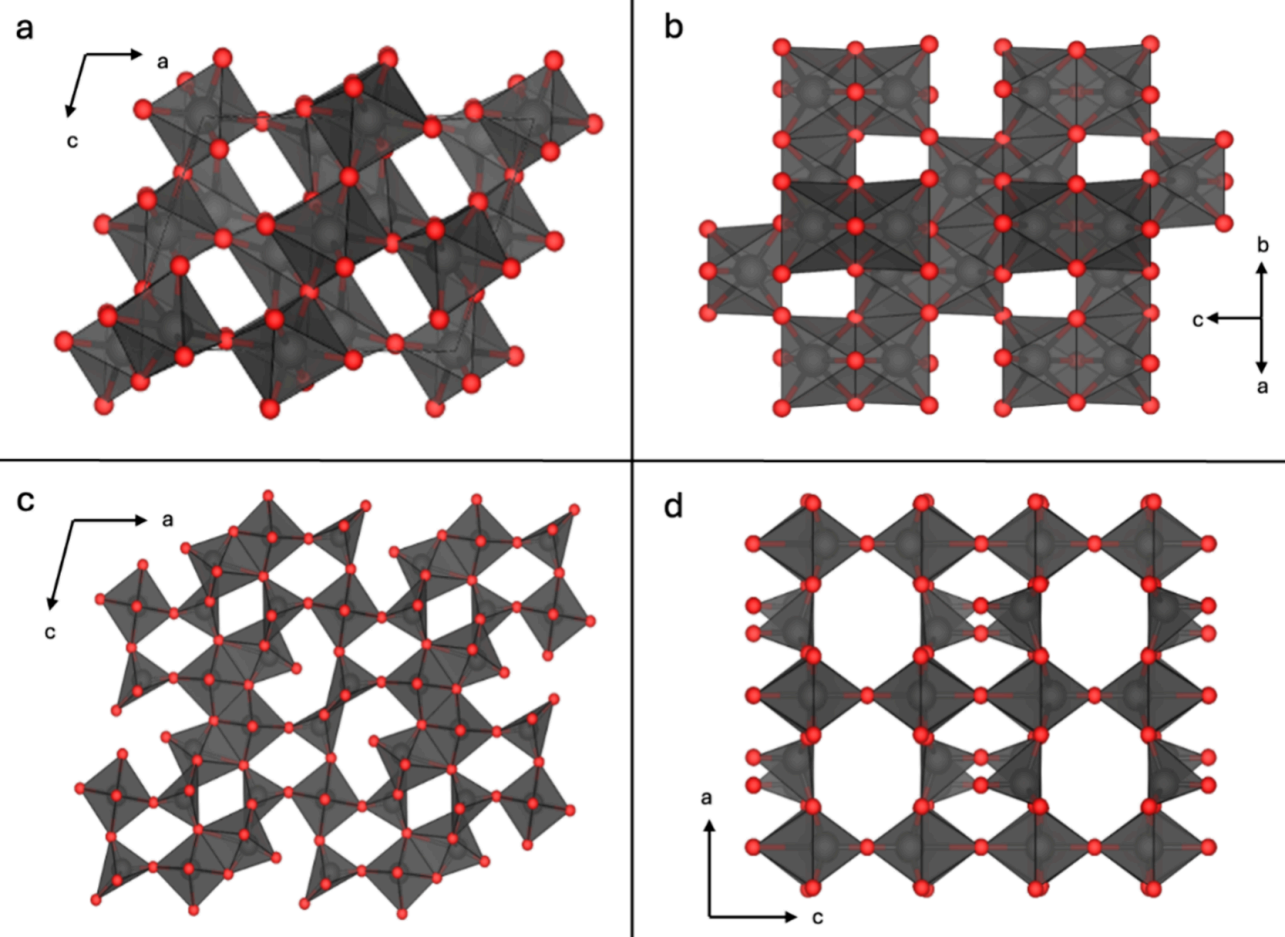
Structures of (a) V_3_O_5_, (b) V_2_O_3_, (c) V_3_O_7_, (d) V_4_O_9_ viewed along (010), (110), (010), and (010), respectively.

V_2_O_3_ adopts a corundum-type hexagonal structure characterized by a three-dimensionally connected framework as shown in Figure 64b. In this configuration, vanadium atoms are octahedrally coordinated by oxygen, forming a dense, close-packed lattice that lacks the large interstitial voids commonly found in tunnel-type materials. Although the available interstitial space is limited, the structural connectivity nevertheless allows for ion transport and intercalation, making V_2_O_3_ a promising candidate for alkali metal-ion storage with a high theoretical capacity. For instance, Zhu et al. demonstrated the electrochemical intercalation of Zn^2+^ ions into V_2_O_3_. The Zn|Zn­(CF_3_SO_4_)_2_||V_2_O_3_ cell delivered reversible maximum capacity of 196 mAh g^–1^ at 0.1 A g^–1^ current density. When the specific current returns to 0.5 A g^–1^, a specific capacity of 163 mAh g^–1^ is restored, thus demonstrating the electrochemical reversibility of the Zn|Zn­(CF_3_SO_4_)_2_||V_2_O_3_ cell.^486^ Despite its potential, V_2_O_3_ faces challenges such as poor electronic conductivity and significant volume changes during prolonged electrochemical cycling.^497^ To mitigate these issues, several strategies have been proposed, including nanoscale structuring, elemental doping, and the incorporation of conductive composite frameworks.^486,497–499,499–501,501–504^

The V_3_O_7_ structure is composed of VO_6_ octahedra and VO_5_ polyhedra, which are linked by corner and edge sharing to form a three-dimensional framework. The unit cell contains 12 formula units of V_3_O_7_, with 12 vanadium atoms octahedrally coordinated to oxygen atoms, 16 within trigonal bipyramids, and eight within square pyramids. The interstitial sites for cation intercalation are primarily located within the tunnels formed by the framework of linked VO_6_ octahedra and VO_5_ polyhedra. These tunnels, oriented along the *b* axis as depicted in Figure 64c, serve as accessible channels for ion insertion.^490,505^ V_3_O_7_ is thermodynamically favorable compared to other Wadsley–Roth phases; this compounds indeed exhibits a lower formation entropy, which reflects its greater inherent stability.^506^ However, electrochemical studies reveal that upon lithiation, a substantial portion of the V_3_O_7_ framework undergoes transformation into V_6_O_13_. This suggests that while V_3_O_7_ is stable under ambient conditions, it is susceptible to phase transitions during electrochemical cycling, particularly upon Li-ion insertion.^505,507,508^

The structure of V_4_O_9_ is made up of three types of VO polyhedra: [VO_5_] pyramids, [VO_6_] octahedra, and [VO_4_] tetrahedra. The [VO_5_] pyramids and [VO_6_] octahedra pair up and connect by corner oxygen atoms from the [VO_4_] tetrahedra. The interstitial sites for ion storage are within the tunnel channels formed by the edge- and corner-sharing VO polyhedra. The presence of these three types of coordination environments yields an abundance of interstitial sites for ion storage.^509^ The tunnels formed by the polyhedral units in V_4_O_9_ create interstitial sites that allow for cation intercalation, as shown in Figure 64d. These tunnels facilitate ion movement and underpin the electrochemical properties of the material. For example, Liang et al. demonstrated efficient Zn^2+^ intercalation into V_2_O_3_ within a zinc-ion battery system. The Zn//V_4_O_9_ cell exhibited outstanding rate capability, delivering 234.4 mAh g^–1^ at 50C, along with a high reversible capacity of 420 mA h g^–1^ at 0.5C and excellent cycling stability.^509^ Mixed-valence V^5+^/V^4+^ yields high electronic conductivity, which enhances ion and electron diffusion through these tunnels. V_4_O_9_, much like V_6_O_13_, can undergo disproportion into VO_2_ and V_3_O_7_.^510^ However, V_4_O_9_ maintains structural stability after electrochemical cycling, reflecting robust structural integrity. Its high capacity, excellent rate performance, and long cycle life make it a highly promising cathode material.

## 6 Surface Defects and Interfaces

The extended ordering of the bulk single crystal enables controlled study of surface facets. The surfaces of single crystals serve as a model for studying initiation of structural transformations, transformation dislocations, facet-dependent reactivity toward surface functionalization, and facet-selectivity of ion insertion reactions. Surface features such as imperfections play a vital role in mediating surface reactivity and in nucleation of displacive and distortive phase transformations.^214,205,26,511^ While the use of epitaxial thin films and single crystals of vanadium oxides as model surfaces to examine phase transformations is underexplored, recent studies have yielded excellent understanding of surface reconstructions and surface defects.^511–513^ Some examples of surface defects include 1D line defects such as edge and screw dislocations; 2D interfacial defects such as grain boundaries and stacking faults; and 3D bulk defects such as pores and cracks. These defects are not static—their mobility and interactions with the ordered single crystal lattice as well as with incoming ion fluxes is of pivotal importance to understanding of ion and electron transport within the material.^514^

The diverse range of composition, electronic structure, and crystal lattices available in vanadium oxides vests great opportunity for tuning of surfaces to mediate reactivity, enable structural transformations, and underpin catalytic transformations. Notably, surface defects play an important role in imparting structural resilience or governing pulverization and failure mechanisms^515^ across charge–discharge cycles during electrochemical cycling.

### 6.1 Mechanics and Interfaces

Surface facets provide a valuable framework for understanding the interplay between structure and ion transport phenomena in V_2_O_5_ systems. Different phases of vanadium oxide single crystals including layered phases such as α-V_2_O_5_ (Figure 65a,b) and γ-V_2_O_5_; and tunnel-structured-phases, such as ζ-V_2_O_5_ exhibit surface facets and topographical defects in an array of structures and features such as terraces, islands, step edges, kinks across different phases as shown in the schematic of Figure 65c.^516^ These surface facets in vanadium oxide single crystals have been observed as naturally occurring as a result of single crystal synthesis or can be artificially engineered through postsynthesis modifications such as electropolishing, focused ion beam etching, or through lithographic patterning. V_2_O_5_ single crystal surface facets may appear on apical faces as shown in Figure 65, lateral faces (due to anisotropy) and rarely basal faces, which may be exposed by re-entrant structures. A complete characterization of the surface facets, boundaries, vertices, angles and lengths obtained through complementary scanning electron microscopy (SEM) and optical imaging provides crucial insights into lithium intercalation- and insertion-induced transformations based on analysis of their topography (Figure 65d,e) and overall morphology (Figure 65b).

**Figure 65:**
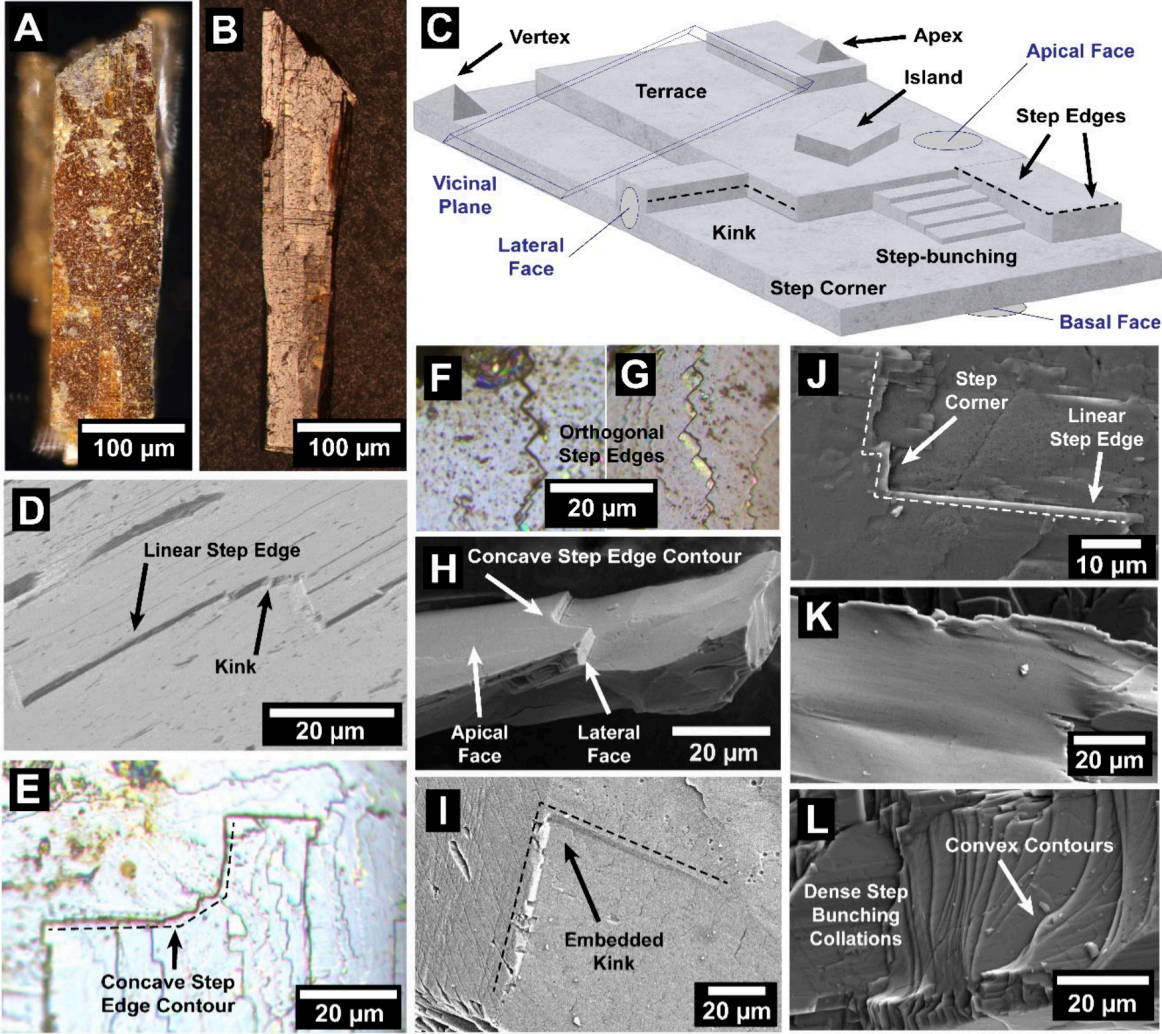
Surfaces of V_2_O_5_ single crystal surfaces. A schematic of the key faces (blue) on a single crystal surface are shown in panel (a) with an illustration of selected topographic surface defects (black). Panel (b) shows the lateral and apical faces of a linear step edge contiguous with a kink visualized through a cross-sectional SEM image in Li-ion-inserted α-V_2_O_5_. Optical imaging of the apical face of the lithium-doped α-V_2_O_5_ single crystal in panel (c) shows the distinct concave step edge contour and additional terraces not visible on the SEM image. Orthogonal step edges shown in panels (d) and (e) with low gamma optical imaging illustrate a series of naturally occurring step corners comprising ordered step edges at 90° angles, which create a staircase appearance on the apical face of an α-V_2_O_5_ single crystal. (f) ζ-V_2_O_5_ single crystal showing terrace surfaces bifurcated by a well-defined discrete step edge. Panels (g) and (h) show the faces of a phase-pure α-V_2_O_5_ single crystal surface. (i) SEM images of electropolished ζ-V_2_O_5_ single crystal surfaces, with a notable embedded kink in panel (j) located within the vicinal plane. SEM images of a (k) smooth pristine ζ-V_2_O_5_ single crystal surface and (l) the occurrence of undulated step-bunching with convex contouring sinuous kinks on a ζ-V_2_O_5_ single crystal surface.

Upon scaling of materials to nanometer-sized dimensions, as a result of the increase in the ratio of surface area to particle volume, surface free energy terms become especially significant and similar in magnitude to bulk free energy differentials.^50,51,10,517^ For instance, metastable polymorphs that can be stabilized with low energy surfaces can be accessed at nanometer-sized dimensions, which corresponds to constrained equilibrium conditions along the free energy landscape rather than kinetic trapping. As such, surface energy as well as strain-effects oftentimes mediated through surfaces constitute vectors along the free-energy landscapes, such as exemplified in Figure 47, and utilized to stabilize polymorphs otherwise inaccessible considering temperature and pressure alone. An important relationship underpinning structural transformations is the contributions to free energy from differentials arising from strain as well as differentials in surface free energy. Considering Mott–Peierls diffusionless phase transformations such as between M1 and R phases of VO_2_, the free energy Δ*G* associated with the transformation is modified by the strain energy *U*_SE_ and the surface free energy *U*_S_ in addition to the chemical free energy *G*_C_.

30 $$\Delta G_{A\to B}=\left(G_{c}^{B}-G_{c}^{A}\right)+\left(U_{\mathrm{SE}}^{B}-U_{\mathrm{SE}}^{A}\right)+\left(U_{S}^{B}-U_{S}^{A}\right)$$

Eq 30 exemplifies numerous approaches through which surfaces can mediate structural transformations through local field enhancements, which can counteract the otherwise dominant role of bulk free energy differentials. For instance, lattice mismatch at quasi-epitaxial interfaces, externally applied strain, and internal chemical strain can be used to modify relative phase stabilities. In addition, such field couplings also modify defect dynamics and can alter ion transport pathways.^54,518^ For insertion reactions, surface defects and local strains modify the kinetics and thermodynamics of ion desolvation/solvation at electrolyte/solid interfaces and solid-state ion diffusion, and as such, can substantially modify nucleation and propagation of insertion-induced phase transformations.^54,468,519^ Imaging with Scanning Transmission X-ray Microscopy (STXM) and analyses with combinatorial Principal Component Analysis (PCA) clustering processes revealed spinodal decomposition with periodically arranged patterns of Li-rich and Li-poor domains^520^ in Li_*x*_V_2_O_5_ nanowires; the degree of lithiation in Li_*x*_V_2_O_5_ was significantly higher in the convex region of a bent V_2_O_5_ nanoparticle, whereas the lowest concentrations of lithium were observed in the linear regions of the V_2_O_5_ nanowire.^521^ Stress mapping in the Li_*x*_V_2_O_5_ systems revealed higher stress levels along domain boundary interfaces corresponding to elastic misfit between Li-rich and Li-poor phases, which highlights the importance of interface character and lattice mismatch in governing phase segregation patterns and resulting stress gradients within battery electrodes. Stresses accumulated within individual particles and particle aggregates result in plastic deformation and crack formation upon prolonged electrochemical cycling.^521^ Such plastic deformation can deteriorate the integrity of the active materials, isolate active material through island formation, exacerbate corrosion and parasitic reactions, and disrupt connectivity to the current collector, thereby resulting in irreversible loss of capacity upon prolonged cycling.^522,523,433^

Inhomogeneities in lithiation and resulting stress accumulation can be ameliorated through architecting V_2_O_5_ single crystal structures to minimize stress-accumulation.^468^ In individual V_2_O_5_, particles kink defects engender residual strains and lithiation inhomogeneities but continuous curvature results in homogeneous lithiation with minimal stress accumulation. Embedded kink-like structures (Figure 65i) and similarly contoured facets (Figure 65h) have been constructed on V_2_O_5_ single crystal surfaces forming well-defined and distinct surface facets, as well as step-edge and step-corner structures in single crystal surfaces (Figure 65j) demonstrating realizable and practical possibilities for architecting electrode surfaces. Lithographic patterning and focused ion beam methods can be used to embed kinks and step corners, such as seen in Figure 65f,g, thereby creating convex and concave facets that reduce strain and surface free energy, ultimately minimizing distortions and lowering the overall free energy.

### 6.2 Single Entity Studies with Single Crystal Particle Systems

A major advantage of single crystal and particle studies is the ability to isolate structural morphological features to understand their effects on lithium intercalation with a clear understanding of the role of anisotropic diffusion pathways and mechanical properties.^523^ During topochemical treatment of α-V_2_O_5_ with *n*-butyllithium, a series of phase transformations take place as a result of the puckering of the V_2_O_5_ crystal structure to charge balance the Li-ions being intercalated. Particle geometry significantly influences lithium diffusivity, with morphologies such as nanowires manifesting inhomogeneous lithiation depending on local curvature and spinodal decomposition patterns, resulting in localized regions of high and low lithium concentration. This variation creates interfacial tension at phase boundaries inducing significant stress-driven cracking and ultimately, cathode pulverization. The homogeneity and rate of lithiation regimes can be examined as a function of morphology (Figure 66a–c), leveraging scanning transmission X-ray microscopy (STXM), which allows for mapping local lithium concentration and to infer stress distributions as shown in Figure 66d.^521^ In a study by Santos et al., it was observed that during delithiation, lithium tends to accumulate around the bend within the particle (Figure 66e), which generates a high stress region shown in Figure 66f on the convex contour of the bend.^521^

**Figure 66:**
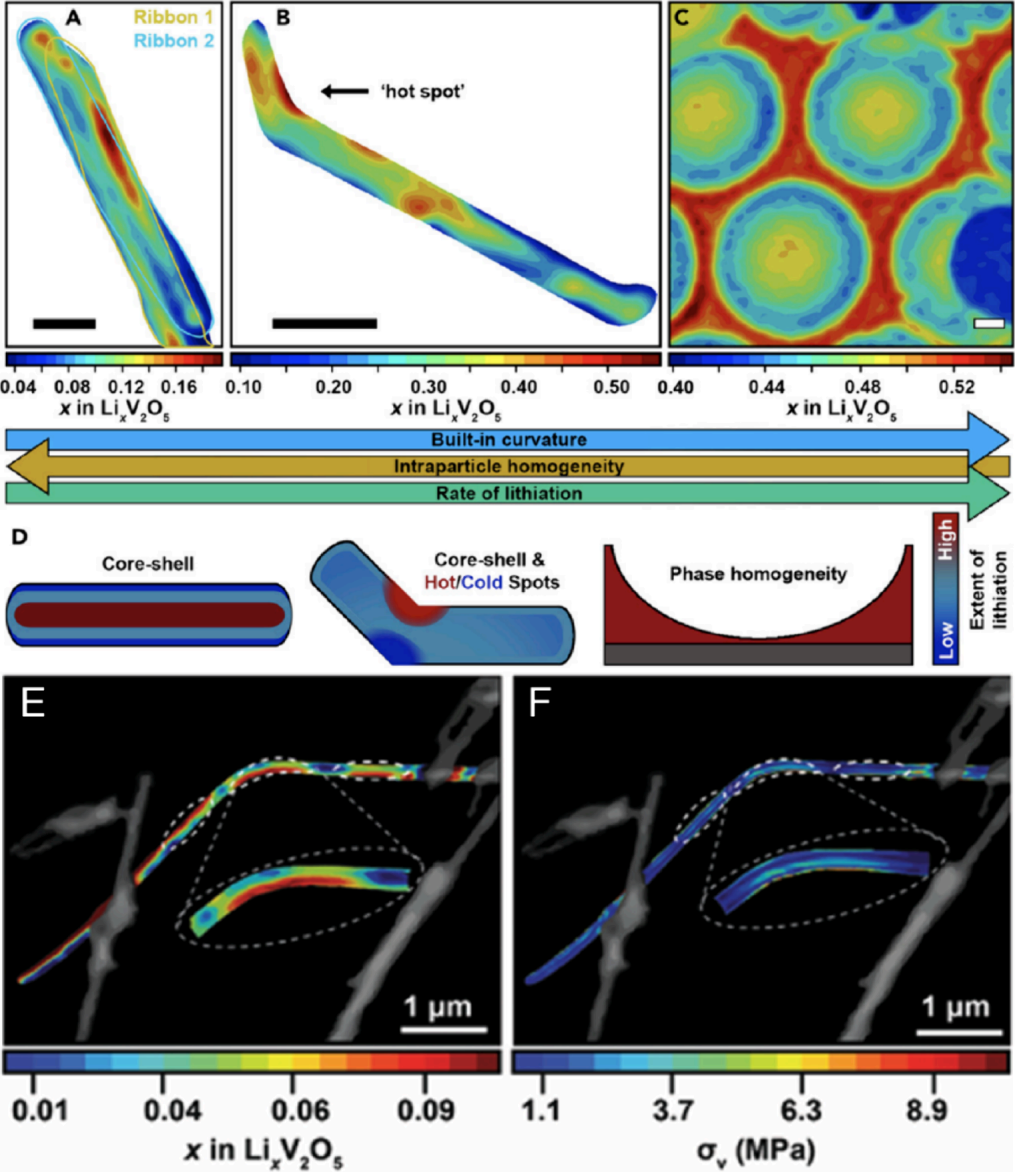
Composition maps generated by PCA-clustered spectra using XANES spectra acquired during STXM experiments for (a) straight nanoribbons, (b) locally curved nanoribbons, and (c) a continuously curved V_2_O_5_ 3D structure with nanobowls. Trends are highlighted by arrows pertaining to rate of lithiation and lithium concentration homogeneity as a function of curvature. (d) Schematic illustration summarizing observed trends resulting from geometry. (e) Lithiation mapping in a V_2_O_5_ particle and a (f) subsequently calculated von Mises stress map. Panels (a)–(d) were reproduced with permission from reference 468. Panels (e)–(f) were reproduced with permission from reference 521. Copyright 2020 Royal Society of Chemistry. Copyright 2020 Matter.

### 6.3 Solid–Solid Interfaces

The single crystal platform for vanadium oxides offers a wealth of opportunities for investigating solid–solid interfaces such as dislocations, twinning faults, grain boundaries, and phase boundaries. As such, single crystals are an excellent model system for interrogating electron, ion, and phonon transport across a diverse array of possible interfaces. These defects play a crucial role in mediating transport phenomena and modulating the effective conductivity of the material.

Grain boundaries serve as crucial solid–solid interfaces in crystalline vanadium oxide systems, offering a powerful means to regulate interconnectivity and enhance performance in energy storage materials. While their exact role remains under investigation, the density of grains and grain boundaries significantly influences key electronic properties such as electronic conductivity. For example, Niang et al. synthesized crystalline VO_2_ thin films via atomic layer deposition and analyzed the resistivity ratio of the films’ crystalline VO_2_ grains. Their study revealed that reducing VO_2_ grain size, thereby increasing grain boundary density resulted in enhanced conductivity.^524^ A notable example corresponds to twin grain boundaries, which represent a unique case where bond lengths remain conserved and maintain coherence across both sides of the interface. Such grain boundaries are relatively low in energy as compared to other configurations. Zhang et al. modeled a twin grain boundary in crystalline VO_2_ using first-principles DFT methods.^525^ They found that grain boundaries have unpaired “dangling” V sites, and when these sites are all paired, the VO_2_ crystal is semiconducting; as such the crystal becomes less conductive when the grain boundary coherency is improved. The interfacial properties from which grain boundaries attenuate ion and charge transport have substantial ramifications for thin films and polycrystalline materials, as well as higher-order interfaces such as interlocking grain surfaces with step-bunching (Figure 65l). Such interfaces exert considerable control over charge transport as well as for thermal transport, which sets up the phase lag critical for electrothermal neurons discussed in Section 4 and determining their resilience when active elements are used to establish self-sustaining oscillations.

In layered vanadium oxide crystal structures, such as α-V_2_O_5_ and γ′-V_2_O_5_, the stacking of layers can lead to stacking faults when disruptions occur within the structure. The classification of these faults depends on whether a layer segment is inserted (intrinsic stacking fault) or missing (extrinsic stacking fault). The extended presence of these stacking faults can significantly influence ion diffusivity as well as phonon transport. Dislocations including screw, shear, and edge dislocations, are characterized by their magnitude and direction as defined by the Burgers vector. These structural defects can significantly impact ion transport pathways, potentially leading to phenomena such as ion “locking,” where ions migrating between layers become trapped at dislocation termini.^526^ Barringer et al. employed Bragg coherent diffractive imaging coupled with optical imaging to study dislocations in V_2_O_5_ single crystals. Their study revealed that, regardless of the phase-fitting method used, the nanoscale defect structures in V_2_O_5_ crystals were highly intricate, comprising a multitude of edge and screw dislocations. These intricate defect networks significantly influence the structural properties of the material, modulate ion transport dynamics, and modify electrochemistry–mechanics coupling.^527^ Notably, hierarchical patterns of defects patterned onto single crystals provide a means of accessing mechanically interlocked stress-resilient architectures that can enable deterministic lithium-ion diffusion, minimize stress gradients, and localize damage (Figure 66a). Single crystals thus provide a means of examining orientationally resolved coupling of diffusion pathways and directional stresses as modulated by the intrinsic crystal structure and extended defects. As vanadium oxides are developed for energy storage applications, design principles gleaned from studies of single crystals can be exploited to design particle, porous electrode, and thin film topologies, morphologies, and packing geometries to obtain desired structures with optimal ion transport and resilience toward chemo-mechanical degradation.

### 6.4 Catalysis with Vanadium Oxides

The surfaces of single crystals such as imaged in Figure 65 can mediate catalytic transformations of molecules in the vapor or liquid phase based on the multielectron redox at vanadium sites, adsorption of molecular species to specific surface facets, and ion/atom transport along surfaces. Site-selective modification of vanadium oxides through substitutional alloying and preintercalation enables modulation of frontier orbital states of relevance to catalytic transformations. Indeed, V_2_O_5_ is an industrial catalyst widely implemented in catalytic converters for deNO_*x*_ and is the primary catalyst for sulfuric acid production by the contact process.^528,506,529,530^ V_2_O_5_ further finds applications in a variety of industrial processes such as the conversion of *o*-xylene to phthalic anhydride, oxidation of alkylpyridines to nicotinic acid, and oxidative degradation of chlorinated hydrocarbons.^530,531^ Recent effort has expanded the scope of vanadium oxide catalysts from environmental and industrial catalysis to energy relevant catalysis such as solar fuel production and CO_2_ reduction.^532,533^ While catalytic transformations mediated by vanadium oxides have been reviewed elsewhere, see for example Wachs and Delfarro and co-workers authoritative reviews on industrial catalysts,^530,534^ compendia from Li and co-workers and Chen and co-workers on vanadium oxide selective catalytic reduction environmental catalysts,^535,536^ and past articles examining progress on vanadium oxide photocatalysts.^12,537,11^ In this section, we briefly examine prospects for single crystals with well-defined facets that can be interfaced with other semiconductors to define photocatalytic architectures. The interfacial structure of semiconductor heterostructures and the resulting thermodynamic band offsets and kinetics of charge transfer are critical to mediating separation of electrons and holes upon photoactivation. In turn, the availability of long-lived electrons and holes delivered at appropriate overpotentials is of pivotal importance to achieving redox catalysis.^11–538^

An expansive and versatile class of photocatalysts pair quantum dots (QDs) such as II–VI semiconductors as light harvesters with vanadium oxide bronzes inserting p-block cations in interstitial sites. The filled *ns* ^2^ states of p-block cations in the fourth, fifth, and sixth periods of the periodic table can yield midgap states above the O 2*p* valence band of binary oxides based on the strength of anion hybridization.^12,537,11^ As sketched in Figure 67a upon photoexcitation, electrons are promoted from the QD valence band to the QD conduction band where the photogenerated electrons can reduce H^+^ to H_2_ or convert CO_2_ into advantageous hydrocarbons with or without the help of a transition metal cocatalyst.^11^ The resulting hole in the valence band can mediate QD photocorrosion in water unless rapidly extracted. Midgap states of *M*_*x*_ *M*′_*y*_V_*z*_O_5_ (where *M* is a *p*-block cation, *M*′ is an *s*-, *p*-, or *d*-block cation, *x* and *y* are their stoichiometries, *z* is the stoichiometry of V^5+/4+^ centers, and V–O connectivity is tunable) can facilitate rapid hole extraction in appropriately designed semiconductor heterostructures. The resulting holes can oxidize water molecules to generate O_2_ or H_2_O_2_. Photocatalytic heterostructures must thus be capable of absorbing sunlight to generate charge carriers; promote efficient charge separation to suppress recombination; deliver electrons and holes at appropriate overpotentials to catalytic sites, and reactant transport between catalytic sites.^539,540^ The thermodynamic energy offsets and kinetics of charge transfer are to a substantial extent modulated by the interfacial structure. Figure 67b shows two distinct approaches of assembling heterostructures either based on a direct interface or through bifunctional linkers. Single crystals with well-defined interfaces thus afford ideal model systems for systematically varying interfacial structure to interrogate modulation of photoexcited charge transfer rates and catalytic performance.

**Figure 67:**
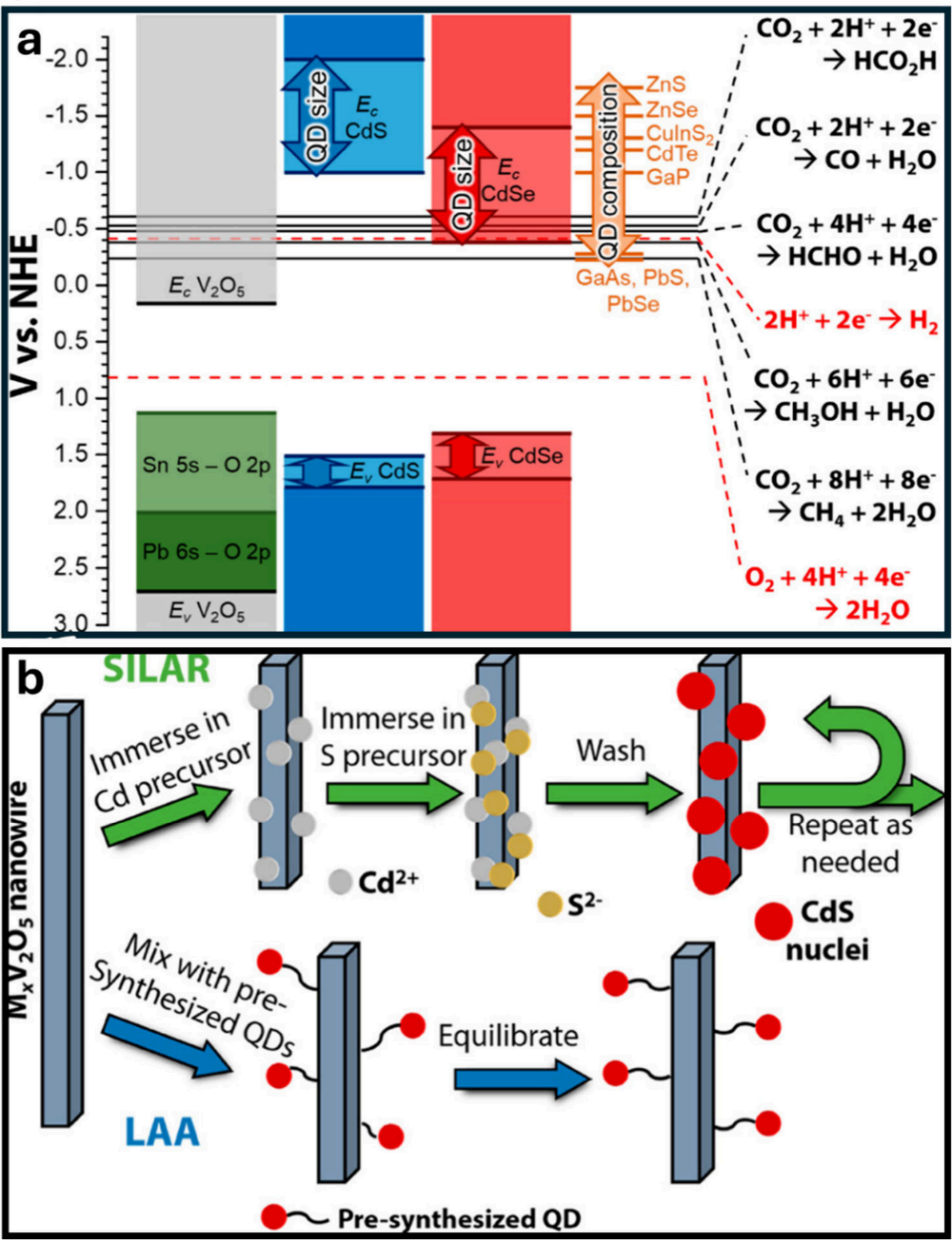
(a) Band structure representation for CdS (blue) and CdSe (red) QDs interfaced with V_2_O_5_ (gray). V_2_O_5_ doped with Pb (dark green) and Sn (light green) introduce midgap states that close the gap in valence bands at the semiconductor–QD interface, facilitating charge transfer mechanisms. Figure was reproduced from reference 11. (b) Representative diagram of the heterostructure interfacial methods SILAR (top) and LAA (bottom) and their respective workflows. Figure was reproduced with permission from reference 11. Copyright 2022 American Chemical Society.

### 6.5 Band Structure Modification for Resonantly Matched Energetic Offsets

First demonstrated with TiO_2_, by Fujishima et al. for photocatalysis,^541^ heterostructures have since become a versatile platform for integrating complementary material properties. Photocatalytic heterostructures consist of two or more closely interfaced materials, typically chosen to exhibit different band structures that facilitate separation.^542,543^ An important characteristic of heterostructures is effective matching of energetic offsets, which enables efficient charge separation and minimizes electron–hole recombination.^11^ This precise tuning of electronic structure is key to enhancing the photocatalytic performance of heterostructures.

Band structure engineering of constituent heterostructures is one common strategy to improve energetic offsets between the materials and improve charge separation efficiency.^11–545^ Vanadium oxide heterostructures interfaced with QDs have shown significant promise for enhancing electron–hole pair separations and delivering them to cocatalysts at low overpotentials.^11^ The distinctive electronic structure of vanadium oxide heterostructures containing p-block cations offers a powerful platform for tuning both the thermodynamic driving forces and kinetic pathways of excited-state charge-transfer processes central to photocatalysis. The energy positioning of p-block cations containing stereochemically active electron lone pairs is tunable within a desirable energy range for enabling hole extraction from QDs in photocatalytic heterostructures.^544,545^ By leveraging p-block cations with stereochemically active lone pairs, it is possible to induce midgap state between the valence and conduction bands of vanadium oxides. The lone-pair-derived midgap states in vanadium oxide heterostructures along with their conduction and valence band edges can be modulated to design photocatalytic architectures with electronic structures that are optimally aligned with redox potentials necessary for water splitting.

Hybridization of the *s*- and *p*-orbitals of p-block cations and the *p*-orbitals of an anion, as per the revised lone pair model by Walsh et al. creates hybrid antibonding states that contribute to the midgap states lying above the valence band maximum.^546^ Photocatalytic heterostructures with optimal band offsets can thus facilitate charge transfer processes while suppressing recombination and enabling catalytic redox reactions.^11–545^ This approach has been implemented in heterostructures incorporating β-Pb_*x*_V_2_O_5_ and β-Sn_*x*_V_2_O_5_ compounds along with CdS and CdSe QDs (Figure 67a).^544,545^

Several interfacial strategies have been explored, including ligand-assisted assembly (LAA) and successive ionic layer adsorption and reaction (SILAR).^547,548^ In LAA, a molecular linker is employed to bridge the QD light harvesters and the vanadium oxide semiconductor. The resulting interfacial distance is defined by the chain length of the linker, introducing spatial separation between the materials. In contrast, SILAR involves the direct growth of QDs on the surface of the vanadium oxide, establishing intimate contact between the two components (Figure 67b).^11,547,548^ These distinct interface architectures have implications for hole transfer kinetics. In SILAR-based systems, the direct interface enables high QD loading and uniform surface coverage, but limits control over QD size and size distribution. Conversely, LAA allows for presynthesis of QDs with tunable size and optical properties, offering finer control over energetic offsets. However, the increased interfacial distance can hinder charge transfer by requiring tunneling or level-crossing mechanisms within donor–bridge–acceptor systems. While LAA facilitates electronic tuning, it often results in lower QD loading and potential barriers to efficient hole transport. Each method presents trade-offs between structural precision, electronic tunability, and charge transfer efficiency.

As a representative example, García-Pedraza et al. investigated β-Pb_0.33_V_2_O_5_ nanowires (NWs) interfaced with Ni-doped CdS (Ni:CdS) quantum dots (QDs) using LAA. In this approach, heterostructures were readily synthesized by reacting β-Pb_0.33_V_2_O_5_ NWs with colloidal cysteinate-capped CdS or Ni:CdS QDs (cys-CdS or cys-Ni:CdS) dispersed in deionized water. The native cysteine ligands on the QD surfaces facilitate attachment to the vanadium oxide substrate under mildly acidic conditions, where the protonated amine group promotes strong electrostatic interaction with the oxide surface. To elucidate the structural characteristics of the resulting heterostructures, García-Pedraza et al. employed high-resolution transmission electron microscopy (HRTEM) along with selected area electron diffraction (SAED). The SAED patterns revealed a bicrystalline interface, confirming the structural integrity and phase preservation of both β-Pb_0.33_V_2_O_5_ and the CdS-based QDs (Figure 68). Scanning electron microscopy (SEM) and Scanning Transmission electron microscopy (STEM) show clear indication of deposited QDs at the surface of the nanowires (Figure 68a,b). Lattice-resolved HRTEM images captured at multiple QD-decorated regions allowed for indexing, using SAED, of distinct diffraction spots to the respective crystal planes of the constituent materials (Figure 68c,d), offering clear visualization and validation of the nanoscale heterointerfaces.^549^ A series of chronocoulometry experiments were performed, which reveal the nature of the charges arising in the as prepared heterostructures (Figure 68e), while subsequent hydrogen evolution was measured with a gas chromatograph. The study shows a significant reduction in H_2_ production with β-Pb_0.33_V_2_O_5_/CdS heterostructures, but a shift from an oxidative to a reductive pathway in Ni-doped β-Pb_0.33_V_2_O_5_/CdS heterostructures (Figure 68f).

**Figure 68:**
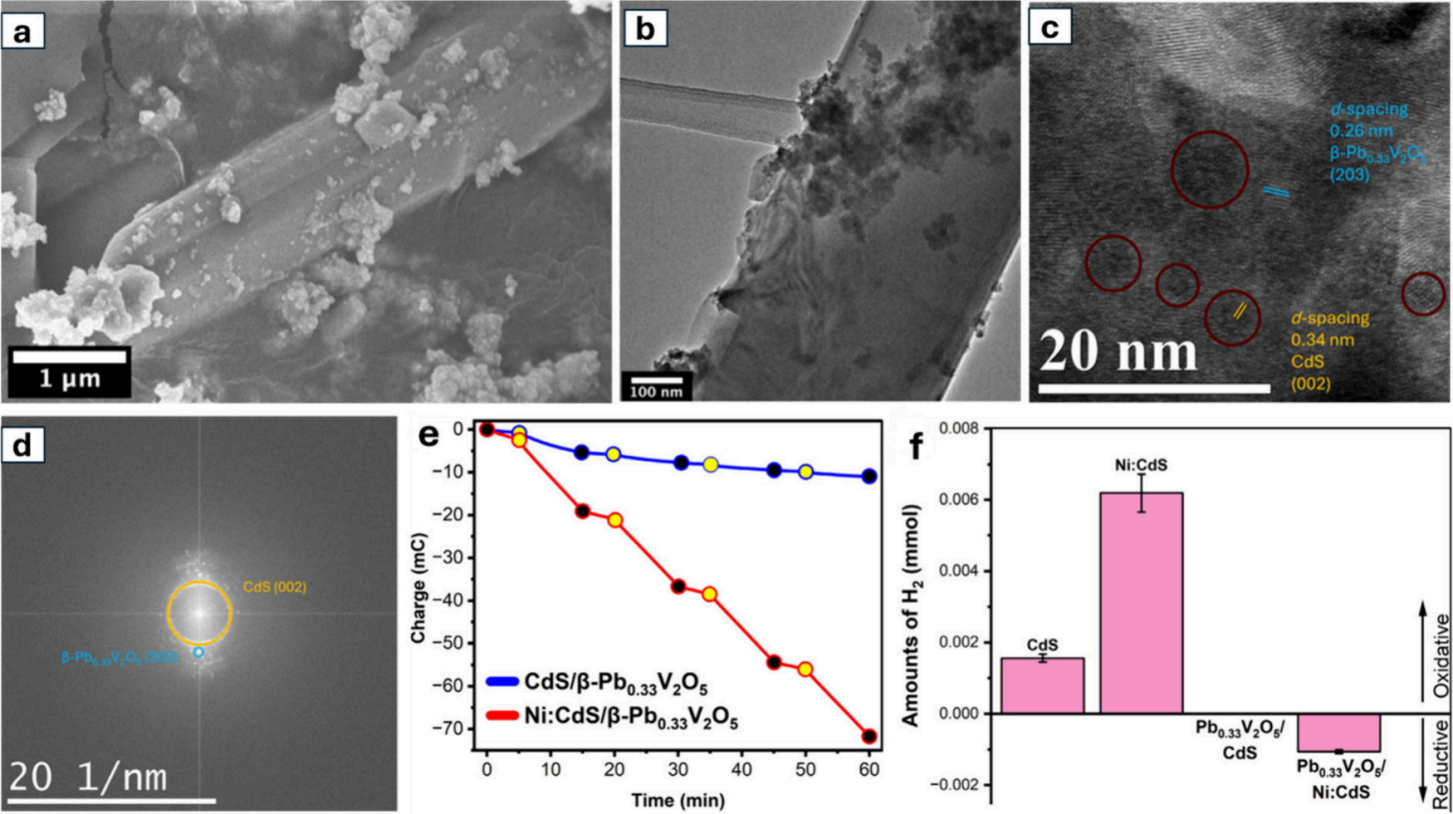
(a–d) Electron microscopy images of Ni:CdS/β-Pb_0.33_V_2_O_5_ nanowire heterostructures: (a) SEM image, (b) STEM image, and (c) HRTEM image. Red circles highlight individual QDs and reveal separation between lattice fringes corresponding to the (002) plane of wurtzite CdS and (203) planes of β-Pb_0.33_V_2_O_5_. (d) SAED pattern with assigned reflections attributed to the lattice fringes in panel (c). (e) Chronocoulometry data for FTO electrodes functionalized with Ni:CdS/β-Pb_0.33_V_2_O_5_ nanowires. (f) Hydrogen evolution resulting from chronocoulometry experiments using CdS, Ni:CdS, and Ni:CdS/β-Pb_0.33_V_2_O_5_ heterostructures. This figure was reproduced with permission from reference 549. Copyright 2024 Nano Research.

Ayala et al. recently investigated the synthesis and interfacial charge transfer characteristics of Sb_2_VO_5_/CdS and Sb_2_VO_5_/CdSe heterostructures fabricated via both LAA SILAR methods. This study aimed to understand how the mode of interfacial connectivity whether direct interfaces assembled by SILAR or interfaces comprising molecular bridges assembled by LAA impacts charge transfer behavior. SEM (Figure 69a), TEM (Figure 69b), HRTEM (Figure 69c–e), and SAED (Figure 69f) were employed to visualize the bicrystalline interfaces formed in each heterostructure type, whereas Raman microprobe spectroscopy served as a diagnostic tool to monitor vibrational modes associated with QD loading (Figure 69g). In the Raman spectra, the characteristic longitudinal optical (LO) and 2LO modes for CdS and CdSe QDs emerged as clear indicators of quantum dot integration, with signal intensity increasing proportionally with the number of SILAR deposition cycles. For example, in Sb_2_VO_5_/CdS heterostructures, higher QD loadings achieved through multiple SILAR cycles (Figure 68g). This reduction suggests limited QD loading or more diffuse interfacial contact when using molecular bridging ligands.^538,550^ The heterostructures showed well-differentiated electron and hole transfer rates, which underpins effective real-space charge separation.

**Figure 69:**
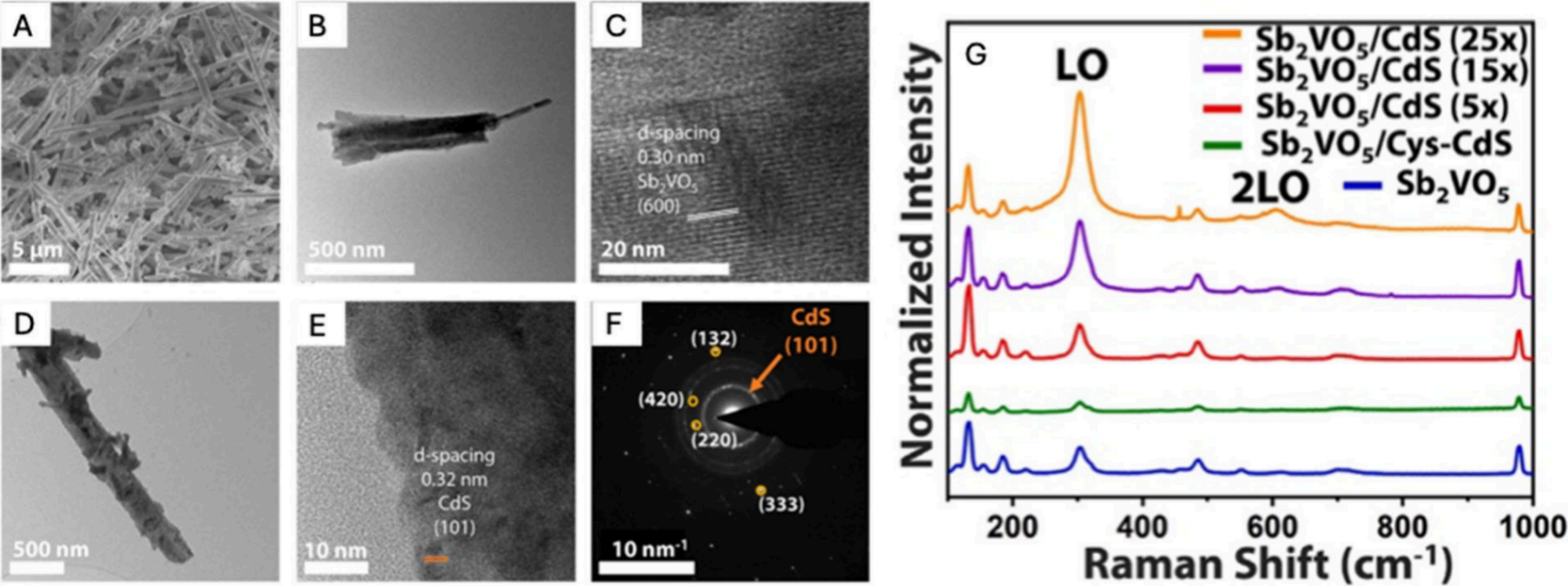
Electron microscopy of Sb_2_VO_5_/CdS heterostructures. (a) SEM image of the synthesized Sb_2_VO_5_ nanorods. (b) Low magnification TEM image of Sb_2_VO_5_ nanorods. (c) Lattice-resolved TEM image of Sb_2_VO_5_ nanorods. (d) Low magnification TEM image of Sb_2_VO_5_/CdS heterostructures. (e) Lattice-resolved TEM image of Sb_2_VO_5_/CdS heterostructures. (f) Indexed SAED pattern of Sb_2_VO_5_/CdS heterostructures with individual spots indexed to Sb_2_VO_5_ rods and the diffuse ring indexed to the (101) planes of CdS QDs. (g) Raman spectra of Sb_2_VO_5_ and Sb_2_VO_5_/CdS heterostructures. The LO and 2LO modes of CdS increase as a function of cycle number in SILAR-derived heterostructures, while the LAA-derived heterostructures show a weak signal. This figure was reproduced with permission from reference 538. Copyright 2024 Elsevier.

The above examples show the remarkable promise of vanadium oxide heterostructures for energy relevant catalysis. However, design principles remain to be refined with regards to details of surface and interface structure. For instance, the facet selectivity of charge transfer rates in M_*x*_M′_*y*_V_*z*_O_5_/QD heterostructures is underexplored. The availability of single crystals affords opportunities for understanding how interfacial structure can be modulated through choice of surface facets and for development of atom-precise quasi-epitaxial interfaces with semiconductors. Single crystals can further enable exploration of alternative functionalization schemes on distinct surfaces to modulate charge transfer dipoles and QD loading. A critical issue that is mostly unexplored is modifications of electronic structure resulting from surface reconstruction. A combination of scanning tunnelling microscopy imaging of surfaces in concert with synchrotron hard X-ray photoemission and inelastic X-ray scattering probes will enable the atom precise design of interface structure as required for precisely engineered photocatalytic architectures.

## 7 Mechanical Properties of Vanadium Oxide Single Crystals

The previous sections have reviewed the structural, electrothermal, and electrochemical characteristics of a rich diversity of vanadium oxide single crystals and examined their diffusionless and ion-insertion driven structural transformations. In this section, we examine the mechanical properties of vanadium oxide single crystals, specifically, hardness, elastic modulus, strength, fracture toughness, and overall stress–strain response with a particular emphasis on V_2_O_5_. We note that the mechanical properties of V_2_O_5_ vary significantly among its polymorphs. The most stable phase under ambient conditions, orthorhombic α-V_2_O_5_ (space group *Pmmn*) (Figure 48a), is highly anisotropic because of its layered structure in which vanadium–oxygen polyhedra create two-dimensional sheets held together by weak van der Waals-like interactions as described in Section 1.2.1 and 1.2.2. Other phases, such as δ-, λ-, γ′-, and ζ-V_2_O_5_, exhibit distinct structural motifs, bonding interactions, and mechanical responses. For instance, γ′-V_2_O_5_ has a distorted layered structure illustrated in Figure 50a; δ-V_2_O_5_ experiences significant volume changes (>11%) during lithium intercalation; and ζ-V_2_O_5_ remains unexplored in terms of its mechanical properties. Additionally, while α-V_2_O_5_ displays pronounced anisotropic behavior because of its well-defined layered structure, polymorphs such as γ′- and ζ-V_2_O_5_ feature more 3D interconnected atomic frameworks.^551–554^

Given these variations, a phase-specific approach is desirable to accurately characterize and compare mechanical properties and stability across the phases. However, obtaining V_2_O_5_ single crystals of adequate size for mechanical testing has been challenging because of their inherent brittleness and tendency to grow in preferred platelet-like geometries. Conventional synthesis methods typically yield crystals of representative sizes of hundreds of nm to μm, necessitating nano-to-microscale testing techniques such as nanoindentation (Figure 71a–e), microcantilever bending (Figure 72a), and micropillar compression, (Figure 72b,c). Although recent advances in crystal growth, such as floating zone melting, have produced larger V_2_O_5_ single crystals, studies on their mechanical properties remain scarce.^555^ Consequently, much of the current understanding is taken from small-scale measurements of single crystals and corresponding comparisons with (larger) polycrystalline samples.

Beyond their fundamental mechanical properties, V_2_O_5_ single crystals are particularly interesting because of the prevalence of their phase transformations when subjected to external stimuli. Indeed, structural transformations induced by pressure, temperature, or ion insertion such as discussed in Section 5 can significantly alter mechanical behavior. For example, in lithium-ion battery applications, lithiation of α-V_2_O_5_ produces phases such as ε-Li_*x*_V_2_O_5_, δ-Li_*x*_V_2_O_5_, and γ-Li_*x*_V_2_O_5_ as illustrated in Figure 48, with each phase transformation inducing volume expansion (or even contraction in some cases), which often generates significant internal stresses that can induce mechanical damage.^330,554^ Similarly, high-pressure studies show that α-V_2_O_5_ becomes amorphous at ∼7 GPa, whereas δ-V_2_O_5_ can crystallize from the amorphous state under specific conditions.^556^ Understanding how these transformations affect mechanical integrity is essential for applications in energy storage, neuromorphic computing, and microelectronics, where structural stability under repeated electrochemical and thermal cycling is critical.

To provide the necessary foundation for understanding such behavior, Section 7.1 briefly introduces essential concepts in solid mechanics relevant to anisotropic single crystals. This brief overview includes definitions of stress and strain, the tensorial nature of mechanical response, and the role of crystal symmetry in determining elastic constants. These principles offer the theoretical basis needed to interpret experimental observations and predict material performance particularly as orientation-specific measurements on large single crystals become accessible.

This review proceeds by systematically examining the mechanical properties of V_2_O_5_ single crystals. Section 7.2 highlights the role of phonon–electron interactions in shaping the mechanical response of single crystals. Section 7.3 focuses on phase-dependent mechanical behavior, while Section 7.4 explores fracture mechanisms and the influence of environmental factors such as temperature and electrochemical operation. Section 7.5 explores the use of nano-to-microscale testing techniques, such as nanoindentation and micropillar compression, to evaluate hardness, modulus, and failure modes. Where relevant, findings from studies on both polycrystalline and amorphous V_2_O_5_ will provide context for understanding the behavior of single crystals. Finally, Section 7.6 highlights research gaps and suggests future directions for studying and enhancing the mechanical properties of V_2_O_5_ single crystals.

### 7.1 Fundamental Mechanical Principles Relevant to Single Crystals

Understanding the mechanical behavior of V_2_O_5_ single crystals requires foundational knowledge of key mechanical concepts such as stress, strain, elasticity, and anisotropy. Engineering stress (σ), e.g., during a uniaxial tension experiment, is defined in eq 31 as the applied force divided by the initial cross-sectional area:

31 $$\sigma =\frac{F}{A_{0}}$$

where *F* is the applied force, and *A*_0_ is the original cross-sectional area. The strain (ε) quantifies the deformation as the relative change in length of the material (e.g., during a uniaxial tension experiment) following eq 32

32 $$\varepsilon =\frac{\Delta l}{l_{0}}$$

with Δ*l* representing the change in length and *l*_0_ the original length. Depending on the loading conditions, stress/strain can be tensile (leading to an increase in a length), compressive (leading to reduction in length), or shear (in which two forces acting parallel to each other across a surface cause the material to deform by shifting layers relative to each other).^557^ Such distinctions are particularly significant for layered structures like V_2_O_5_, where mechanical responses vary with loading direction due to anisotropic bonding.

Mechanical deformation can be broadly classified into elastic and plastic regimes. Elastic deformation is reversible: when the applied stress is removed, the material returns to its original shape and dimensions. In contrast, plastic deformation is permanent: the material does not fully recover its original dimensions/shape once the load is released. In brittle materials such as V_2_O_5_, plastic deformation is typically minimal, and fracture typically occurs shortly after the elastic limit is reached.

In the elastic deformation regime, crystalline materials typically exhibit linear, reversible behavior described by Hooke’s law. For isotropic materials, this relation is given by eq 33.

33 $$\varepsilon_{11}=\frac{1}{E}\left[\sigma_{11}-v\left(\sigma_{22}+\sigma_{33}\right)\right]$$

Here, *E* represents Young’s modulus, a fundamental property describing material stiffness or its resistance to elastic deformation, and ν is Poisson’s ratio, which describes the ratio of the transverse contractional (or extensional) strain relative to the longitudinal extensional strain in the direction of the applied load.^558^ For anisotropic crystals such as V_2_O_5_, the stiffness (i.e., the effective elastic modulus) varies considerably along different crystallographic orientations because of differences in interatomic bonding strength, particularly between covalently bonded layers and weaker interlayer interactions.

A full characterization of mechanical response in three-dimensional space thus necessitates generalizing the stress and strain as second-order tensors, which provide comprehensive information about loading conditions and resulting deformation states at every point within a material as displayed in Figure 70. The stress tensor (σ*_ij_*), provided in eq 34, includes normal stresses (σ_11_, σ_22_, and σ_33_) that act perpendicular to planes and shear stresses (σ_12_, σ_13_, and σ_23_,) that act parallel to planes.

34 $$\sigma_{ij}=\left[\begin{array}{lll} \sigma_{11} & \sigma_{12} & \sigma_{13}\\ \sigma_{21} & \sigma_{22} & \sigma_{23}\\ \sigma_{31} & \sigma_{32} & \sigma_{33} \end{array}\right]$$

Similarly, the strain tensor (ε*_ij_*), provided in eq 35, describes normal strains (relative elongations) and shear strains (angular distortions).

35 $$\varepsilon_{ij}=\left[\begin{array}{lll} \varepsilon_{11} & \varepsilon_{12} & \varepsilon_{13}\\ \varepsilon_{21} & \varepsilon_{22} & \varepsilon_{23}\\ \varepsilon_{31} & \varepsilon_{32} & \varepsilon_{33} \end{array}\right]$$

**Figure 70:**
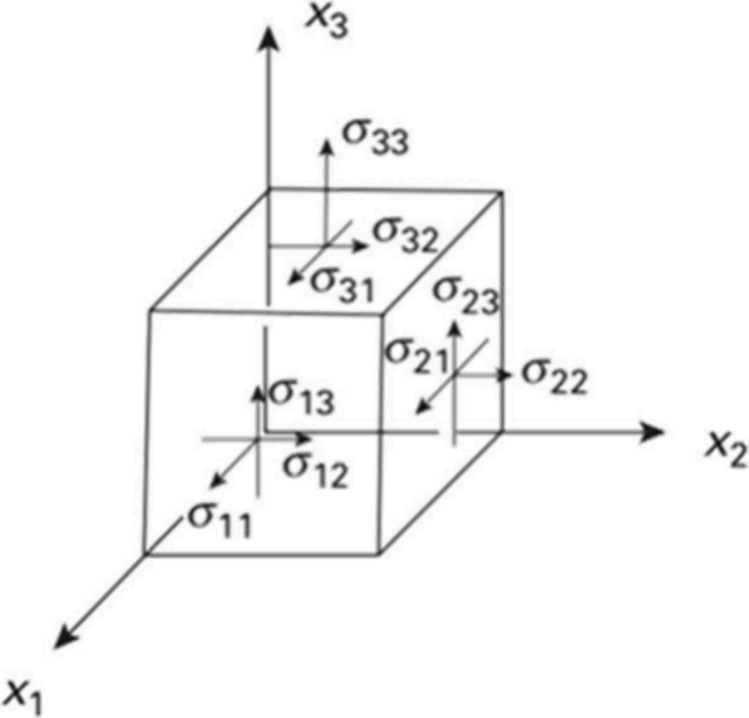
Illustration of the stress tensor components in a three-dimensional coordinate system, showing normal stresses (σ_11_, σ_22_, σ_33_) acting perpendicular to each plane, and shear stresses (e.g., σ_12_, σ_13_, σ_23_) acting parallel to the planes.

In tensorial notation, the indices *i* and *j* specify the directions of both the applied forces and the planes upon which these forces act.^559^ For a linear elastic (but anisotropic) material, the general constitutive law relating the stress and strain is shown in eq 36:

36 $$\sigma_{ij}=C_{ijkl}\varepsilon_{kl}$$

where *C*_*ijkl*_ is a fourth-order tensor analogous to the elastic modulus. Due to fundamental physical considerations (e.g., equilibrium of angular momentum), the stress and strain tensors are both symmetric, i.e., ε_*ij*_ = ε_*ji*_ and σ_*ij*_ = σ_*ji*_, thereby reducing the number of independent components in each tensor from nine to six.^560^ As such, the relationship between the stresses and strains for a linear elastic (anisotropic) material is often written in a contracted form as shown in eq 37:

37 $$\left(\begin{array}{l} \sigma_{1}\\ \sigma_{2}\\ \sigma_{3}\\ \sigma_{4}\\ \sigma_{5}\\ \sigma_{6} \end{array}\right)=\left(\begin{array}{llllll} C_{11} & C_{12} & C_{13} & C_{14} & C_{15} & C_{16}\\ C_{21} & C_{22} & C_{23} & C_{24} & C_{25} & C_{26}\\ C_{31} & C_{32} & C_{33} & C_{34} & C_{35} & C_{36}\\ C_{41} & C_{42} & C_{43} & C_{44} & C_{45} & C_{46}\\ C_{51} & C_{52} & C_{53} & C_{54} & C_{55} & C_{56}\\ C_{61} & C_{62} & C_{63} & C_{64} & C_{65} & C_{66} \end{array}\right)\left(\begin{array}{l} \varepsilon_{1}\\ \varepsilon_{2}\\ \varepsilon_{3}\\ \varepsilon_{4}\\ \varepsilon_{5}\\ \varepsilon_{6} \end{array}\right)$$

where *C*_*ij*_ is known as the stiffness matrix. The general stiffness tensor comprises 36 coefficients. However, from strain energy considerations, *C*_*ij*_ itself is symmetric, thereby reducing the number of independent elastic constants to 21 (e.g., for highly nonsymmetric (triclinic) single crystals).^561^ The number of elastic constants is further reduced for more symmetric crystal systems. For instance, orthorhombic crystals, which includes α-V_2_O_5_, possess higher symmetry and thus require only nine independent elastic constants. These constants directly correlate the applied stresses to induced strains as per eq 38, thereby enabling detailed mechanical predictions specific to V_2_O_5_’s crystallographic orientation.^562^

38 $$C_{ij}=\left[\begin{array}{llllll} C_{11} & C_{12} & C_{13} & 0 & 0 & 0\\ - & C_{22} & C_{23} & 0 & 0 & 0\\ - & - & C_{33} & 0 & 0 & 0\\ - & - & - & C_{44} & 0 & 0\\ - & - & - & - & C_{55} & 0\\ - & - & - & - & - & C_{66} \end{array}\right]$$

The orthorhombic symmetry of single-crystal α-V_2_O_5_, characterized by distinct lattice parameters along its crystallographic axes, leads to pronounced anisotropic mechanical behavior in various phases. As such, properties such as stiffness, hardness, and fracture toughness differ depending on crystallographic direction, especially in layered phases like α-V_2_O_5_, where strong in-plane covalent bonds contrast with weak interlayer van der Waals-type bonding. Collectively, these foundational concepts, including stress–strain relationships, tensorial representations, and elastic symmetry considerations form the basis for interpreting experimental measurements and predicting mechanical deformation, stress, and damage in single-crystal V_2_O_5_ systems.^563^

### 7.2 Phonon–Electron Interactions and their Role in the Mechanical Response of Single Crystals

Phonons and corresponding lattice vibrations are observable through techniques such as Raman or infrared spectroscopy and underly many mechanical properties of a given material by governing how a crystal absorbs, transmits, and dissipates energy. Namely, when an external load is applied to a material, either as static strain or dynamic loading, the atoms within the lattice respond via collective vibrations, and the nature of these vibrational modes dictates the stiffness, resilience, and damping characteristics of the material.^564–566^ These physics become particularly important under dynamic loading (i.e., vibration), thermal cycling, phase transformation, or repeated electrochemical insertion, in which mechanical fatigue and failure are directly linked to how efficiently the material dissipates vibrational energy. As such, understanding phononic behavior can link a material’s elastic response to time-dependent, cyclic phenomena like mechanical damping, phase-transformation-induced fatigue, and structural degradation.^25,567,568^

One critical mechanism connects phonons to a wide range of material responses is phonon–electron coupling. This fundamental process refers to the interaction between lattice vibrations and mobile charge carriers (electrons or holes) in a solid. When a lattice vibrates, the periodic potential experienced by the electrons is modulated, allowing energy to transfer between the phononic and electronic subsystems. This energy exchange plays a central role in determining electrical resistance (as electrons are scattered by thermally populated phonons) and thermal conductivity (as phonons scatter off electrons).^566,569^ From a mechanical perspective, phonon–electron coupling serves as a critical pathway for energy dissipation, enabling vibrational energy introduced into a crystal lattice through external stress, strain, or dynamic loading to be transferred into the electronic subsystem. In this process, the energy from lattice vibrations is absorbed by mobile charge carriers and then dissipated as heat, reducing the amount of energy stored elastically.^570,571^

This coupling effectively adds a nonelastic damping channel to the material, supplementing or even dominating over traditional mechanisms of energy loss such as viscoelasticity or anelasticity (delayed elastic response in metals and ceramics). In simple terms, upon mechanical disturbance/loading, a portion of the energy is transferred into the electron system, as opposed to storing the entirety of the energy like a spring. These electrons absorb vibrational energy and release it thermally, thus providing an efficient internal dissipation mechanism. The degree to which this phenomenon occurs depends on how strongly the vibrations of the atoms (phonons) interact with the movement of electrons, i.e., the strength of the phonon–electron coupling. This coupling can vary depending on several factors: the carrier concentration (how many mobile electrons or holes are present), the symmetry of the crystal lattice, and the availability of (energetically) nearby electronic states that make it easier for electrons to be excited by lattice vibrations. Materials with more available electronic states near the Fermi level or with localized electrons that strongly interact with lattice motion (such as vanadium oxides) tend to exhibit stronger coupling.^565,571,572^

In experimental terms, stronger coupling produces observable changes in the vibrational properties of the material. When phonons rapidly lose energy to the electron system, their lifetimes become shorter, which manifests as broader peaks in the Raman or infrared spectra. Likewise, the frequencies of the phonon modes can shift under strain, temperature, or doping, reflecting changes in bond stiffness or lattice symmetry due to energy exchange with electrons. These spectral features are direct evidence of internal energy dissipation pathways and serve as fingerprints of the underlying electron–phonon interactions that govern how the material responds to dynamic mechanical stimuli.^573–575^

This interplay becomes particularly critical under repeated or cyclic loading, such as in electrochemically cycled battery electrodes or phase-change switching devices. When subjected to such conditions, the lattice undergoes recurrent deformation, and phonon–electron coupling can lead to a gradual accumulation of microstructural damage. Over time, vibrational modes may broaden irreversibly, weaken in intensity, or shift permanently. This degradation reflects a loss of vibrational coherence as energy dissipation pathways become dominant, and the lattice structure deviates from its original symmetry or stability. This behavior has been observed in materials such as VO_2_, in which repeated metal–insulator transitions (e.g., as induced by cyclic heating/cooling) result in accumulation of strain and eventual crack formation, as well as in V_2_O_5_, in which electrochemical cycling has been shown to induce internal stress and fracture through repeated vibrational energy absorption and release.^330,576–578^

Phonon–electron coupling also contributes to mechanical hysteresis, the irreversible energy dissipation that manifests in “loop-like behavior” in plots of work-conjugate variable quantities, e.g., stress–strain curves, for instance during heating/cooling through phase transformations. In strongly coupled systems like VO_2_ and V_2_O_3_, where structural and electronic transitions are intertwined, each loading and unloading cycle does not trace the same path. Instead, a portion of the energy is lost in each cycle, manifesting as internal friction. This phenomenon is particularly important in applications such as neuromorphic computing, in which repeated phase changes are central to function. Here, phonon–electron coupling is not only responsible for triggering transitions, but also for shaping their energy path and mechanical reversibility.^576,578–580^

First-principles calculations have shown that the vibrational responses of vanadium oxides, particularly in V_2_O_5_, are highly sensitive to both dimensionality and long-range Coulomb interactions. For instance, Bhandari and Lambrecht demonstrated that LO–TO splitting and phonon mode polarization in V_2_O_5_ vary significantly between the bulk crystal and its monolayer form, thereby highlighting the role of anisotropic bonding and dielectric screening in modulating vibrational properties. This work has provided a critical foundation for understanding how phonon modes evolve under external perturbations such as strain, doping, or confinement, conditions commonly encountered during device operation and mechanical cycling.^581^

Recognizing these effects opens valuable pathways for materials engineering and design. By tuning phonon–electron interactions through chemical doping, strain engineering, or nanoscale structuring, the degree of damping can be controlled; fatigue resistance can be improved; and mechanical resilience can be enhanced as a whole. For example, W-doped VO_2_ exhibits reduced phase-transition hysteresis and enhanced cycling stability compared to its undoped counterpart. Similarly, composite architectures in V_2_O_5_ cathodes can aid in dissipating stress more uniformly. Overall, phonon interactions are critical to the mechanical life cycle of functional materials, especially those subjected to repeated loading or structural transformation, such as vanadium oxides.^554,582,583^

### 7.3 Mechanical Properties of V_2_O_5_ Single Crystals

Building on the anisotropic context discussed in the introduction to Section 7, this section examines the experimentally measured mechanical properties of V_2_O_5_ single crystals across various polymorphs. The anisotropy is most evident in α-V_2_O_5_, where the contrast between strong in-plane bonding and weak interlayer interactions produces a high degree of directional dependence in the mechanical response. As a result, α-V_2_O_5_ typically exhibits high stiffness and strength parallel to the layers but significantly reduced mechanical performance perpendicular to them. In contrast, γ- and δ-Li_*x*_V_2_O_5_, feature more three-dimensional bonding arrangements that induce more isotropic-type behavior.^9,330,554^ Although the mechanical behavior of α-V_2_O_5_ has been reasonably well studied, there is still a lack of data for other phases, which calls for further experimental studies. Additionally, given the structural differences among the polymorphs, it is essential to adopt a specific approach for the different phases that enables characterizing the nanomechanical properties from an anisotropic perspective to fully understand and describe the mechanical behavior of V_2_O_5_ single crystals.

#### 7.3.1 Hardness and Elastic Modulus

Nanoindentation studies indicate that polycrystalline α-V_2_O_5_ generally displays relatively low hardness (compared to other polycrystalline film measurements), typically ranging from 2 to 3 GPa.^551,554^ This relatively low hardness has been attributed to weak bonding between the layers. In contrast, thin-film samples containing dopants have exhibited substantially higher hardness values of up to 7 GPa.^551^ The “elastic modulus” (here we use the term loosely in generalizing for an anisotropic material) of α-V_2_O_5_ is highly anisotropic, with in-plane modulus values reaching approximately 220 GPa along the *b* axis but dropping to ∼70 GPa perpendicular to the layers.^554^

For other phases of V_2_O_5_, limited experimental data has suggested that δ-Li_*x*_V_2_O_5_, which forms through lithium intercalation, may (somewhat counterintuitively) show increased stiffness as a result of reduced interlayer spacing. By comparison, γ-Li_*x*_V_2_O_5_, which has a more distorted structure, is expected to exhibit intermediate mechanical properties and more isotropic mechanical response due its distorted stacking of layers, which partially reduces anisotropy. Nevertheless, further studies are needed to confirm these predictions.^458,584^

#### 7.3.2 Fracture Toughness and Failure Behavior

V_2_O_5_ single crystals are intrinsically brittle, with reported fracture toughness values typically in the range of 0.5–1.5 MPa·m^1^/^2^. In the orthorhombic α-V_2_O_5_ phase, fracture predominantly occurs through cleavage along (between) the (001) planes, where van der Waals interlayer interactions are significantly weaker than the covalent in-plane bonds, as illustrated in Figure 7b. This pronounced anisotropy facilitates crack propagation between the layers, making α-V_2_O_5_ particularly vulnerable to failure under tensile loading conditions.^585^

The fracture behavior varies notably among different polymorphs due to their distinct structural architectures. For instance, γ-Li_*x*_V_2_O_5_, which forms upon lithium intercalation, possesses a distorted layered framework with slightly enhanced interlayer bonding. Although still cleavage-prone, the distortion likely incresaes the specific energy required for crack initiation, suggesting improved fracture resistance relative to α-V_2_O_5_. However, experimental verification of this hypothesis is still lacking, as precise toughness measurements for this phase remain unavailable.^554,584^

More isotropic polymorphs such as δ-Li_*x*_V_2_O_5_ and ζ-V_2_O_5_ show comparatively enhanced mechanical robustness. δ-Li_*x*_V_2_O_5_ forms via significant structural reorganization during lithiation, reducing the prominence of well-defined cleavage planes. ζ-V_2_O_5_, characterized by its three-dimensional tunnel network with continuous V–O bonding (as shown in Figure 50c), demonstrates notably improved fracture resistance. This structural connectivity enhances stability under repeated electrochemical cycling, which makes it a promising candidate for applications requiring improved mechanical durability such as resistance to chemo-mechanical degradation.^586^

By comparison, polycrystalline V_2_O_5_ may exhibit slightly higher fracture resistance, primarily due to grain boundary interactions that can deflect or arrest propagating cracks; fracture can occur transgranularly (through grains) or intergranularly (along grain boundaries). The prevalence of intergranular fracture is related to weak grain boundary cohesion, often leading to premature mechanical failure under stress.^587,588^ However, all V_2_O_5_ polymorphs are fundamentally brittle and show limited plastic deformation and fracture resistance under mechanical stress.^554,589^

#### 7.3.3 On the Impact of Phase Transformations in Vanadium Oxides

Phase transformations in V_2_O_5_, especially those caused by lithium intercalation or external pressures, significantly impact their mechanical integrity. For instance, the transition from the α-phase to the δ-Li_*x*_V_2_O_5_ phase upon lithiation, involves a substantial volume expansion of approximately 11%. This expansion generates internal stresses that can induce mechanical damage, particularly fracture, of the material. Similarly, when subjected to high pressures of around 7 GPa, α-V_2_O_5_ transforms into a high-density δ-phase, which alters its mechanical properties by increasing the dimensionality of bonding.^554,590^

Of important related note, recent studies on vanadium dioxide (VO_2_) have demonstrated how mechanical stress and anisotropic deformation are intricately tied to crystallographic orientation during phase transitions. In VO_2_ thin films, metal–insulator transitions (MITs) induce either tensile or compressive stresses depending on the epitaxial growth conditions and the corresponding crystal orientation, ultimately affecting the film’s fracture resistance and mechanical stability. While VO_2_ and V_2_O_5_ differ structurally and electronically, this behavior highlights a fundamental principle that can also be applied to V_2_O_5_ in which the crystallography strongly governs how stress evolves during structural transformations. Additionally, these observations suggest that phase transformation-induced stress may be similarly orientation-dependent, with mechanical resilience varying across differently aligned single crystals.^9^

Understanding these transformations is crucial for applications, e.g., in energy storage systems in which repeated phase transformations during electrochemical cycling can result in mechanical degradation. Therefore, further research is needed to quantify the evolution of stress and fracture behavior in the different phases, especially in the context of crystal orientation and anisotropy.

### 7.4 Failure Mechanisms and Strategies for Improving Longevity

#### 7.4.1 Mechanical Degradation under External Stimuli

Lithium insertion into V_2_O_5_ induces significant mechanical stresses due to volumetric expansions and contractions associated with the phase transformations. The α-to-δ transformation typically proceeds through the intermediate ε-phase, with the overall process involving an approximate 11% volume expansion, generating large internal compressive stresses upon lithiation. Upon delithiation, the structure may experience tensile stresses depending on the extent of plastic deformation and residual strain accumulation, which can vary with cycling conditions. These cyclic stresses accumulate, promoting crack initiation, growth, and eventual material fragmentation, thereby deteriorating electrode integrity and electrochemical performance.^554^ In contrast, ζ-V_2_O_5_ accommodates lithium insertion via ionic rearrangement within its one-dimensional tunnel structure, thereby reducing volume changes and internal stress generation. This structural behavior significantly reduces the propensity for fracture compared to α-V_2_O_5_, thereby engendering superior mechanical and electrochemical durability over repeated cycling.^472^

High-pressure conditions can also induce structural transitions in V_2_O_5_, such as the α-to-δ transformation above ∼7 GPa, thereby resulting in permanent microstructural changes or amorphization upon removal of the pressure. These irreversible structural transformations can introduce microcracks, compromising mechanical integrity.^47,586,591^

Thermal cycling also affects fracture behavior due to anisotropic thermal expansion and mismatch in thermal expansion with nearby materials (e.g., underlying substrates). Differential thermal expansion within V_2_O_5_ crystals generates stress concentrations, potentially driving crack initiation and growth. This issue becomes particularly relevant in battery electrodes, where localized heating during cycling exacerbates mechanical degradation.^592^

#### 7.4.2 Strategies for Improving Fracture Resistance

Enhancing the fracture resistance of V_2_O_5_ can be achieved through a combination of structural engineering approaches aimed at mitigating its inherent brittleness and anisotropic failure tendencies. One of the most promising methods is chemical doping, which involves introducing cations such as Ti, Nb, Cu, or Si into the V_2_O_5_ lattice. These dopants strengthen interlayer interactions, reduce cleavage susceptibility, and promote improved fracture toughness. Similarly, studies on VO_2_ have demonstrated that substitutional doping with W, Ge, Cr, Fe, or Ti not only modifies the electronic phase stability but also influences the mechanics of phase transitions by shifting the metal–insulator transition temperature (*T*_eq_), altering nucleation barriers, and modifying defect landscapes.^560,593,594^ Depending on the valence state and ionic radius of the dopant relative to vanadium (e.g., V^4+^ or V^5+^), the resulting structural distortions can either stabilize or destabilize polymorphs and alter the structural mismatch or lattice incoherence between the two polymorphs potentially leading to reduced transformation-induced stress and enhanced mechanical durability.^595,596^ The underlying principles of reducing misfit strain across phase transforming materials are transferable and highlight a promising pathway for tuning the mechanical and electrochemical resilience of V_2_O_5_ through targeted compositional modifications.^267,594,597,598,463^

In addition to doping, nanostructuring represents an effective strategy for limiting crack propagation and enabling strain relaxation. By synthesizing V_2_O_5_ in nanoscale morphologies—such as nanowires, nanobelts, or thin platelets—researchers have observed reduced fracture susceptibility due to the confinement of stress accumulation and increased tolerance for localized deformation. These structures inherently possess shorter crack propagation paths and improved load distribution, which together contribute to enhanced damage tolerance under repeated cycling.^9^

Composite formation offers another powerful route to mechanically stabilize V_2_O_5_. Integrating the material with ductile or flexible matrices, including polymers, carbon nanotubes, or graphene oxide creates heterogeneous interfaces that act as effective barriers against crack initiation and propagation. Crack deflection, bridging, and energy dissipation at these interfaces provide synergistic improvements in both mechanical and electrochemical longevity. These benefits are particularly relevant in battery applications, where V_2_O_5_-based composites have demonstrated marked enhancements in cycle life and resistance to mechanical degradation.^599,600^

Lastly, prelithiation strategies, which involve the intentional insertion of lithium or other alkali ions (e.g., Na^+^, K^+^) prior to battery cycling, help reduce the amplitude of volumetric changes that occur during lithiation/delithiation. By minimizing the mechanical stresses associated with these transitions, prelithiated electrodes show superior structural integrity and longer operational lifetimes, especially in high-rate cycling scenarios.^463,554,601,602^ Collectively, these approaches offer substantial promise for overcoming the inherent brittleness of V_2_O_5_ and enhancing its long-term mechanical stability in practical applications.

### 7.5 Experimental Techniques for Mechanical Characterization

Due to the typically small dimensions and brittleness of V_2_O_5_ single crystals, mechanical characterization requires advanced microto-nanoscale testing techniques. Traditional bulk mechanical testing methods, such as bulk tension and compression testing, are typically impractical because of the fragility and limited size of the crystals. Instead, techniques such as nanoindentation, micropillar compression, microcantilever bending, and microbeam tensile testing have provided valuable insights into the mechanical properties of V_2_O_5_ at small scales.^603,604^ Additionally, multibeam optical stress (MOS) measurements have enabled real-time monitoring of stress evolution (e.g., during electrochemical cycling, heating, etc.), particularly in thin films, and offer insights into single-crystal behavior more generally.^605^

#### 7.5.1 Nanoindentation

Nanoindentation is a widely employed technique for characterizing mechanical properties and behavior in single crystals. In this method, a stiff (typically diamond) indenter, commonly with a sharp Berkovich, conical, or spherical tip, is pressed into the crystal surface under precisely controlled loading conditions while monitoring the resulting load and displacement. From this raw data, various properties can be extracted, primarily that of the elastic modulus and hardness (which is related to the yield strength) of the material. Specifically, the Oliver–Pharr method is most commonly employed, as it can directly obtain mechanical properties such as hardness and elastic modulus from the resultant load versus displacement curve.^606^

Berkovich indentation is particularly advantageous for testing single crystals due to its sharp triangular pyramid geometry, which produces clearly defined indentation impressions and facilitates understanding of anisotropic behavior (particularly in terms of fracture behavior). This sharp geometry often allows for a detailed examination of the response of the material (e.g., fracture) along specific crystallographic orientations. As such, it is suitable for capturing subtle differences in mechanical behavior related to directional bonding characteristics inherent in layered crystals such as V_2_O_5_.^607^ SEM images in Figure 71a–d demonstrate anisotropic analysis through nanoindentation, with indents performed along varying crystallographic orientations to investigate direction-dependent fracture behavior at (b) 0°, (c) 30°, and (d) 60° of rotation. Cross-sectional SEM analysis (Figure 71e) of a Berkovich indent reveals subsurface deformation, crack morphology, and internal defects induced by the indentation process.

**Figure 71:**
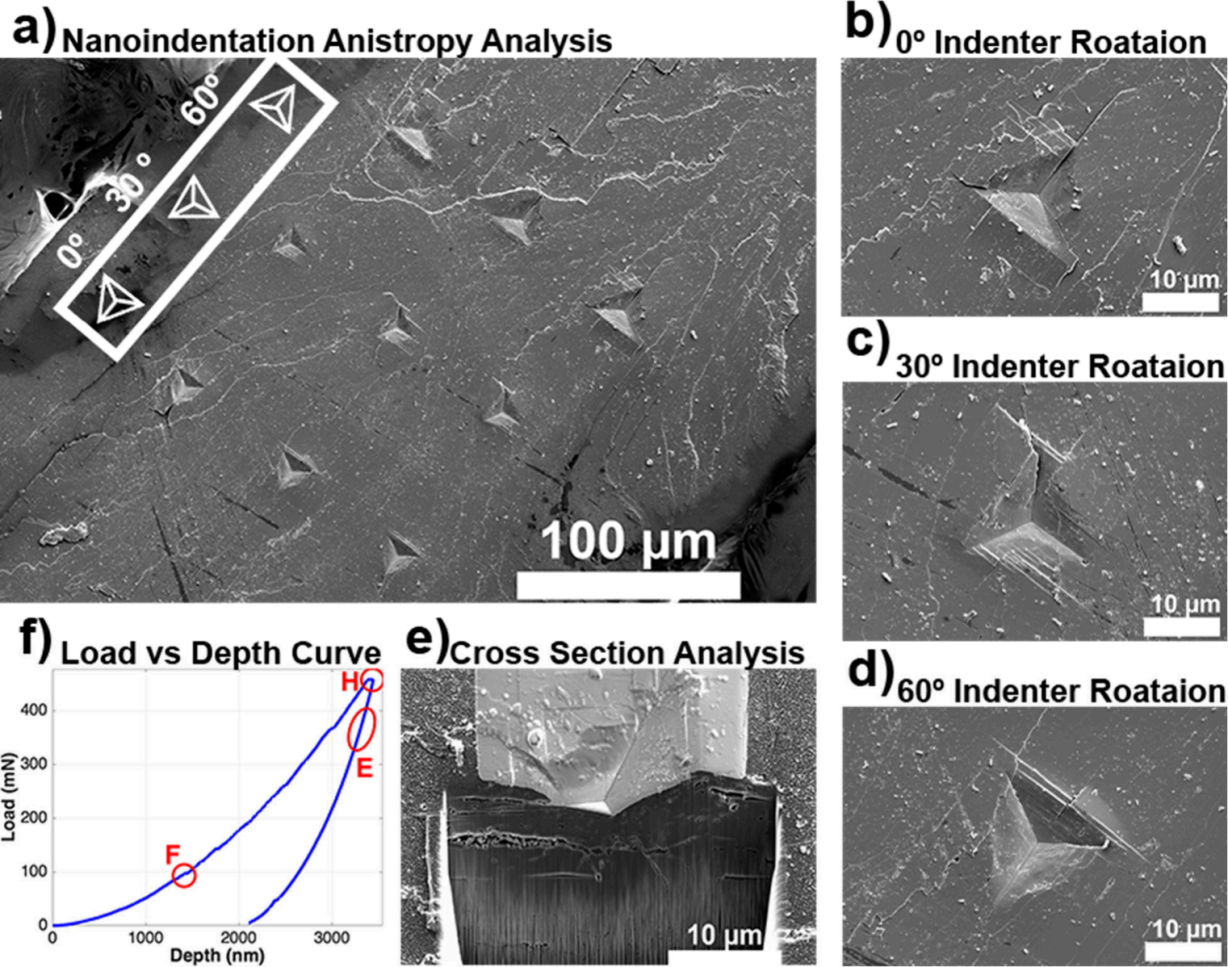
Nanomechanical characterization methods applied to testing of α-V_2_O_5_ single crystals. (a) SEM image demonstrating anisotropic analysis by nanoindentation at 0°, 30°, and 60° rotation angles, magnified in panels (b)–(d), respectively. (e) SEM Cross-section and (f) load–displacement curve of a Berkovich indent, with circled regions in (f) representing (E) elastic modulus, (H) hardness, and (F) fracture events or “pop-ins”.

By comparison, spherical nanoindentation has its strengths, primarily in probing elastic-plastic transitions. However, for materials such as V_2_O_5_, which predominantly undergo brittle fracture rather than plastic deformation (see Figure 71d), spherical indentation typically reveals discrete fracture events (normally called “pop-ins”) as sudden displacement bursts in load–displacement curves (see Figure 71f). These pop-ins are critical indicators of crack initiation or fracture beneath or around the indenter tip and thus offer valuable insights into brittle fracture processes characteristic of layered oxide crystals.^608^

Experimental considerations such as meticulous surface preparation to minimize roughness and artifacts are essential to obtaining reliable and repeatable indentation data. Moving forward, further studies involving orientation-dependent nanoindentation, temperature-controlled tests, and detailed analysis of pop-in phenomena would advance the understanding of deformation and fracture behavior across different polymorphs of V_2_O_5_.

#### 7.5.2 Micropillar Compression Testing

Micropillar compression is an effective technique for evaluating the stress–strain response and failure mechanisms of single crystals. This method utilizes focused ion beam (FIB) milling to create micron-scale pillars from single-crystal samples, which are then subjected to uniaxial compression using a flat punch nanoindenter. This approach allows for direct measurement of yield strength, fracture strength, and deformation mechanisms. Figure 72b shows representative engineering stress–strain curves obtained from micropillar compression tests performed on single-crystalline Cu_6_Sn_5_ micropillars. Insets (b) and (c) show SEM images of micropillars at different stages of deformation, revealing the micropillar geometry and surface morphology prior to fracture. Inset (d) highlights a slip plane observed after deformation, indicative of slip-induced plastic deformation occurring prior to final brittle failure.

**Figure 72:**
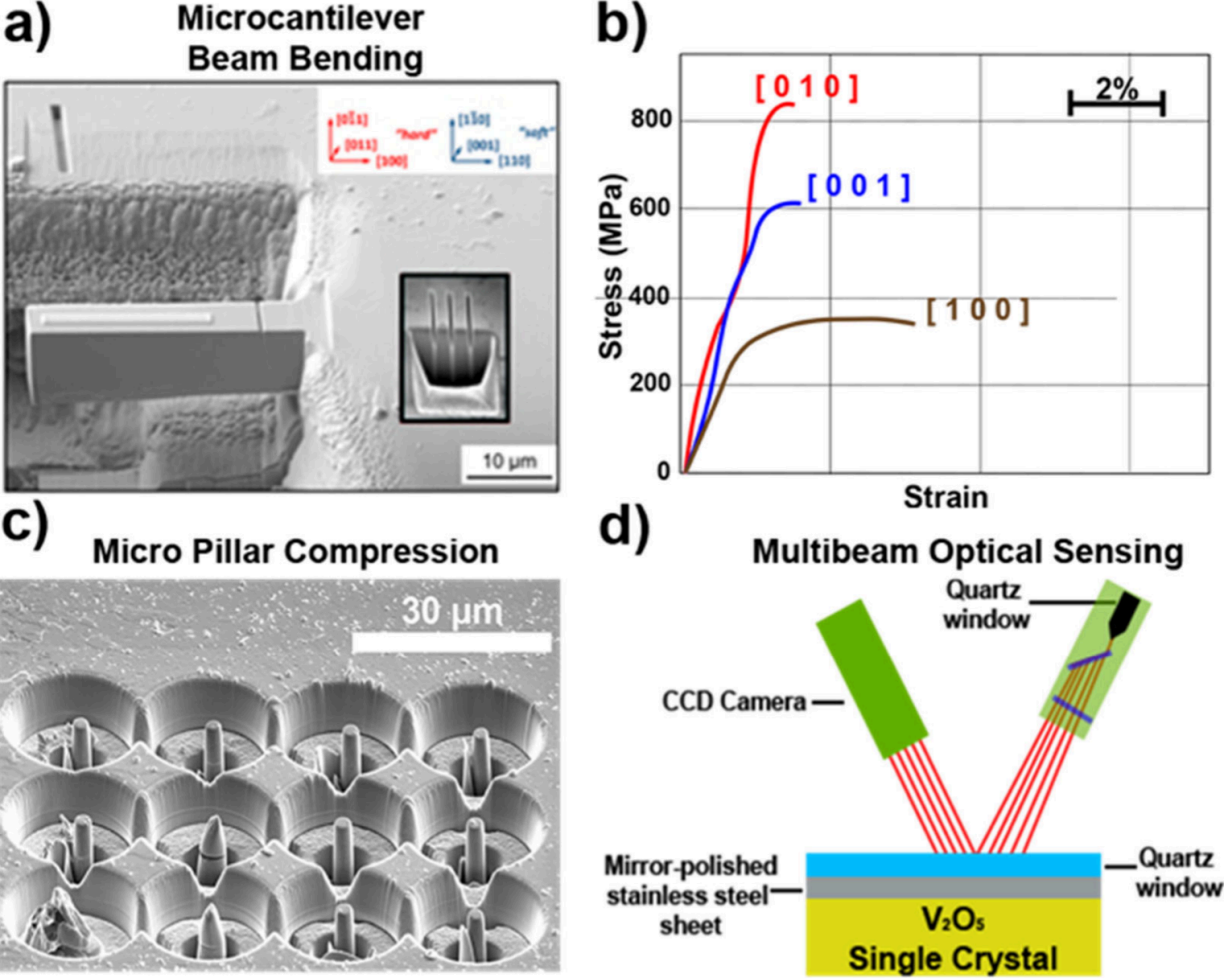
Microscale mechanical testing methods used to obtain stress–strain responses and extract mechanical properties of V_2_O_5_ single crystals. (a) SEM image of microcantilever bending experiments conducted on NiAl single crystals. (b) Schematic representations of stress–strain curves from micromechanical testing (e.g., micro pillar compression and microcantilever beam bending) along three principal crystallographic directions [010], [001], and [100], showing representative mechanical anisotropy inherent to V_2_O_5_ single crystals. (c) SEM image of micropillar compression test specimens fabricated via focused ion beam (FIB) milling of a V_2_O_5_ single crystal. (d) Schematic illustration of multibeam optical stress sensor (MOS) measurements for real-time, noncontact monitoring of stress evolution in V_2_O_5_ single crystals while being subjected to external stimuli, such as electrochemical cycling. Panel (a) reproduced with permission from reference 609. Copyright 2014 Springer Nature.

While micropillar compression has been successfully applied to other layered materials such as Ga_2_O_3_ and SiC, its use for single-crystal V_2_O_5_ is still in early stages.^610,611^ The SEM image in Figure 72c showing micropillar compression test specimens fabricated by FIB milling of a V_2_O_5_ single crystal that are designed to measure compressive strength, deformation, and fracture properties. In layered materials such as α-V_2_O_5_, micropillar compression is expected to reveal significant anisotropy in mechanical behavior. When loaded (e.g., in tension) perpendicular to the layers, the pillars often fail by splitting along the weak van der Waals interfaces, indicative of a cleavage-dominated fracture. In contrast, in-plane loading typically exhibits higher strength due to the presence of stronger covalent bonding. Preliminary studies suggest that γ- and δ-Li_*x*_V_2_O_5_ may display different deformation mechanisms due to their altered structural connectivity, but further experimental validation is necessary.^43,444,612^

#### 7.5.3 Microcantilever Bending (Fracture)

Fracture toughness measurements of V_2_O_5_ single crystals remain scarce, but microcantilever bending tests offer a valuable method for assessing crack propagation resistance. In this approach, FIB milling is used to fabricate microscale cantilever beams from single crystals. A notch is introduced to serve as a site for crack initiation, and the cantilever beam is then bent by pushing on the end of the beam with a nanoindenter. The critical load needed for crack propagation is recorded to determine fracture toughness. Figure 72a illustrates microcantilever bending experiments conducted on NiAl single crystals, providing an illustrative example of the specimen geometry and notch positioning. Microcantilevers were fabricated by focused ion beam (FIB) milling along specific crystallographic orientations designated as “soft” [110] and “hard” [100]. Notches were precisely introduced to control the crack initiation point, which facilitated the study of fracture toughness anisotropy and the evaluation of crack propagation behavior through subsequent *in situ* SEM observations and mechanical testing.

For α-V_2_O_5_, fracture is expected to occur preferentially along the (001) planes due to weak interlayer bonding. The estimated fracture toughness (*K*_IC_) ranges from 0.5 to 1.5 MPa·m^1/ 2^, which confirms the brittle nature of the material.^585^ Though this technique has been successfully applied to other layered oxides, direct application to V_2_O_5_ single crystals is still underexplored.

#### 7.5.4 Multibeam Optical Stress (MOS) Testing

Multibeam optical stress (MOS) testing is a noncontact technique used to measure stress evolution in thin films (e.g., during thermal cycling, electrochemical cycling, etc.). In this method, an array of laser beams reflects off a reflective substrate coated with a thin film of the material of interest, depicted schematically in Figure 72d. Changes in the spacing of the reflected beams correspond to changes in the substrate’s curvature, which can then be quantitatively related to the average stress in the film using Stoney’s equation. This is given by eq 39

39 $$\sigma_{f}=\frac{E_{s}h_{s}^{2}}{6\left(1-v_{s}\right)h_{f}R}$$

where σ_f_ is the average biaxial stress in the film, *E*_s_ and *v*_s_ are the Young’s modulus and Poisson’s ratio of the substrate, respectively, *h*_s_ is the substrate thickness, *h*_f_ is the film thickness, and *R* is the radius of curvature of the substrate. The equation assumes that the film is significantly thinner than the substrate and that the substrate remains within the elastic deformation regime. This experimental setup enables precise, real-time characterization of stress generation arising from external influences such as fabrication processes (e.g., sputtering), thermal gradients, or ion insertion/extraction during battery operation. In the context of V_2_O_5_, MOS has proven particularly valuable for understanding the buildup of stress during lithiation-induced phase transformations and provides insight into how such stress contributes to eventual mechanical degradation.

MOS has proven particularly useful for studying stress evolution in thin-film V_2_O_5_, especially during electrochemical cycling of V_2_O_5_ cathodes of Li-ion batteries. Studies have indicated that upon lithiation, α-V_2_O_5_ experiences a buildup of compressive stress owing to layer expansion (and the corresponding constraints induced by the underlying substrate). This stress accumulates over multiple charge–discharge cycles and can eventually induce crack formation and mechanical degradation. During phase transitions, such as the α→ε, ε→δ, and δ→γ transformations (see Figure 48), abrupt changes in stress are observed, correlating with variations in lattice parameters and volumetric expansion or contraction as displayed in Figure 73.^554,605,613^

**Figure 73:**
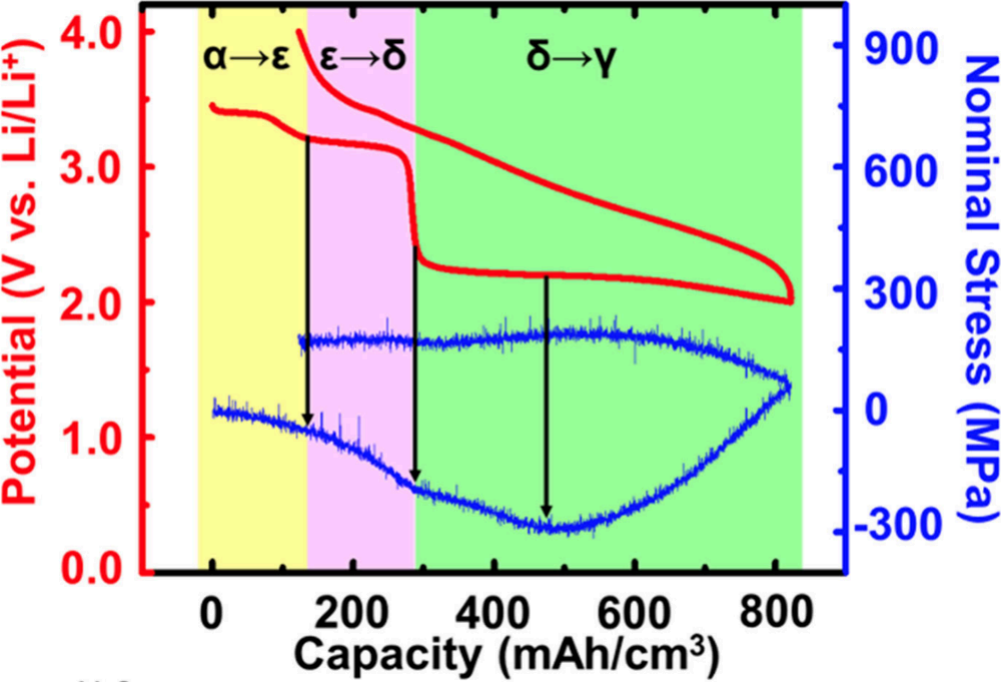
In situ stress and potential evolution of thin-film V_2_O_5_ during lithiation, measured using multibeam optical stress (MOS) technique. The red curve shows the electrochemical voltage profile versus capacity, while the blue curve indicates the corresponding nominal stress evolution. Distinct jumps (in the slope of stress with respect to capacity, i.e., with respect to lithium concentration) are observed during phase transformations from α→ε, ε→δ, and δ→γ, reflecting abrupt volumetric changes associated with lithium intercalation. MOS enables real-time, noncontact quantification of these stress changes, offering valuable insight into chemo-mechanical coupling in thin-film V_2_O_5_ cathodes. Figure reproduced and modified with permission from reference 554. Copyright 2019 Royal Society of Chemistry.

Although MOS has been primarily used to characterize polycrystalline V_2_O_5_, its findings can provide indirect insights into single-crystal behavior, particularly regarding how mechanical stresses develop during phase transformations. Figure 72d portrays a schematic illustration of multibeam optical stress sensor for real-time, noncontact monitoring of stress evolution in V_2_O_5_ single crystals while being subjected to external stimuli, such as electrochemical cycling. However, applying MOS directly to single crystals requires further methodological adaptations, such as specialized substrate-free sample preparation, transfer printing, or single-crystal epitaxial growth onto substrates (over large areas). Future research should explore how MOS measurements correlate with direct mechanical testing of single crystals of V_2_O_5_ under electrochemical or temperature-dependent cycling conditions.

### 7.6 Mechanical Properties as an Integral Element of Materials Design

The mechanical properties of V_2_O_5_ single crystals represent a critical yet relatively underexplored aspect of their performance, particularly for energy storage, electrothermal neurons and synapses, and other advanced technological applications. Their distinctive layered atomic structures engender significant anisotropy, leading to direction-dependent elastic modulus, strength/hardness, and fracture toughness. Moreover, their inherent brittleness remains a substantial obstacle to practical implementation. Gaining a comprehensive understanding of fracture mechanisms, phase-specific responses, and the influence of external stimuli, such as lithium intercalation, pressure-induced transitions, and electrothermal cycling is thus essential for optimizing their mechanical integrity during use-case scenarios.

Experimental strategies focusing on microscale testing, chemical doping, nanostructuring, and composite formation offer viable pathways to substantially enhance mechanical resilience. Site-selective modification with elements such as silicon, niobium, or titanium can strengthen interlayer bonding, significantly reducing susceptibility to cleavage. Nanostructuring techniques, including fabricating V_2_O_5_ nanowires and nanobelts, facilitate improved strain relaxation and reduce internal stress accumulation during electrochemical cycling. Additionally, composite materials incorporating V_2_O_5_ with flexible substrates or ductile matrices enhance fracture resistance by obstructing crack propagation through mechanisms like crack deflection and bridging.

Despite these promising approaches, critical research gaps persist. Direct fracture toughness measurements across different V_2_O_5_ polymorphs remain limited, underscoring the need for systematic phase-specific characterization as millimeter-sized single crystals become accessible. Future research should prioritize these direct mechanical measurements, coupled with *in situ* visualization of crack initiation and propagation under realistic cycling conditions, to reveal real-time degradation mechanisms. Moreover, advanced structural engineering approaches—such as strain-tolerant microstructures, controlled defect engineering, and/or utilizing fabrication approaches that induce advantageous residual stresses/strain (e.g., compressive stress/strain)—could further enhance mechanical durability without compromising functionality.

Integrating experimental methodologies with computational tools, including molecular dynamics simulations and phase-field modeling, will also yield predictive insights into fracture processes and mechanical stability under diverse operating conditions. Ultimately, addressing these challenges through a combined experimental and theoretical approach will be critical for advancing the mechanical reliability of V_2_O_5_ single crystals, thereby facilitating integration of mechanical anisotropies as a design principle in next-generation energy storage devices, neuromorphic elements, and other demanding technological environments.

## 8 Conclusions and Outlook

In the preceding sections, we discuss myriad aspects of structure–function correlations deciphered for vanadium oxides using single crystals as a distinctive lens to understand and rationalize structural preferences, electronic structure, electronic instabilities, diffusionless and distortive structural transformations, mobility of point defects and guest ions, extended defects, surfaces, and interfaces in binary, ternary, and more complex vanadium oxides. Atomic resolution understanding of bulk, defect, surface, and interfacial atomistic and electronic structure derived from single crystals has provided extensive insight into functional properties such as electron, ion, and phonon transport as well as mechanical properties. The rich diversity of compositions and structures that can be accessed across the rugged free energy landscapes of vanadium oxides makes them a valuable sandbox for investigating the strong coupling of spin, charge, orbital, lattice, and atomic degrees of freedom and their implications for functional properties. These properties in turn map to numerous applications with the emphasis here on neuromorphic computing, electrochemical energy storage, and catalysis.

In comparison to polycrystalline thin films or powders, single crystals enable more direct measurement of the intrinsic properties of a material. As highlighted in Figure 1, the consistent crystal orientation allows the anisotropy of a material’s response to external stimuli to be probed directly. Ideal single crystals contain no domain boundaries which disrupt transport, and their single-domain nature allows properties to be measured without ensemble averaging. In the next few sections, we provide a future perspective of gaps, challenges, and opportunities in the study of single crystals of vanadium oxides. The critical roadblock has been the growth of high-quality crystals with extended “clean” surfaces, which would enable deployment of the entire suite of modern photon, neutron, ion-beam, electron beam probes as well as scanning probe techniques to determine average bulk and surface structure and to track transformations of structure as a function of state variables, ion insertion, or coupling to external fields. High-quality single crystals are also imperative to understand facet selectivity of the topochemical processes and catalytic transformations noted above and for heterointegration to form device stacks. We explore future perspectives in five broad categories: (a) crystal growth—addressing foundational opportunities for obtaining high-quality crystals; (b) surfaces of single crystals and implications for chemical reactivity and heterointerfaces; (c) control of disorder and defect dynamics; (d) reversible ion insertion; and (e) applications in brain-inspired computing including neuronal and synaptic emulation;

### 8.1 Inverse Design Strategies for Crystal Growth

The synthesis of crystalline materials with targeted properties has traditionally relied on empirical knowledge and experimental trial-and-error approaches. Recent advances in machine learning (ML) and computational modeling have fundamentally transformed this paradigm. Artificial intelligence and ML methods offer predictive capabilities and prospects for dramatically accelerating materials discovery from relatively sparse experimental data. While AI/ML methods stand poised to reshape materials science from the perspective of materials design (and discovery), crystal structure prediction, and process optimization/intensification,^614–618^ in this section, we have primarily emphasized prediction, optimization, and mechanistic understanding of crystal growth.^81,619^

Machine learning-enabled inverse design (Figure 74) represents a paradigm shift in crystal engineering, where computational tools predict synthesis pathways based on desired target structures or properties. Unlike conventional forward approaches that map synthesis conditions to outcomes, inverse design allows researchers to specify target properties and receive guidance on viable synthesis routes from computational simulations or mining of literature data and experimental libraries. This approach is particularly valuable for accessing metastable polymorphs that occupy challenging regions of free energy landscapes as sketched in Figure 47. Accessing such polymorphs requires navigating rugged energy landscapes along specific reaction trajectories without an excess of energy that would allow the system to relax to the equilibrium structure. For example, Ren and colleagues employed a gradient-boosted decision tree model to predict synthesis conditions for ferroelectric HfO_2_ thin films, enabling precise control over critical parameters including precursor ratios, annealing temperatures, and dopant concentrations.^620^ Their model accurately predicted conditions yielding the metastable orthorhombic phase (*Pca*2_1_) responsible for ferroelectric behavior while suppressing competing monoclinic and tetragonal phases. Similar inverse design strategies have proven effective across diverse material classes, including metal–organic frameworks (MOFs),^621^ perovskites,^172,622,623^ and functional ceramics.^624^ Notably, Moosavi et al.^625^ demonstrated that convolutional neural networks (CNNs) could predict synthetic accessibility of hypothetical MOF structures by learning structural patterns associated with successful synthesis, effectively guiding computational screening toward experimentally realizable candidates.

**Figure 74:**
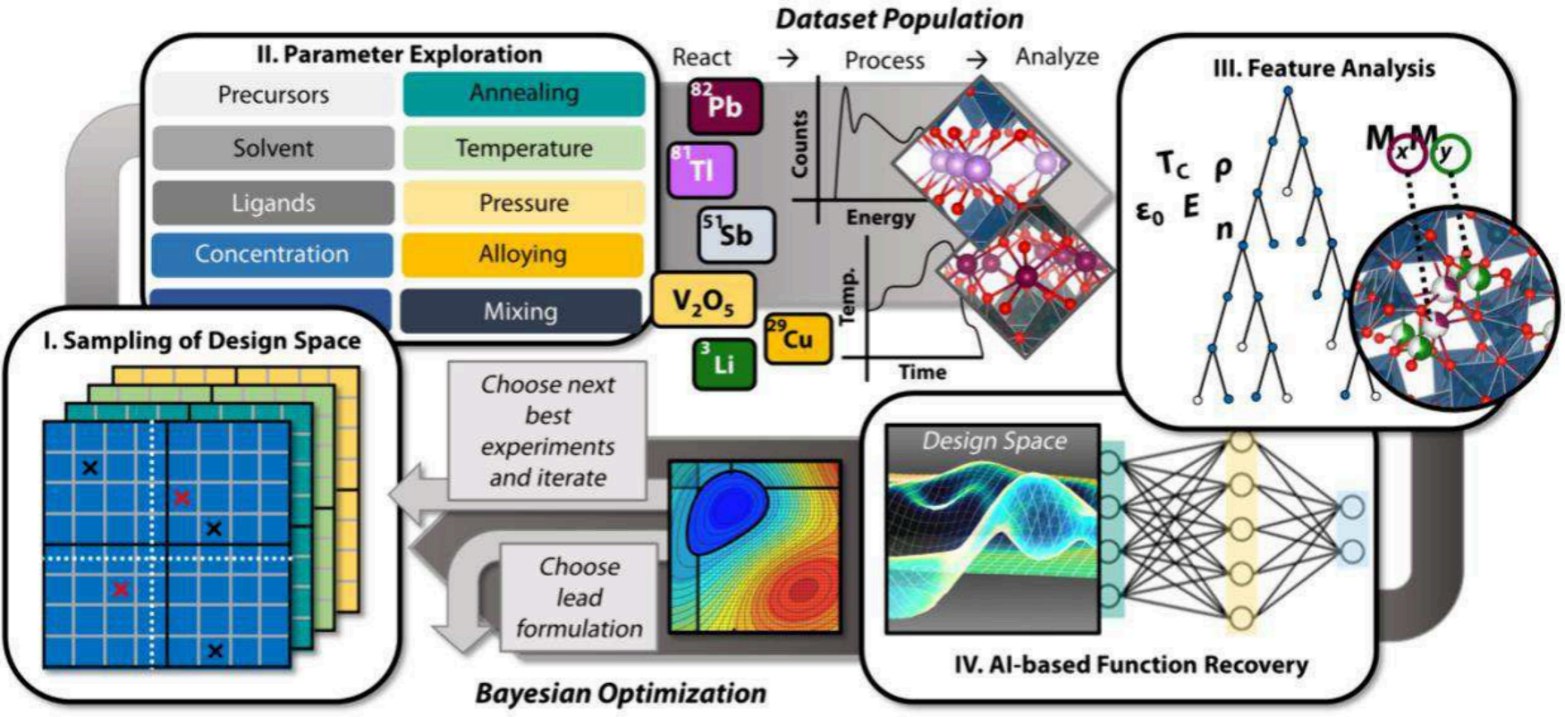
Schematic depiction of a machine-learning workflow for the iterative exploration and exploitation of synthetic design space for identifying active variables that determine structural motifs and crystal structure outcomes in a range of metastable *M*_*x*_V_2_O_5_ and *M*_*x*_ *M*′_*y*_V_2_O_5_ polymorphs. Reproduced with permission from reference 11. Copyright 2022 American Chemical Society.

#### 8.1.1 Efficient Exploration of High-Dimensional Parameter Spaces

Crystal synthesis typically involves numerous interdependent variables—temperature profiles, precursor concentrations (of course, choice of the precursor and its reaction/decomposition pathways), pH conditions, additive selections, and processing parameters—which creates vast parameter spaces that would be exceedingly challenging to explore exhaustively through conventional one-variable-at-a-time approaches. AI/ML methods address this challenge through systematic sampling and response surface mapping and can be connected in active learning loops to high-throughput experimentation such as to enable efficient navigation.

Design of Experiments (DOE) methodologies strategically sample parameter space to maximize information gain while minimizing experimental burden. When coupled with machine learning algorithms, these approaches efficiently navigate complex synthesis landscapes. For instance, Mekki-Berrada et al. employed fractional factorial designs with Gaussian process regression to optimize gold nanorod synthesis, revealing nonintuitive relationships between silver nitrate concentration, reducing agent concentration, and the temperature that controlled aspect ratio development.^626^

Bayesian optimization has also emerged as a particularly powerful approach for crystallization process development due to its ability to balance exploration (testing novel conditions) with exploitation (refining promising regions). Several researchers have demonstrated this capability in the context of perovskite quantum dot synthesis, where an autonomous platforms such as combining robotic experimentation or microfluidic arrays with Bayesian optimization, which has identified synthesis conditions yielding precise control over nanocrystal thickness and photoluminescence properties after exploring only a small fraction of the available parameter space.^627,628^ Multiobjective optimization approaches further extend these capabilities by addressing competing priorities in crystal engineering. Researchers have extensively worked on developing a multiobjective Bayesian optimization framework for pharmaceutical crystal form screening that simultaneously optimized for thermodynamic stability, dissolution rate, and processability metrics.^629–631^

#### 8.1.2 Feature Engineering and Mechanistic Insight Generation

Beyond prediction and optimization, machine learning approaches can decipher critical descriptors governing synthesis outcomes, thereby providing mechanistic insights that extend beyond empirical correlations. This capability addresses a fundamental challenge in crystal engineering such as identifying which combination of synthetic parameters most strongly influences specific structural or morphological features.

Support vector machines (SVMs) and random forest models have proven particularly effective for feature importance analysis in crystallization processes. This approach has aided in elucidation of structure-directing roles of organic additives in biomineralization,^632^ identified critical supersaturation thresholds in continuous crystallization processes,^633^ and mapped solvent-dependent polymorphic transitions in pharmaceutical compounds. In each case, machine learning-derived feature importance metrics provided insights that would have been challenging to obtain through conventional experiments alone.

The effectiveness of machine learning approaches intrinsically depends on data quality and quantity. This is a major challenge in crystal engineering where generating comprehensive data sets can be resource intensive. Several strategies have emerged to address these limitations. For instance, automated experimental platforms including microfluidic systems,^634^ flow reactors,^635^ and robotic synthesis units generate high-throughput data to train robust models.^636^ These platforms systematically vary synthesis parameters while collecting multimodal data (spectroscopic, diffraction, imaging) to build comprehensive structure-synthesis relationships. Such data is perhaps best parsed and navigated in active learning loops through physics- and chemistry-informed ML models that incorporate theoretical constraints and domain knowledge to guide predictions with limited data. For example, Feng et al. demonstrated that neural networks incorporating free energy terms as regularization constraints could predict nucleation behavior of organic crystals with significantly smaller training data sets than conventional black-box approaches.^637^ In systems with extreme data sparsity such as to predict synthetic strategies for heretofore unknown compounds, transfer learning strategies leverage knowledge from related materials systems to inform predictions about novel compounds. This approach has proven particularly valuable for predicting synthesis parameters across chemical families, as demonstrated by Jensen and colleagues who developed a transferable model for predicting MOF crystallization conditions across diverse metal centers and organic linkers.^638^

#### 8.1.3 Implementing AI/ML in Growth of Vanadium Oxide Single Crystals

Section 2 presented an extensive compendium of methods used to grow single crystals of binary and ternary vanadium oxides. AI/ML approaches provide interesting prospects for improving crystal growth strategies, thereby enabling growth of higher quality crystals. Effective active learning loops will require multiscale models that bridge atomic-level phenomena with macroscopic crystallization behavior. Integrating quantum mechanical calculations, molecular dynamics simulations, and continuum models with machine learning frameworks will provide more comprehensive predictive capabilities. The issue that is ever-present with machine learning-based predictive methods is the requirement for substantive data sets so that the model can accurately extract trends based on the given training set that can be applied to an unseen test set. Overfitting, describing when a machine learning model becomes familiar with the training data hindering its generalizability, becomes a significant problem in cases with small data sets. The limited availability of high-quality data sets is a prevalent issue when attempting to predict the growth conditions of vanadium oxide single crystals. Some interesting workarounds have been proposed in the literature to overcome this issue. Banad et al., endeavored to build an inverse design framework to accelerate the discovery of stable vanadium oxides, and instead addressed the data limitation issue by developing a “systematic data generation pipeline.”^639^ This involved implementing a targeted substitution approach in which existing metal oxides were substituted with vanadium to produce a new data set containing only binary vanadium oxides whose feasibility were subsequently examined through first-principles formation energy calculations.^639^ Utilizing this data set with a variational autoencoder, which preserves the structural and chemical characteristics of the input data, and a generative adversarial network, new hypothetical structures were generated. The final stability of each structure was evaluated through phonon calculations to determine the dynamic stability of each structure. The result was 91 computationally stable and 41 computationally metastable structures. However, the existence and synthetic viability of the predicted metastable structures has yet to been experimentally verified. While successful, it is important to note that this work aimed to identify novel crystal structures and not their synthetic conditions. Predicting synthetic conditions using a training set obtained from a targeted substitution approach would be difficult due to inherent differences in elemental precursors, melting points, and general reactivity.

A second method, whose data could come directly from vanadium oxide-based syntheses, that has been proposed is to utilize the results from “dark” reactions—failed or successful syntheses—to bolster a data set.^640^ While this approach has not yet been applied to vanadium oxides, the intrinsic difficulty and multiple steps in reaction optimization required for obtaining single crystals of metastable vanadium oxides would likely generate data sets that could be used for training machine learning models.

Prediction of temporal evolution remains challenging, particularly for understanding nucleation events, complex growth kinetics, and polymorph transformation pathways. Simplified attempts at this have been made to develop machine learning models to predict successful synthetic pathways. For example, a Random Forest-based model was constructed to predict parameters for the hydrothermal synthesis of VO_2_ (B) by utilizing training set variables such as precursor to V_2_O_5_ ratio, concentration of V_2_O_5_, filling ratio of the hydrothermal vessel, reaction temperature, and reaction time. The resulting synthesis suggested by the machine learning model did yield the desired VO_2_ (B) phase.^641^ The main drawback to this approach is that these models require an abundance of synthetic data to build a viable training data set, which would not be available for metastable phases whose synthesis is poorly understood and under-explored. One interesting method to construct a physics-informed machine learning model would be to use active learning as a tool to navigate Pourbaix diagrams. This approach would intrinsically be more computationally and experimentally expensive as the metastable phases of vanadium oxide exist in a smaller, constrained phase space and would therefore require much higher levels of precision and accuracy. Fortunately, this level of fine control is more readily enabled by machine learning/AI methods. The resulting model would essentially be trained to identify “needles in a haystack,” but would represent a powerful means toward crystal growth prediction.

Advanced time-series machine learning models and recurrent neural networks show promise for capturing these dynamic processes and can potentially be informed by establishing physics- and chemistry-informed frameworks based on *operando* studies of synthesis processes. Expanded understanding of crystal growth mechanisms gained for select systems such as through synchrotron X-ray scattering and spectroscopy methods can be deployed in transfer learning formats to predict growth pathways. Notwithstanding increasing reliance on high-throughput methods, informed by robotic discovery processes that have gained considerable traction in protein crystallography, uncertainty quantification in synthetic predictions will be essential for practical implementation, particularly for high-value materials where experimental validation is resource-intensive. Autonomous discovery platforms that seamlessly integrate computation, robotics, and *in situ* characterization represent the ultimate expression of machine learning-guided synthesis. Early demonstrations of such closed-loop systems have shown promise for navigating complex synthesis spaces.^636,642^ The utility of such methods will be amplified by advances in generative design such as enabled by large language models. As these frontiers are explored, the empirical art of growing single crystals of vanadium oxides can be transformed to a predictive science, enabling targeted access to structures with tailored properties. The promise of AI/ML-augmented inverse design lies not just in the discovery and crystallization of new materials but also in accessing growth conditions that yield crystals with large dimensions and of unprecedented quality.

### 8.2 Surfaces of Vanadium Oxide Single Crystals

The extended-ordering of single crystals enables them to serve as an exceptional platform for understanding fundamental materials properties. Well-defined surface facets provide the opportunity to interrogate surface atomistic and electronic structure as well as defect and interface dynamics with the help of a broad expanse of modern surface science methods. For instance, as a result of challenges with availability of high-quality single crystals, there is only a relatively limited set of high-quality scanning tunneling microscopy studies of surface reconstructions and interfaces of vanadium oxides.^511,643–645^ Large single crystals with atomically well-defined “clean” surfaces would provide direct insight not just into reconstructions but also into specifics of surface defect structures, their temperature and field-dependent dynamics, binding sites and associated energetics of adsorbates (as is critical for understanding catalytic transformations mediated by vanadium oxides, ion insertion, and topochemical modification),^646^ and electronic structure modulation. A specific requirement herein would be vacuum cleavability as is indeed now readily accessible for several of the layered polymorphs described above (such as λ-V_2_O_5_ and intercalated phases shown in Figures 47 and 58).

Beyond STM studies of well-defined interfaces, many surface spectroscopy probes such as angle-resolved photoemission spectroscopy (ARPES), resonant inelastic X-ray scattering, and X-ray standing wave methods require clean surfaces to visualize intrinsic material properties and to follow structural and electronic transformations. The availability of high-quality single crystal surfaces would yield unprecedented insights into electronic structure and its dynamical evolution. Such understanding can be challenging to glean from first-principles density functional theory calculations given strong electron correlation effects that are extensively modulated across phase transformations.

Single crystals with well-defined clean surfaces further provide opportunities for single-entity studies based on extraction of lamellae using focused ion beam or other sectioning methods. Such surfaces can further be patterned using lithography methods to interrogate fundamental effects of dimensionality, surface strain, and geometry on surface atomistic and electronic structure and its evolution with ion flux, adsorbates, or coupling to external fields. Indeed, mechanically interlocked metamaterials constructed from single crystals using additive manufacturing or two-photon lithography methods hold great promise for highly mechanically resilient architectures.^54,647^ Similarly, different facets can exhibit vastly distinctive catalytic activity as exemplified in the photocatalytic activity exhibited by BiVO_4_, where the surface structure strongly modifies charge separation, excited state lifetime, and binding energetics of catalytic intermediates.^648–650^

From the perspective of practical applications, clean single crystal surfaces would enable exploration of vanadium oxides as substrates for epitaxial growth. The vast diversity of structures described in the review tunable through control of oxygen stoichiometry as well as identity and stoichiometry of inserted ion(s) affords a broad range of surface structures that can be used to grow other transition metal oxides, mixed oxyanion compounds, or altogether different compounds with varying degrees of epitaxial strain. As a glimpse of the possibilities in this regard, epitaxial matching of V_2_O_3_/HfO_2_ interfaces has enabled stabilization of the metastable cubic polymorph of HfO_2_ under ambient conditions, which is otherwise only accessible above 2600 °C.^83^ Multilayered stacks with topotactic or epitaxial relationships afford interesting opportunities for ion transport that could enable interesting electrochemical random access memory (EC-RAM) concepts as pioneered by Talin and all solid-state battery configurations with well-defined interfaces.^651,652^

### 8.3 Control of Disorder and Defect Dynamics

With a near perfect ordered starting configuration of atoms, single crystals provide an ideal model design space for multimodal characterization of defect structure and dynamics. Single crystals provide an opportunity to develop a deeper understanding of defect-driven behaviors across different dimensionalities. Single crystals and epitaxial films are ideal substrates wherein ion implantation, exposure to high-energy radiation, electrochemical gating, or mechanical stress can be used to selectively induce defects—from oxygen vacancies and vanadium interstitials to line dislocations and grain boundaries. Oxygen vacancies and interstitial ions and the selective evolution of disorder and its correlations can be monitored through techniques such as diffuse scattering, muon spectroscopy, and X-ray/neutron total scattering.^653,654^

Both oxygen vacancies and vanadium interstitials as well as implanted ions strongly modify the electronic phase diagrams of compounds such as VO_2_ by altering the relative thermodynamic stability of low- and high-temperature phases and by providing sites where transformations can be nucleated.^655^ Indeed, the hysteresis of the monoclinic—tetragonal phase transformation in VO_2_ depends strongly on the defect concentration in general and specifically on the concentration of oxygen vacancies.^319,656^ Single crystals provide a means of precisely correlating defect type and concentration to specific modifications of transformation characteristics and for evaluating defect interactions with the lattice and one another.^646^

Extended defects such as dislocations and grain boundaries provide a means to engineer crystal architectures to selectively guide ion flux and modulate stress gradients. As described in Section 7, anisotropies in mechanical properties and lithium diffusion require explicit consideration of mechanical properties as design elements in the construction of electrochemical devices. Single crystals of vanadium oxides are critical linchpins to understand direction-dependent elastic moduli, strength/hardness, and fracture toughness and their evolution as a function of ion insertion or coupling to external fields. Eliciting detailed understanding of mechanisms of plastic deformation, phase- and polymorph-specific mechanical response, and the evolution of mechanical properties with external stimuli, such as ion-insertion/deinsertion, electrothermal cycling, and interface coupling is thus essential for designing mechanically resilient electrochemical devices.

As introduced in Section 7, several critical research gaps remain to be addressed. Direct fracture toughness measurements are unavailable beyond a few polymorphs of V_2_O_5_ and are imperative as millimeter-sized single crystals become available. Such measurements coupled with *in situ* and *operando* visualization of plastic deformation can provide valuable insights into more useful utilization of mechanical properties as design elements. Single crystals provide a distinctive opportunity to image nucleation and growth of lithiation hotspots, strain fields, and dislocation networks across surfaces and interfaces.^450^ These materials further provide a platform for designing and validating advanced structural engineering approaches—such as strain-tolerant mechanically interlocked microstructures, controlled defect placement, and/or utilizing nanofabrication approaches to introduce advantageous residual stresses/strain (e.g., compressive stress/strain)—to deterministically drive ion fluxes and further enhance mechanical durability without compromising functionality. For instance, the rational incorporation of low-energy interfaces such as twin grain boundaries, and the exploitation of coherent dislocation networks to promote hierarchical structuring, present compelling routes to optimize conductivity, capacity retention, and mechanical stability.

### 8.4 Prospects for Ion Insertion

Some of the first examples of atomic resolution mapping of ion diffusion pathways through single-crystal-to-single-crystal transformations derive from different V_2_O_5_ polymorphs and V_6_O_13_ as discussed at some length in Section 5. Just as the ability to crystallize proteins and nucleic acids paved the way to macromolecular crystallography and the determination of high-resolution structures of biologically relevant motifs, enabling the development of detailed structure–function correlations and launching a new paradigm of drug design, atomic resolution views of ion diffusion pathways can inform site-selective modification strategies to enhance diffusion kinetics and increase energy density. Analogous to the design of targeted approaches to modulate protein and nucleic acid function by binding of small molecules in specific pockets or by specific modifications of amino acid sequences, single crystal electrochemistry provides a power lens for atomistic design of intercalation electrodes used in energy storage.

Where much of the current knowledge of diffusion pathways is derived from mesoscale mapping of lithiation inhomogeneities using electron and X-ray microscopy at larger length scales (providing particle- and electrode-level information), topochemical and electrochemical single-crystal-to-single-crystal transformations provide atomic resolution views of ion diffusion pathways in solids as exemplified in Figures 51 and 58. An urgent imperative is to examine topochemical insertion of other ions into large single crystals to develop an angstrom-resolution atomistic view of the diffusion pathways traversed by other charge carriers and to accordingly develop site-selective modification strategies such as preintercalation, pillaring, and lattice expansion to increase the concentration of accessible sites and ease site-to-site migration.

Based on the rugged energy landscapes of vanadium oxides and the distinctive ion diffusion pathways defined by [VO_5_]/[VO_6_] polyhedral connectivity in different polymorphs, an intriguing prospect is the relative rates of diffusion for different ions. For instance, the tunnel-structured ζ-V_2_O_5_ polymorph (Figure 53) shows several orders of magnitude Li/Na selectivity and even specific selectivity for ^6^Li over ^7^Li, which suggests intriguing means of hybrid capacitive deionization that could be precisely tailored based on atomic resolution structures of different insertion hosts derived from single-crystal diffraction.^473,657^

Scarce little has been accomplished thus far in imaging insertion reactions at single crystal surfaces and to examine propagation of intercalation fronts through single crystals. Such measurements combining imaging and scattering methods would provide insights into the role of surface reactions in initiating ion insertion processes, especially with regards to the role of extended defects and possible approaches to direct mesoscale ion diffusion pathways through defects and coupling to crystal structure anisotropies.

### 8.5 Applications in Brain-Inspired Computing

The strongly nonlinear dynamical modulation of conductance across a broad range of vanadium oxides derives specifically from their infinite chains of edge-sharing [VO_6_] octahedra where V3*d–*V3*d* interactions are at the cusp of being able to mediate metallic transport. Insulator–metal transitions are triggered by thermal activation or in response to an electric field. In contrast, corner- and face-sharing [VO_6_] octahedra in binary and ternary vanadium oxides have V–V distances above and below a critical crossover distance. The nonlinear conductance switching manifests as negative differential resistance (NDR) in current-controlled circuits under specific circumstances. When coupled to intrinsic or extrinsic resistors and capacitors, the NDR behavior can be utilized within specific “edge-of-chaos” regimes to obtain electrothermal neurons with self-sustaining oscillations as discussed in Section 4.

The frequency and amplitude of the self-sustaining oscillations of electrothermal neurons depend on intrinsic properties of the active element, as well as its dimensions, interfaces, and external circuit elements such as capacitors and resistors.^366,404^ A recent series of analytical frameworks and compact models have identified key intrinsic material properties that serve as necessary conditions for manifesting stable periodic oscillations across a range of circuit conditions.^404,416^ The forward design framework identifies three distinctive properties: (i) the extent of conductance nonlinearity resulting upon thermal activation (or the thermal coefficient of resistivity); (ii) specific heat capacity of the active element (which determines its thermal gradient with respect to the surroundings); and (iii) the ratio between electrical and thermal conductivity in the region that has the sharpest thermally activated change of resistivity.^416^ As these fundamental design principles formulated in terms of material properties have become available, they provide a means of linking back to structure and composition. Specifically, site-selective modification such as engendered through alloying on cation or anion sublattices or incorporation of interstitial dopants can modulate transformation characteristics and substantially alter regimes where periodic oscillations can be sustained. Mapping site-selective modification across binary vanadium oxide Mott–Peierls systems and predominantly Mott M_*x*_V_2_O_5_ to specific transformation characteristics of the active element and then to oscillator characteristics represents a key gap and opportunity (Figure 43).

Signal amplification needs only a single state variable such as temperature.^411^ A simple periodic oscillator requires coupling of two state variables.^386,416^ Chaotic oscillations and biological-type action potentials require at least three state variables. The Hodgkin-Huxley mechanism for neuron signaling has four state variables based on three types of Na-ion gates and one type of K-ion gates. In general, with more state variables, more complex behaviors can be elicited from a system (e.g., bursting). Similar to Na- and K-ion channels in biological systems, cointercalated systems with multiple ions occupying their distinctive sites (single crystals of quinary vanadium bronzes are now accessible)^306^ represent intriguing systems for eliciting high-fidelity neuronal emulation based on the migration of multiple ions. Explicitly using ion concentration as a state variable or as a means of imbuing synaptic response also represents an intriguing opportunity. This will require systems where ion concentration induces a nonlinear modulation of conductance, which requires that the mobilities and the diffusion constants change the local concentrations on a “reasonable” time scale of ms or shorter. Shear transformations commonly engendered by ion insertion in layered vanadium oxides thus provide a possible means of providing an additional state variable and for eliciting both neuronal and synaptic characteristics as observed in EC-RAM. Another possibility for surfaces of single crystals is to use the Ridley effect of symmetry breaking by forming a high current density conduction channel in the realm of negative differential resistance across single crystal oscillators. This essentially adds a new net state variable—one for the high density conduction channel and one for the low current density “background,” which are coupled together.^399^ Single crystals of vanadium oxides are thus critical to enabling a new paradigm of inverse and forward design linking complex brain-inspired function modalities to structure and composition.

The preceding sections have provided an illustrative compendium of opportunities enabled by the vast compositional and structural diversity of single crystals of vanadium oxides and their distinctive role in elucidating mechanisms of structural transformations, electronic instabilities, defect dynamics, and ion transport. Improvements in crystal quality and size will further expand the scope of fundamental studies as well as potential applications and provide an exemplary “sandbox” for disentangling structure–function correlations in strongly correlated systems.
